# C-Linked Glycomimetic Libraries Accessed by the Passerini Reaction

**DOI:** 10.3390/ijms20246236

**Published:** 2019-12-10

**Authors:** Kristina Vlahoviček-Kahlina, Josipa Suć Sajko, Ivanka Jerić

**Affiliations:** Division of Organic Chemistry and Biochemistry, Ruđer Bošković Institute, Bijenička cesta 54, 10000 Zagreb, Croatia; kristina.vlahovicek-kahlina@irb.hr (K.V.-K.); jsuc@irb.hr (J.S.S.)

**Keywords:** multicomponent reactions, carbohydrates, diastereoselective synthesis, glycosylation, C-glycosides

## Abstract

Carbohydrates and their conjugates are the most abundant natural products, with diverse and highly important biological roles. Synthetic glycoconjugates are versatile tools used to probe biological systems and interfere with them. In an endeavor to provide an efficient route to glycomimetics comprising structurally diverse carbohydrate units, we describe herein a robust, stereoselective, multicomponent approach. Isopropylidene-protected carbohydrate-derived aldehydes and ketones were utilized in the Passerini reaction, giving different glycosylated structures in high yields and diastereoselectivities up to 90:10 diastereomeric ratio (d.r). Access to highly valuable building blocks based on α-hydroxy C-glycosyl acids or more complex systems was elaborated by simple post-condensation methodologies.

## 1. Introduction

Carbohydrates are a ubiquitous group of natural products that mediate numerous, highly diverse cellular events and, consequently, participate in development of various diseases. Synthetic carbohydrates and their derivatives are powerful tools in biology and medicinal chemistry with diverse applications ranging from diagnostics to vaccines and therapeutics [1,2,3,4]. Moreover, carbohydrates represent attractive chiral synthons susceptible to diverse modifications and transformations, a highly desirable feature in diversity-oriented synthesis [5] and in the synthesis of chiral catalyst systems [6] or of noncovalent systems built upon weak interactions [1]. Carbohydrates are still a relatively untapped pool of new therapeutics [7,8]; however, advances in the functional understanding of carbohydrate-involving biological processes enabled carbohydrates to take over a prominent role in drug design. A particular group of compounds, glycomimetics, has emerged in the response to the growing demand for highly specific structures aimed to probe biological processes or interfere with them [9]. Klebe and Diederich described structurally rigid and metabolically stable lin-benzoguanines functionalized with isopropylidene-protected carbohydrate moieties as superior ligands for tRNA-guanine transglycosylase, the enzyme associated with *Shigella flexneri*-caused infection [10]. According to the DrugBank [11], there are 14 approved drugs bearing the isopropylidene motif in their structure; however, such structures are generally neglected in drug development.

**Figure 1:**
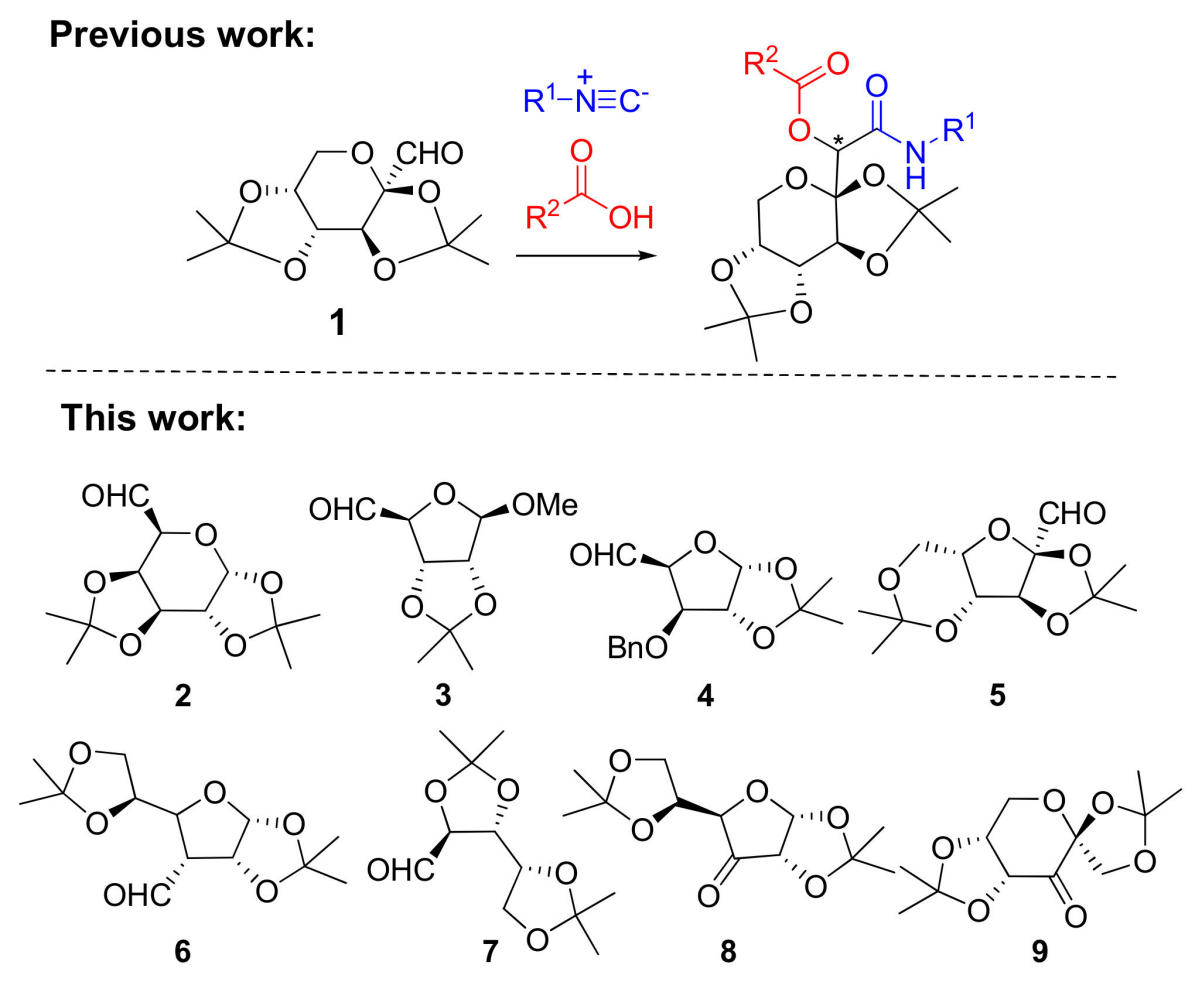
The selection of isopropylidene-protected aldehydes and ketones used as carbonyl components in the Passerini reaction.

As a part of our ongoing project aimed to enrich the chemical space of natural product-like compounds, we have recently reported a strategy to access a library of densely functionalized glycomimetics. We utilized, for the first time, bis-isopropylidene-protected d-fructose-derived aldehyde **1** (Figure 1) in the Passerini reaction with various acids and isocyanides [12]. Although carbohydrate-derived aldehydes were reported to affect diastereoselectivity in multicomponent reactions (MCRs), observed stereoselectivity was highly influenced by OH-protecting groups (acetyl or benzyl protecting groups were frequently used) and other components in MCRs [13]. Contrary to that, all reactions performed with bis-isopropylidene-protected aldehyde **1** yielded glycomimetics in high yields and diastereoselectivities up to diastereomeric ratio (d.r.) 94:6. Therefore, to validate the methodology, in this work, we expanded the pool of carbohydrate-derived carbonyl compounds to obtain Passerini products adorned with structurally different carbohydrate units. We were also intrigued by the possible role of the rigid isorpopylidene protecting group(s) in the diastereoselective outcome of the reactions. Moreover, an important advantage of the selected carbohydrate derivatives is the fixed anomeric configuration, since the presence of multiple anomers often hampers the purification and characterization of carbohydrate-related compounds.

We selected commercially available hexoses and pentoses in pyranose, furanose, or open chain forms, easily convertible into aldehyde or keto-derivatives by oxidation with Dess−Martin periodinane. Six aldehydes were utilized in this study (Figure 1): 1,2:3,4-di-*O*-isopropylidene-α-d-galactopyranose-derived aldehyde **2**, methyl-2,3-*O*-isopropylidene-β-d-ribofuranoside-derived aldehyde **3**, 3-*O*-benzyl-1,2-*O*-isopropylidene-α-d-xylofuranose-derived aldehyde **4**, 2,3:4,6-di-*O*-isopropylidene-α-l-sorbofuranose-derived aldehyde **5**, 1,2:5,6-di-*O*-isopropylidene-α-d-allofuranose-derived aldehyde **6**, and 2,3:4,6-di-*O*-isopropylidene-d-arabinose-derived aldehyde **7**. In addition, to complement our selection of carbohydrate-related carbonyl components, two ketones were envisaged: derivatives **8** and **9**, obtained from 1,2:5,6-di-*O*-isopropylidene-α-d-glucofuranose and 1,2:4,5-di-*O*-isopropylidene-β-d-fructopyranose, respectively (Figure 1).

## 2. Results and Discussion

**Figure 2:**
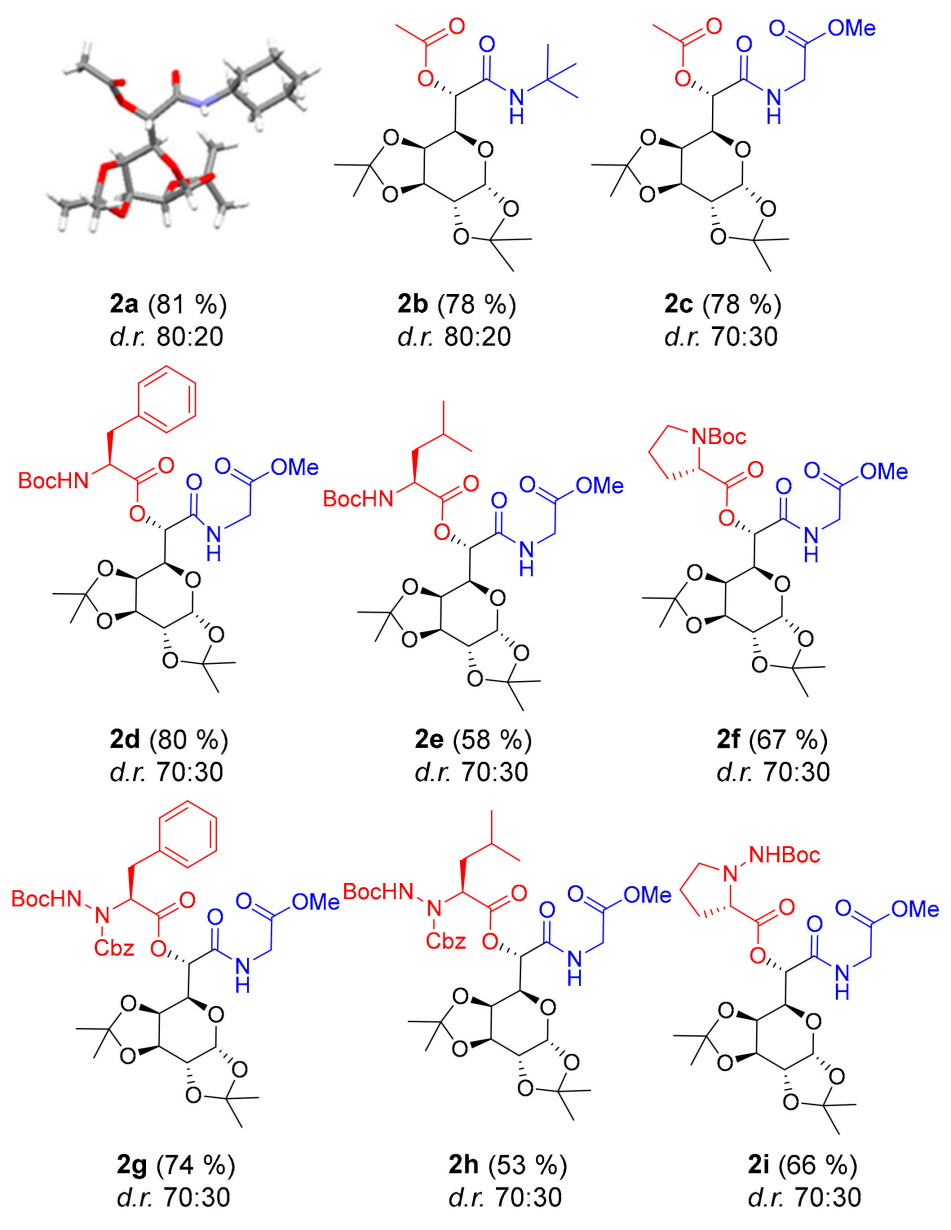
Passerini products obtained with aldehyde **2**. Isolated yields are given in parentheses. Diastereomeric ratios (d.r.) were determined by ^1^H NMR analysis of the reaction mixtures.

Our initial experiments were performed with aldehyde **2**, and a collection of examples is listed in Figure 2. All reactions were performed in dichloromethane (DCM) at room temperature until the consumption of the aldehyde, typically 6–24 h. Passerini reactions with acetic acid and cyclohexyl isocyanide, *tert*-butyl isocyanide, or methyl isocyanoacetate furnished the desired Passerini products **2a**–**c** in very good yields (~80%, Figure 2). The ratio of two diastereoisomers, determined by ^1^H NMR spectroscopy of the reaction mixture, was 80:20 d.r. for compounds **2a** and **2b** and 70:30 d.r. for **2c**. Next, we expanded the scope of the acid components by utilizing *N*-terminally protected amino acids. Gratifyingly, Boc-protected phenylalanine, leucine, and proline in the reaction with methyl isocyanoacetate produced depsitripeptides **2d**–**f** in very good yields (58–80%). In earlier work from our group, we successfully used α-hydrazino acids in the Passerini reaction to obtain hydrazino depsipeptides, a novel class of backbone-extended peptidomimetics [14]. Therefore, we performed Passerini reactions with *N*^α^-Cbz-, *N*^β^-Boc-protected hydrazino acids related to phenylalanine, leucine, and proline and obtained the corresponding hydrazino depsitripeptides in very good yields (53–74%). The ratio of two diastereoisomers, ~70:30 d.r. remained constant through all reactions performed with methyl isocyanoacetate, with the exception of **2f**, for which lower diastereoselectivity can be attributed to a somewhat problematic purification. We managed to isolate the major diastereoisomer of compound **2a** and prepared crystals suitable for X-ray analysis (Supplementary Materials). The configuration of the new stereocenter formed in the Passerini reaction was determined as *S*, as found for products obtained with fructose-derived aldehyde **1** [12].

**Figure 3:**
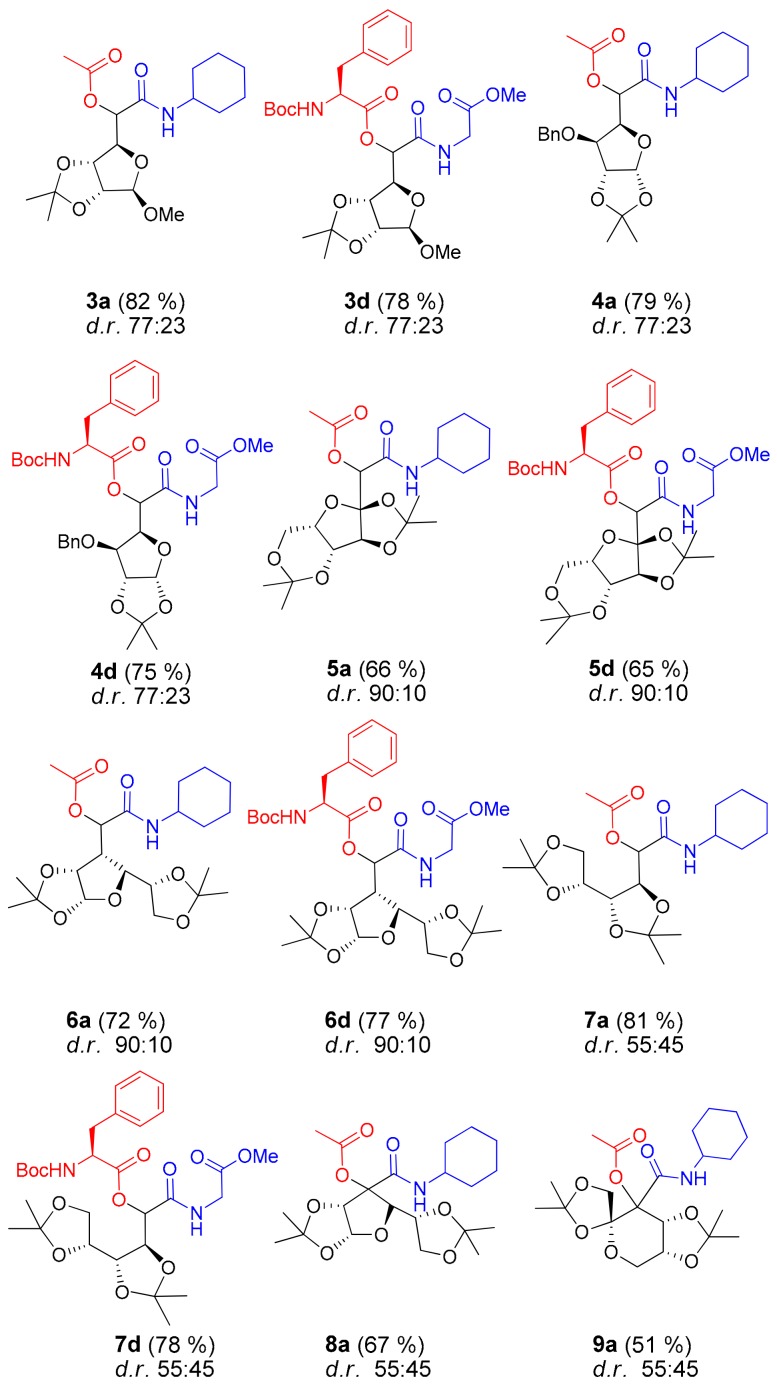
Passerini products obtained with aldehydes and ketones **3**–**9**. Isolated yields are given in parentheses; d.r. was determined by ^1^H NMR analysis of the reaction mixture.

Encouraged by the highly consistent results obtained with aldehyde **2**, we proceeded to examine the Passerini reaction with carbonyl compounds **3**–**9**. In all further experiments, two groups of acid–isocyanide components were used: a “simple” acetic acid–cyclohexyl isocyanide combination and an “amino acid-derived” combination with Boc-protected phenylalanine as the acid component and methyl isocyanoacetate (glycine-derived isocyanide). Such selection enabled us to ascertain the influence of sugar-derived carbonyl compounds on the yield and diastereoselectivity of the reaction, as well as the contribution of other components. To our satisfaction, Passerini reactions performed with ribofuranoside-derived aldehyde **3** gave the products **3a** and **3d** in high yields, i.e., 82% and 78%, respectively (Figure 3). Unlike aldehyde **2**, the ratio of two diastereoisomers was the same for the two combination of reactants, i.e., 77:23 d.r., determined by ^1^H NMR spectroscopy of the reaction mixtures.

Comparable results were obtained with xylofuranose-derived aldehyde **4**, with high yields (79% for **4a** and 75% for **4d**) and moderate diastereoselectivity (77:23 d.r.). We then turned our attention to sorbofuranose-derived aldehyde **5**, which can be considered a furanose equivalent of the fructose-derived aldehyde **1**. The reaction turned out to be efficient with both groups of acid–isocyanide components, giving Passerini products **5a** and **5d** in satisfactory yields (~65%, Figure 3). More importantly, the reactions performed with aldehyde **5** were highly diastereoselective (90:10 d.r.), as previously observed with aldehyde **1** [12]. Further study was directed toward allofuranose-derived aldehyde **6**, with a carbonyl group attached to the C3 position of the furanose ring. We were pleased to find that reactions proceeded with very good yields, i.e., 72% for **6a** and 77% for **6d**, and again with high diastereoselectivity, i.e., 90:10 d.r. Riva et al. reported Lewis acid catalyzed Passerini reactions on chiral aldehydes derived from desymmetrized erythritol with diastereoselectivities up to 92:8 [15]. This report prompted us to examine aldehyde **7**, obtained by oxidation of 2,3:4,6-di-*O*-isopropylidene-d-arabinose, under our Passerini reaction conditions. The reactions proceeded smoothly, giving the products **7a** and **7d** in very good yields (~80%, Figure 3), but stereoselectivity was disappointing, corresponding to 55:45 d.r.. Riva et al. used 0.4 equiv. of Lewis acid (ZnBr_2_) to improve the diastereoselectivity with erythritol-derived aldehydes; however, our attempts to affect stereoselectivity by adding a Lewis acid were unsuccessful. A possible explanation can be the presence of only one dioxolane ring in erythritol-derived aldehydes, where chelation of the metal occurred by the carbonyl oxygen and one of the dioxolane oxygens [15], while bis isopropylidene-protected aldehyde **7** possesses multiple binding sites for the Lewis acid. Finally, to complete our pool of sugar-derived carbonyl compounds, Passerini reactions were performed with furanose ketone **8** and pyranose ketone **9**. Although ketones are generally less reactive in the Passerini reaction, products **8a** and **9a** were obtained in satisfactory yields (67% and 51%, respectively), while isolation and purification of products **8d** and **9d** were inefficient, so their data are not included in this paper. As with aldehyde **7**, there was virtually no stereoselectivity (55:45 d.r.).

At this point, we were able to observe that bis-isorpopylidene-protected compounds bearing an aldehyde group attached to the tetrasubstituted carbon atoms (**1** and **5**) are efficient chiral inductors, supplying Passerini products with 90:10 d.r. The same diastereoselectivity was observed with compound **6**, in which an aldehyde group is attached to the trisubstituted carbon atom (C3 position of the furanose ring). Other compounds with an aldehyde group attached to the trisubstituted carbon atom (**2**, **3**, and **4**) were less efficient chiral inductors, giving Passerini products with up to 77:23 d.r. A possible explanation can be the increased sterically hindrance of aldehyde **6** compared with **2**, **3**, or **4**, where the aldehyde group is adjacent to the ring oxygen. Acyclic aldehyde **7** and ketones **8** and **9** gave practically racemic mixtures of Passerini products.

**Scheme 1:**
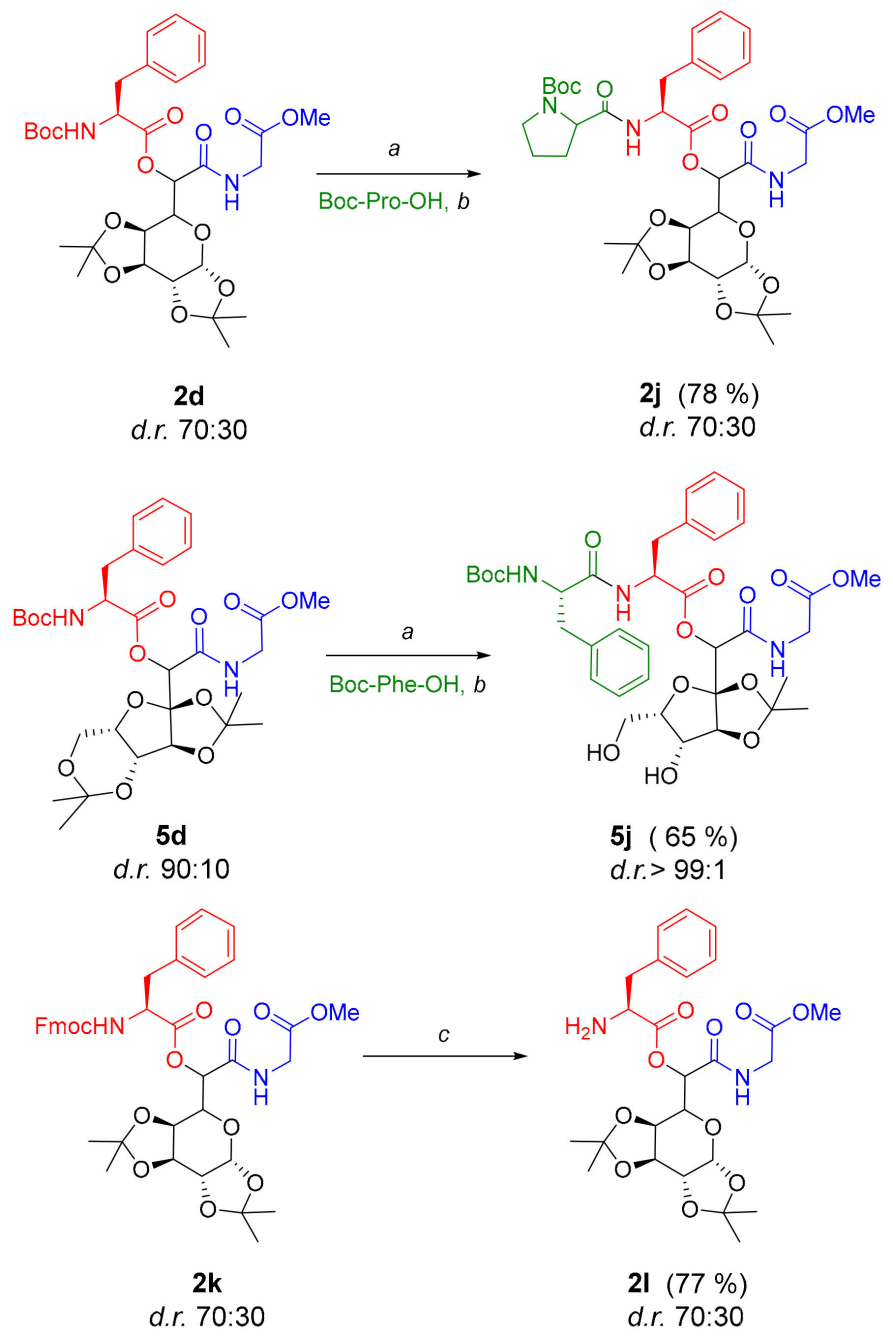
Post-condensation modification of selected Passerini products: *a* trifluoroacetic acid (TFA)/ dichloromethane (DCM) = 1:1, room temperature (RT), 2 min; *b* benzotriazol-1-yloxytris(dimethylamino)phosphonium (BOP, 1.5 eq), hydroxybenzotriazole (HOBt,1.2 eq), *N*-methylmorpholine (NMM, 2 eq), dimethylformamide (DMF)/DCM = 1:1, RT, overnight; *c* 20% piperidine in DMF, RT, 15 min.

There are many examples of synthetic glycopeptide structures exploited for the generation of new biomaterials based on weak noncovalent interactions [1]. For example, sulfated glycopeptide supramolecular nanostructures were demonstrated to amplify the signaling of bone morphogenetic protein 2 and promote the regeneration of bone [16], while glycosaminoglycan-like nanofibers were found to mimic the bioactive functions of natural hyaluronic acid molecules [17]. Due to the fact that different supramolecular architectures can be accessed by varying peptide length and composition, we considered further functionalization of Passerini products, especially those derived from N-terminally protected amino acids. Although the presence of two different acid-labile groups, Boc and isopropylidene, seems an aggravating factor, we found a smooth deprotection/coupling approach and confirmed it on two examples presented in Scheme 1. The removal of the Boc group in product **2d** and the subsequent coupling with Boc–Pro–OH afforded the tetradepsipeptide **2j** in 78% yield. Following the same methodology, in situ deprotection of **5d** and coupling with Boc–Phe–OH gave the partially deprotected product **5j** in 65% yield. It seems that the six-membered dioxolane ring is more susceptible to acid cleavage under the applied conditions compared to the five-membered ring. Additionally, we prepared Passerini product **2k** (Scheme 1) comprising Fmoc-protected phenylalanine to confirm that selective Fmoc-deprotection in the presence of other base-labile groups is feasible with 20% piperidine in dimethylformamide (DMF) to yield 77% of the free amine **2l**.

**Scheme 2:**
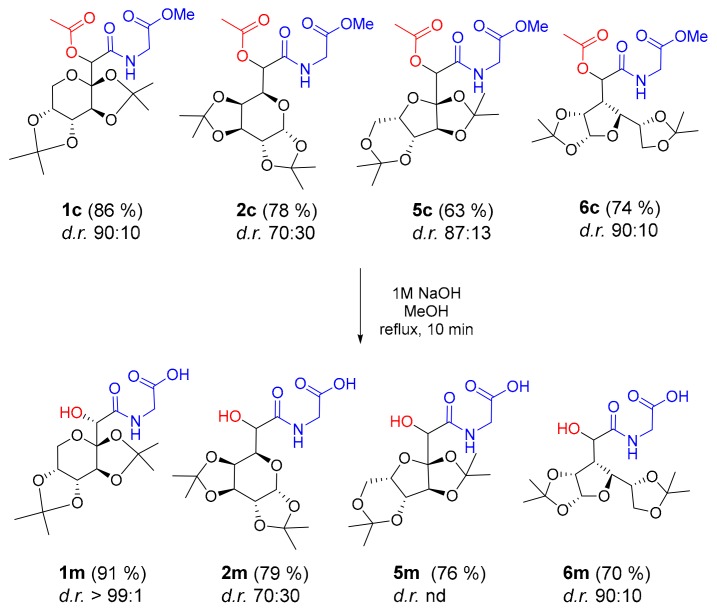
Base-mediated deprotection of selected Passerini products; nd: not determined.

The presence of a base-labile ester group in Passerini products can be of interest for another post-condensation modification, namely, the synthesis of highly valuable α-hydroxy amides or acids [18,19,20]. Therefore, we attempted the NaOH-mediated removal of acetyl groups in Passerini products **1c**, **2c**, **5c**, and **6c** (Scheme 2). All reactions worked efficiently affording the C-terminally protected dimers **1m**, **2m**, **5m**, and **6m**, comprising α-hydroxy C-glycosyl acid and glycine. Moreover, NMR analysis revealed the preservation of the chiral information derived from the diastereoselective Passerini reaction. C-glycosyl amino acids are widely exploited for the synthesis of glycomimetics, peptidomimetics, and foldamers [21]. The conformational bias imposed by the presence of a furanoid or pyranoid ring, adjustable hydrophilicity, and tunable properties in terms of substituents and stereochemistry are highly desirable features, making C-glycosyl amino acids and their derivatives versatile building blocks for multiple purposes.

With a rich portfolio of different glycosylated structures in hands and an elaborated methodology to access more complex glycopeptide systems, we are currently focused toward building up multivalent glycosylated structures, exploring and understanding the functions of the obtained molecules. We are particularly interested in understanding how the selection of carbohydrate scaffolds as well as their number and distribution within the “carrier” molecule can help in solving highly challenging problems, like targeting carbohydrate–peptide or carbohydrate–carbohydrate interactions [22].

## 3. Materials and Methods

All experiments were monitored by analytical thin-layer chromatography (TLC) on Silica Gel 60 F254 plates (Merck; Darmstadt, Germany) after spraying with 10% H_2_SO_4_ and heating. Flash column chromatography was performed on silica gel (Merck, 40–63 μm particle size) by standard techniques, eluting with solvents as indicated. All NMR experiments were carried out by using a Bruker Avance 600 spectrometer (600.13 MHz, ^1^H; 150.91 MHz, ^13^C). Samples in CDCl_3_ solutions were recorded in 5 mm NMR tubes at 298 K. Chemical shifts in parts per million were referenced to tetramethylsilane (TMS) as internal standard. Spectra were assigned on the basis of 2D homonuclear (COSY) and heteronuclear (HMQC, HMBC) experiments. ^1^H chemical shifts were assigned to the particular starting component, and the following abbreviations were used: cyclohexyl = CyHex; tetrabutyl = tBu; methyl cyanoacetate = Gly; fructopyranose-derived aldehyde **1** = Fru; galactose-derived aldehyde **2** = Gal; ribose-derived aldehyde **3** = Rib; xylose-derived aldehyde **4** = Xyl; sorbose-derived aldehyde **5** = Sor; allose-derived aldehyde **6** = Alo; arabinose-derived aldehyde **7** = Ara; glucofuranose-derived ketone **8** = GluF; fructopyranose-derived ketone **9** = FruP. High-resolution mass spectrometry (HRMS) was performed with a MALDI–TOF/TOF spectrometer in positive ionization mode. The calibration type was internal, with calibrants produced by matrix ionization dissolved in α-cyano-4-hydroxycinnamic acid matrix. Accurately measured spectra were internally calibrated, and elemental analysis was performed on Data Explorer v. 4.9 software with mass accuracy better than 5 ppm. NMR spectra of all compounds are given in Supplementary Materials.

DS1,2 refers to a product isolated as an inseparable mixture of two diastereoisomers, while DS1 and DS2 refer to isolated diastereoisomer 1 and diastereoisomer 2.

### 3.1. General Procedure for Passerini Reactions

To a glass vial containing 1 M solution of oxo-compound (0.1 mmol) in DCM under nitrogen were added acid (0.11 mmol) and the isocyanide component (0.11 mmol) dissolved in 100 µL DCM. With all reactants added, the reaction mixture was stirred at room temperature until the consumption of the aldehyde/ketone component, which typically required 6–24 h. The reactions were concentrated under reduced pressure, and the reaction mixtures were purified by flash column chromatography.

#### 3.1.1. Methyl 2-(2-acetoxy-2-((3aR,5aR,8aR,8bS)-2,2,7,7-tetramethyltetrahydro-3aH-bis([1,3]dioxolo)[4–b:4′,5′-d]pyran-3a-yl)acetamido)acetate (**1c**)

Yield 86% (36 mg); colorless oil; R_f_ (DS1,2) = 0.47 (EtOAc/petroleum ether 2:1, *v*/*v*); d.r. 90:10. ^1^H NMR (CDCl_3_) Chemical shifts are given for both isomers. δ = 6.95 (s, 0.9H, NH), 6.91 (s, 0.1H, NH), 5.50 (s, 0.1H, H-1 Fru), 5.18 (s, 0.9H, H-1 Fru), 4.69 (d, *J* = 2.6 Hz, 0.1H, H-3 Fru), 4.58 (dd, *J* = 7.9, 2.8 Hz, 0.9H, H-4 Fru), 4.54 (dd, *J* = 8.0, 2.6 Hz, 0.1H, H-4 Fru), 4.36 (d, *J* = 2.8 Hz, 0.9H, H-3 Fru), 4.22 (dd, *J* = 7.9, 1.3 Hz, 0.9H, H-5 Fru), 4.17 (dd, *J* = 8.0, 1.6 Hz, 0.1H, H-5 Fru), 4.09 (dd, *J* = 18.5, 5.0 Hz, 1H, CH_2_ Gly), 4.02 (dd, *J* = 18.5, 4.9 Hz, 1H, CH_2_ Gly) 3.96 (dd, *J* = 18.5, 4.6 Hz, 0.1H, H-6 Fru), 3.92 (dd, *J* = 13.0, 1.9 Hz, 0.9H, H-6 Fru), 3.83 (d, *J* = 13.0 Hz, 1H, H-6 Fru), 3.73 (s, 0.3H, CH_3_ Gly), 3.72 (s, 2.7H, CH_3_ Gly), 2.19 (s, 0.3H, CH_3_ Ac), 2.17 (s, 2.7H CH_3_ Ac), 1.51 (s, 3H, CH3 isop), 1.48 (s, 3H, CH_3_ isop), 1.44 (s, 3H, CH_3_ isop), 1.31 (s, 3H, CH_3_ isop). ^13^C NMR (CDCl_3_) δ = (170.2, 169.2, 165.7) (CO), (109.9, 109.6) (CH3 isop), (102.1, 101.5) (C-2 Fru), (75.7, 73.8, 71.6, 70.8, 70.8, 70.4, 70.3) (C-1,3,4,5 Fru), (62.0, 61.8) (C-6 Fru), (52.6, 52.4) (CH_3_ Gly), (41.6, 41.3) (CH_2_ Gly), (26.7, 26.7, 26.2, 26.0, 25.5, 25.2, 24.3, 24.2) (CH_3_ isop), (21.1, 20.9) (CH_3_ Ac). HRMS (MALDI–TOF/TOF) *m*/*z*: calcd. for C_18_H_27_NO_10_Na [M + Na]^+^ 440.1533; found 440.1528.

#### 3.1.2. 2-(Cyclohexylamino)-2-oxo-1-((3aR,5S,5aS,8aS,8bR)-2,2,7,7-tetramethyltetrahydro-3aH-bis([1,3]dioxolo)[4–b:4′,5′-d]pyran-5-yl)ethyl acetate (**2a**)

Yield 81% (35 mg); white solid mp = 111–112 °C; R_f_ (DS1) = 0.52, R_f_ (DS2) = 0.47 (EtOAc/petroleum ether 1:1, *v*/*v*); d.r. 80:20. ^1^H NMR (CDCl_3_) Chemical shifts are given for major isomer. δ = 6.18 (d, *J* = 7.9 Hz, 1H, NH), 5.49 (d, *J* = 4.8 Hz, 1H, H-1 Gal), 4.95 (d, *J* = 9.7 Hz, 1H, H-6 Gal), 4.60 (dd, *J* = 7.9, 2.6 Hz, 1H, H-3 Gal), 4.31 (dd, *J* = 4.8, 2.6 Hz, 1H, H-2 Gal), 4.28 (dd, *J* = 8.0, 1.8 Hz, 1H, H-4 Gal), 4.17 (dd, *J* = 9.7, 1.7 Hz, 1H, H-5 Gal), 3.84–3.70 (m, 1H, CH-1 CyHex), 2.13 (s, 3H, CH3 Ac), 1.88 (m, 2H, CyHex), 1.67 (m, 2H, CyHex), 1.56 (m, 1H, CyHex), 1.50 (s, 3H, CH_3_ isop), 1.41 (s, 3H, CH_3_ isop), 1.36–1.32 (m, 2H, CyHex), 1.31 (s, 3H, CH_3_ isop), 1.30 (s, 3H, CH_3_ isop), 1.16 (d, *J* = 11.9 Hz, 3H, CyHex). ^13^C NMR (CDCl3): δ = (169.9, 167.0) (CO), (109.8, 109.7) (C isop), 96.4 (C-1 Gal), (71.0, 70.7, 70.6, 70.6, 67.2) (C-2,3,4,5,6 Gal), 48.6 (CH CyHex), (33.04, 32.99) (CyHex), (26.29, 26.17) (CH_3_ isop), 25.78 (CyHex), 25.23 (CH_3_ isop), (24.95, 24.91) (CyHex), 24.63 (CH_3_ isop), 20.95 (CH_3_ Ac). HRMS (MALDI–TOF/TOF) *m*/*z*: calcd. for C_21_H_33_NO_8_ [M + H]^+^ 428.2284; found 428.2291.

#### 3.1.3. 2-(tert-Butylamino)-2-oxo-1-((3aR,5S,5aS,8aS,8bR)-2,2,7,7-tetramethyltetrahydro-3aH-bis([1,3]dioxolo)[4–b:4′,5′-d]pyran-5-yl)ethyl acetate (**2b**)

Yield 78% (31 mg); colorless oil; R_f_ (DS1) = 0.58, R_f_ (DS2) = 0.60 (EtOAc/petroleum ether 1:1, *v*/*v*); d.r. 80:20. ^1^H NMR (CDCl_3_) Chemical shifts are given for both isomers. δ = 6.19 (s, 0.8H, NH), 6.09 (s, 0.2H, NH), 5.49 (d, *J* = 4.8 Hz, 1H, H-1 Gal), 5.14 (d, *J* = 6.5 Hz, 0.2H, H-6 Gal), 4.87 (d, *J* = 9.8 Hz, 0.8H, H-6 Gal), 4.61–4.55 (m, 1H, H-3 Gal), 4.32–4.23 (m, 2H, H-2,4 Gal), 4.18 (d, *J* = 6.4 Hz, 0.2H, H-5 Gal), 4.11 (dd, *J* = 9.8, 1.4 Hz, 0.8H, H-5 Gal), 2.13 (s, 3H, CH_3_ Ac), 1.49 (s, 3H, CH_3_ isop), 1.40 (s, 3H, CH_3_ isop), 1.36–1.25 (m, 15H, CH_3_ isop, CH3 tBu). ^13^C NMR (CDCl_3_) δ = (170.5, 169.7, 167.2, 166.3) (CO), (109.8, 109.7, 109.7, 109.3) (C isop), (96.6, 96.4) (C-1 Gal), (73.9, 71.6, 71.1, 71.0, 70.9, 70.6, 70.6, 67.4, 67.4) (C-2,3,4,5,6 Gal), 51.7 (C tBu), (28.9, 28.7) (CH_3_ tBu), (26.3, 26.2, 26.2, 26.1, 25.2, 24.6, 24.3) (CH_3_ isop), (21.2, 20.9) (CH_3_ OAc). HRMS (MALDI–TOF/TOF) *m*/*z*: calcd. for C_19_H_31_NO_8_ [M + H]^+^ 402.2128; found 402.2146.

#### 3.1.4. Methyl 2-(2-acetoxy-2-((3aR,5S,5aS,8aS,8bR)-2,2,7,7-tetramethyltetrahydro-3aH-bis([1,3]dioxolo)[4,5-b:4′,5′-d]pyran-5-yl)acetamido)acetate (**2c**)

Yield 78% (32 mg); colorless oil; R_f_ (DS1, DS2) = 0.45 (EtOAc/petroleum ether 1:1, *v*/*v*); d.r. 70:30. ^1^H NMR (CDCl_3_) Chemical shifts are given for both isomers. δ = 6.88 (s, 0.3H, NH), 6.79 (s, 0.7H, NH), 5.50 (d, *J* = 4.7 Hz, 1H, H-1 Gal), 5.37 (d, *J* = 6.8 Hz, 0.3H, H-6 Gal), 5.08 (d, *J* = 9.5 Hz, 0.7H, H-6 Gal), 4.61 (m, 1H, H-3 Gal), 4.41 (d, *J* = 8.0 Hz, 0.3H, H-4 Gal), 4.32–4.26 (m, 2H, H-4 Gal, H-2 Gal, H-5 Gal), 4.20 (dd, *J* = 16.5, 8.4 Hz, 1H, H-5 Gal, CH_2_ Gly), 4.04 (m, 1.4H, CH_2_ Gly), 3.91 (dd, *J* = 18.3, 4.3 Hz, 0.3H, CH_2_ Gly), 3.72 (s, 3H, CH_3_ Gly), 2.15 (s, 0.9H, CH_3_ Ac), 2.12 (s, 2.1H, CH_3_ Ac), 1.53 (s, 0.9H, CH_3_ isop), 1.51 (s, 2.1H, CH_3_ isop), 1.44 (s, 0.9H, CH_3_ isop), 1.42 (s, 2.1H, CH_3_ isop), 1.31 (s, 6H, CH_3_ isop). ^13^C NMR (CDCl_3_) δ = (170.5, 170.0, 169.9, 169.8, 168.3, 167.6) (CO), (110.0, 109.8, 109.6, 109.3) (C isop), (96.5, 96.4) (C-1 Gal), (72.9, 71.3, 71.1, 70.91, 70.8, 70.7, 70.7, 70.5, 67.2, 67.0) (C-2,3,4,5,6 Gal), (52.5, 52.5) (CH_3_ Gly), (41.6, 41.4) (CH_2_ Gly)), (26.3, 26.2, 26.1, 26.1, 25.2, 25.2, 24.7, 24.4) (CH_3_ isop), (21.1, 20.8) (CH_3_ Ac). HRMS (MALDI–TOF/TOF) *m*/*z*: calcd. for C_18_H_27_NO_10_ [M + H]^+^ 418.1713; found 418.1711.

#### 3.1.5. 2-((2-Methoxy-2-oxoethyl)amino)-2-oxo-1-((3aR,5S,5aS,8aS,8bR)-2,2,7,7-tetramethyltetrahydro-3aH-bis([1,3] dioxolo)[4,5-b:4′,5′-d] pyran-5-yl)ethyl 2-((tert-butoxycarbonyl)amino)-3-phenylpropanoate (**2d**)

Yield 80% (49 mg); colorless oil; R_f_ (DS1,2) = 0.47 (EtOAc/petroleum ether 1:1, *v*/*v*); d.r. 70:30. ^1^H NMR (CDCl_3_) δ = 7.27–6.92 (m, 6H, Ph, NH), 5.50 (d, *J* = 5.0 Hz, 1.3H, H-1,6 Gal), 5.19 (d, *J* = 8.9 Hz, 0.7H, H-6 Gal), 4.97 (t, *J* = 8.0 Hz, 1H, NH), 4.67–4.45 (m, 2H, H-3 Gal, α-Phe), 4.30 (dd, *J* = 6.8, 4.4 Hz, 1.3H, H-2,4 Gal), 4.25–4.09 (m, 1.7H, H-5, H-4 Gal), 4.07–3.87 (m, 2H, CH_2_ Gly), 3.72 (d, *J* = 4.6 Hz, 3H, CH_3_ Gly), 3.22 (dd, *J* = 14.0, 6.2 Hz, 1H, β-Phe), 3.08 (dd, *J* = 14.8, 6.5 Hz, 1H, β-Phe), 1.54 (d, *J* = 3.8 Hz, 3H, CH_3_ isop), 1.46 (s, 1H, CH_3_ isop), 1.37 (bs, 11H, CH3 Boc, CH_3_ isop), 1.30 (dd, *J* = 8.0, 3.5 Hz, 6H, CH_3_ isop). ^13^C NMR (CDCl_3_) δ = (171.5, 170.8, 169.8, 169.8, 167.8, 167.3) (CO), (155.5, 155.4) (CO Boc), (136.5, 136.4) (C Ph), (129.8, 129.7, 128.7, 128.6, 127.1, 126.9) (CH Ph), (109.9, 109.8, 109.6, 109.3) (C isop), (96.4, 96.4) (C-1), (80.3, 80.1) (C Boc), (73.3, 71.6, 71.43, 71.03, 70.9, 70.6, 70.3) (C-2,3,4,6), (67.2, 67.0) (C-5 Gal), (54.9, 54.5) (α-Phe), 52.5 (CH_3_Gly), (41.5, 41.3) (CH_2_ Gly), (38.3, 37.5) (β-Phe), 28.5 (CH_3_ Boc), (26.3, 26.2, 26.2, 26.0, 25.2, 24.5, 24.5) (CH_3_ isop). HRMS (MALDI–TOF/TOF) *m*/*z*: calcd. for C_30_H_42_N_2_O_12_Na [M + Na]^+^ 645.2635; found 645.2615.

#### 3.1.6. 2-((2-Methoxy-2-oxoethyl)amino)-2-oxo-1-((3aR,5S,5aS,8aS,8bR)-2,2,7,7-tetramethyltetrahydro-3aH-bis([1,3] dioxolo)[4,5-b:4′,5′-d] pyran-5-yl)ethyl 2-((tert-butoxycarbonyl)amino)-4-methylpentanoate (**2e**)

Yield 58% (34 mg); colorless oil; R_f_ (DS1,2) = 0.55, (EtOAc/petroleum ether 1:1, *v*/*v*); *¡* 75:25. ^1^H NMR (CDCl_3_) Chemical shifts are given for both isomers. δ = 7.33 (s, 0.25H, NH), 6.93 (s, 0.75H, NH), 5.50 (0.25, H-6 Gal), 5.50 (d, *J* = 4.8 Hz, 0.75H, H-1 Gal), 5.44 (d, *J* = 4.8 Hz, 0.25H, H-1 Gal), 5.19 (d, *J* = 8.9 Hz, 0.75H, H-6 Gal), 4.90 (dd, *J* = 16.4, 7.7 Hz, 1H, NH Leu), 4.63–4.60 (m, 0.75H, H-3 Gal), 4.57 (dd, *J* = 7.9, 2.1 Hz, 0.25H, H-3 Gal), 4.50 (d, *J* = 7.6 Hz, 0.25H, α-Leu), 4.36 (m, 0.75H, α-Leu), 4.33–4.16 (m, 3H, H-2,4,5 Gal), 4.02 (ddd, *J* = 41.5, 18.2, 5.3 Hz, 2H, CH_2_ Gly), 3.70 (d, *J* = 6.9 Hz, 3H, CH_3_ Gly), 1.74 (s, 3H, β-Leu, γ-Leu), 1.51 (d, *J* = 7.5 Hz, 3H, CH_3_ isop), 1.45–1.37 (m, 15H, CH_3_ Boc, CH3 isop), 1.31–1.27 (m, 3H, CH_3_ isop), 0.94–0.91 (m, 6H, CH3 Leu). ^13^C NMR (CDCl_3_) δ = (172.8, 172.0, 169.9, 169.8, 167.8, 167.5) (CO), (156.0, 155.7) (CO Boc), (109.9, 109.6, 109.1) (C isop), 96.4 (C-1 Gal), (80.3, 80.0) (C Boc), (72.7, 71.6, 71.0, 71.0, 70.6, 70.4) (C-2,3,4,6 Gal), (67.4, 67.0) (C-5 Gal), (52.9, 52.4, 52.3) (CH_3_ Gly, α-Leu), (41.8, 41.4, 41.3, 41.0) (CH_2_ Gly, β-Leu), 29.9, (28.5, 28.5) (Boc), (26.3, 26.2, 26.1) (CH_3_ isop), (25.3, 25.2) (γ-Leu), (24.9, 24.8, 24.6, 23.1, 23.0) (CH_3_ isop), (22.3, 22.0) (δ-Leu). HRMS (MALDI–TOF/TOF) *m*/*z*: calcd. for C_27_H_44_N_2_O_12_Na [M + Na]^+^ 611.2792; found 611.2805.

#### 3.1.7. 1-tert-Butyl 2-(2-((2-methoxy-2-oxoethyl) amino)-2-oxo-1-((3aR,5S,5aS,8aS,8bR)-2,2,7,7-tetramethyltetrahydro-3aH-bis([1,3] dioxolo)[4,5-b:4′,5′-d] pyran-5-yl)ethyl)pyrrolidine-1,2-dicarboxylate (**2f**)

Yield 67% (40 mg); colorless oil; R_f_ (DS1,2) = 0.35, (EtOAc/petroleum ether 1:1, *v*/*v*); d.r. 55:45. ^1^H NMR (CDCl_3_) δ = 7.38 (dd, J = 19.5, 14.3 Hz, 0.45H, NH), 6.74 (t, *J* = 4.7 Hz, 0.55H, NH), 5.49 (d, *J* = 4.8 Hz, 1H, H-1 Gal), 5.28 (d, *J* = 8.0 Hz, 0.55H, H-6 Gal), 5.05 (d, *J* = 9.7 Hz, 0.45H, H-6 Gal), 4.60 (ddd, *J* = 25.8, 7.9, 2.3 Hz, 1H, H-3 Gal), 4.38–4.23 (m, 4H, H-2, H-4, H-5 Gal, CH_2_ Gly), 4.10 (dd, *J* = 17.9, 6.0 Hz, 0.55H, CH_2_ Gly), 4.02 (t, *J* = 5.7 Hz, 1H, α-Pro), 3.88 (dd, *J* = 17.9, 5.3 Hz, 0.45H, CH_2_ Gly), 3.71 (s, 1.6H, CH_3_ Gly), 3.68 (s, 1.4H, CH_3_ Gly), 3.55–3.46 (m, 1H, δ-Pro), 3.42–3.30 (m, 1H, δ-Pro), 2.33–2.26 (m, 0.55H, β-Pro), 2.22–2.13 (m, 1.5H, β-Pro), 1.88 (ddd, *J* = 25.6, 11.5, 8.4 Hz, 2H, γ-Pro), 1.36 (ddd, *J* = 36.5, 29.4, 26.3 Hz, 21H, CH_3_ Boc, CH_3_ isop). ^13^C NMR (CDCl_3_) δ = (171.8, 171.5, 169.9, 168.3, 167.8) (CO), (155.3, 154.1) (CO Boc), (109.8, 109.7, 109.6, 109.3) (C isop), (96.5, 96.4) (C-1 Gal), (80.2, 80.1) (C Boc), (71.9, 71.2, 70.9, 70.8, 70.7, 70.5, 70.4) (C-2,3,4,6 Gal), (67.1, 66.9) (C-5 Gal), (59.2, 58.9) (α-Pro), (52.4, 52.3) (CH3 Gly), (47.0, 46.5) (δ-Pro), (41.5, 41.2) (CH_2_ Gly), (30.9, 30.2) (β-Pro), (28.7, 28.5) (CH_3_ Boc), (26.3, 26.2, 26.2, 25.2, 25.2, 24.7, 24.4) (CH_3_ isop), (24.4, 23.4) (γ-Pro). HRMS (MALDI–TOF/TOF) *m*/*z*: calcd. for C_26_H_40_N_2_O_12_ [M + H]^+^ 595.2479; found 595.2453.

#### 3.1.8. 1-Benzyl 2-tert-butyl 1-(1-(2-((2-methoxy-2-oxoethyl) amino)-2-oxo-1-((3aR,5S,5aS,8aS,8bR)-2,2,7,7-tetramethyltetrahydro-3aH-bis([1,3]dioxolo)[4,5-b:4′,5′-d] pyran-5-yl)ethoxy)-1-oxo-3-phenylpropan-2-yl) hydrazine-1,2-dicarboxylate (**2g**)

Yield 74% (57 mg); colorless oil; R_f_ (DS1,2) = 0.44 (EtOAc/petroleum ether 1:1, *v*/*v*); d.r. 70:30. ^1^H NMR (CDCl_3_) δ = 7.27 (d, *J* = 16.6 Hz, 11H, CH Ph, NH), 5.50 (d, *J* = 4.8 Hz, 1H, H-1 Gal), 5.25–5.16 (m, 4H, H-6 Gal, CH_2_ Cbz, NH), 5.07 (m, 1H, α-hPhe), 4.55 (d, *J* = 10.2 Hz, 1H, H-3 Gal), 4.46 (dd, *J* = 8.0, 1.3 Hz, 1H, H-4 Gal), 4.30 (dd, *J* = 4.7, 2.5 Hz, 1H, H-2 Gal), 4.15–3.98 (m, 3H, H-5 Gal, CH_2_ Gly), 3.72 (s, 1.8H, CH_3_ Gly), 3.70 (s, 1.2H, CH_3_ Gly), 3.31 (dd, *J* = 12.8, 6.8 Hz, 1H, β-hPhe), 3.24–3.07 (m, 1H, β-hPhe), 1.54–1.12 (m, 21H, CH_3_ Boc, CH_3_ isop). ^13^C NMR (CDCl_3_) δ (169.8, 169.1, 168.5, 167.6) (CO), 155.3 (CO Boc), (137.9, 136.0) (C Ph), (129.6, 128.5, 128.4, 128.2, 127.9, 126.6) (CH Ph), (110.1, 109.8, 109.6, 109.6) (C isop), (96.4, 96.3) (C-1 Gal), (81.2, 81.2) (C Boc), (70.9, 70.8, 70.8, 70.4, 70.2) (H-2,3,4,6 Gal), (68.5, 68.3) (CH_2_ Cbz), (67.7, 67.1) (C-5 Gal), (62.7, 62.2) (α-Phe), 52.5 (CH_3_ Gly), (41.5, 41.2) (CH_2_ Gly), (34.6, 34.3) (β-Phe), (28.3, 28.1) (CH_3_ Boc), (26.2, 26.1, 26.0, 25.9, 25.1, 24.4, 24.4, 24.2) (CH_3_ isop). HRMS (MALDI–TOF/TOF) *m*/*z*: calcd. for C_38_H_49_N_3_O_12_Na [M + Na]^+^ 794.3112; found 794.3111.

#### 3.1.9. 1-Benzyl 2-tert-butyl 1-(1-(2-((2-methoxy-2-oxoethyl)amino)-2-oxo-1-((3aR,5S,5aS,8aS,8bR)-2,2,7,7-tetramethyltetrahydro-3aH-bis([1,3]dioxolo)[4,5-b:4′,5′-d]pyran-5-yl)ethoxy)-4-methyl-1-oxopentan-2-yl)hydrazine-1,2-dicarboxylate (**2h**)

Yield 53% (39 mg); colorless oil; R_f_ (DS1,2) = 0.54 (EtOAc/petroleum ether 1:1, *v*/*v*); d.r. 70:30. ^1^H NMR (CDCl_3_) δ = 7.31 (m, 5H, Ph Cbz), 6.91 (s, H, NH), 6.72 (s, H, NH), 5.49 (dd, *J* = 13.6, 4.7 Hz, 1H, H-1 Gal), 5.15 (ddd, *J* = 33.8, 24.8, 12.0 Hz, 4H, H-6 Gal, CH_2_ Cbz, NH), 4.79 (t, *J* = 10.2 Hz, 1H, α-hLeu), 4.63–4.48 (m, 1H, H-3 Gal), 4.39–3.86 (m, 5H, H-2,4,5 Gal, CH_2_ Gly), 3.72 (d, *J* = 3.1 Hz, 3H, CH_3_ Gly), 1.94 (m, 1H, β-hLeu), 1.63 (m, 1H, β-hLeu), 1.56–1.19 (m, 21H, CH_3_ Boc, CH_3_ isop), 1.06–0.80 (m, 6H, δ-hLeu). ^13^C NMR (CDCl_3_) δ = (169.8, 169.7, 167.7) (CO), (156.4, 155.1) (CO Boc), 136.1 (C Cbz), (128.8, 128.8, 128.3) (CH Cbz), (109.9, 109.86, 109.8, 109.8) (C isop), (96.4, 96.3) (C-1 Gal), (81.5, 81.4) (CO Boc), (74.4, 71.6, 71.4, 70.9, 70.6, 70.4, 70.2) (C-2,3,4,6), (68.8, 68.7) (CH_2_ Cbz), (67.2, 67.1, 66.9) (C-5 Gal), (60.3, 59.3) (α-Leu), 52.5 (CH_3_ Gly), (41.6, 39.9) (CH_2_ Gly), (37.0, 36.8) (β-Leu), 28.4 (CH_3_ Boc), (26.2, 26.2, 26.1, 26.0) (CH_3_ isop), (25.2, 25.2) (γ-Leu), (24.5, 24.4, 24.3, 23.9, 23.5) (CH_3_ isop), (21.7, 21.7) (δ-Leu).HRMS (MALDI–TOF/TOF) *m*/*z*: calcd. for C_35_H_51_N_3_O_14_ [M + Na]^+^ 760.3269; found 760.3243.

#### 3.1.10. 2-((2-Methoxy-2-oxoethyl)amino)-2-oxo-1-((3aR,5S,5aS,8aS,8bR)-2,2,7,7-tetramethyltetrahydro-3aH-bis([1,3]dioxolo)[4,5-b:4′,5′-d]pyran-5-yl)ethyl 1-((tert-butoxycarbonyl)amino)pyrrolidine-2-carboxylate (**2i**)

Yield 66% (39 mg); colorless oil; R_f_ (DS1,2) = 0.38 (EtOAc/petroleum ether 2:1, *v*/*v*); d.r. 70:30. ^1^H NMR (CDCl_3_) δ = 7.03 (s, 0.3H), 6.95 (s, 0.7H), 6.71 (d, *J* = 13.8 Hz, 1H), 5.52 (d, *J* = 4.8 Hz, 0.7H), 5.49 (d, *J* = 4.9 Hz, 0.3H), 5.37 (d, *J* = 8.0 Hz, 0.3H), 5.19 (d, *J* = 9.3 Hz, 0.7H), 4.61 (dd, *J* = 7.9, 2.4 Hz, 1H), 4.47 (dd, *J* = 8.0, 1.7 Hz, 0.3H), 4.32 (dd, *J* = 4.9, 2.6 Hz, 0.7H), 4.28 (dd, *J* = 7.8, 1.8 Hz, 1H), 4.21 (dd, *J* = 9.3, 1.8 Hz, 0.3H), 4.18–4.13 (m, 0.7H), 4.03 (dd, *J* = 7.4, 5.4 Hz, 2H), 3.92 (dd, *J* = 18.3, 4.8 Hz, 0.3H), 3.71 (s, 0.9H), 3.71 (s, 2.1H), 3.29 (td, *J* = 14.2, 7.6 Hz, 1H, δ-Pro), 3.13–3.06 (m, 1H, δ-Pro), 2.21 (dq, *J* = 16.4, 8.2 Hz, 1H, β-Pro), 2.13–2.03 (m, 1H, β-Pro), 1.93–1.84 (m, 2H, γ-Pro), 1.38 (m, 21H, CH_3_ Boc, CH_3_ isop). ^13^C NMR (CDCl_3_) δ = (173.0, 172.0, 170.0, 169.9, 168.3, 167.1) (CO), (110.1, 110.0, 109.7, 109.3) (C isop), (96.5, 96.4) (C-1 Gal), (80.2, 80.0) (CO Boc), (72.8, 71.1, 70.9, 70.8, 70.7, 70.6, 70.4) (C-2,3,4,6 Gal), (67.5, 67.2) (C-5 Gal), (64.8, 64.0) (α-Pro), (53.4, 53.2) (δ-Pro), (52.5, 52.4) (CH_3_ Gly), (41.5, 41.3) (CH_2_ Gly), (28.6, 28.5) (CH_3_ Boc), (27.9, 27.6) (β-Pro), (26.2, 26.2, 26.1, 25.2, 25.1, 24.6, 24.5) (CH_3_ isop), (22.3, 22.2) (γ-Pro). HRMS (MALDI–TOF/TOF) *m*/*z*: calcd. for C_26_H_41_N_3_O_12_Na [M + Na]^+^ 610.2588; found 610.2571.

#### 3.1.11. 2-((2-Methoxy-2-oxoethyl)amino)-2-oxo-1-((3aR,5S,5aS,8aS,8bR)-2,2,7,7-tetramethyltetrahydro-3aH-bis([1,3]dioxolo)[4,5-b:4′,5′-d]pyran-5-yl)ethyl 2-((((9H-fluoren-9-yl)methoxy)carbonyl)amino)-3-phenylpropanoate (**2k**)

Yield 74% (55 mg); colorless oil; R_f_ (DS1,2) = 0.39, (EtOAc/petroleum ether 1:1, *v*/*v*); d.r. 70:30. ^1^H NMR (CDCl_3_) δ = 7.73 (d, *J* = 7.5 Hz, 2H, Fmoc), 7.54–7.49 (m, 2H, Fmoc), 7.37 (t, *J* = 7.4 Hz, 2H, Fmoc), 7.30–7.19 (m, 7H, Ph), 6.96 (s, 0.3H, NH), 6.87 (s, 0.7H, NH), 5.52 (d, *J* = 4.7 Hz, 0.3H, H-1 Gal), 5.50 (d, *J* = 4.8 Hz, 0.7H, H-1 Gal), 5.44 (d, *J* = 7.6 Hz, 0.3H, H-6 Gal), 5.31 (d, *J* = 8.3 Hz, 0.7H, NH), 5.29–5.24 (m, 0.3H, NH), 5.17 (d, *J* = 9.2 Hz, 0.7H, H-6 Gal), 4.78–4.69 (m, 1H, α-Phe), 4.58 (ddd, *J* = 32.1, 7.8, 2.2 Hz, 1H, H-3 Gal), 4.38 (dt, *J* = 18.2, 9.1 Hz, 1H, CH_2_ Fmoc), 4.30 (dd, *J* = 4.9, 2.6 Hz, 1H, H-2 Gal), 4.27–4.00 (m, 6H, CH_2_ Gly, CH Fmoc, CH_2_ Fmoc, H-5,4 Gal), 3.72 (s, 2.1H, CH_3_ Gly), 3.68 (s, 0.9H, CH_3_ Gly), 3.26 (dd, *J* = 14.0, 6.2 Hz, 1H, β-Phe), 3.13 (dd, *J* = 14.0, 6.4 Hz, 1H, β-Phe), 1.55–1.21 (m, 12H, CH_3_ isop). ^13^C NMR (CDCl_3_) δ (171.1, 170.5, 169.8, 167.7) (CO Fmoc), (155.9, 155.8) (CO Fmoc), (144.0, 143.9, 141.5) (C Fmoc), (136.4, 136.1) (C Ph), (129.8, 129.8, 128.8, 128.6, 127.9, 127.2, 127.1) (CH Ph), (125.3, 125.3, 125.2) (CH Fmoc-Ph), (120.2, 120.1) (CH Fmoc-Ph), (110.0, 109.9, 109.6, 109.3) (C isop), (96.4, 96.4) (C-1 Gal), (73.5, 71.5, 71.3, 71.0, 70.9, 70.8, 70.5, 70.3) (C-2,3,4,6 Gal), 67.4 (C-5 Gal), 67.2 (CH_2_ Fmoc), 67.0 (C-5 Gal), (55.1, 54.9) (α-Phe), (52.5, 52.4) (CH_3_ Gly), (47.4, 47.3) (CH Fmoc), (41.5, 41.3) (CH_2_ Gly), (38.3, 37.6) (β-Phe), (26.3, 26.2, 26.2, 26.0, 25.2, 24.5, 24.4) (CH_3_ isop). HRMS (MALDI–TOF/TOF) m/z: calcd. for C_40_H_44_N_2_O_12_ [M + H]^+^ 745.2972; found 745.2990.

#### 3.1.12. 2-(Cyclohexylamino)-1-((3aR,4S,6R,6aR)-6-methoxy-2,2-dimethyltetrahydrofuro[3,4-d][1,3] dioxol-4-yl)-2-oxoethyl acetate (**3a**)

Yield 82% (30 mg); colorless oil; R_f_ = 0.63 (EtOAc/petroleum ether 1:1, *v*/*v*); d.r. 77:23. ^1^H NMR (CDCl_3_) Chemical shifts are given for both isomers. δ = 5.97 (d, *J* = 7.5 Hz, 0.77H, NH), 5.90 (d, *J* = 7.7 Hz, 0.23H, NH), 5.11 (d, *J* = 7.6 Hz, 0.23H, H-5 Rib), 5.07 (d, *J* = 6.9 Hz, 0.77H, H-5 Rib), 4.95 (s, 0.23H, H-1 Rib), 4.94 (s, 0.77H, H-1 Rib), 4.87 (dd, *J* = 6.0, 1.4 Hz, 0.23H, H-2 Rib), 4.71 (dd, *J* = 6.2, 0.5 Hz, 0.77H, H-2 Rib), 4.60 (d, *J* = 6.9 Hz, 0.77H, H-4 Rib), 4.55 (d, *J* = 6.0 Hz, 0.77H, H-3 Rib), 4.53 (d, *J* = 6.0 Hz, 0.23H, H-3 Rib), 4.50 (dd, *J* = 7.6, 1.4 Hz, 0.23H, H-4 Rib), 3.79–3.72 (m, 1H, CH-1 CyHex), 3.32 (s, 2.3H, OCH_3_), 3.28 (s, 0.7H, OCH_3_), 2.15 (s, 0.7H, CH_3_ Ac), 2.13 (s, 2.3H, CH_3_ Ac), 1.89 (m, 2H, CyHex), 1.66 (m, 2H, CyHex), 1.58 (m, 1H, CyHex), 1.45 (s, 3H, CH_3_ isop), 1.34 (m, 2H, CyHex), 1.29 (s, 2.3H, CH_3_ isop), 1.28 (s, 0.7H, CH_3_ isop), 1.16–1.14 (m, 3H, CyHex). ^13^C NMR (CDCl_3_) δ = (170.0, 166.1) (CO), 112.9 (C isop), (111.1, 110.3) (C-1 Rib), (86.2, 86.0) (C-4 Rib), (85.4, 85.3) (C-3 Rib), (81.7, 81.4) (C-2 Rib), (74.8, 73.6) (C-5 Rib), (56.2, 55.5) (OCH3), 48.5 (CH CyHex), 48.35 (CH CyHex), (33.1, 33.1) (CyHex), (26.8, 26.7) (CH_3_ isop), (25.7, 25.6, 25.3, 24.9, 24.9) (CyHex), (21.4, 21.1) (CH_3_ Ac). HRMS (MALDI–TOF/TOF) *m*/*z*: calcd. for C_18_H_29_NO_7_Na [M + Na]^+^ 394.1842; found 394.1843.

#### 3.1.13. 1-((3aR,4S,6R,6aR)-6-Methoxy-2,2-dimethyltetrahydrofuro[3,4-d][1,3]dioxol-4-yl)-2-((2-methoxy-2-oxoethyl)amino)-2-oxoethyl 2-((tert-butoxycarbonyl)amino)-3-phenylpropanoate (**3d**)

Yield 78% (44 mg); colorless oil; R_f_ (DS1) = 0.53, (EtOAc/petroleum ether 1:1, *v*/*v*); d.r. 77:23. ^1^H NMR (CDCl_3_) δ = 7.36–7.08 (m, 5H, CH Ph), 7.15 (s, 1H, NH), 5.36 (d, *J* = 4.6 Hz, 1H, NH), 5.19 (d, *J* = 4.8 Hz, 0.23H, H-5 Rib), 5.04 (d, *J* = 7.6 Hz, 0.77H, H-5 Rib), 4.96 (s, 0.77H, H-1 Rib), 4.94 (s, 0.23H, H-1 Rib), 4.74–4.53 (m, 4H, H-2,3,4 Rib, α-Phe), 4.09 (d, *J* = 5.8 Hz, 0.23H, CH_2_ Gly), 4.03 (d, *J* = 5.8 Hz, 0.77H, CH_2_ Gly), 3.94 (d, *J* = 5.4 Hz, 0.77H, CH2 Gly), 3.88 (d, *J* = 5.4 Hz, 0.23H, CH_2_ Gly), 3.71 (d, *J* = 5.2 Hz, 3H, CH_3_ Gly), 3.33–3.30 (m, 3.77H, CH3 Rib, β-Phe), 2.98 (dd, *J* = 14.3, 8.8 Hz, 1.23H, β-Phe), 1.46 (s, 2.31H, CH_3_ isop), 1.42 (s, 0.69H, CH_3_ isop), 1.39 (s, 2.31H, Boc), 1.37 (s, 6.9H, Boc), 1.31 (s, 2.1H, CH_3_ isop), 1.27 (s, 0.69H, CH_3_ isop). ^13^C NMR (CDCl_3_) δ = (170.5, 169.6, 167.2) (CO), 156.2 (CO Boc), 136.4 (C Ph), (129.6, 129.3, 129.0, 128.9, 127.6, 127.3) (CH Ph), 112.8 (C isop), (111.9, 109.9) (C-1 Rib), (86.4, 86.2) (C-4 Rib), (85.7, 85.6) (C-3 Rib), (81.2, 81.0) (C-2 Rib), (80.9, 80.7) (C Boc), (75.0, 74.5) (C-5 Rib), (56.3, 55.9) (α-Phe), (55.3, 54.9) (CH_3_ Rib), (52.5, 52.3) (CH_3_ Gly), (41.2, 41.1) (CH_2_ Gly), (37.6, 37.6) (β-Phe), (28.5, 28.4) (CH_3_ Boc), (26.9, 26.7) (CH_3_ isop), (25.7, 25.3) CH3 isop). HRMS (MALDI–TOF/TOF) *m*/*z*: calcd. for C_27_H_38_N_2_O_11_Na [M + Na]^+^ 589.2373; found 589.2399.

#### 3.1.14. 1-((3aR,5S,6S,6aR)-6-(Benzyloxy)-2,2-dimethyltetrahydrofuro[2,3-d][1,3]dioxol-5-yl)-2-(cyclohexylamino)-2-oxoethyl acetate (**4a**)

Yield 79% (36 mg); colorless oil; R_f_ (DS1) = 0.40, R_f_ (DS2) = 0.26 (EtOAc/petroleum ether 1:2, *v*/*v*); d.r. 77:23. ^1^H NMR (CDCl_3_) Chemical shifts are given for major isomer. δ = 7.41–7.14 (m, 5H, Ph), 6.01 (d, *J* = 7.9 Hz, 1H, NH), 5.92 (d, *J* = 3.7 Hz, 1H, H-1 Xyl), 5.11 (d, *J* = 9.2 Hz, 1H, H-5 Xyl), 4.62 (d, *J* = 11.6 Hz, 1H, CH_2_a-Ph), 4.60 (d, *J* = 3.7 Hz, 1H, H-2 Xyl), 4.45–4.38 (m, 2H, H-4 Xyl, CH2b-Ph), 3.99 (d, *J* = 3.1 Hz, 1H, H-3 Xyl), 3.79–3.70 (m, 1H, CH-1 CyHex), 1.98 (s, 3H, CH_3_ Ac), 1.84 (m, 2H, CyHex), 1.63 (m, 2H), 1.55–1.50 (m, 1H, CyHex), 1.45 (s, 3H, CH_3_ isop), 1.30 (bs, 5H, CH_3_ isop, CyHex), 1.16–1.06 (m, 3H, CyHex). ^13^C NMR (CDCl_3_) δ (169.70, 166.78) (CO), 136.97 (C Ph), (128.84, 128.45, 128.35) (CH Ph), 112.61 (C isop), 105.65 (C-1 Xyl), (81.79, 81.30, 78.64, 72.47, 70.22) (C-2,3,4,5-Xyl, CH_2_-Ph), 48.52 (CH CyHex), (32.89, 32.83) (CyHex), (27.01, 26.58) (CH_3_ isop), (25.73, 24.79, 24.75) (CyHex), 20.78 (CH_3_ Ac). HRMS (MALDI–TOF/TOF) *m*/*z*: calcd. for C_24_H_33_NO_7_ [M + H]^+^ 448.2335; found 448.2346.

#### 3.1.15. 1-((3aR,5S,6S,6aR)-6-(Benzyloxy)-2,2-dimethyltetrahydrofuro[2,3-d][1,3]dioxol-5-yl)-2-((2-methoxy-2-oxoethyl) amino)-2-oxoethyl 2-((tert-butoxycarbonyl) amino)-3-phenylpropanoate (**4d**)

Yield 75% (48 mg); colorless oil; R_f_ (DS1) = 0.52, (EtOAc/petroleum ether 1:1, *v*/*v*); d.r. 77:23. ^1^H NMR (CDCl_3_) δ = 7.42–7.11 (m, 10H, Bn, Ph), 6.86 (s, 1H, NH), 5.94 (d, *J* = 3.6 Hz, 1H, H-1 Xyl), 5.65 (d, *J* = 7.2 Hz, 0.23H, H-5 Xyl), 5.43 (d, *J* = 9.0 Hz, 0.77H, H-5 Xyl), 4.91 (m, 1H, α-Phe), 4.67–4.39 (m, 5.77H, H-2, H-4, H-3 Xyl, NH, CH_2_ OBn), 4.24 (d, *J* = 3.9 Hz, 0.23H, H-3 Xyl), 4.02 (d, *J* = 5.3 Hz, 0.77H, CH_2_ Gly), 3.99 (m, 0.77H, CH_2_ Gly), 3.92 (d, *J* = 5.7 Hz, 0.46H, CH_2_ Gly), 3.71 (s, 2.31H, CH_3_ Gly), 3.69 (s, 0.69H, CH_3_ Gly), 3.28 (dd, *J* = 14.1, 5.6 Hz, 0.77H, β-Phe), 3.12 (dd, *J* = 14.2, 5.3 Hz, 0.23H, β-Phe), 3.00 (dd, *J* = 14.1, 7.7 Hz, 0.77H, β-Phe), 2.88 (dd, *J* = 14.1, 8.3 Hz, 0.23H, β-Phe), 1.48 (s, 3H, CH_3_ isop), 1.35 (s, 3H, CH_3_ isop), 1.30 (s, 9H, CH_3_ Boc). ^13^C NMR (CDCl_3_) δ (171.0, 169.8, 167.7) (CO), 155.4 (CO Boc), (137.1, 136.5, 136.1) (C Ph), (129.7, 129.5, 128.8, 128.7, 128.6, 128.4, 128.3, 128.2, 127.2, 127.0) (CH Ph), (112.8, 112.4) (C isop), (105.8, 105.2) (C-1 Xyl), (83.0, 83.0) (C-2 Xyl), (81.9, 81.4) (C-3 Xyl), (80.6, 80.0) (C Boc), (79.0, 78.5) (C-4 Xyl), (72.8, 72.6) (CH_2_-OBn), (72.1, 70.0) (C-5 Xyl), (55.0, 54.3) (α-Phe), (52.5, 52.4) (CH_3_ Gly), (41.5, 41.1) (CH_2_ Gly), (37.9, 37.1) (β-Phe), 28.3 (CH_3_ Boc), (28.35, 27.2, 27.0, 26.6, 26.5) (CH_3_ isop). HRMS (MALDI–TOF/TOF) *m*/*z*: calcd. for C_33_H_42_N_2_O_11_Na [M + Na]^+^ 665.2686; found 665.2682.

#### 3.1.16. 2-(Cyclohexylamino)-2-oxo-1-((3aS,3bR,7aS,8aR)-2,2,5,5-tetramethyltetrahydro-3aH-[1,3]dioxolo[4′,5′:4,5]furo[3,2-d][1,3]dioxin-8a-yl)ethyl acetate (**5a**)

Yield 66% (28 mg); colorless oil; R_f_ (DS1) = 0.50, Rf (DS2) = 0.40 (toluen/aceton 5:1, *v*/*v*); d.r. 90:10. ^1^H NMR (CDCl_3_) Chemical shifts are given for major isomer. δ = 6.30 (d, *J* = 7.6 Hz, 1H, NH), 5.26 (s, 1H, H-1 Sor), 4.41 (s, 1H, H-5 Sor), 4.31 (d, *J* = 2.2 Hz, 1H, H-4 Sor), 4.17 (d, *J* = 1.5 Hz, 1H, H-3 Sor), 4.07 (dd, *J* = 13.6, 2.2 Hz, 1H, H-6a Sor), 3.98 (d, *J* = 13.5 Hz, 1H, H-6b Sor), 3.85–3.75 (m, 1H, CH-1 CyHex), 2.17 (s, 3H, CH_3_ Ac), 1.89 (m, 2H, CyHex), 1.63 (s, 3H, CyHex), 1.54 (m, 1H, CyHex), 1.46 (s, 3H, CH_3_ isop), 1.41 (d, 6H, CH_3_ isop), 1.38 (bs, 3H, CH_3_ isop), 1.34 (m, 1H, CyHex), 1.24–1.17 (m, 3H, CyHex). ^13^C NMR (CDCl_3_): δ = (169.6, 164.6) (CO), (113.8, 113.2) (C isop), 97.8 (C-2 Sor), 85.66 (C-5 Sor), 73.7 (C-3 Sor), 72.9 (C-4 Sor), 72.7 (C-1 Sor), 60.6 (C-6 Sor), 48.4 (CH CyHex), 32.9 (CyHex), (32.8, 29.2) (CyHex), (27.8, 26.5, 25.9) (CH_3_ isop), (24.7, 24.7) (CyHex), 21.0 (CH_3_ isop), 18.9 (CH_3_ Ac). HRMS (MALDI–TOF/TOF) *m*/*z*: calcd. for C_21_H_33_NO_8_ [M + H]^+^ 428.2284; found 428.2272.

#### 3.1.17. Methyl 2-(2-acetoxy-2-((3aS,3bR,7aS,8aR)-2,2,5,5-tetramethyltetrahydro-3aH-[1,3]dioxolo[4′,5′:4,5]furo [3,2-d][1,3] dioxin-8a-yl)acetamido)acetate (**5c**)

Yield 63% (26 mg); colorless oil; R_f_ (DS1) = 0.30, R_f_ (DS2) = 0.27 (EtOAc/petroleum ether 1:1, *v*/*v*); d.r. 87:13. ^1^H NMR (CDCl_3_) δ = 7.10 (s, 0.87H, NH), 6.84 (s, 0.13H, NH), 5.68 (s, 0.13H, H-1 Sor), 5.39 (s, 0.87H, H-1 Sor), 4.87 (s, 0.13H, H-5 Sor), 4.41 (s, 0.87H, H-5 Sor), 4.33 (d, *J* = 1.7 Hz, 0.87H, H-4 Sor), 4.29 (d, *J* = 2.3 Hz, 0.13H, H-4 Sor), 4.23–3.96 (m, 5H, H-3,6 Sor, CH_2_ Gly), 3.73 (d, *J* = 1.8 Hz, 3H, CH_3_ Gly), 2.22 (s, 0.39H, CH_3_ Ac), 2.18 (s, 2.61H, CH_3_ Ac), 1.47–1.30 (m, 12H, CH_3_ isop). ^13^C NMR (CDCl_3_) δ (170.2, 169.4, 165.6) (CO), 114.0 (C isop), 112.9 (C isop), 97.8 (C-2 Sor), 85.6 (C-5 Sor), (74.5, 74.0, 73.5, 73.2, 72.8, 72.4) (C-1,3,4 Sor), 60.4 (C-6 Sor), 52.5 (CH_3_ Gly), (41.7, 41.3) (CH_2_ Gly), (29.1, 28.5, 27.9, 27.7, 26.4) (CH_3_ isop), (19.2, 18.8) (CH_3_ Ac). HRMS (MALDI–TOF/TOF) *m*/*z*: calcd. for C_18_H_27_NO_10_ [M + H]^+^ 418.1713; found 418.1697.

#### 3.1.18. 2-((2-Methoxy-2-oxoethyl) amino)-2-oxo-1-((3aS,3bR,7aS,8aR)-2,2,5,5-tetramethyltetrahydro-3aH-[1,3]dioxolo[4′,5′:4,5]furo[3,2-d][1,3]dioxin-8a-yl)ethyl 2-((tert-butoxycarbonyl) amino)-3-phenylpropanoate (**5d**)

Yield 65% (40 mg); colorless oil; R_f_ (DS1,2) = 0.34 (EtOAc/petroleum ether 1:1, *v*/*v*); d.r. 90:10. ^1^H NMR (CDCl_3_) δ = 7.35–7.05 (m, 6H, CH Ph, NH), 5.50 (s, 1H, H-1 Sor), 4.94 (d, *J* = 8.4 Hz, 1H, NH Phe), 4.65 (dd, *J* = 12.9, 8.0 Hz, 1H, α-Phe), 4.42 (s, 1H, H-5 Sor), 4.36 (s, 1H, H-4 Sor), 4.25 (s, 1H, H-3 Sor), 4.17 (dd, *J* = 18.5, 4.9 Hz, 1H, CH_2_ Gly), 4.14–4.10 (m, 2H, H-6 Sor), 4.07 (dd, *J* = 18.5, 4.7 Hz, 1H, CH_2_ Gly), 3.76 (s, 3H, CH_3_ Gly), 3.42 (dd, *J* = 14.2, 4.7 Hz, 1H, β-Phe), 3.06 (dd, *J* = 14.2, 8.2 Hz, 1H, β-Phe), (1.51, 1.44, 1.40, 1.36) (21H, CH_3_ isop, CH_3_ Boc). ^13^C NMR (CDCl_3_) δ (170.8, 170.1, 165.3) (CO), 155.4 (CO Boc), 136.6 (C Ph), (129.8, 128.7, 126.9) (CH Ph), (114.1, 112.8) (C isop), 97.8 (C-2 Sor), 85.5 (C-5 Sor), 80.0 (C Boc), 74.2 (C-3 Sor), 72.7 (C-4 Sor), 60.5 (C-6 Sor), 54.6 (α-Phe), 52.5 (CH_3_ Gly), 41.8 (CH_2_ Gly), 38.4 (β-Phe), (29.1, 28.4, 27.7, 26.5) (CH_3_ isop), 18.9 (CH_3_ Boc). HRMS (MALDI–TOF/TOF) *m*/*z*: calcd. for C_30_H_42_NO_12_Na [M + Na]^+^ 645.2635; found 645.2641.

#### 3.1.19. 2-(Cyclohexylamino)-1-((3aR,5S,6R,6aR)-5-((R)-2,2-dimethyl-1,3-dioxolan-4-yl)-2,2-dimethyltetrahydrofuro[2,3-d][1,3]dioxol-6-yl)-2-oxoethyl acetate (**6a**)

Yield 72% (32 mg); colorless oil; R_f_ (DS1, DS2) = 0.37, (EtOAc/petroleum ether 1:1, *v*/*v*); d.r. 90:10. ^1^H NMR (CDCl_3_) Chemical shifts are given for both isomers. δ = 6.04 (d, *J* = 7.9 Hz, 1H, NH), 5.75 (d, *J* = 3.6 Hz, 0.9H, H-3’ Alo), 5.73 (d, *J* = 3.7 Hz, 0.1H, H-3’ Alo), 5.37 (d, *J* = 6.4 Hz, 0.1H, H-1 Alo), 5.21 (d, *J* = 8.7 Hz, 0.9H, H-1 Alo), 4.69–4.62 (m, 1H, H-2 Alo), 4.27–4.16 (m, 2H, H-4,5 Alo), 4.03 (dd, *J* = 8.4, 6.5 Hz, 1H, H-6 Alo), 3.95 (dd, *J* = 8.4, 6.6 Hz, 0.9H, H-6 Alo), 3.88 (dd, *J* = 8.1, 5.4 Hz, 0.1H, H-6 Alo), 3.76 (m, 1H, CH-1 CyHex), 2.51 (m, 0.1H, H-3 Alo), 2.40–2.28 (m, 0.9H, H-3 Alo), 2.11 (s, 2.7H, CH_3_ Ac), 2.09 (s, 0.3H, CH_3_ Ac), 1.86 (s, 2H, CyHex), 1.70–1.54 (m, 3H, CyHex), 1.50 (s, 3H, CH_3_ isop), 1.40 (s, 3H, CH_3_ isop), 1.38–1.33 (m, 2H, CyHex), 1.32 (s, 3H, CH_3_ isop), 1.27 (s, 3H, CH_3_ isop), 1.20–1.11 (m, 3H, CyHex). ^13^C NMR (CDCl_3_) δ (170.2, 167.2) (CO), (112.7, 109.8) (C isop), (105.1, 104.6) (C-1 Alo), (81.6, 81.4, 80.3, 78.5, 76.9, 71.4, 71.3) (C-2,3’,4,5), (66.9, 65.8) (C-6 Alo), (48.6) (C-3 Alo), (48.4, 48.3) (CH CyHex), 32.9 (CyHex), (27.0, 26.6, 26.5, 25.7) (CH_3_ isop), (25.7, 24.8) (CyHex), 21.1 (CH_3_ Ac). HRMS (MALDI–TOF/TOF) *m*/*z*: calcd. for C_22_H_35_NO_8_Na [M + Na]^+^ 464.2260; found 464.2254.

#### 3.1.20. Methyl 2-(2-acetoxy-2-((3aS,5S,6R,6aS)-5-((R)-2,2-dimethyl-1,3-dioxolan-4-yl)-2,2-dimethyltetrahydrofuro[2,3-d][1,3]dioxol-6-yl)acetamido) acetate (**6c**)

Yield 74% (32 mg); colorless oil; R_f_ (DS1, DS2) = 0.38, (EtOAc/petroleum ether 1:1, *v*/*v*); d.r. 90:10. ^1^H NMR (CDCl_3_) Chemical shifts are given for both isomers. δ = 6.77 (s, 0.9H, NH), 6.74 (s, 0.1H, NH), 5.76 (d, *J* = 3.6 Hz, 0.9H, H-3’ Alo), 5.73 (d, *J* = 3.7 Hz, 0.1H, H-3’ Alo), 5.48 (d, *J* = 6.0 Hz, 0.1H, H-1 Alo), 5.38 (d, *J* = 8.2 Hz, 0.9H, H-1 Alo), 4.82 (t, *J* = 4.1 Hz, 0.9H, H-2 Alo), 4.77 (t, *J* = 4.1 Hz, 0.1H, H-2 Alo), 4.26 (dd, *J* = 9.7, 5.0 Hz, 0.9H, H-4 Alo), 4.22–4.06 (m, 2.1H, H-4,5 Alo, CH_2_ Gly), 4.05–4.00 (m, 1H, H-6 Alo), 3.94 (dd, *J* = 8.2, 6.9 Hz, 0.9H, H-6 Alo), 3.87 (dd, *J* = 18.3, 4.4 Hz, 1.1H, H-6 Alo, CH_2_ Gly), 3.74 (s, 0.3H, CH_3_ Gly), 3.72 (s, 2.7H, CH_3_ Gly), 2.52–2.45 (m, 0.1H, H-3 Alo), 2.37–2.30 (m, 0.9H, H-3 Alo), 2.12 (s, 2.7H, CH_3_ Ac), 2.10 (s, 0.3H, CH_3_ Gly), 1.77 (s, 0.3H, CH_3_ isop), 1.50 (s, 0.3H, CH_3_ isop), 1.47 (s, 2.7H, CH_3_ isop), 1.39 (s, 2.7H, CH_3_ isop), 1.30 (s, 3H, CH3 isop), 1.28 (s, 3H, CH_3_ isop). ^13^C NMR (CDCl_3_) δ (170.1, 170.0, 168.8) (CO), (112.7, 109.8) (C isop), (105.1, 104.6) (C-1 Alo), (81.3, 81.2, 79.9, 78.4, 77.3, 76.9, 71.0, 70.7) (C-2,3’,4,5), (67.2, 66.0) (C-6 Alo), (52.7, 52.5) (CH_3_ Gly), (48.8, 48.7, 48.3) (CH CyHex, C-3 Alo)), (41.3, 41.2) (CH_2_ Gly), (26.9, 26.8, 26.7, 26.5, 26.4, 25.6, 25.2) (CH_3_ isop), (21.0, 20.9) (CH_3_ Ac). HRMS (MALDI–TOF/TOF) *m*/*z*: calcd. for C_19_H_29_NO_10_Na [M + Na]^+^ 454.1689; found 454.1696.

#### 3.1.21. 1-((3aS,6aS)-5-(2,2-Dimethyl-1,3-dioxolan-4-yl)-2,2-dimethyltetrahydrofuro[2,3-d][1,3]dioxol-6-yl)-2-((2-methoxy-2-oxoethyl)amino)-2-oxoethyl 2-((tert-butoxycarbonyl)amino)-3-phenylpropanoate (**6d**)

Yield 77% (49 mg); colorless oil; R_f_ (DS1,2) = 0.47 (EtOAc/petroleum ether 1:1, *v*/*v*); d.r. 90:10. ^1^H NMR (CDCl_3_) δ 7.33–7.16 (m, 5H, Ph), 6.92 (s, 1H, NH), 5.75 (d, *J* = 3.7 Hz, 1H, H-3’ Alo), 5.49 (d, *J* = 7.6 Hz, 1H, H-1 Alo), 5.01 (d, *J* = 7.4 Hz, 1H, NH Phe), 4.78 (t, *J* = 4.0 Hz, 1H, α-Phe), 4.59 (dd, *J* = 13.3, 6.7 Hz, 1H, H-2 Alo), 43.1 (dd, *J* = 9.7, 4.4 Hz, 1H, H-5 Alo), 4.12 (ddd, *J* = 22.6, 11.3, 5.5 Hz, 2H, H-4 Alo, CH_2_ Gly), 3.98 (d, *J* = 6.5 Hz, 2H, H-6 Alo), 3.86 (dd, *J* = 18.0, 4.9 Hz, 1H, CH_2_ Gly), 3.72 (s, 3H, CH_3_ Gly), 3.22 (dd, *J* = 14.1, 5.7 Hz, 1H, β-Phe), 3.07 (dd, *J* = 14.0, 7.1 Hz, 1H, β-Phe), 2.35 (ddd, *J* = 9.9, 7.8, 4.6 Hz, 1H, H-3 Alo), 1.57–1.28 (m, 21H, CH_3_ Boc, CH_3_ isop). ^13^C NMR (CDCl_3_) δ (171.1, 169.9, 168.6) (CO), 155.7 (CO Boc), 136.2 (C Ph), (129.6, 128.8, 127.3) (CH Ph), (112.7, 110.0) (C isop), 104.6 (C-1 Alo), 81.3 (C-2 Alo), 80.5 (C Boc), 79.5 (C-4 Alo), 76.6 (C-5 Alo), 71.3 (C-3’ Alo), 65.8 (C-6 Alo), 54.8 (α-Phe), 52.5 (CH_3_ Gly), 48.2 (C-3 Alo), 41.3 (CH_2_ Gly), 37.9 (β-Phe), 28.49 (CH_3_ Boc), (26.86, 26.62, 26.39, 25.44) (CH_3_ isop).HRMS (MALDI–TOF/TOF) *m*/*z*: calcd. for C_31_H_44_N_2_O_12_Na [M + Na]^+^ 659,2792; found 659,2790.

#### 3.1.22. 2-(Cyclohexylamino)-2-oxo-1-((4R,4’R,5S)-2,2,2’,2’-tetramethyl-[4,4’-bi(1,3-dioxolan)]-5-yl)ethyl acetate (**7a**)

Yield 81% (32 mg); colorless oil; R_f_ (DS1) = 0.50, R_f_ (DS2) = 0.48 (EtOAc/petroleum ether 1:1, *v*/*v*); d.r. 55:45. ^1^H NMR (CDCl_3_) δ = 5.95 (d, *J* = 7.9 Hz, 0.55H, NH), 5.77 (d, *J* = 8.1 Hz, 0.45H, NH), 5.37 (d, *J* = 2.7 Hz, 0.55H, H-1 Ara), 5.28 (d, *J* = 2.3 Hz, 0.45H, H-1 Ara), 4.37 (dd, *J* = 7.5, 2.3 Hz, 0.45H, H-2 Ara), 4.32 (t, *J* = 7.8 Hz, 0.55H, H-3 Ara), 4.10 (dd, *J* = 8.0, 2.7 Hz, 0.55H, H-2 Ara), 4.08–3.99 (m, 2H, H-4, H-5 Ara), 3.94 (dd, *J* = 8.4, 4.9 Hz, 0.55H, H-5 Ara), 3.89 (m, 0.45H, H-5 Ara), 3.78–3.70 (m, 1.45H, H-3 Ara, CH-1 CyHex), 2.15 (s, 1.35H, CH_3_ Ac), 2.14 (s, 1.65H, CH_3_ Ac), 1.85 (m, 2H, CyHex), 1.64 (m, 2H, CyHex), 1.56 (m, 1H, CyHex), 1.45 (s, 2H, CH_3_ isop), 1.36–1.26 (m, 12H, CH_3_ isop, CyHex), 1.17–1.07 (m, 3H, CyHex). ^13^C NMR (CDCl_3_) δ = (169.3, 169.13, 166.7, 165.9) (CO), (110.5, 110.2, 110.2, 110.1) (C isop), (80.1, 79.4) (C-2 Ara), (77.4, 77.2) (C-3 Ara), (77.1, 76.9) (C-4 Ara), (73.7, 72.7) (C-1 Ara), 67.4 (C-5 Ara), (48.4, 48.3) (CH CyHex), (33.2, 33.1, 33.1, 33.0) (CyHex), (27.4, 27.4, 27.0, 27.0, 26.7) (CH_3_ isop), (25.7, 25.7) (CyHex), (25.6, 25.3) (CH_3_ isop), (24.9, 24.8) (CyHex), (21.1, 21.0) (CH_3_ Ac). HRMS (MALDI–TOF/TOF) *m*/*z*: calcd. for C_20_H_33_NO_7_Na [M + Na]^+^ 422.2155; found 422.2138.

#### 3.1.23. 2-((2-Methoxy-2-oxoethyl)amino)-2-oxo-1-((4R,4’R,5S)-2,2,2’,2’-tetramethyl-[4,4’-bi(1,3-dioxolan)]-5-yl)ethyl 2-((tert-butoxycarbonyl)amino)-3-phenylpropanoate (**7d**)

Yield 78% (46 mg); colorless oil; R_f_ (DS1) = 0.48 (EtOAc/petroleum ether 1:1, *v*/*v*); d.r. 55:45. ^1^H NMR (CDCl_3_) δ 7.60 (m, 0.55H, NH), 7.36–7.25 (m, 5H, CH Ph), 7.11 (m, 0.45H, NH), 5.50 (d, *J* = 2.7 Hz, 0.55H, H-1 Ara), 5.44 (d, *J* = 1.6 Hz, 0.45H, H-1 Ara), 5.12 (d, *J* = 6.0 Hz, 0.45H, α-Phe), 4.97 (d, *J* = 7.1 Hz, 0.55H, α-Phe), 4.65–4.45 (m, 1.45H, H-2 Ara, NH), 4.29 (t, *J* = 7.4 Hz, 0.55H, H-3 Ara), 4.15–3.92 (m, 5.45H, H-2, H-3, H-4, H-5, CH_2_ Gly), 3.81 (td, *J* = 7.9, 3.4 Hz, 0.55H, H-5 Ara), 3.73 (s, 1.65H, CH_3_ Gly), 3.71 (s, 1.35H, CH_3_ Gly), 3.21–3.05 (m, 2H, β-Phe), 1.47–1.27 (m, 21H, CH_3_ Boc, CH_3_ isop). ^13^C NMR (CDCl_3_) δ (171.7, 170.6, 169.8, 169.6, 168.0, 167.0) (CO), 156.0 (CO Boc), (135.9, 135.5) (C Ph), (129.4, 129.3, 129.2, 129.0, 127.6, 127.4) (CH Ph), (110.5, 110.3, 110.0, 109.9) (C isop), (81.0, 80.8) (C Boc), (79.6, 79.2) (C-2 Ara), 77.1 (C-3 Ara), (76.8, 76.6) (C-4 Ara), (74.2, 72.9) (C-1 Ara), (67.47, 67.2) (C-5 Ara), (55.9, 54.8) (α-Phe), (52.4, 52.3) (CH_3_ Gly), (41.3, 41.1) (CH_2_ Gly), (37.8, 37.7) (β-Phe), (28.4, 28.4) (CH_3_ Boc), (27.4, 27.4, 26.9, 26.9, 26.7, 26.6, 25.5, 25.1) (CH_3_ isop). HRMS (MALDI–TOF/TOF) *m*/*z*: calcd. for C_29_H_42_N_2_O_11_Na [M + Na]^+^ 617.2686; found 617.2687.

#### 3.1.24. (3aR,5S,6aR)-6-(Cyclohexylcarbamoyl)-5-((R)-2,2-dimethyl-1,3-dioxolan-4-yl)-2,2-dimethyltetrahydrofuro[2,3-d][1,3]dioxol-6-yl acetate (**8a**)

Yield 67% (29 mg); colorless oil; R_f_ (DS1) = 0.70, R_f_ (DS2) = 0.50 (EtOAc/petroleum ether 1:1, *v*/*v*); d.r. 55:45. ^1^H NMR (CDCl_3_) Major isomer: δ = 7.40 (d, *J* = 7.5 Hz, 1H, NH), 5.88 (d, *J* = 3.6 Hz, 1H, H-1 GlcF), 4.87 (d, *J* = 3.6 Hz, 1H, H-2, GlcF), 4.45 (t, *J* = 4.7 Hz, 1H, H-5, GlcF), 4.42 (d, *J* = 2.8 Hz, 1H, H-4 GlcF), 4.13 (dd, *J* = 8.4, 6.5 Hz, 1H, H-6a GlcF), 4.06 (dd, *J* = 8.4, 6.9 Hz, 1H, H-6b, GlcF), 3.80–3.73 (m, 1H, CH-1 CyHex), 2.09 (m, 3H, CH_3_ Ac), 1.93–1.82 (m, 2H, CyHex), 1.67–1.60 (m, 2H, CyHex), 1.59 (m, 5H, CH_3_ isop, CyHex), 1.41 (s, 3H, CH_3_ isop), 1.36 (m, 4H, CH_3_ isop, CyHex), 1.33 (s, 3H, CH_3_ isop), 1.27–1.15 (m, 3H, CyHex). Minor isomer: δ = 6.17 (d, *J* = 8.1 Hz, 1H, NH), 5.85 (d, *J* = 3.8 Hz, 1H, H-1 GlcF), 5.33 (d, *J* = 3.8 Hz, 1H, H-2 GlcF), 4.10–4.06 (m, 1H, H-6a GlcF), 4.02 (d, *J* = 9.3 Hz, 1H, H-5 GlcF), 3.97–3.91 (m, 2H, H-4, H-6b GlcF), 3.83–3.77 (m, 1H, CH-1 CyHex), 2.11 (s, 3H, CH_3_ Ac), 1.85 (ddd, *J* = 27.8, 12.4, 3.5 Hz, 2H, CyHex), 1.69–1.54 (m, 4H, CyHex), 1.50 (s, 3H, CH_3_ isop), 1.46 (s, 3H, CH_3_ isop), 1.41–1.33 (m, 1H, CyHex), 1.33 (s, 3H, CH_3_ isop), 1.31 (s, 3H, CH_3_ isop), 1.22–1.08 (m, 3H, CyHex). ^13^C NMR (CDCl_3_) Major isomer: δ = (170.0, 163.9) (CO), (114.0, 108.7) (C isop), 105.4 (C-1 GlcF), 85.6 (C-3 GlcF), 82.8 (C-2 GlcF), 82.3 (C-4 GlcF), 73.5 (C-5 GlcF), 65.0 (C-6 GlcF), 48.4 (CH CyHex), (32.7, 32.7) (CyHex), (27.0, 26.7, 26.5) (CH_3_ isop), 25.8 (CyHex), 25.5 (CH_3_ isop), (24.6, 24.5) (CyHex), 21.5(CH_3_ Ac). Minor isomer: δ = (169.0, 166.6) (CO), (112.8, 110.1) (C isop), 105.7 (C-1 GlcF), 85.3 (C-3 GlcF), 80.9 (C-2 GlcF), 80.4 (C-4 GlcF), 74.6 (C-5 GlcF), 68.4 (C-6 GlcF), 48.2 (CH CyHex), (33.1, 32.7) (CyHex), (27.1, 27.0, 26.8, 25.8) (CH_3_ isop), (25.7, 24.7, 24.7) (CyHex), 21.0 (CH_3_ Ac). HRMS (MALDI–TOF/TOF) *m*/*z*: calcd. for C_21_H_33_NO_8_ [M + H]^+^ 428.2284; found 428.2302.

#### 3.1.25. (3aR,4′R,7aR)-7-(Cyclohexylcarbamoyl)-2,2,2′,2′-tetramethyltetrahydrospiro[[1,3]dioxolo[4,5-c]pyran-6,4′-[1,3]dioxolan]-7-yl acetate (**9a**)

Yield 51% (22 mg); colorless oil; R_f_ (DS1) = 0.80, R_f_ (DS2) = 0.40 (EtOAc/petroleum ether 1:1, *v*/*v*); d.r. 55:45. ^1^H NMR (CDCl_3_) Major isomer: δ = 5.99 (d, *J* = 7.7 Hz, 1H, NH), 5.30 (d, *J* = 7.0 Hz, 1H, H-4 FruP), 4.60 (dt, *J* = 7.3, 3.7 Hz, 1H, H-5 FruP), 4.57 (d, *J* = 9.6 Hz, 1H, H-1a FruP), 4.07 (m, 1H, H-1b FruP), 4.05 (m, 1H, H-6a FruP), 3.80–3.74 (m, 1H, CH-1 CyHex), 3.68 (dd, *J* = 12.8, 3.4 Hz, 1H, H-6b FruP), 2.11 (s, 3H, CH3 Ac), 1.86 (t, *J* = 17.8 Hz, 2H, CyHex), 1.65–1.57 (m, 4H, CyHex), 1.47 (s, 6H, CH3 isop), 1.46 (s, 3H, CH3 isop), 1.38 (s, 1H, CyHex), 1.30 (s, 3H, CH3 isop), 1.21–1.06 (m, 3H, CyHex). Minor isomer: δ = 6.66 (d, *J* = 7.3 Hz, 1H, NH), 5.08 (d, *J* = 7.2 Hz, 1H, H-4 FruP), 4.31 (d, *J* = 9.9 Hz, 1H, H-1a FruP), 4.30–4.24 (m, 1H, H-5 FruP), 4.20 (dd, *J* = 12.6, 3.3 Hz, 1H, H-6a FruP), 3.93 (d, *J* = 9.9 Hz, 1H, H-1b FruP), 3.79 (dd, *J* = 12.6, 0.9 Hz, 1H, H-6b FruP), 3.77–3.73 (m, 1H, CyHex CH-1), 2.12 (s, 3H, CH_3_ Ac), 1.92 (dd, *J* = 33.6, 9.8 Hz, 2H, CyHex), 1.66 (m, 2H, CyHex), 1.58 (m, 2H, CyHex), 1.51 (s, 3H, CH_3_ isop), 1.49 (s, 3H, CH_3_ isop), 1.43 (s, 3H, CH_3_ isop), 1.36 (s, 3H, CH_3_ isop), 1.33 (m, 1H, CyHex), 1.23–1.12 (m, 3H, CyHex). ^13^C NMR (CDCl_3_) Major isomer: δ = 168.8, 166.6 (CO), 110.6, 109.5 (C isop), 104.5 (C-2 FruP), 80.5 (C-3 FruP), 73.1 (C-4 FruP), 72.8 (C-5 FruP), 72.3 (C-1 FruP), 64.7 (C-6 FruP), 48.3 (CH-1 CyHex), 32.9, 32.7 (CyHex), 26.9, 26.6, 26.1, 25.8, 25.0, 24.7, 24.6 (CH_3_ isop), 21.6 (CH_3_ Ac). Minor isomer: δ = 169.3, 164.6 (CO), 111.6, 110.8 (C isop), 103.9 (C-2 FruP), 81.0 (C-3 FruP), 73.60 (C-1 FruP), 73.4 (C-4 FruP), 73.0 (C-5 FruP), 64.4 (C-6 FruP), 48.9 (CH-1 CyHex), 32.9, 32.7 (CyHex), 26.4, 26.3, 26.2 (CH_3_ isop), 25.8 (CyHex), 25.3 (CH_3_ isop), 24.9 (CyHex), 21.7 (CH_3_ Ac). HRMS (MALDI–TOF/TOF) *m*/*z*: calcd. for C_21_H_33_NO_8_Na [M + Na]^+^ 450.2104; found 450.2107.

### 3.2. Post-Condensation Modification of Selected Passerini Products

Boc deprotection and coupling of amino acid: Passerini product 2d (10 mg, 0.016 mmol) was dissolved in trifluoroacetic acid (TFA)/DCM (1:1, *v*/*v*, 100 µL), the reaction was stirred at room temperature for 2 min, and the reaction mixture was evaporated with toluene. Boc–Pro–OH (4 mg, 0.018 mmol, 1.2 eq), benzotriazol-1-yloxytris(dimethylamino)phosphonium (BOP, 10 mg, 0.023 mmol, 1.5 eq), and hydroxybenzotriazole (HOBt, 3.5 mg, 0.023 mmol, 1.5 eq) were dissolved in 1 mL DCM/DMF (1:1), followed by the addition of the Boc-deprotected compound (8 mg, 0.015 mmol) and *N*-methylmorpholine (NMM, 2eq). The reaction mixture was stirred for 24 h. The product was purified by flash column chromatography (petrol ether/ethyl acetate, *v*/*v* = 1:6).

#### 3.2.1. tert-Butyl 2-((1-(2-((2-methoxy-2-oxoethyl)amino)-2-oxo-1-((3aR,5aS,8aS,8bR)-2,2,7,7-tetramethyltetrahydro-3aH-bis([1,3]dioxolo)[4,5-b:4′,5′-d]pyran-5-yl)ethoxy)-1-oxo-3-phenylpropan-2-yl)carbamoyl) pyrrolidine-1-carboxylate (**2j**)

Yield 78% (9 mg); colorless oil; R_f_ (DS1,2) = 0.55 (EtOAc/petroleum ether 6/1, *v*/*v*); d.r. 70:30. ^1^H NMR (MeOD) δ = 7.36–7.18 (m, 5H, Ph), 5.52 (d, *J* = 4.9 Hz, 0.3H, H-1 Gal), 5.49 (d, *J* = 4.7 Hz, 0.7H, H-1 Gal), 5.44 (m, 0.3H, H-6 Gal) 5.13 (m, 0.7H, H-6 Gal), 4.84 (s, 1H, α-Phe), 4.66 (dd, *J* = 8.0, 2.3 Hz, 1H, H-3 Gal), 4.39 (ddd, *J* = 11.1, 4.7, 2.4 Hz, 1H, H-2 Gal), 4.28–4.18 (m, 2H, H-4,5 Gal), 4.15 (bs, 1H, α-Pro), 4.06–3.92 (m, 2H, CH_2_ Gly), 3.74 (d, *J* = 2.7 Hz, 3H, CH_3_ Gly), 3.44 (m, 1H, δ-Pro), 3.39–3.34 (m, 2H, δ-Pro, β-Phe), 3.01 (bs, 1H, β-Phe), 2.17 (m, 1H, β-Pro), 1.91 (m, 1H, β-Pro), 1.78 (m, 2H, γ-Pro), 1.55 (d, *J* = 11.6 Hz, 3H, CH_3_ isop), 1.46 (d, *J* = 16.2 Hz, 6H, CH_3_ isop), 1.37–1.24 (m, 12H, CH_3_ isop, Boc). ^13^C NMR (MeOD) δ (170.0, 169.8, 169.3, 169.1, 168.5, 167.7, 167.4) (CO), 136.4 (C Ph), (128.4, 127.6, 125.9) (CH Ph), (108.9, 108.5, 108.3) (C isop), (95.9, 95.7) (C-1 Gal), (72.5, 71.1, 70.3, 70.3, 70.2, 70.0, 69.5, 67.2, 66.5) (C-2,3,4,5,6 Gal), 59.7 (α-Pro), (53.2, 52.9) (α-Phe), (50.7, 50.7) (CH_3_ Gly), 45.9 (δ-Pro), (40.1, 39.9) (CH_2_ Gly), (36.3, 36.0) (β-Phe), 30.3 (Boc), (26.7, 24.5, 24.4, 23.3) (CH_3_ isop), (22.8, 22.5) (γ-Pro). HRMS (MALDI–TOF/TOF) *m*/*z*: calcd. for C_35_H_49_N_3_O_13_Na [M + Na]^+^ 742.3163; found 742.3190.

Boc deprotection and coupling of amino acid: Passerini product **5d** (13 mg, 0.02 mmol) was dissolved in TFA/DCM (1:1, *v*/*v*, 100 µL), the reaction was stirred at room temperature for 2 min, and then the solvent was evaporated. Boc–Phe–OH (6.4 mg, 0.024 mmol, 1.2 eq), BOP (13 mg, 0.03 mmol, 1.5 eq), and HOBt (4.6 mg, 0.03 mmol, 1.5 eq) were dissolved in 1 mL DCM/DMF (1:1) followed by the addition of the Boc-deprotected compound and NMM (2 eq). The reaction mixture was stirred for 24 h, the solvent was evaporated, and the product was extracted with DCM. The product was purified by flash column chromatography (petrol ether/ethyl acetate, *v*/*v* = 1/1).

#### 3.2.2. (S)-(S)-1-((3aR,5S,6R,6aS)-6-Hydroxy-5-(hydroxymethyl)-2,2-dimethyltetrahydrofuro[2,3-d][1,3]dioxol-3a-yl)-2-((2-methoxy-2-oxoethyl)amino)-2-oxoethyl 2-((S)-2-((tert-butoxycarbonyl)amino)-3-phenylpropanamido)-3-phenylpropanoate (**5j**)

Yield 65% (9.5 mg); colorless oil; R_f_ = 0.30 (EtOAc/petroleum ether 1/1, *v*/*v*). ^1^H NMR (CDCl_3_): δ = 7.68 (s, 1H, NH), 7.38–7.16 (m, 8H, CH Ph), 6.91 (dd, *J* = 6.4, 2.9 Hz, 2H, CH Ph), 6.00 (d, *J* = 5.4 Hz, 1H, NH Phe), 5.77 (s, 1H, H-1 Sor), 5.16 (d, *J* = 7.5 Hz, 1H, NH Phe), 4.93 (s, 1H, H-5 Sor), 4.60 (dt, *J* = 8.8, 5.3 Hz, 1H, α-Phe), 4.35–4.12 (m, 4H, α-Phe, CH_2_ Gly, H-3,4 Sor), 3.91–3.78 (m, 3H, CH_2_ Gly, H-6 Sor), 3.75 (s, 3H, CH_3_ Gly), 3.14 (dd, *J* = 14.3, 5.1 Hz, 1H, β-Phe), 3.02 (d, *J* = 7.1 Hz, 2H, β-Phe), 2.94–2.81 (m, 1H, β-Phe), 1.91 (bs, 2H, OH), 1.49 (s, 3H, CH_3_ isop), 1.34–1.23 (m, 12H, CH_3_ isop, Boc). ^13^C NMR (CDCl_3_): δ = (173.0, 170.0, 169.30, 168.3) (CO), (137.1, 135.2) (C Ph), (129.9, 129.2, 129.1, 129.1, 127.72, 127.5) (CH Ph), 112.7 (C isop), 112.0 (C-2 Sor), (88.0, 82.7, 80.6, 76.7, 75.2) (C-1,3,4,5 Sor), 61.2 (C-6 Sor), (55.9, 54.3) (α-Phe), 52.8 (CH_3_ Gly), 41.1 (CH_2_ Gly), (38.6, 36.9) (β-Phe), 28.5 (Boc), (27.4, 26.3) (CH_3_ isop). HRMS (MALDI–TOF/TOF) *m*/*z*: calcd. for C_35_H_49_N_3_O_13_Na [M + Na]^+^ 742.3163; found 742.3190.

Fmoc deprotection: A solution of 20% piperidine in DMF (0.2 mL) was added to the Passerini product **2k** (20 mg, 0.03 mmol), the mixture was stirred at room temperature for 15 min, and then the solvent was evaporated. The product was purified by flash column chromatography (petrol ether/ethyl acetate, *v*/*v* = 1/1).

#### 3.2.3. 2-((2-Methoxy-2-oxoethyl)amino)-2-oxo-1-((3aR,5aS,8aS,8bR)-2,2,7,7-tetramethyltetrahydro-3aH-bis([1,3]dioxolo)[4,5-b:4′,5′-d]pyran-5-yl)ethyl 2-amino-3-phenylpropanoate (**2l**)

Yield 77% (12 mg); colorless oil; R_f_ (DS1,2) = 0.40 (EtOAc/petroleum ether 1:1, *v*/*v*); d.r. 70:30. ^1^H NMR (CDCl_3_): δ = 7.25 (m, 5H, Ph), 5.45 (d, *J* = 4.9 Hz, 0.7H, H-1 Gal), 5.41 (d, *J* = 4.6 Hz, 0.3H, H-1 Gal), 4.91–4.84 (m, 0.7H, H-6 Gal), 4.75 (td, *J* = 8.3, 4.9 Hz, 0.3H, H-6 Gal), 4.62 (ddd, *J* = 14.4, 8.0, 2.5 Hz, 1H, H-3 Gal), 4.46 (dd, *J* = 8.0, 1.6 Hz, 0.3H, α-Phe), 4.40 (dd, *J* = 7.9, 1.7 Hz, 0.7H, α-Phe), 4.31 (dd, *J* = 4.9, 2.5 Hz, 1H, H-2 Gal), 4.28–3.87 (m, 4H, CH_2_ Gly, H-5,4 Gal), 3.72 (m, 3H, CH_3_ Gly), 3.29 (ddd, *J* = 37.2, 14.3, 5.2 Hz, 1H, β-Phe), 3.15–3.10 (m, 1H, β-Phe), 2.95 (s, 1H, NH), 2.88 (s, 1H, NH), 1.51–1.25 (m, 12H, CH_3_ isop). ^13^C NMR (CDCl_3_): δ 171.7, 170.4, 170.3, 170.1, 169.8, 169.5 (CO), 136.1, 135.8 (C Ph), 128.8, 128.8, 128.2, 126.5, 126.4 (CH isop), 109.7, 109.1, 109.1, 108.8 (CH_3_ isop), 95.9, 95.4 (C-1 Gal), 73.5, 73.5, 70.3, 70.2, 70.2, 70.0, 69.8, 68.8, 67.8, 66.5 (C-2,3,4,5,6 Gal), 53.6, 53.2 (α-Phe), 51.7, 51.5 (CH_3_ Gly), 40.7, 40.6 (CH_2_ Gly), 36.9, 36.1 (β-Phe), 25.5, 25.4, 25.3, 25.2, 24.6, 24.3, 23.7, 23.3 (CH_3_ isop). HRMS (MALDI–TOF/TOF) *m*/*z*: calcd. for C_25_H_34_N_2_O_10_Na [M + Na]^+^ 545.2111; found 545.2109.

### 3.3. Base-Mediated Deprotection of Selected Passerini Products

General procedure: the Passerini product (0.035 mmol) was dissolved in MeOH (0.5 mL), and 1N NaOH (2 eq) was added. The reaction was stirred under reflux (10 min) or at room temperature (30 min), followed by TLC (EtOAc/EtOH/HOAc/H_2_O 70:10:2:2, *v*/*v*). The solvent was evaporated, and the product was purified by flash column chromatography.

#### 3.3.1. 2-((S)-2-Hydroxy-2-((3aS,5aR,8aR,8bS)-2,2,7,7-tetramethyltetrahydro-3aH-bis([1,3]dioxolo)[4,5-b:4′,5′-d]pyran-3a-yl)acetamido)acetic acid (**1m**)

Yield 91% (11.5 mg); colorless oil; R_f_ = 0.74 (EtOAc/EtOH/HOAc/H_2_O 70:10:2:2, *v*/*v*). ^1^H NMR (CDCl_3_) δ = 7.36–7.29 (m, 1H, NH), 5.52 (bs, 2H, OH), 4.66 (d, *J* = 2.6 Hz, 1H, H-3 Fru), 4.61 (dd, *J* = 7.8, 2.6 Hz, 1H, H-4 Fru), 4.22 (d, *J* = 8.2 Hz, 1H, H-5 Fru), 4.16–4.09 (m, 2H, H-1 Fru, CH_2_ Gly), 3.99 (dd, *J* = 18.1, 4.6 Hz, 1H, CH_2_ Gly), 3.90 (d, *J* = 11.7 Hz, 1H, H-6 Fru), 3.80 (d, *J* = 12.9 Hz, 1H, H-6 Fru), 1.47 (s, 3H, CH_3_ isop), 1.46 (s, 3H, CH_3_ isop), 1.38 (s, 3H, CH_3_ isop), 1.33 (s, 3H, CH_3_ isop). ^13^C NMR (CDCl_3_) δ 171.3 (CO), 109.7, 109.4 (C isop), 103.5 (C-2 Fru), 71.6, 70.9, 70.5, 70.4 (C-1,3,4,5 Fru), 61.9 (C-6 Fru), 42.4 (CH_2_ Gly), 26.6, 26.2, 25.7, 24.2 (CH_3_ isop). HRMS (MALDI–TOF/TOF) *m*/*z*: Calcd. for C_15_H_23_NO_9_Na [M + Na]^+^ 384.1271; found 384.1269.

#### 3.3.2. 2-(2-Hydroxy-2-((3aR,5R,5aS,8aS,8bR)-2,2,7,7-tetramethyltetrahydro-3aH-bis([1,3]dioxolo)[4,5-b:4′,5′-d]pyran-5-yl)acetamido)acetic acid (**2m**)

Yield 79% (10 mg); colorless oil; R_f_ (DS1,2) = 0.58 (EtOAc/EtOH/HOAc/H_2_O 70:10:2:2, *v*/*v*); d.r. 70:30. ^1^H NMR (CDCl_3_) δ 7.44 (s, 1H, NH), 5.54 (dd, *J* = 16.6, 4.7 Hz, 1H, H-1 Gal), 5.13 (bs, 2H, OH), 4.61 (dt, *J* = 8.0, 4.0 Hz, 1H, H-3 Gal), 4.48 (d, *J* = 8.1 Hz, 1H, H-4 Gal), 4.38–4.23 (m, 2H, H-2,6 Gal), 4.12–3.96 (m, 3H, CH_2_ Gly, H-5 Gal), 1.53–1.26 (m, 12H, CH_3_ isop). ^13^C NMR (CDCl_3_) δ 173.5, 171.8 (CO), 110.1, 109.8, 109.60, 109.6 (C isop), 96.6, 96.5 (C-1 Gal), 73.7, 72.9, 71.3, 71.2, 70.9, 70.8, 70.7, 70.4, 67.8, 66.7 (C-2,3,4,5,6), 41.78 (CH_2_ Gly), 26.1, 26.1, 26.0, 25.2, 25.1, 24.4, 24.2 (CH_3_ isop). HRMS (MALDI–TOF/TOF) *m*/*z*: calcd. for C_15_H_23_NO_9_Na [M + Na]^+^ 384.1271; found 384.1272.

#### 3.3.3. 2-((S)-2-Hydroxy-2-((3aS,3bR,7aS,8aS)-2,2,5,5-tetramethyltetrahydro-3aH-[1,3]dioxolo[4′,5′:4,5]furo[3,2-d][1,3]dioxin-8a-yl)acetamido)acetic acid (**5m**)

Yield 76% (9.6 mg); colorless oil; R_f_ (DS1,2) = 0.75 (EtOAc/EtOH/HOAc/H_2_O 70:10:2:2, *v*/*v*); d.r. nd. ^1^H NMR (CDCl_3_) δ 7.35 (d, *J* = 5.2 Hz, 1H), 4.63 (s, 1H), 4.32 (d, *J* = 8.0 Hz, 2H), 4.16 (s, 1H), 4.13–3.98 (m, 4H), 1.40 (m, 12H). ^13^C NMR (CDCl_3_) δ 171.1 (CO), 114.5, 113.7 (C isop), 97.9 (C-2 Sor), 85.3 (C-5), 73.7, 73.0, 70.8 (C-1,3,4), 60.5 (C-6), 42.5 (CH_2_ Gly), 29.2, 27.7, 26.6, 18.8 (CH_3_ isop). HRMS (MALDI–TOF/TOF) *m*/*z*: calcd. for C_15_H_23_NO_9_Na [M + Na]^+^ 384.1271; found 384.1281.

#### 3.3.4. 2-(2-((3aS,5S,6R,6aS)-5-((R)-2,2-Dimethyl-1,3-dioxolan-4-yl)-2,2-dimethyltetrahydrofuro[2,3-d][1,3]dioxol-6-yl)-2-hydroxyacetamido)acetic acid (**6m**)

Yield 70% (0.11 mmol, 29 mg); colorless oil; R_f_ (DS1,2) = 0.62 (EtOAc/EtOH/HOAc/H_2_O 70:10:2:2, *v*/*v*); *d.r.* 90:10. ^1^H NMR (CDCl_3_): δ 7.44 (t, *J* = 5.3 Hz, 0.9H, NH), 7.35–7.31 (m, 0.1H, NH), 5.76 (d, *J* = 3.4 Hz, 0.1H, H-3 Alo), 5.74 (d, *J* = 3.5 Hz, 0.9H, H-3 Alo), 5.58 (s, 2H, OH), 4.86 (t, *J* = 4.0 Hz, 0.9H, H-2 Alo), 4.83 (t, *J* = 4.0 Hz, 0.1H, H-2 Alo), 4.63 (d, *J* = 3.3 Hz, 0.1H, H-4 Alo), 4.51 (d, *J* = 7.0 Hz, 0.9H, H-4 Alo), 4.28–4.22 (m, 0.1H, H-5 Alo), 4.20 (dd, *J* = 9.5, 6.7 Hz, 0.9H, H-5 Alo), 4.12–4.01 (m, 3H, H-1 Alo, H-6, CH_2_ Gly), 4.00–3.90 (m, 2H, H-6 Alo, CH_2_ Gly), 2.66 (dt, *J* = 10.2, 3.8 Hz, 0.1H, H-3 Alo), 2.28 (ddd, *J* = 9.7, 7.1, 4.7 Hz, 0.9H, H-3 Alo), 1.54 (s, 0.3H, CH_3_ isop), 1.52 (s, 2.7H, CH_3_ isop), 1.43 (s, 0.3H, CH_3_ isop), 1.41 (s, 2.7H, CH_3_ isop), 1.33 (s, 2.7H, CH_3_ isop), 1.31 (s, 2.7H, CH_3_ isop), 1.28 (s, 0.6H, CH_3_ isop). ^13^C NMR (CDCl_3_): δ 173.2 (CO), (112.7, 110.4) (C isop), 104.7 (C1 Alo), (82.9, 79.5, 77.0, 69.3) (C-2,4,5,3 Alo), 67.2 (C-6 Alo), 51.1 (CH_3_ Gly), 47.6 (C-3 Alo), 41.6 (CH_2_ Gly), (27.0, 26.5, 26.4, 25.5) (CH_3_ isop). HRMS (MALDI–TOF/TOF) *m*/*z*: calcd. for C_16_H_25_NO_9_Na [M + Na]^+^ 398.1427; found 398.1445

## 4. Conclusions

In summary, we systematically validated the reactivity of eight different isopropylidene-protected carbohydrate-derived aldehydes and ketones in the Passerini-type multicomponent reaction. Simple post-condensation modifications allowed to gain more complex structures or versatile building blocks to access new types of biomimetics. The here described methodology can be also applied to other classes of biomolecules, like lipids and peptides, and used as a platform for generating new nature-mimicking systems.

## Supplementary Materials

Supplementary materials can be found at https://www.mdpi.com/1422-0067/20/24/6236/s1.

## Abbreviations

MCRs	multicomponent reactions
DCM	dichloromethane
NMR	nuclear magnetic resonance
DMF	dimethylformamide
BOP	benzotriazol-1-yloxytris(dimethylamino)phosphonium hexafluorophosphate
HOBt	hydroxybenzotriazole
TFA	trifluoroacetic acid
NMM	*N*-methylmorpholine
RT	room temperature
MALDI TOF/TOF	matrix-assisted laser desorption/ionization with time-of-flight mass spectrometer
