# Supplementary material for: C-Linked Glycomimetic Libraries Accessed by the Passerini Reaction
Source: Int J Mol Sci. 2019 Dec 10;20(24):6236. doi: 10.3390/ijms20246236 (PMC6940731; doi:10.3390/ijms20246236)
Supplement: Supplementary file 1 [file ijms-20-06236-s001.pdf]

Supporting Information

C-linked glycomimetic libraries accessed by the Passerini reaction

*Kristina Vlahoviček-Kahlina[1], Josipa Suć Sajko[1], and Ivanka Jerić[1,]**

[1] Ruđer Bošković Institute, Division of Organic Chemistry and Biochemistry, Bijenička cesta 54, 10000 Zagreb, Croatia

Table of Content

General methods	SI-2
Synthesis of aldehydes and ketones	SI-3
Synthesis and characterisation of Passerini products	SI-5
Post-condensation modification of selected Passerini products	SI-17
Base-mediated deprotection of selected Passerini products	SI-19
NMR and HRMS spectra of Passerini products	SI-21
X-ray structure of **2a**	SI-77

General methods

All experiments were monitored by analytical thin layer chromatography (TLC) on Silica Gel 60 F254 plates (Merck; Darmstadt, Germany) after spraying with 10 % H_2SO_4 and heating. Flash column chromatography was performed on silica gel (Merck, 40−63 μm particle size) by standard techniques eluting with solvents as indicated. All NMR experiments were carried out by using Bruker Avance 600 spectrometer (600.13 MHz, ^1H; 150.91 MHz, ^{13}C). Samples in $CDCl_3$ solutions were recorded in 5 mm NMR tubes at 298 K. Chemical shifts in parts per million were referenced to TMS as internal standard. Spectra were assigned based on 2D homonuclear (COSY) and heteronuclear (HMQC, HMBC) experiments. ^1H chemical shifts are assigned to the particular starting component, and the following abbreviations are used: cyclohexyl = CyHex; tetrabutyl = tBu; methyl cyanoacetate = Gly; fructopyranose derived aldehyde **1** = Fru; galactose derived aldehyde **2** = Gal; ribose derived aldehyde **3** = Rib; xylose derived aldehyde **4** = Xyl; sorbose derived aldehyde **5** = Sor; allose derived aldehyde **6** = Alo; arabinose derived aldehyde **7** = Ara; glucofuranose derived ketone **8** = GluF; fructopyranose derived ketone **9** = FruP, respectively.

High resolution mass spectrometry (HRMS) was performed on a MALDI-TOF/TOF spectrometer in positive ionization mode. Calibration type was internal with calibrants produced by matrix ionization dissolved in α-cyano-4-hydroxycinnamic acid matrix. Accurately measured spectra were internally calibrated and elemental analysis was performed on Data Explorer v. 4.9 software with mass accuracy better than 5 ppm.

Experimental Procedures and Spectroscopic Characterization of New Compounds

1. Synthesis of aldehydes/ketones to be used in the Passerini reaction:

General procedure for the syntheisis of aldehydes and ketones:

Isopropylidene-protected alcohols were dissolved in dichloromethane. Dess-Martin periodinane (1.2 eq) was added and the reaction mixture was stirred for 1 h (aldehydes) / 24 h (ketones) at room temperature. The reaction was quenched with solution of $Na_2S_2O_3$/sat. $NaHCO_3$ and the product was extracted with dichloromethane. Organic layer was washed with brine and water, dried over Na_2SO_4 and concentrated under reduced pressure. The products was purified by flash column chromatography (petrol ether/ethyl acetate (v/v = 1/1) to afford corresponding carbonyl products **1-9**.

Aldehyde **1** was synthesized according to a literature procedure. (R.F. Brady, *Carbohydrate Reasearch* 1970 **15** 35-40).

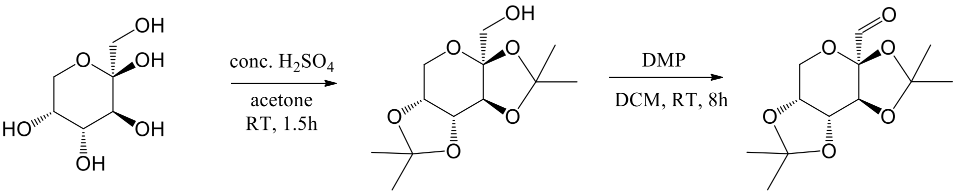

Aldehyde **2** was synthesized according to generale procedure:

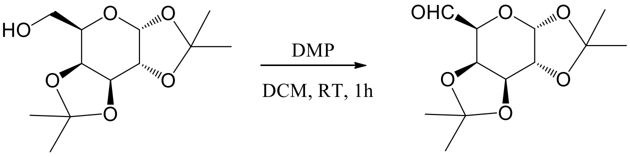

Aldehyde **3** was synthesized according to a literature procedure. (Sairam at al. *Carbohydrate Reasearch* 2003 **338** 303-306):

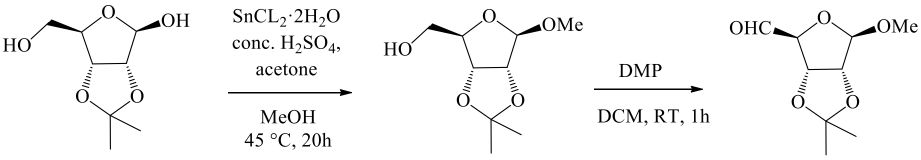

Aldehyde **4** was synthesized according to a literature procedure. (Shun-Yuan Luo at al. *RCS Adv.* 2015 **5** 19027-19033).

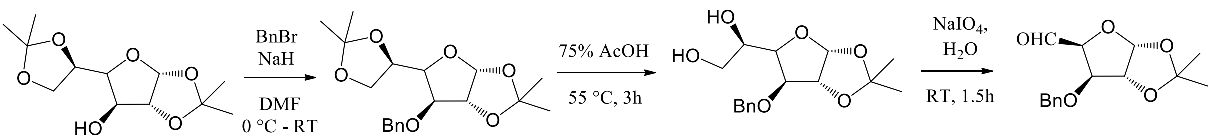

Aldehyde **5** was synthesized according to generale procedure:

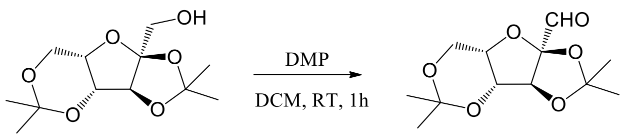

Aldehyde **6** was synthesized according to a literature procedure. (Chun at al. *Nucleosides Nucleotides Nucleic Acid* 2008 **27** 408-420):

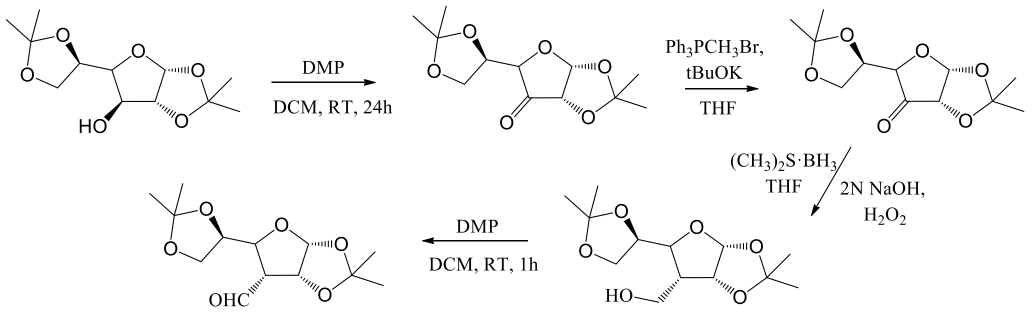

Aldehyde **7** was synthesized according to generale procedure:

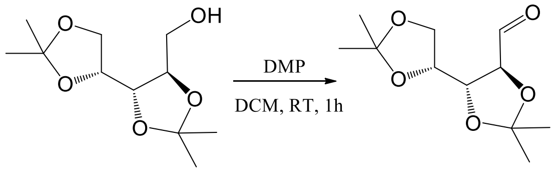

Ketone **8** was synthesized according to generale procedure:

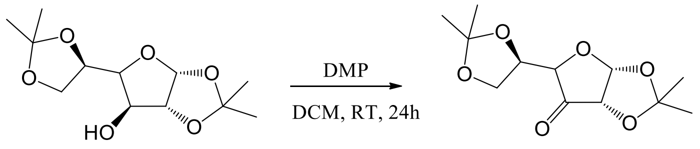

Ketone **9** was synthesized according to a literature procedure. (*Organic Synthesis* 2003 **80** 1-8; *Coll.Voll.* 2009 **11** 177-182)

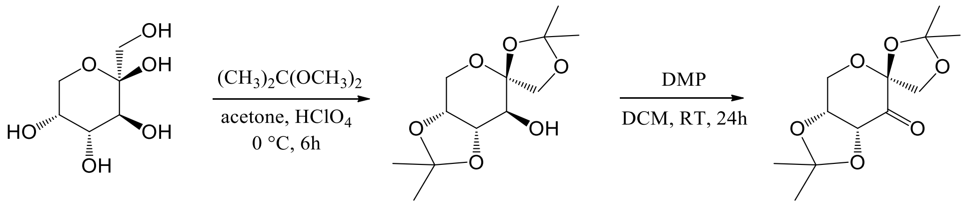

2. Synthesis and characterisation of Passerini products:

General procedure for Passerini reactions:

To a glass vial containing 1 M solution of oxo-compound (0.1 mmol) in DCM under nitrogen were added acid (0.11 mmol) and the isocyanide component (0.11 mmol) dissolved in 100 µL DCM. With all reactants added, the reaction mixture was stirred at room temperature until the consumption of the aldehyde/ketone component, typically 6-24h. The reactions were concentrated under reduced pressure and reaction mixtures were purified by flash column chromatography.

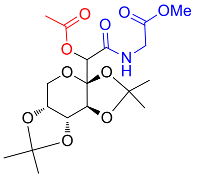

methyl 2-(2-acetoxy-2-((3aR,5aR,8aR,8bS)-2,2,7,7-tetramethyltetrahydro-3aH-bis([1,3]dioxolo)[4,5-b:4',5'-d]pyran-3a-yl)acetamido)acetate (1c) Yield 86 % (36 mg); colorless oil; R_f (DS1,2) = 0.47 (EtOAc/PE 2:1, v/v); *d.r.* 90:10. Chemical shifts are given for both isomers. ^{1}H NMR ($CDCl_3$) δ 6.95 (s, 0.9H, NH), 6.91 (s, 0.1H, NH), 5.50 (s, 0.1H, H-1 Fru), 5.18 (s, 0.9H, H-1 Fru), 4.69 (d, *J* = 2.6 Hz, 0.1H, H-3 Fru), 4.58 (dd, *J* = 7.9, 2.8 Hz, 0.9H, H-4 Fru), 4.54 (dd, *J* = 8.0, 2.6 Hz, 0.1H, H-4 Fru), 4.36 (d, *J* = 2.8 Hz, 0.9H, H-3 Fru), 4.22 (dd, *J* = 7.9, 1.3 Hz, 0.9H, H-5 Fru), 4.17 (dd, *J* = 8.0, 1.6 Hz, 0.1H, H-5 Fru), 4.09 (dd, J = 18.5, 5.0 Hz, 1H, CH_2 Gly), 4.02 (dd, J = 18.5, 4.9 Hz, 1H, CH_2 Gly) 3.96 (dd, *J* = 18.5, 4.6 Hz, 0.1H, H-6 Fru), 3.92 (dd, *J* = 13.0, 1.9 Hz, 0.9H, H-6 Fru), 3.83 (d, *J* = 13.0 Hz, 1H, H-6 Fru), 3.73 (s, 0.3H, CH_3 Gly), 3.72 (s, 2.7H, CH_3 Gly), 2.19 (s, 0.3H, CH_3 Ac), 2.17 (s, 2.7H CH_3 Ac), 1.51 (s, 3H, CH_3 isop), 1.48 (s, 3H, CH_3 isop), 1.44 (s, 3H, CH_3 isop), 1.31 (s, 3H, CH_3 isop). ^{13}C NMR ($CDCl_3$) δ (170.2, 169.2, 165.7) (CO), (109.9, 109.6) (CH_3 isop), (102.1, 101.5) (C-2 Fru), (75.7, 73.8, 71.6, 70.8, 70.8, 70.4, 70.3) (C-1,3,4,5 Fru), (62.0, 61.8) (C-6 Fru), (52.6, 52.4) (CH_3 Gly), (41.6, 41.3) (CH_2 Gly), (26.7, 26.7, 26.2, 26.0, 25.5, 25.2, 24.3, 24.2) (CH_3 isop), (21.1, 20.9) (CH_3 Ac). HRMS (MALDI-TOF/TOF) *m/z*: calcd. for $C_{18}H_{27}NO_{10}Na$ $[M+Na]^+$ 440.1533; found 440.1528.

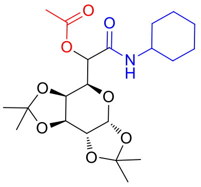

2-(cyclohexylamino)-2-oxo-1-((3aR,5S,5aS,8aS,8bR)-2,2,7,7-tetramethyltetrahydro-3aH-bis([1,3]dioxolo)[4,5-b:4',5'-d]pyran-5-yl)ethyl acetate (2a) Yield 81 % (35 mg); white solid mp = 111-112 °C ; R_f (DS1) = 0.52, R_f (DS2) = 0.47 (EtOAc/PE 1:1, v/v); *d.r.* 80:20. Chemical shifts are given for major isomer. ^{1}H NMR ($CDCl_3$): δ 6.18 (d, *J* = 7.9 Hz, 1H, NH), 5.49 (d, *J* = 4.8 Hz, 1H, H-1 Gal), 4.95 (d, *J* = 9.7 Hz, 1H, H-6 Gal), 4.60 (dd, *J* = 7.9, 2.6 Hz, 1H, H-3 Gal), 4.31 (dd, *J* = 4.8, 2.6 Hz, 1H, H-2 Gal), 4.28 (dd, *J* = 8.0, 1.8 Hz, 1H, H-4 Gal), 4.17 (dd, *J* = 9.7, 1.7 Hz, 1H, H-5 Gal), 3.84 – 3.70 (m, 1H, CH-1 CyHex), 2.13 (s, 3H, CH_3 Ac), 1.88 (m, 2H, CyHex), 1.67 (m, 2H, CyHex), 1.56 (m, 1H, CyHex), 1.50 (s, 3H, CH_3 isop), 1.41 (s, 3H, CH_3 isop), 1.36 – 1.32 (m, 2H, CyHex), 1.31 (s, 3H, CH_3 isop), 1.30 (s, 3H, CH_3 isop), 1.16 (d, *J* = 11.9 Hz, 3H, CyHex). ^{13}C NMR ($CDCl_3$): δ (169.9, 167.0) (CO), (109.8,

109.7) (C isop), 96.4 (C-1 Gal), (71.0, 70.7, 70.6, 70.6, 67.2) (C-2,3,4,5,6 Gal), 48.6 (CH CyHex), (33.04, 32.99) (CyHex), (26.29, 26.17) (CH_3 isop), 25.78 (CyHex), 25.23 (CH_3 isop), (24.95, 24.91) (CyHex), 24.63 (CH_3 isop), 20.95 (CH_3 Ac). HRMS (MALDI-TOF/TOF) *m/z*: calcd. for $C_{21}H_{33}NO_8$ $[M+H]^+$ 428.2284; found 428.2291.

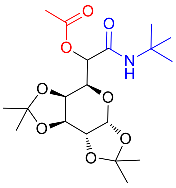

2-(tert-butylamino)-2-oxo-1-((3aR,5S,5aS,8aS,8bR)-2,2,7,7-tetramethyltetrahydro-3aH-bis([1,3]dioxolo)[4,5-b:4',5'-d]pyran-5-yl)ethyl acetate (2b) Yield 78 % (31 mg); colorless oil; R_f (DS1) = 0.58, R_f (DS2) = 0.60 (EtOAc/PE 1:1, v/v); *d.r.* 80:20. Chemical shifts are given for both isomers. ^1H NMR ($CDCl_3$) δ 6.19 (s, 0.8H, NH), 6.09 (s, 0.2H, NH), 5.49 (d, *J* = 4.8 Hz, 1H, H-1 Gal), 5.14 (d, *J* = 6.5 Hz, 0.2H, H-6 Gal), 4.87 (d, *J* = 9.8 Hz, 0.8H, H-6 Gal), 4.61 – 4.55 (m, 1H, H-3 Gal), 4.32 – 4.23 (m, 2H, H-2,4 Gal), 4.18 (d, *J* = 6.4 Hz, 0.2H, H-5 Gal), 4.11 (dd, *J* = 9.8, 1.4 Hz, 0.8H, H-5 Gal), 2.13 (s, 3H, CH_3 Ac), 1.49 (s, 3H, CH_3 isop), 1.40 (s, 3H, CH_3 isop), 1.36 – 1.25 (m, 15H, CH_3 isop, CH_3 tBu). ^{13}C NMR ($CDCl_3$) δ (170.5, 169.7, 167.2, 166.3) (CO), (109.8, 109.7, 109.7, 109.3) (C isop), (96.6, 96.4) (C-1 Gal), (73.9, 71.6, 71.1, 71.0, 70.9, 70.6, 70.6, 67.4, 67.4) (C-2,3,4,5,6 Gal), 51.7 (C tBu), (28.9, 28.7) (CH_3 tBu), (26.3, 26.2, 26.2, 26.1, 25.2, 24.6, 24.3) (CH_3 isop), (21.2, 20.9) (CH_3 OAc). HRMS (MALDI-TOF/TOF) *m/z*: calcd. for $C_{19}H_{31}NO_8$ $[M+H]^+$ 402.2128; found 402.2146.

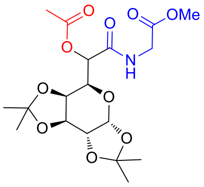

methyl 2-(2-acetoxy-2-((3aR,5S,5aS,8aS,8bR)-2,2,7,7-tetramethyltetrahydro-3aH-bis([1,3]dioxolo)[4,5-b:4',5'-d]pyran-5-yl)acetamido)acetate (2c) Yield 78 % (32 mg); colorless oil; R_f (DS1, DS2) = 0.45 (EtOAc/PE 1:1, v/v); *d.r.* 70:30. Chemical shifts are given for both isomers. ^1H NMR ($CDCl_3$) δ 6.88 (s, 0.3H, NH), 6.79 (s, 0.7H, NH), 5.50 (d, *J* = 4.7 Hz, 1H, H-1 Gal), 5.37 (d, *J* = 6.8 Hz, 0.3H, H-6 Gal), 5.08 (d, *J* = 9.5 Hz, 0.7H, H-6 Gal), 4.61 (m, 1H, H-3 Gal), 4.41 (d, *J* = 8.0 Hz, 0.3H, H-4 Gal), 4.32 – 4.26 (m, 2H, H-4 Gal, H-2 Gal, H-5 Gal), 4.20 (dd, *J* = 16.5, 8.4 Hz, 1H, H-5 Gal, CH_2 Gly), 4.04 (m, 1.4H, CH_2 Gly), 3.91 (dd, *J* = 18.3, 4.3 Hz, 0.3H, CH_2 Gly), 3.72 (s, 3H, CH_3 Gly), 2.15 (s, 0.9H, CH_3 Ac), 2.12 (s, 2.1H, CH_3 Ac), 1.53 (s, 0.9H, CH_3 isop), 1.51 (s, 2.1H, CH_3 isop), 1.44 (s, 0.9H, CH_3 isop), 1.42 (s, 2.1H, CH_3 isop), 1.31 (s, 6H, CH_3 isop). ^{13}C NMR ($CDCl_3$) δ (170.5, 170.0, 169.9, 169.8, 168.3, 167.6) (CO), (110.0, 109.8, 109.6, 109.3) (C isop), (96.5, 96.4) (C-1 Gal), (72.9, 71.3, 71.1, 70.91, 70.8, 70.7, 70.7, 70.5, 67.2, 67.0) (C-2,3,4,5,6 Gal), (52.5, 52.5) (CH_3 Gly), (41.6, 41.4) (CH_2 Gly)), (26.3, 26.2, 26.1, 26.1, 25.2, 25.2, 24.7, 24.4) (CH_3 isop), (21.1, 20.8) (CH_3 Ac). HRMS (MALDI-TOF/TOF) *m/z*: calcd. for $C_{18}H_{27}NO_{10}$ $[M+H]^+$ 418.1713; found 418.1711.

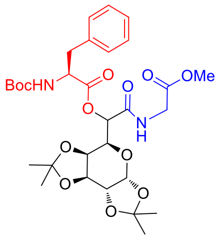

2-((2-methoxy-2-oxoethyl)amino)-2-oxo-1-((3aR,5S,5aS,8aS,8bR)-2,2,7,7-tetramethyltetrahydro-3aH-bis([1,3]dioxolo)[4,5-b:4',5'-d]pyran-5-yl)ethyl 2-((tert-butoxycarbonyl)amino)-3-phenylpropanoate (2d) Yield 80 % (49 mg); colorless oil; R_f (DS1,2) = 0.47 (EtOAc/PE 1:1, v/v); *d.r.* 70:30. ^{1}H NMR ($CDCl_3$) δ 7.27 – 6.92 (m, 6H, Ph, NH), 5.50 (d, *J* = 5.0 Hz, 1.3H, H-1,6 Gal), 5.19 (d, *J* = 8.9 Hz, 0.7H, H-6 Gal), 4.97 (t, *J* = 8.0 Hz, 1H, NH), 4.67 – 4.45 (m, 2H, H-3 Gal, α-Phe), 4.30 (dd, *J* = 6.8, 4.4 Hz, 1.3H, H-2,4 Gal), 4.25 – 4.09 (m, 1.7H, H-5, H-4 Gal), 4.07 – 3.87 (m, 2H, CH_2 Gly), 3.72 (d, *J* = 4.6 Hz, 3H, CH_3 Gly), 3.22 (dd, *J* = 14.0, 6.2 Hz, 1H, β-Phe), 3.08 (dd, *J* = 14.8, 6.5 Hz, 1H, β-Phe), 1.54 (d, *J* = 3.8 Hz, 3H, CH_3 isop), 1.46 (s, 1H, CH_3 isop), 1.37 (bs, 11H, CH_3 Boc, CH_3 isop), 1.30 (dd, *J* = 8.0, 3.5 Hz, 6H, CH_3 isop). ^{13}C NMR ($CDCl_3$) δ (171.5, 170.8, 169.8, 169.8, 167.8, 167.3) (CO), (155.5, 155.4) (CO Boc), (136.5, 136.4) (C Ph), (129.8, 129.7, 128.7, 128.6, 127.1, 126.9) (CH Ph), (109.9, 109.8, 109.6, 109.3) (C isop), (96.4, 96.4) (C-1), (80.3, 80.1) (C Boc), (73.3, 71.6, 71.43, 71.03, 70.9, 70.6, 70.3) (C-2,3,4,6), (67.2, 67.0) (C-5 Gal), (54.9, 54.5) (α-Phe), 52.5 (CH_3 Gly), (41.5, 41.3) (CH_2 Gly), (38.3, 37.5) (β-Phe), 28.5 (CH_3 Boc), (26.3, 26.2, 26.2, 26.0, 25.2, 24.5, 24.5) (CH_3 isop). HRMS (MALDI-TOF/TOF) *m/z*: calcd. for $C_{30}H_{42}N_2O_{12}Na$ $[M+Na]^+$ 645.2635; found 645.2615.

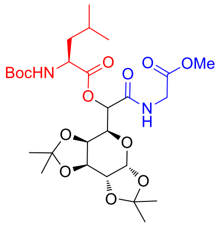

2-((2-methoxy-2-oxoethyl)amino)-2-oxo-1-((3aR,5S,5aS,8aS,8bR)-2,2,7,7-tetramethyltetrahydro-3aH-bis([1,3]dioxolo)[4,5-b:4',5'-d]pyran-5-yl)ethyl 2-((tert-butoxycarbonyl)amino)-4-methylpentanoate (2e) Yield 58 % (34 mg); colorless oil; R_f (DS1,2) = 0.55, (EtOAc/PE 1:1, v/v); *d.r.* 75:25. ^{1}H NMR ($CDCl_3$) δ 7.33 (s, 0.25H, NH), 6.93 (s, 0.75H, NH), 5.50 (0.25, H-6 Gal), 5.50 (d, *J* = 4.8 Hz, 0.75H, H-1 Gal), 5.44 (d, *J* = 4.8 Hz, 0.25H, H-1 Gal), 5.19 (d, *J* = 8.9 Hz, 0.75H, H-6 Gal), 4.90 (dd, *J* = 16.4, 7.7 Hz, 1H, NH Leu), 4.63 – 4.60 (m, 0.75H, H-3 Gal), 4.57 (dd, *J* = 7.9, 2.1 Hz, 0.25H, H-3 Gal), 4.50 (d, *J* = 7.6 Hz, 0.25H, α-Leu), 4.36 (m, 0.75H, α-Leu), 4.33 – 4.16 (m, 3H, H-2,4,5 Gal), 4.02 (ddd, *J* = 41.5, 18.2, 5.3 Hz, 2H, CH_2 Gly), 3.70 (d, *J* = 6.9 Hz, 3H, CH_3 Gly), 1.74 (s, 3H, β-Leu, γ-Leu), 1.51 (d, *J* = 7.5 Hz, 3H, CH_3 isop), 1.45 – 1.37 (m, 15H, CH_3 Boc, CH_3 isop), 1.31 – 1.27 (m, 3H, CH_3 isop), 0.94 – 0.91 (m, 6H, CH_3 Leu). ^{13}C NMR (151 MHz, $CDCl_3$) δ (172.8, 172.0, 169.9, 169.8, 167.8, 167.5) (CO), (156.0, 155.7) (CO Boc), (109.9, 109.6, 109.1) (C isop), 96.4 (C-1 Gal), (80.3, 80.0) (C Boc), (72.7, 71.6, 71.0, 71.0, 70.6, 70.4) (C-2,3,4,6 Gal), (67.4, 67.0) (C-5 Gal), (52.9, 52.4, 52.3) (CH_3 Gly, α-Leu), (41.8, 41.4, 41.3, 41.0) (CH_2 Gly, β-Leu), 29.9, (28.5, 28.5) (Boc), (26.3, 26.2, 26.1) (CH_3 isop), (25.3, 25.2) (γ-Leu), (24.9, 24.8, 24.6, 23.1, 23.0) (CH_3 isop), (22.3, 22.0) (δ-Leu). HRMS (MALDI-TOF/TOF) *m/z*: calcd. for $C_{27}H_{44}N_2O_{12}Na$ $[M+Na]^+$ 611.2792; found 611.2805.

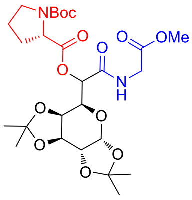

1-tert-butyl 2-(2-((2-methoxy-2-oxoethyl)amino)-2-oxo-1-((3aR,5S,5aS,8aS,8bR)-2,2,7,7-tetramethyltetrahydro-3aH-bis([1,3]dioxolo)[4,5-b:4',5'-d]pyran-5-yl)ethyl) pyrrolidine-1,2-dicarboxylate (2f) Yield 67 % (40 mg); colorless oil; R_f (DS1,2) = 0.35, (EtOAc/PE 1:1, v/v); *d.r.* 55:45. ^{1}H NMR ($CDCl_3$) δ 7.38 (dd, *J* = 19.5, 14.3 Hz, 0.45H, NH), 6.74 (t, *J* = 4.7 Hz, 0.55H, NH), 5.49 (d, *J* = 4.8 Hz, 1H, H-1 Gal), 5.28 (d, *J* = 8.0 Hz, 0.55H, H-6 Gal), 5.05 (d, *J* = 9.7 Hz, 0.45H, H-6 Gal), 4.60 (ddd, *J* = 25.8, 7.9, 2.3 Hz, 1H, H-3 Gal), 4.38 – 4.23 (m, 4H, H-2, H-4, H-5 Gal, CH_2 Gly), 4.10 (dd, *J* = 17.9, 6.0 Hz, 0.55H, CH_2 Gly), 4.02 (t, *J* = 5.7 Hz, 1H, α-Pro), 3.88 (dd, *J* = 17.9, 5.3 Hz, 0.45H, CH_2 Gly), 3.71 (s, 1.6H, CH_3 Gly), 3.68 (s, 1.4H, CH_3 Gly), 3.55 – 3.46 (m, 1H, δ-Pro), 3.42 – 3.30 (m, 1H, δ-Pro), 2.33 – 2.26 (m, 0.5H, β-Pro), 2.22 – 2.13 (m, 1.5H, β-Pro), 1.88 (ddd, *J* = 25.6, 11.5, 8.4 Hz, 2H, γ-Pro), 1.36 (ddd, *J* = 36.5, 29.4, 26.3 Hz, 21H, CH_3 Boc, CH_3 isop). ^{13}C NMR ($CDCl_3$) δ (171.8, 171.5, 169.9, 168.3, 167.8) (CO), (155.3, 154.1) (CO Boc), (109.8, 109.7, 109.6, 109.3) (C isop), (96.5, 96.4) (C-1 Gal), (80.2, 80.1) (C Boc), (71.9, 71.2, 70.9, 70.8, 70.7, 70.5, 70.4) (C-2,3,4,6 Gal), (67.1, 66.9) (C-5 Gal), (59.2, 58.9) (α-Pro), (52.4, 52.3) (CH_3 Gly), (47.0, 46.5) (δ-Pro), (41.5, 41.2) (CH_2 Gly), (30.9, 30.2) (β-Pro), (28.7, 28.5) (CH_3 Boc), (26.3, 26.2, 26.2, 25.2, 25.2, 24.7, 24.4) (CH_3 isop), (24.4, 23.4) (γ-Pro). HRMS (MALDI-TOF/TOF) *m/z*: calcd. for $C_{26}H_{40}N_2O_{12}$ $[M+H]^+$ 595.2479; found 595.2453.

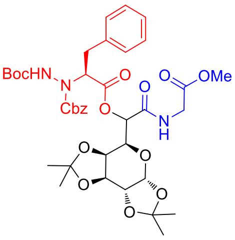

1-benzyl 2-tert-butyl 1-(1-(2-((2-methoxy-2-oxoethyl)amino)-2-oxo-1-((3aR,5S,5aS,8aS,8bR)-2,2,7,7-tetramethyltetrahydro-3aH-bis([1,3]dioxolo)[4,5-b:4',5'-d]pyran-5-yl)ethoxy)-1-oxo-3-phenylpropan-2-yl)hydrazine-1,2-dicarboxylate (2g) Yield 74 % (57 mg); colorless oil; R_f (DS1,2) = 0.44 (EtOAc/PE 1:1, v/v); *d.r.* 70:30. ^{1}H NMR ($CDCl_3$) δ 7.27 (d, *J* = 16.6 Hz, 11H, CH Ph, NH), 5.50 (d, *J* = 4.8 Hz, 1H, H-1 Gal), 5.25 – 5.16 (m, 4H, H-6 Gal, CH_2 Cbz, NH), 5.07 (m, 1H, α-hPhe), 4.55 (d, *J* = 10.2 Hz, 1H, H-3 Gal), 4.46 (dd, *J* = 8.0, 1.3 Hz, 1H, H-4 Gal), 4.30 (dd, *J* = 4.7, 2.5 Hz, 1H, H-2 Gal), 4.15 – 3.98 (m, 3H, H-5 Gal, CH_2 Gly), 3.72 (s, 1.8H, CH_3 Gly), 3.70 (s, 1.2H, CH_3 Gly), 3.31 (dd, *J* = 12.8, 6.8 Hz, 1H, β-hPhe), 3.24 – 3.07 (m, 1H, β-hPhe), 1.54 – 1.12 (m, 21H, CH_3 Boc, CH_3 isop). ^{13}C NMR ($CDCl_3$) δ (169.8, 169.1, 168.5, 167.6) (CO), 155.3 (CO Boc), (137.9, 136.0) (C Ph), (129.6, 128.5, 128.4, 128.2, 127.9, 126.6) (CH Ph), (110.1, 109.8, 109.6, 109.6) (C isop), (96.4, 96.3) (C-1 Gal), (81.2, 81.2) (C Boc), (70.9, 70.8, 70.8, 70.4, 70.2) (H-2,3,4,6 Gal), (68.5, 68.3) (CH_2 Cbz), (67.7, 67.1) (C-5 Gal), (62.7, 62.2) (α-Phe), 52.5 (CH_3 Gly), (41.5, 41.2) (CH_2 Gly), (34.6, 34.3) (β-Phe), (28.3, 28.1) (CH_3 Boc), (26.2, 26.1, 26.0, 25.9, 25.1, 24.4, 24.4, 24.2) (CH_3 isop). HRMS (MALDI-TOF/TOF) *m/z*: calcd. for $C_{38}H_{49}N_3O_{12}Na$ $[M+Na]^+$ 794.3112; found 794.3111.

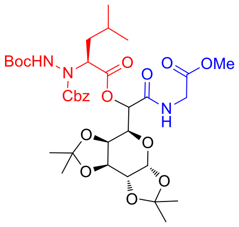

1-benzyl 2-tert-butyl 1-(1-(2-((2-methoxy-2-oxoethyl)amino)-2-oxo-1-((3aR,5S,5aS,8aS,8bR)-2,2,7,7-tetramethyltetrahydro-3aH-bis([1,3]dioxolo)[4,5-b:4',5'-d]pyran-5-yl)ethoxy)-4-methyl-1-oxopentan-2-yl)hydrazine-1,2-dicarboxylate (2h) Yield 53 % (39 mg); colorless oil; R_f (DS1,2) = 0.54 (EtOAc/PE 1:1, v/v); *d.r.* 70:30. ^{1}H NMR ($CDCl_3$) δ 7.31 (m, 5H, Ph Cbz), 6.91 (s, H, NH), 6.72 (s, H, NH), 5.49 (dd, *J* = 13.6, 4.7 Hz, 1H, H-1 Gal), 5.15 (ddd, *J* = 33.8, 24.8, 12.0 Hz, 4H, H-6 Gal, CH_2 Cbz, NH), 4.79 (t, *J* = 10.2 Hz, 1H, α-hLeu), 4.63 – 4.48 (m, 1H, H-3 Gal), 4.39 – 3.86 (m, 5H, H-2,4,5 Gal, CH_2 Gly), 3.72 (d, *J* = 3.1 Hz, 3H, CH_3 Gly), 1.94 (m, 1H, β-hLeu), 1.63 (m, 1H, β-hLeu), 1.56 – 1.19 (m, 21H, CH_3 Boc, CH_3 isop), 1.06 – 0.80 (m, 6H, δ-hLeu). ^{13}C NMR ($CDCl_3$) δ (169.8, 169.7, 167.7) (CO), (156.4, 155.1) (CO Boc), 136.1 (C Cbz), (128.8, 128.8, 128.3) (CH Cbz), (109.9, 109.86, 109.8, 109.8) (C isop), (96.4, 96.3) (C-1 Gal), (81.5, 81.4) (CO Boc), (74.4, 71.6, 71.4, 70.9, 70.6, 70.4, 70.2) (C-2,3,4,6), (68.8, 68.7) (CH_2 Cbz), (67.2, 67.1, 66.9) (C-5 Gal), (60.3, 59.3) (α-Leu), 52.5 (CH_3 Gly), (41.6, 39.9) (CH_2 Gly), (37.0, 36.8) (β-Leu), 28.4 (CH_3 Boc), (26.2, 26.2, 26.1, 26.0) (CH_3 isop), (25.2, 25.2) (γ-Leu), (24.5, 24.4, 24.3, 23.9, 23.5) (CH_3 isop), (21.7, 21.7) (δ-Leu). HRMS (MALDI-TOF/TOF) *m/z*: calcd. for $C_{35}H_{51}N_3O_{14}$ $[M+Na]^+$ 760.3269; found 760.3243.

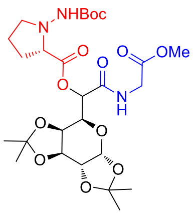

2-((2-methoxy-2-oxoethyl)amino)-2-oxo-1-((3aR,5S,5aS,8aS,8bR)-2,2,7,7-tetramethyltetrahydro-3aH-bis([1,3]dioxolo)[4,5-b:4',5'-d]pyran-5-yl)ethyl 1-((tert-butoxycarbonyl)amino)pyrrolidine-2-carboxylate (2i) Yield 66 % (39 mg); colorless oil; R_f (DS1,2) = 0.38 (EtOAc/PE 2:1, v/v); *d.r.* 70:30. ^{1}H NMR ($CDCl_3$) δ 7.03 (s, 0.3H), 6.95 (s, 0.7H), 6.71 (d, *J* = 13.8 Hz, 1H), 5.52 (d, *J* = 4.8 Hz, 0.7H), 5.49 (d, *J* = 4.9 Hz, 0.3H), 5.37 (d, *J* = 8.0 Hz, 0.3H), 5.19 (d, *J* = 9.3 Hz, 0.7H), 4.61 (dd, *J* = 7.9, 2.4 Hz, 1H), 4.47 (dd, *J* = 8.0, 1.7 Hz, 0.3H), 4.32 (dd, *J* = 4.9, 2.6 Hz, 0.7H), 4.28 (dd, *J* = 7.8, 1.8 Hz, 1H), 4.21 (dd, *J* = 9.3, 1.8 Hz, 0.3H), 4.18 – 4.13 (m, 0.7H), 4.03 (dd, *J* = 7.4, 5.4 Hz, 2H), 3.92 (dd, *J* = 18.3, 4.8 Hz, 0.3H), 3.71 (s, 0.9H), 3.71 (s, 2.1H), 3.29 (td, *J* = 14.2, 7.6 Hz, 1H, δ-Pro), 3.13 – 3.06 (m, 1H, δ-Pro), 2.21 (dq, *J* = 16.4, 8.2 Hz, 1H, β-Pro), 2.13 – 2.03 (m, 1H, β-Pro), 1.93 – 1.84 (m, 2H, γ-Pro), 1.38 (m, 21H, CH_3 Boc, CH_3 isop). ^{13}C NMR ($CDCl_3$) δ (173.0, 172.0, 170.0, 169.9, 168.3, 167.1) (CO), (110.1, 110.0, 109.7, 109.3) (C isop), (96.5, 96.4) (C-1 Gal), (80.2, 80.0) (CO Boc), (72.8, 71.1, 70.9, 70.8, 70.7, 70.6, 70.4) (C-2,3,4,6 Gal), (67.5, 67.2) (C-5 Gal), (64.8, 64.0) (α-Pro), (53.4, 53.2) (δ-Pro), (52.5, 52.4) (CH_3 Gly), (41.5, 41.3) (CH_2 Gly), (28.6, 28.5) (CH_3 Boc), (27.9, 27.6) (β-Pro), (26.2, 26.2, 26.1, 25.2, 25.1, 24.6, 24.5) (CH_3 isop), (22.3, 22.2) (γ-Pro). HRMS (MALDI-TOF/TOF) *m/z*: calcd. for $C_{26}H_{41}N_3O_{12}Na$ $[M+Na]^+$ 610.2588; found 610.2571.

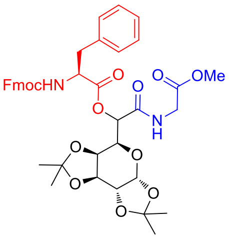

2-((2-methoxy-2-oxoethyl)amino)-2-oxo-1-((3aR,5S,5aS,8aS,8bR)-2,2,7,7-tetramethyltetrahydro-3aH-bis([1,3]dioxolo)[4,5-b:4',5'-d]pyran-5-yl)ethyl 2-((((9H-fluoren-9-yl)methoxy)carbonyl)amino)-3-phenylpropanoate (2k) Yield 74% (55 mg); colorless oil; R_f (DS1,2) = 0.39, (EtOAc/PE 1:1, v/v); *d.r.* 70:30. ^{1}H NMR (CDCl$_3$) δ 7.73 (d, *J* = 7.5 Hz, 2H, Fmoc), 7.54 – 7.49 (m, 2H, Fmoc), 7.37 (t, *J* = 7.4 Hz, 2H, Fmoc), 7.30 – 7.19 (m, 7H, Ph), 6.96 (s, 0.3H, NH), 6.87 (s, 0.7H, NH), 5.52 (d, *J* = 4.7 Hz, 0.3H, H-1 Gal), 5.50 (d, *J* = 4.8 Hz, 0.7H, H-1 Gal), 5.44 (d, *J* = 7.6 Hz, 0.3H, H-6 Gal), 5.31 (d, *J* = 8.3 Hz, 0.7H, NH), 5.29 – 5.24 (m, 0.3H, NH), 5.17 (d, *J* = 9.2 Hz, 0.7H, H-6 Gal), 4.78 – 4.69 (m, 1H, α-Phe), 4.58 (ddd, *J* = 32.1, 7.8, 2.2 Hz, 1H, H-3 Gal), 4.38 (dt, *J* = 18.2, 9.1 Hz, 1H, CH$_2$ Fmoc), 4.30 (dd, *J* = 4.9, 2.6 Hz, 1H, H-2 Gal), 4.27 – 4.00 (m, 6H, CH$_2$ Gly, CH Fmoc, CH$_2$ Fmoc, H-5,4 Gal), 3.72 (s, 2.1H, CH$_3$ Gly), 3.68 (s, 0.9H, CH$_3$ Gly), 3.26 (dd, *J* = 14.0, 6.2 Hz, 1H, β-Phe), 3.13 (dd, *J* = 14.0, 6.4 Hz, 1H, β-Phe), 1.55 – 1.21 (m, 12H, CH$_3$ isop). ^{13}C NMR (CDCl$_3$) δ (171.1, 170.5, 169.8, 167.7) (CO Fmoc), (155.9, 155.8) (CO Fmoc), (144.0, 143.9, 141.5) (C Fmoc), (136.4, 136.1) (C Ph), (129.8, 129.8, 128.8, 128.6, 127.9, 127.2, 127.1) (CH Ph), (125.3, 125.3, 125.2) (CH Fmoc-Ph), (120.2, 120.1) (CH Fmoc-Ph), (110.0, 109.9, 109.6, 109.3) (C isop), (96.4, 96.4) (C-1 Gal), (73.5, 71.5, 71.3, 71.0, 70.9, 70.8, 70.5, 70.3) (C-2,3,4,6 Gal), 67.4 (C-5 Gal), 67.2 (CH$_2$ Fmoc), 67.0 (C-5 Gal), (55.1, 54.9) (α-Phe), (52.5, 52.4) (CH$_3$ Gly), (47.4, 47.3) (CH Fmoc), (41.5, 41.3) (CH$_2$ Gly), (38.3, 37.6) (β-Phe), (26.3, 26.2, 26.2, 26.0, 25.2, 24.5, 24.4) (CH$_3$ isop). HRMS (MALDI-TOF/TOF) *m/z*: calcd. for $C_{40}H_{44}N_2O_{12}$ $[M+H]^+$ 745.2972; found 745.2990.

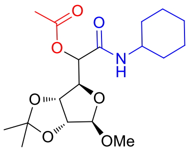

2-(cyclohexylamino)-1-((3aR,4S,6R,6aR)-6-methoxy-2,2-dimethyltetrahydrofuro[3,4-d][1,3]dioxol-4-yl)-2-oxoethyl acetate (3a) Yield 82% (30 mg); colorless oil; R_f = 0.63 (EtOAc/PE 1:1, v/v); *d.r.* 77:23. Chemical shifts are given for both isomers. ^{1}H NMR (CDCl$_3$): δ 5.97 (d, *J* = 7.5 Hz, 0.77H, NH), 5.90 (d, *J* = 7.7 Hz, 0.23H, NH), 5.11 (d, *J* = 7.6 Hz, 0.23H, H-5 Rib), 5.07 (d, *J* = 6.9 Hz, 0.77H, H-5 Rib), 4.95 (s, 0.23H, H-1 Rib), 4.94 (s, 0.77H, H-1 Rib), 4.87 (dd, *J* = 6.0, 1.4 Hz, 0.23H, H-2 Rib), 4.71 (dd, *J* = 6.2, 0.5 Hz, 0.77H, H-2 Rib), 4.60 (d, *J* = 6.9 Hz, 0.77H, H-4 Rib), 4.55 (d, *J* = 6.0 Hz, 0.77H, H-3 Rib), 4.53 (d, *J* = 6.0 Hz, 0.23H, H-3 Rib), 4.50 (dd, *J* = 7.6, 1.4 Hz, 0.23H, H-4 Rib), 3.79 – 3.72 (m, 1H, CH-1 CyHex), 3.32 (s, 2.3H, OCH$_3$), 3.28 (s, 0.7H, OCH$_3$), 2.15 (s, 0.7H, CH$_3$ Ac), 2.13 (s, 2.3H, CH$_3$ Ac), 1.89 (m, 2H, CyHex), 1.66 (m, 2H, CyHex), 1.58 (m, 1H, CyHex), 1.45 (s, 3H, CH$_3$ isop), 1.34 (m, 2H, CyHex), 1.29 (s, 2.3H, CH$_3$ isop), 1.28 (s, 0.7H, CH$_3$ isop), 1.16 – 1.14 (m, 3H, CyHex). ^{13}C NMR (CDCl$_3$) δ (170.0, 166.1) (CO), 112.9 (C isop), (111.1, 110.3) (C-1 Rib), (86.2, 86.0) (C-4 Rib), (85.4, 85.3) (C-3 Rib), (81.7, 81.4) (C-2 Rib), (74.8, 73.6) (C-5 Rib), (56.2, 55.5) (OCH$_3$), 48.5 (CH CyHex), 48.35 (CH CyHex), (33.1, 33.1) (CyHex), (26.8, 26.7) (CH$_3$ isop), (25.7, 25.6, 25.3, 24.9, 24.9) (CyHex), (21.4, 21.1) (CH$_3$ Ac). HRMS (MALDI-TOF/TOF) *m/z*: calcd. for $C_{18}H_{29}NO_7Na$ $[M+Na]^+$ 394.1842; found 394.1843.

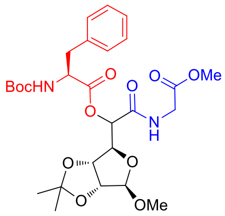

1-((3aR,4S,6R,6aR)-6-methoxy-2,2-dimethyltetrahydrofuro[3,4-d][1,3]dioxol-4-yl)-2-((2-methoxy-2-oxoethyl)amino)-2-oxoethyl 2-((tert-butoxycarbonyl)amino)-3-phenylpropanoate (3d) Yield 78 % (44 mg); colorless oil; R_f (DS1) = 0.53, (EtOAc/PE 1:1, v/v); *d.r.* 77:23. ^{1}H NMR ($CDCl_3$) δ 7.36 – 7.08 (m, 5H, CH Ph), 7.15 (s, 1H, NH), 5.36 (d, *J* = 4.6 Hz, 1H, NH), 5.19 (d, *J* = 4.8 Hz, 0.23H, H-5 Rib), 5.04 (d, *J* = 7.6 Hz, 0.77H, H-5 Rib), 4.96 (s, 0.77H, H-1 Rib), 4.94 (s, 0.23H, H-1 Rib), 4.74 - 4.53 (m, 4H, H-2,3,4 Rib, α-Phe), 4.09 (d, *J* = 5.8 Hz, 0.23H, CH_2 Gly), 4.03 (d, *J* = 5.8 Hz, 0.77H, CH_2 Gly), 3.94 (d, *J* = 5.4 Hz, 0.77H, CH_2 Gly), 3.88 (d, *J* = 5.4 Hz, 0.23H, CH_2 Gly), 3.71 (d, *J* = 5.2 Hz, 3H, CH_3 Gly), 3.33 – 3.30 (m, 3.77H, CH_3 Rib, β-Phe), 2.98 (dd, *J* = 14.3, 8.8 Hz, 1.23H, β-Phe), 1.46 (s, 2.31H, CH_3 isop), 1.42 (s, 0.69H, CH_3 isop), 1.39 (s, 2.31H, Boc), 1.37 (s, 6.9H, Boc), 1.31 (s, 2.1H, CH_3 isop), 1.27 (s, 0.69H, CH_3 isop). ^{13}C NMR ($CDCl_3$) δ (170.5, 169.6, 167.2) (CO), 156.2 (CO Boc), 136.4 (C Ph), (129.6, 129.3, 129.0, 128.9, 127.6, 127.3) (CH Ph), 112.8 (C isop), (111.9, 109.9) (C-1 Rib), (86.4, 86.2) (C-4 Rib), (85.7, 85.6) (C-3 Rib), (81.2, 81.0) (C-2 Rib), (80.9, 80.7) (C Boc), (75.0, 74.5) (C-5 Rib), (56.3, 55.9) (α-Phe), (55.3, 54.9) (CH_3 Rib), (52.5, 52.3) (CH_3 Gly), (41.2, 41.1) (CH_2 Gly), (37.6, 37.6) (β-Phe), (28.5, 28.4) (CH_3 Boc), (26.9, 26.7) (CH_3 isop), (25.7, 25.3) CH_3 isop). HRMS (MALDI-TOF/TOF) *m/z*: calcd. for $C_{27}H_{38}N_2O_{11}Na$ $[M+Na]^+$ 589.2373; found 589.2399.

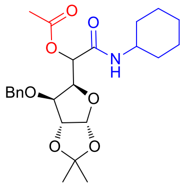

1-((3aR,5S,6S,6aR)-6-(benzyloxy)-2,2-dimethyltetrahydrofuro[2,3-d][1,3]dioxol-5-yl)-2-(cyclohexylamino)-2-oxoethyl acetate (4a) Yield 79% (36 mg); colorless oil; R_f (DS1) = 0.40, R_f (DS2) = 0.26 (EtOAc/PE 1:2, v/v); *d.r.* 77:23. Chemical shifts are given for major isomer. ^{1}H NMR ($CDCl_3$): δ 7.41 – 7.14 (m, 5H, Ph), 6.01 (d, *J* = 7.9 Hz, 1H, NH), 5.92 (d, *J* = 3.7 Hz, 1H, H-1 Xyl), 5.11 (d, *J* = 9.2 Hz, 1H, H-5 Xyl), 4.62 (d, *J* = 11.6 Hz, 1H, CH_{2a}-Ph), 4.60 (d, *J* = 3.7 Hz, 1H, H-2 Xyl), 4.45 – 4.38 (m, 2H, H-4 Xyl, CH_{2b}-Ph), 3.99 (d, *J* = 3.1 Hz, 1H, H-3 Xyl), 3.79 – 3.70 (m, 1H, CH-1 CyHex), 1.98 (s, 3H, CH_3 Ac), 1.84 (m, 2H, CyHex), 1.63 (m, 2H), 1.55 – 1.50 (m, 1H, CyHex), 1.45 (s, 3H, CH_3 isop), 1.30 (bs, 5H, CH_3 isop, CyHex), 1.16 – 1.06 (m, 3H, CyHex). ^{13}C NMR ($CDCl_3$) δ (169.70, 166.78) (CO), 136.97 (C Ph), (128.84, 128.45, 128.35) (CH Ph), 112.61 (C isop), 105.65 (C-1 Xyl), (81.79, 81.30, 78.64, 72.47, 70.22) (C-2,3,4,5-Xyl, CH_2-Ph), 48.52 (CH CyHex), (32.89, 32.83) (CyHex), (27.01, 26.58) (CH_3 isop), (25.73, 24.79, 24.75) (CyHex), 20.78 (CH_3 Ac). HRMS (MALDI-TOF/TOF) *m/z*: calcd. for $C_{24}H_{33}NO_7$ $[M+H]^+$ 448.2335; found 448.2346.

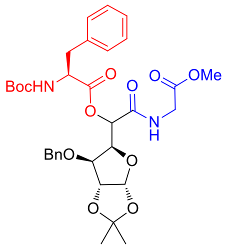

1-((3aR,5S,6S,6aR)-6-(benzyloxy)-2,2-dimethyltetrahydrofuro[2,3-d][1,3]dioxol-5-yl)-2-((2-methoxy-2-oxoethyl)amino)-2-oxoethyl 2-((tert-butoxycarbonyl)amino)-3-phenylpropanoate (4d) Yield 75 % (48 mg); colorless oil; R_f (DS1) = 0.52, (EtOAc/PE 1:1, v/v); *d.r.* 77:23. ^{1}H NMR ($CDCl_3$) δ 7.42 – 7.11 (m, 10H, Bn, Ph), 6.86 (s, 1H, NH), 5.94 (d, *J* = 3.6 Hz, 1H, H-1 Xyl), 5.65 (d, *J* = 7.2 Hz, 0.23H, H-5 Xyl), 5.43 (d, *J* = 9.0 Hz, 0.77H, H-5 Xyl), 4.91 (m, 1H, α-Phe), 4.67 – 4.39 (m, 5.77H, H-2, H-4, H-3 Xyl, NH, CH_2 OBn), 4.24 (d, *J* = 3.9 Hz, 0.23H, H-3 Xyl), 4.02 (d, *J* = 5.3 Hz, 0.77H, CH_2 Gly), 3.99 (m, 0.77H, CH_2 Gly), 3.92 (d, *J* = 5.7 Hz, 0.46H, CH_2 Gly), 3.71 (s, 2.31H, CH_3 Gly), 3.69 (s, 0.69H, CH_3 Gly), 3.28 (dd, *J* = 14.1, 5.6 Hz, 0.77H, β-Phe), 3.12 (dd, *J* = 14.2, 5.3 Hz, 0.23H, β-Phe), 3.00 (dd, *J* = 14.1, 7.7 Hz, 0.77H, β-Phe), 2.88 (dd, *J* = 14.1, 8.3 Hz, 0.23H, β-Phe), 1.48 (s, 3H, CH_3 isop), 1.35 (s, 3H, CH_3 isop), 1.30 (s, 9H, CH_3 Boc). ^{13}C NMR ($CDCl_3$) δ (171.0, 169.8, 167.7) (CO), 155.4 (CO Boc), (137.1, 136.5, 136.1) (C Ph), (129.7, 129.5, 128.8, 128.7, 128.6, 128.4, 128.3, 128.2, 127.2, 127.0) (CH Ph), (112.8, 112.4) (C isop), (105.8, 105.2) (C-1 Xyl), (83.0, 83.0) (C-2 Xyl), (81.9, 81.4) (C-3 Xyl), (80.6, 80.0) (C Boc), (79.0, 78.5) (C-4 Xyl), (72.8, 72.6) (CH_2-OBn), (72.1, 70.0) (C-5 Xyl), (55.0, 54.3) (α-Phe), (52.5, 52.4) (CH_3 Gly), (41.5, 41.1) (CH_2 Gly), (37.9, 37.1) (β-Phe), 28.3 (CH_3 Boc), (28.35, 27.2, 27.0, 26.6, 26.5) (CH_3 isop). HRMS (MALDI-TOF/TOF) *m/z*: calcd. for $C_{33}H_{42}N_2O_{11}Na$ $[M+Na]^+$ 665.2686; found 665.2682.

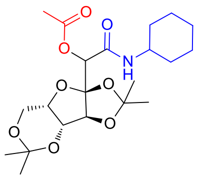

2-(cyclohexylamino)-2-oxo-1-((3aS,3bR,7aS,8aR)-2,2,5,5-tetramethyltetrahydro-3aH-[1,3]dioxolo[4',5':4,5]furo[3,2-d][1,3]dioxin-8a-yl)ethyl acetate (5a) Yield 66 % (28 mg); colorless oil; R_f (DS1) = 0.50, R_f (DS2) = 0.40 (toluen/aceton 5:1, v/v); *d.r.* 90:10. Chemical shifts are given for major isomer. ^{1}H NMR ($CDCl_3$): δ 6.30 (d, *J* = 7.6 Hz, 1H, NH), 5.26 (s, 1H, H-1 Sor), 4.41 (s, 1H, H-5 Sor), 4.31 (d, *J* = 2.2 Hz, 1H, H-4 Sor), 4.17 (d, *J* = 1.5 Hz, 1H, H-3 Sor), 4.07 (dd, *J* = 13.6, 2.2 Hz, 1H, H-6a Sor), 3.98 (d, *J* = 13.5 Hz, 1H, H-6b Sor), 3.85 – 3.75 (m, 1H, CH-1 CyHex), 2.17 (s, 3H, CH_3 Ac), 1.89 (m, 2H, CyHex), 1.63 (s, 3H, CyHex), 1.54 (m, 1H, CyHex), 1.46 (s, 3H, CH_3 isop), 1.41 (d, 6H, CH_3 isop), 1.38 (bs, 3H, CH_3 isop), 1.34 (m, 1H, CyHex), 1.24 – 1.17 (m, 3H, CyHex). ^{13}C NMR ($CDCl_3$): δ (169.6, 164.6) (CO), (113.8, 113.2) (C isop), 97.8 (C-2 Sor), 85.66 (C-5 Sor), 73.7 (C-3 Sor), 72.9 (C-4 Sor), 72.7 (C-1 Sor), 60.6 (C-6 Sor), 48.4 (CH CyHex), 32.9 (CyHex), (32.8, 29.2) (CyHex), (27.8, 26.5, 25.9) (CH_3 isop), (24.7, 24.7) (CyHex), 21.0 (CH_3 isop), 18.9 (CH_3 Ac). HRMS (MALDI-TOF/TOF) *m/z*: calcd. for $C_{21}H_{33}NO_8$ $[M+H]^+$ 428.2284; found 428.2272.

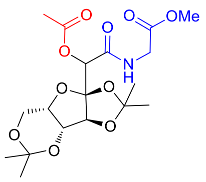

methyl 2-(2-acetoxy-2-((3aS,3bR,7aS,8aR)-2,2,5,5-tetramethyltetrahydro-3aH-[1,3]dioxolo[4',5':4,5]furo[3,2-d][1,3]dioxin-8a-yl)acetamido)acetate (5c) Yield 63% (26 mg); colorless oil; R_f (DS1) = 0.30, R_f (DS2) = 0.27 (EtOAc/PE 1:1, v/v); *d.r.* 87:13. ^{1}H NMR ($CDCl_3$) δ 7.10 (s, 0.87H, NH), 6.84 (s, 0.13H, NH), 5.68 (s, 0.13H, H-1 Sor), 5.39 (s, 0.87H, H-1 Sor), 4.87 (s, 0.13H, H-5 Sor), 4.41 (s, 0.87H, H-5 Sor), 4.33 (d, *J* = 1.7 Hz, 0.87H, H-4 Sor), 4.29 (d, *J* = 2.3 Hz, 0.13H, H-4 Sor), 4.23 – 3.96 (m, 5H, H-3,6 Sor, CH_2 Gly), 3.73 (d, *J* = 1.8 Hz, 3H, CH_3 Gly), 2.22 (s, 0.39H, CH_3 Ac), 2.18 (s, 2.61H, CH_3 Ac), 1.47-1.30 (m, 12H, CH_3 isop).^{13}C NMR ($CDCl_3$) δ (170.2, 169.4, 165.6) (CO), 114.0 (C isop), 112.9 (C isop), 97.8 (C-2 Sor), 85.6 (C-5 Sor), (74.5, 74.0, 73.5, 73.2, 72.8, 72.4) (C-1,3,4 Sor), 60.4 (C-6 Sor), 52.5 (CH_3 Gly), (41.7, 41.3) (CH_2 Gly), (29.1, 28.5, 27.9, 27.7, 26.4) (CH_3 isop), (19.2, 18.8) (CH_3 Ac). HRMS (MALDI-TOF/TOF) *m/z*: calcd. for $C_{18}H_{27}NO_{10}$ $[M+H]^+$ 418,1713; found 418,1697.

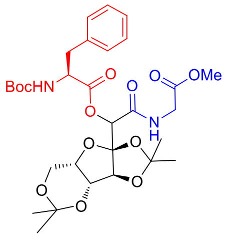

2-((2-methoxy-2-oxoethyl)amino)-2-oxo-1-((3aS,3bR,7aS,8aR)-2,2,5,5-tetramethyltetrahydro-3aH-[1,3]dioxolo[4',5':4,5]furo[3,2-d][1,3]dioxin-8a-yl)ethyl 2-((tert-butoxycarbonyl)amino)-3-phenylpropanoate (5d) Yield 65 % (40 mg); colorless oil; R_f (DS1,2) = 0.34 (EtOAc/PE 1:1, v/v); *d.r.* 90:10. ^{1}H NMR ($CDCl_3$) δ 7.35 – 7.05 (m, 6H, CH Ph, NH), 5.50 (s, 1H, H-1 Sor), 4.94 (d, *J* = 8.4 Hz, 1H, NH Phe), 4.65 (dd, *J* = 12.9, 8.0 Hz, 1H, α-Phe), 4.42 (s, 1H, H-5 Sor), 4.36 (s, 1H, H-4 Sor), 4.25 (s, 1H, H-3 Sor), 4.17 (dd, *J* = 18.5, 4.9 Hz, 1H, CH_2 Gly), 4.14 – 4.10 (m, 2H, H-6 Sor), 4.07 (dd, *J* = 18.5, 4.7 Hz, 1H, CH_2 Gly), 3.76 (s, 3H, CH_3 Gly), 3.42 (dd, *J* = 14.2, 4.7 Hz, 1H, β-Phe), 3.06 (dd, *J* = 14.2, 8.2 Hz, 1H, β-Phe), (1.51, 1.44, 1.40, 1.36) (21H, CH_3 isop, CH_3 Boc). ^{13}C NMR ($CDCl_3$) δ (170.8, 170.1, 165.3) (CO), 155.4 (CO Boc), 136.6 (C Ph), (129.8, 128.7, 126.9) (CH Ph), (114.1, 112.8) (C isop), 97.8 (C-2 Sor), 85.5 (C-5 Sor), 80.0 (C Boc), 74.2 (C-3 Sor), 72.7 (C-4 Sor), 60.5 (C-6 Sor), 54.6 (α-Phe), 52.5 (CH_3 Gly), 41.8 (CH_2 Gly), 38.4 (β-Phe), (29.1, 28.4, 27.7, 26.5) (CH_3 isop), 18.9 (CH_3 Boc). HRMS (MALDI-TOF/TOF) *m/z*: calcd. for $C_{30}H_{42}NO_{12}Na$ $[M+Na]^+$ 645.2635; found 645.2641.

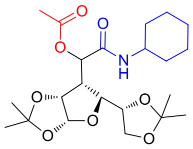

2-(cyclohexylamino)-1-((3aR,5S,6R,6aR)-5-((R)-2,2-dimethyl-1,3-dioxolan-4-yl)-2,2-dimethyltetrahydrofuro[2,3-d][1,3]dioxol-6-yl)-2-oxoethyl acetate (6a) Yield 72% (32 mg); colorless oil; R_f (DS1, DS2) = 0.37, (EtOAc/PE 1:1, v/v); *d.r.* 90:10. Chemical shifts are given for both isomers. ^{1}H NMR ($CDCl_3$) δ 6.04 (d, *J* = 7.9 Hz, 1H, NH), 5.75 (d, *J* = 3.6 Hz, 0.9H, H-3' Alo), 5.73 (d, *J* = 3.7 Hz, 0.1H, H-3' Alo), 5.37 (d, *J* = 6.4 Hz, 0.1H, H-1 Alo), 5.21 (d, *J* = 8.7 Hz, 0.9H, H-1 Alo), 4.69 – 4.62 (m, 1H, H-2 Alo), 4.27 – 4.16 (m, 2H, H-4,5 Alo), 4.03 (dd, *J* = 8.4, 6.5 Hz, 1H, H-6 Alo), 3.95 (dd, *J* = 8.4, 6.6 Hz, 0.9H, H-6 Alo), 3.88 (dd, *J* = 8.1, 5.4 Hz, 0.1H, H-6 Alo), 3.76 (m, 1H, CH-1 CyHex), 2.51 (m, 0.1H, H-3 Alo), 2.40 – 2.28 (m, 0.9H, H-3 Alo), 2.11 (s, 2.7H, CH_3 Ac), 2.09 (s, 0.3H, CH_3 Ac), 1.86 (s, 2H, CyHex), 1.70 –

1.54 (m, 3H, CyHex), 1.50 (s, 3H, CH_3 isop), 1.40 (s, 3H, CH_3 isop), 1.38 – 1.33 (m, 2H, CyHex), 1.32 (s, 3H, CH_3 isop), 1.27 (s, 3H, CH_3 isop), 1.20 – 1.11 (m, 3H, CyHex). ^{13}C NMR ($CDCl_3$) δ (170.2, 167.2) (CO), (112.7, 109.8) (C isop), (105.1, 104.6) (C-1 Alo), (81.6, 81.4, 80.3, 78.5, 76.9, 71.4, 71.3) (C-2,3',4,5), (66.9, 65.8) (C-6 Alo), (48.6) (C-3 Alo), (48.4, 48.3) (CH CyHex), 32.9 (CyHex), (27.0, 26.6, 26.5, 25.7) (CH_3 isop), (25.7, 24.8) (CyHex), 21.1 (CH_3 Ac). HRMS (MALDI-TOF/TOF) *m/z*: calcd. for $C_{22}H_{35}NO_8Na$ $[M+Na]^+$ 464.2260; found 464.2254.

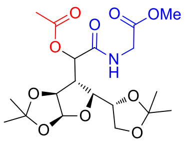

methyl 2-(2-acetoxy-2-((3aS,5S,6R,6aS)-5-((R)-2,2-dimethyl-1,3-dioxolan-4-yl)-2,2-dimethyltetrahydrofuro[2,3-d][1,3]dioxol-6-yl)acetamido)acetate (6c) Yield 74 % (32 mg); colorless oil; R_f (DS1, DS2) = 0.38, (EtOAc/PE 1:1, v/v); *d.r.* 90:10. Chemical shifts are given for both isomers. ^{1}H NMR ($CDCl_3$) δ 6.77 (s, 0.9H, NH), 6.74 (s, 0.1H, NH), 5.76 (d, *J* = 3.6 Hz, 0.9H, H-3' Alo), 5.73 (d, *J* = 3.7 Hz, 0.1H, H-3' Alo), 5.48 (d, *J* = 6.0 Hz, 0.1H, H-1 Alo), 5.38 (d, *J* = 8.2 Hz, 0.9H, H-1 Alo), 4.82 (t, *J* = 4.1 Hz, 0.9H, H-2 Alo), 4.77 (t, *J* = 4.1 Hz, 0.1H, H-2 Alo), 4.26 (dd, *J* = 9.7, 5.0 Hz, 0.9H, H-4 Alo), 4.22 – 4.06 (m, 2.1H, H-4,5 Alo, CH_2 Gly), 4.05 – 4.00 (m, 1H, H-6 Alo), 3.94 (dd, *J* = 8.2, 6.9 Hz, 0.9H, H-6 Alo), 3.87 (dd, *J* = 18.3, 4.4 Hz, 1.1H, H-6 Alo, CH_2 Gly), 3.74 (s, 0.3H, CH_3 Gly), 3.72 (s, 2.7H, CH_3 Gly), 2.52 – 2.45 (m, 0.1H, H-3 Alo), 2.37 – 2.30 (m, 0.9H, H-3 Alo), 2.12 (s, 2.7H, CH_3 Ac), 2.10 (s, 0.3H, CH_3 Gly), 1.77 (s, 0.3H, CH_3 isop), 1.50 (s, 0.3H, CH_3 isop), 1.47 (s, 2.7H, CH_3 isop), 1.39 (s, 2.7H, CH_3 isop), 1.30 (s, 3H, CH_3 isop), 1.28 (s, 3H, CH_3 isop). ^{13}C NMR ($CDCl_3$) δ (170.1, 170.0, 168.8) (CO), (112.7, 109.8) (C isop), (105.1, 104.6) (C-1 Alo), (81.3, 81.2, 79.9, 78.4, 77.3, 76.9, 71.0, 70.7) (C-2,3',4,5), (67.2, 66.0) (C-6 Alo), (52.7, 52.5) (CH_3 Gly), (48.8, 48.7, 48.3) (CH CyHex, C-3 Alo)), (41.3, 41.2) (CH_2 Gly), (26.9, 26.8, 26.7, 26.5, 26.4, 25.6, 25.2) (CH_3 isop), (21.0, 20.9) (CH_3 Ac). HRMS (MALDI-TOF/TOF) *m/z*: calcd. for $C_{19}H_{29}NO_{10}Na$ $[M+Na]^+$ 454.1689; found 454.1696.

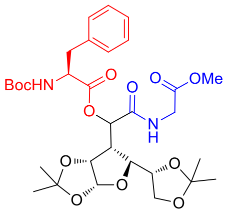

1-((3aS,6aS)-5-(2,2-dimethyl-1,3-dioxolan-4-yl)-2,2-dimethyltetrahydrofuro[2,3-d][1,3]dioxol-6-yl)-2-((2-methoxy-2-oxoethyl)amino)-2-oxoethyl 2-((tert-butoxycarbonyl)amino)-3-phenylpropanoate (6d) Yield 77 % (49 mg); colorless oil; R_f (DS1,2) = 0.47 (EtOAc/PE 1:1, v/v); *d.r.* 90:10. ^{1}H NMR ($CDCl_3$) δ 7.33-7.16 (m, 5H, Ph), 6.92 (s, 1H, NH), 5.75 (d, *J* = 3.7 Hz, 1H, H-3' Alo), 5.49 (d, *J* = 7.6 Hz, 1H, H-1 Alo), 5.01 (d, *J* = 7.4 Hz, 1H, NH Phe), 4.78 (t, *J* = 4.0 Hz, 1H, α-Phe), 4.59 (dd, *J* = 13.3, 6.7 Hz, 1H, H-2 Alo), 4.31 (dd, *J* = 9.7, 4.4 Hz, 1H, H-5 Alo), 4.12 (ddd, *J* = 22.6, 11.3, 5.5 Hz, 2H, H-4 Alo, CH_2 Gly), 3.98 (d, *J* = 6.5 Hz, 2H, H-6 Alo), 3.86 (dd, *J* = 18.0, 4.9 Hz, 1H, CH_2 Gly), 3.72 (s, 3H, CH_3 Gly), 3.22 (dd, *J* = 14.1, 5.7 Hz, 1H, β-Phe), 3.07 (dd, *J* = 14.0, 7.1 Hz, 1H, β-Phe), 2.35 (ddd,

J = 9.9, 7.8, 4.6 Hz, 1H, H-3 Alo), 1.57-1.28 (m, 21H, CH_3 Boc, CH_3 isop). ^{13}C NMR ($CDCl_3$) δ (171.1, 169.9, 168.6) (CO), 155.7 (CO Boc), 136.2 (C Ph), (129.6, 128.8, 127.3) (CH Ph), (112.7, 110.0) (C isop), 104.6 (C-1 Alo), 81.3 (C-2 Alo), 80.5 (C Boc), 79.5 (C-4 Alo), 76.6 (C-5 Alo), 71.3 (C-3' Alo), 65.8 (C-6 Alo), 54.8 (α-Phe), 52.5 (CH_3 Gly), 48.2 (C-3 Alo), 41.3 (CH_2 Gly), 37.9 (β-Phe), 28.49 (CH_3 Boc), (26.86, 26.62, 26.39, 25.44) (CH_3 isop). HRMS (MALDI-TOF/TOF) *m/z*: calcd. for $C_{31}H_{44}N_2O_{12}Na$ $[M+Na]^+$ 659,2792; found 659,2790.

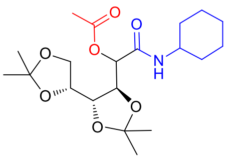

2-(cyclohexylamino)-2-oxo-1-((4R,4'R,5S)-2,2,2',2'-tetramethyl-[4,4'-bi(1,3-dioxolan)]-5-yl)ethyl acetate (7a) Yield 81 % (32 mg); colorless oil; R_f (DS1) = 0.50, R_f(DS2) = 0.48 (EtOAc/PE 1:1, v/v); *d.r.* 55:45. ^{1}H NMR ($CDCl_3$) δ 5.95 (d, J = 7.9 Hz, 0.55H, NH), 5.77 (d, J = 8.1 Hz, 0.45H, NH), 5.37 (d, J = 2.7 Hz, 0.55H, H-1 Ara), 5.28 (d, J = 2.3 Hz, 0.45H, H-1 Ara), 4.37 (dd, J = 7.5, 2.3 Hz, 0.45H, H-2 Ara), 4.32 (t, J = 7.8 Hz, 0.55H, H-3 Ara), 4.10 (dd, J = 8.0, 2.7 Hz, 0.55H, H-2 Ara), 4.08 – 3.99 (m, 2H, H-4, H-5 Ara), 3.94 (dd, J = 8.4, 4.9 Hz, 0.55H, H-5 Ara), 3.89 (m, 0.45H, H-5 Ara), 3.78 – 3.70 (m, 1.45H, H-3 Ara, CH-1 CyHex), 2.15 (s, 1.35H, CH_3 Ac), 2.14 (s, 1.65H, CH_3 Ac), 1.85 (m, 2H, CyHex), 1.64 (m, 2H, CyHex), 1.56 (m, 1H, CyHex), 1.45 (s, 2H, CH_3 isop), 1.36 – 1.26 (m, 12H, CH_3 isop, CyHex), 1.17 – 1.07 (m, 3H, CyHex). ^{13}C NMR ($CDCl_3$) δ (169.3, 169.13, 166.7, 165.9) (CO), (110.5, 110.2, 110.2, 110.1) (C isop), (80.1, 79.4) (C-2 Ara), (77.4, 77.2) (C-3 Ara), (77.1, 76.9) (C-4 Ara), (73.7, 72.7) (C-1 Ara), 67.4 (C-5 Ara), (48.4, 48.3) (CH CyHex), (33.2, 33.1, 33.1, 33.0) (CyHex), (27.4, 27.4, 27.0, 27.0, 26.7) (CH_3 isop), (25.7, 25.7) (CyHex), (25.6, 25.3) (CH_3 isop), (24.9, 24.8) (CyHex), (21.1, 21.0) (CH_3 Ac). HRMS (MALDI-TOF/TOF) *m/z*: calcd. for $C_{20}H_{33}NO_7Na$ $[M+Na]^+$ 422.2155; found 422.2138.

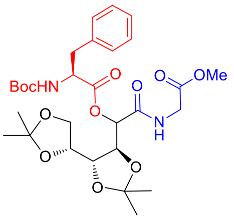

2-((2-methoxy-2-oxoethyl)amino)-2-oxo-1-((4R,4'R,5S)-2,2,2',2'-tetramethyl-[4,4'-bi(1,3-dioxolan)]-5-yl)ethyl 2-((tert-butoxycarbonyl)amino)-3-phenylpropanoate (7d) Yield 78 % (46 mg); colorless oil; R_f (DS1) = 0.48 (EtOAc/PE 1:1, v/v); *d.r.* 55:45. ^{1}H NMR ($CDCl_3$) δ 7.60 (m, 0.55H, NH), 7.36 – 7.25 (m, 5H, CH Ph), 7.11 (m, 0.45H, NH), 5.50 (d, J = 2.7 Hz, 0.55H, H-1 Ara), 5.44 (d, J = 1.6 Hz, 0.45H, H-1 Ara), 5.12 (d, J = 6.0 Hz, 0.45H, α-Phe), 4.97 (d, J = 7.1 Hz, 0.55H, α-Phe), 4.65 – 4.45 (m, 1.45H, H-2 Ara, NH), 4.29 (t, J = 7.4 Hz, 0.55H, H-3 Ara), 4.15 – 3.92 (m, 5.45H, H-2, H-3, H-4, H-5, CH_2 Gly)), 3.81 (td, J = 7.9, 3.4 Hz, 0.55H, H-5 Ara), 3.73 (s, 1.65H, CH_3 Gly), 3.71 (s, 1.35H, CH_3 Gly), 3.21 – 3.05 (m, 2H, β-Phe), 1.47 – 1.27 (m, 21H, CH_3 Boc, CH_3 isop). ^{13}C NMR ($CDCl_3$) δ (171.7, 170.6, 169.8, 169.6, 168.0, 167.0) (CO), 156.0 (CO Boc), (135.9, 135.5) (C Ph), (129.4, 129.3, 129.2, 129.0, 127.6, 127.4) (CH Ph), (110.5, 110.3, 110.0, 109.9) (C isop), (81.0, 80.8) (C Boc), (79.6, 79.2) (C-2 Ara), 77.1 (C-3 Ara), (76.8, 76.6)

(C-4 Ara), (74.2, 72.9) (C-1 Ara), (67.47, 67.2) (C-5 Ara), (55.9, 54.8) (α-Phe), (52.4, 52.3) (CH_3 Gly), (41.3, 41.1) (CH_2 Gly), (37.8, 37.7) (β-Phe), (28.4, 28.4) (CH_3 Boc), (27.4, 27.4, 26.9, 26.9, 26.7, 26.6, 25.5, 25.1) (CH_3 isop). HRMS (MALDI-TOF/TOF) *m/z*: calcd. for $C_{29}H_{42}N_2O_{11}Na$ $[M+Na]^+$ 617.2686; found 617.2687.

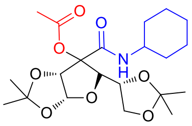

(3aR,5S,6aR)-6-(cyclohexylcarbamoyl)-5-((R)-2,2-dimethyl-1,3-dioxolan-4-yl)-2,2-dimethyltetrahydrofuro[2,3-d][1,3]dioxol-6-yl acetate (8a) Yield 67 % (29 mg); colorless oil; R_f (DS1) = 0.70, R_f (DS2) = 0.50 (EtOAc/PE 1:1, v/v); *d.r.* 55:45. Major isomer: ^1H NMR ($CDCl_3$): δ 7.40 (d, *J* = 7.5 Hz, 1H, NH), 5.88 (d, *J* = 3.6 Hz, 1H, H-1 GlcF), 4.87 (d, *J* = 3.6 Hz, 1H, H-2, GlcF), 4.45 (t, *J* = 4.7 Hz, 1H, H-5, GlcF), 4.42 (d, *J* = 2.8 Hz, 1H, H-4 GlcF), 4.13 (dd, *J* = 8.4, 6.5 Hz, 1H, H-6a GlcF), 4.06 (dd, *J* = 8.4, 6.9 Hz, 1H, H-6b, GlcF), 3.80 – 3.73 (m, 1H, CH-1 CyHex), 2.09 (m, 3H, CH_3 Ac), 1.93 – 1.82 (m, 2H, CyHex), 1.67 – 1.60 (m, 2H, CyHex), 1.59 (m, 5H, CH_3 isop, CyHex), 1.41 (s, 3H, CH_3 isop), 1.36 (m, 4H, CH_3 isop, CyHex), 1.33 (s, 3H, CH_3 isop), 1.27 – 1.15 (m, 3H, CyHex). ^{13}C NMR ($CDCl_3$): δ (170.0, 163.9) (CO), (114.0, 108.7) (C isop), 105.4 (C-1 GlcF), 85.6 (C-3 GlcF), 82.8 (C-2 GlcF), 82.3 (C-4 GlcF), 73.5 (C-5 GlcF), 65.0 (C-6 GlcF), 48.4 (CH CyHex), (32.7, 32.7) (CyHex), (27.0, 26.7, 26.5) (CH_3 isop), 25.8 (CyHex), 25.5 (CH_3 isop), (24.6, 24.5) (CyHex), 21.5(CH_3 Ac). Minor isomer: ^1H NMR ($CDCl_3$): δ 6.17 (d, *J* = 8.1 Hz, 1H, NH), 5.85 (d, *J* = 3.8 Hz, 1H, H-1 GlcF), 5.33 (d, *J* = 3.8 Hz, 1H, H-2 GlcF), 4.10 – 4.06 (m, 1H, H-6a GlcF), 4.02 (d, *J* = 9.3 Hz, 1H, H-5 GlcF), 3.97 – 3.91 (m, 2H, H-4, H-6b GlcF), 3.83 – 3.77 (m, 1H, CH-1 CyHex), 2.11 (s, 3H, CH_3 Ac), 1.85 (ddd, *J* = 27.8, 12.4, 3.5 Hz, 2H, CyHex), 1.69 – 1.54 (m, 4H, CyHex), 1.50 (s, 3H, CH_3 isop), 1.46 (s, 3H, CH_3 isop), 1.41 – 1.33 (m, 1H, CyHex), 1.33 (s, 3H, CH_3 isop), 1.31 (s, 3H, CH_3 isop), 1.22 – 1.08 (m, 3H, CyHex). ^{13}C NMR ($CDCl_3$): δ (169.0, 166.6) (CO), (112.8, 110.1) (C isop), 105.7 (C-1 GlcF), 85.3 (C-3 GlcF), 80.9 (C-2 GlcF), 80.4 (C-4 GlcF), 74.6 (C-5 GlcF), 68.4 (C-6 GlcF), 48.2 (CH CyHex), (33.1, 32.7) (CyHex), (27.1, 27.0, 26.8, 25.8) (CH_3 isop), (25.7, 24.7, 24.7) (CyHex), 21.0 (CH_3 Ac). HRMS (MALDI-TOF/TOF) *m/z*: calcd. for $C_{21}H_{33}NO_8$ $[M+H]^+$ 428.2284; found 428.2302.

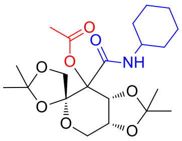

(3aR,4'R,7aR)-7-(cyclohexylcarbamoyl)-2,2,2',2'-tetramethyltetrahydrospiro[[1,3]dioxolo[4,5-c]pyran-6,4'-[1,3]dioxolan]-7-yl acetate (9a) Yield 51 % (22 mg); colorless oil; R_f (DS1) = 0.80, R_f (DS2) = 0.40 (EtOAc/PE 1:1, v/v); *d.r.* 55:45. Major isomer: ^1H NMR ($CDCl_3$): δ 5.99 (d, *J* = 7.7 Hz, 1H, NH), 5.30 (d, *J* = 7.0 Hz, 1H, H-4 FruP), 4.60 (dt, *J* = 7.3, 3.7 Hz, 1H, H-5 FruP), 4.57 (d, *J* = 9.6 Hz, 1H, H-1a FruP), 4.07 (m, 1H, H-1b FruP), 4.05 (m, 1H, H-6a FruP), 3.80 – 3.74 (m, 1H, CH-1 CyHex), 3.68 (dd, *J* = 12.8, 3.4 Hz, 1H, H-6b FruP), 2.11 (s, 3H, CH_3 Ac), 1.86 (t, *J* = 17.8 Hz, 2H, CyHex), 1.65-1.57 (m, 4H, CyHex), 1.47 (s, 6H, CH_3 isop), 1.46 (s, 3H, CH_3 isop), 1.38 (s, 1H, CyHex), 1.30 (s, 3H, CH_3 isop),

1.21 – 1.06 (m, 3H, CyHex). ^{13}C NMR ($CDCl_3$): δ (168.8, 166.6) (CO), (110.6, 109.5) (C isop), 104.5 (C-2 FruP), 80.5 (C-3 FruP), 73.1 (C-4 FruP), 72.8 (C-5 FruP), 72.3 (C-1 FruP), 64.7 (C-6 FruP), 48.3 (CH-1 CyHex), (32.9, 32.7) (CyHex), (26.9, 26.6, 26.1, 25.8, 25.0, 24.7, 24.6) (CH_3 isop), 21.6 (CH_3 Ac). Minor isomer: ^{1}H NMR ($CDCl_3$): δ 6.66 (d, *J* = 7.3 Hz, 1H, NH), 5.08 (d, *J* = 7.2 Hz, 1H, H-4 FruP), 4.31 (d, *J* = 9.9 Hz, 1H, H-1a FruP), 4.30 – 4.24 (m, 1H, H-5 FruP), 4.20 (dd, *J* = 12.6, 3.3 Hz, 1H, H-6a FruP), 3.93 (d, *J* = 9.9 Hz, 1H, H-1b FruP), 3.79 (dd, *J* = 12.6, 0.9 Hz, 1H, H-6b FruP), 3.77 – 3.73 (m, 1H, CyHex CH-1), 2.12 (s, 3H, CH_3 Ac), 1.92 (dd, *J* = 33.6, 9.8 Hz, 2H, CyHex), 1.66 (m, 2H, CyHex), 1.58 (m, 2H, CyHex), 1.51 (s, 3H, CH_3 isop), 1.49 (s, 3H, CH_3 isop), 1.43 (s, 3H, CH_3 isop), 1.36 (s, 3H, CH_3 isop), 1.33 (m, 1H, CyHex), 1.23-1.12 (m, 3H, CyHex). ^{13}C NMR ($CDCl_3$): δ (169.3, 164.6) (CO), (111.6, 110.8) (C isop), 103.9 (C-2 FruP), 81.0 (C-3 FruP), 73.60 (C-1 FruP), 73.4 (C-4 FruP), 73.0 (C-5 FruP), 64.4 (C-6 FruP), 48.9 (CH-1 CyHex), (32.9, 32.7) (CyHex), (26.4, 26.3, 26.2) (CH_3 isop), 25.8 (CyHex), 25.3 (CH_3 isop), 24.9 (CyHex), 21.7 (CH_3 Ac). HRMS (MALDI-TOF/TOF) *m/z*: calcd. for $C_{21}H_{33}NO_8Na$ $[M+Na]^+$ 450.2104; found 450.2107.

3. Post-condensation modification of selected Passerini products

Boc depritection and coupling of amino acid: Passerini product **2d** (10 mg, 0.016 mmol) was dissolved in TFA-DCM (1:1, v/v, 100 µL), reaction was stirred at room temperature for 2 min and the reaction mixture was evaporated with toluene. Boc-Pro-OH (4 mg, 0.018 mmol, 1.2 eq), BOP (10 mg, 0.023 mmol, 1.5 eq), and HOBt (3.5 mg, 0.023 mmol, 1.5 eq) were dissolved in 1 mL DCM:DMF (1:1) followed by the addition of Boc-deprotected compound (8 mg, 0.015 mmol) and NMM (2eq). Reaction mixture was stirred for 24 h. Product was purified by flash column chromatography (petrol ether/ethyl acetate, v/v = 1/6).

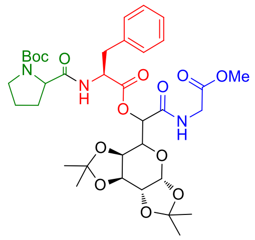

tert-butyl 2-((1-(2-((2-methoxy-2-oxoethyl)amino)-2-oxo-1-((3aR,5aS,8aS,8bR)-2,2,7,7-tetramethyltetrahydro-3aH-bis([1,3]dioxolo)[4,5-b:4',5'-d]pyran-5-yl)ethoxy)-1-oxo-3-phenylpropan-2-yl)carbamoyl)pyrrolidine-1-carboxylate (2j) Yield 78 % (9 mg); colorless oil; R_f (DS1,2) = 0.55 (EtOAc/PE 6/1, v/v); *d.r.* 70:30. ^{1}H NMR (600 MHz, MeOD) δ 7.36 – 7.18 (m, 5H, Ph), 5.52 (d, *J* = 4.9 Hz, 0.3H, H-1 Gal), 5.49 (d, *J* = 4.7 Hz, 0.7H, H-1 Gal), 5.44 (m, 0.3H, H-6 Gal) 5.13 (m, 0.7H, H-6 Gal), 4.84 (s, 1H, α-Phe), 4.66 (dd, *J* = 8.0, 2.3 Hz, 1H, H-3 Gal), 4.39 (ddd, *J* = 11.1, 4.7, 2.4 Hz, 1H, H-2 Gal), 4.28 – 4.18 (m, 2H, H-4,5 Gal), 4.15 (bs, 1H, α-Pro), 4.06 – 3.92 (m, 2H, CH_2 Gly), 3.74 (d, *J* = 2.7 Hz, 3H, CH_3 Gly), 3.44 (m, 1H, δ-Pro), 3.39 – 3.34 (m, 2H, δ-Pro, β-Phe), 3.01 (bs, 1H, β-Phe), 2.17 (m, 1H, β-Pro), 1.91 (m, 1H, β-Pro), 1.78 (m, 2H, γ-Pro), 1.55 (d, *J* = 11.6 Hz, 3H, CH_3 isop), 1.46 (d, *J* = 16.2 Hz, 6H, CH_3 isop), 1.37 – 1.24 (m, 12H, CH_3 isop, Boc). ^{13}C NMR (151 MHz, MeOD) δ (170.0, 169.8, 169.3, 169.1, 168.5, 167.7, 167.4) (CO), 136.4

(C Ph), (128.4, 127.6, 125.9) (CH Ph), (108.9, 108.5, 108.3) (C isop), (95.9, 95.7) (C-1 Gal), (72.5, 71.1, 70.3, 70.3, 70.2, 70.0, 69.5, 67.2, 66.5) (C-2,3,4,5,6 Gal), 59.7 (α-Pro), (53.2, 52.9) (α-Phe), (50.7, 50.7) (CH_3 Gly), 45.9 (δ-Pro), (40.1, 39.9) (CH_2 Gly), (36.3, 36.0) (β-Phe), 30.3 (Boc) , (26.7, 24.5, 24.4, 23.3) (CH_3 isop), (22.8, 22.5) (γ-Pro). HRMS (MALDI-TOF/TOF) *m/z*: calcd. for $C_{35}H_{49}N_3O_{13}Na$ $[M+Na]^+$ 742.3163; found 742.3190.

Boc deprotection and coupling of amino acid: Passerini product **5d** (13 mg, 0.02 mmol) was dissolved in TFA-DCM (1:1, v/v, 100 µL), reaction was stirred at room temperature for 2 min and then the solvent was evaporated. Boc-Phe-OH (6.4 mg, 0.024 mmol, 1.2 eq), BOP (13 mg, 0.03 mmol, 1.5 eq), and HOBt (4.6 mg, 0.03 mmol, 1.5 eq) were dissolved in 1 mL DCM:DMF (1:1) followed by the addition of Boc-deprotected compound and NMM (2 eq). Reaction mixture was stirred for 24 h, solvent evaporated and product extracted with DCM. Product was purified by flash column chromatography (petrol ether/ethyl acetate, v/v = 1/1).

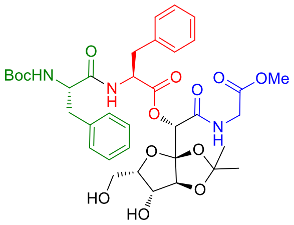

(S)-(S)-1-((3aR,5S,6R,6aS)-6-hydroxy-5-(hydroxymethyl)-2,2-dimethyltetrahydrofuro[2,3-d][1,3]dioxol-3a-yl)-2-((2-methoxy-2-oxoethyl)amino)-2-oxoethyl 2-((S)-2-((tert-butoxycarbonyl)amino)-3-phenylpropanamido)-3-phenylpropanoate (5j) Yield 65 % (9.5 mg); colorless oil; R_f = 0.30 (EtOAc/PE 1/1, v/v). ^{1}H NMR ($CDCl_3$): δ 7.68 (s, 1H, NH), 7.38 – 7.16 (m, 8H, CH Ph), 6.91 (dd, *J* = 6.4, 2.9 Hz, 2H, CH Ph), 6.00 (d, *J* = 5.4 Hz, 1H, NH Phe), 5.77 (s, 1H, H-1 Sor), 5.16 (d, *J* = 7.5 Hz, 1H, NH Phe), 4.93 (s, 1H, H-5 Sor), 4.60 (dt, *J* = 8.8, 5.3 Hz, 1H, α-Phe), 4.35 – 4.12 (m, 4H, α-Phe, CH_2 Gly, H-3,4 Sor), 3.91 – 3.78 (m, 3H, CH_2 Gly, H-6 Sor), 3.75 (s, 3H, CH_3 Gly), 3.14 (dd, *J* = 14.3, 5.1 Hz, 1H, β-Phe), 3.02 (d, *J* = 7.1 Hz, 2H, β-Phe), 2.94 – 2.81 (m, 1H, β-Phe), 1.91 (bs, 2H, OH), 1.49 (s, 3H, CH_3 isop), 1.34 – 1.23 (m, 12H, CH_3 isop, Boc). ^{13}C NMR ($CDCl_3$): δ (173.0, 170.0, 169.30, 168.3) (CO), (137.1, 135.2) (C Ph), (129.9, 129.2, 129.1, 129.1, 127.72, 127.5) (CH Ph), 112.7 (C isop), 112.0 (C-2 Sor), (88.0, 82.7, 80.6, 76.7, 75.2) (C-1,3,4,5 Sor), 61.2 (C-6 Sor), (55.9, 54.3) (α-Phe), 52.8 (CH_3 Gly), 41.1 (CH_2 Gly), (38.6, 36.9) (β-Phe), 28.5 (Boc), (27.4, 26.3) (CH_3 isop). HRMS (MALDI-TOF/TOF) *m/z*: calcd. for $C_{35}H_{49}N_3O_{13}Na$ $[M+Na]^+$ 742.3163; found 742.3190.

Fmoc deprotection: A solution of 20 % piperidine in DMF (0.2 mL) was added to the Passerini product **2k** (20 mg, 0.03 mmol), the mixture was stirred at room temperature for 15 min and then the solvent was evaporated. Product was purified by flash column chromatography (petrol ether/ethyl acetate, v/v = 1/1).

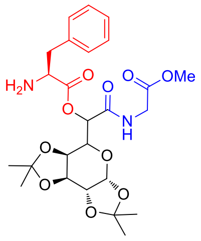

2-((2-methoxy-2-oxoethyl)amino)-2-oxo-1-((3aR,5aS,8aS,8bR)-2,2,7,7-tetramethyltetrahydro-3aH-bis([1,3]dioxolo)[4,5-b:4',5'-d]pyran-5-yl)ethyl 2-amino-3-phenylpropanoate (2l) Yield 77 % (12 mg); colorless oil; R_f (DS1,2) = 0.40 (EtOAc/PE 1:1, v/v); *d.r.* 70:30. ^{1}H NMR ($CDCl_3$): δ 7.25 (m, 5H, Ph), 5.45 (d, *J* = 4.9 Hz, 0.7H, H-1 Gal), 5.41 (d, *J* = 4.6 Hz, 0.3H, H-1 Gal), 4.91 – 4.84 (m, 0.7H, H-6 Gal), 4.75 (td, *J* = 8.3, 4.9 Hz, 0.3H, H-6 Gal), 4.62 (ddd, *J* = 14.4, 8.0, 2.5 Hz, 1H, H-3 Gal), 4.46 (dd, *J* = 8.0, 1.6 Hz, 0.3H, α-Phe), 4.40 (dd, *J* = 7.9, 1.7 Hz, 0.7H, α-Phe), 4.31 (dd, *J* = 4.9, 2.5 Hz, 1H, H-2 Gal), 4.28 – 3.87 (m, 4H, CH_2 Gly, H-5,4 Gal), 3.72 (m, 3H, CH_3 Gly), 3.29 (ddd, *J* = 37.2, 14.3, 5.2 Hz, 1H, β-Phe), 3.15 – 3.10 (m, 1H, β-Phe), 2.95 (s, 1H, NH), 2.88 (s, 1H, NH), 1.51 – 1.25 (m, 12H, CH_3 isop). ^{13}C NMR ($CDCl_3$): δ (171.7, 170.4, 170.3, 170.1, 169.8, 169.5) (CO), (136.1, 135.8) (C Ph), (128.8, 128.8, 128.2, 126.5, 126.4) (CH isop), (109.7, 109.1, 109.1, 108.8) (CH_3 isop), (95.9, 95.4) (C-1 Gal), (73.5, 73.5, 70.3, 70.2, 70.2, 70.0, 69.8, 68.8, 67.8, 66.5) (C-2,3,4,5,6 Gal), (53.6, 53.2) (α-Phe), (51.7, 51.5) (CH_3 Gly), (40.7, 40.6) (CH_2 Gly), (36.9, 36.1) (β-Phe), (25.5, 25.4, 25.3, 25.2, 24.6, 24.3, 23.7, 23.3) (CH_3 isop). HRMS (MALDI-TOF/TOF) *m/z*: calcd. for $C_{25}H_{34}N_2O_{10}Na$ $[M+Na]^+$ 545.2111; found 545.2109.

4. Base-mediated deprotection of selected Passerini products - sugar α-hydroxy acids

General procedure for the reactions:

Passerini product (0.035 mmol) was dissolved in MeOH (0.5 mL) and 1N NaOH (2 eq) was added. Reaction was stirred under reflux (10 min) or at room temperature (30 min) and followed by TLC (EtOAc/EtOH/HOAc/H_2O 70:10:2:2, v/v). Solvent was evaporated and the product was purified by flash column chromatography.

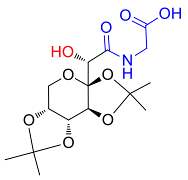

2-((S)-2-hydroxy-2-((3aS,5aR,8aR,8bS)-2,2,7,7-tetramethyltetrahydro-3aH-bis([1,3]dioxolo)[4,5-b:4',5'-d]pyran-3a-yl)acetamido)acetic acid (1m) Yield 91 % (11.5 mg); colorless oil; R_f = 0.74 (EtOAc/EtOH/HOAc/H_2O 70:10:2:2, v/v). ^{1}H NMR ($CDCl_3$) δ 7.36 – 7.29 (m, 1H, NH), 5.52 (bs, 2H, OH), 4.66 (d, *J* = 2.6 Hz, 1H, H-3 Fru), 4.61 (dd, *J* = 7.8, 2.6 Hz, 1H, H-4 Fru), 4.22 (d, *J* = 8.2 Hz, 1H, H-5 Fru), 4.16 – 4.09 (m, 2H, H-1 Fru, CH_2 Gly), 3.99 (dd, *J* = 18.1, 4.6 Hz, 1H, CH_2 Gly), 3.90 (d, *J* = 11.7 Hz, 1H, H-6 Fru), 3.80 (d, *J* = 12.9 Hz, 1H, H-6 Fru), 1.47 (s, 3H, CH_3 isop), 1.46 (s, 3H, CH_3 isop), 1.38 (s, 3H, CH_3 isop), 1.33 (s, 3H, CH_3 isop). ^{13}C NMR ($CDCl_3$) δ 171.3 (CO), (109.7, 109.4) (C isop), 103.5 (C-2 Fru), (71.6, 70.9, 70.5, 70.4) (C-1,3,4,5 Fru), 61.9 (c-6 Fru), 42.4 (CH_2 Gly), (26.6, 26.2, 25.7, 24.2) (CH_3 isop). HRMS (MALDI-TOF/TOF) *m/z*: Cclcd. for $C_{15}H_{23}NO_9Na$ $[M+Na]^+$ 384.1271; found 384.1269.

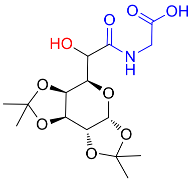

2-(2-hydroxy-2-((3aR,5R,5aS,8aS,8bR)-2,2,7,7-tetramethyltetrahydro-3aH-bis([1,3]dioxolo)[4,5-b:4',5'-d]pyran-5-yl)acetamido)acetic acid (2m) Yield 79 % (10 mg); colorless oil; R_f (DS1,2) = 0.58 (EtOAc/EtOH/HOAc/H_2O 70:10:2:2, v/v); *d.r.* 70:30. ^1H NMR ($CDCl_3$) δ 7.44 (s, 1H, NH), 5.54 (dd, J = 16.6, 4.7 Hz, 1H, H-1 Gal), 5.13 (bs, 2H, OH), 4.61 (dt, J = 8.0, 4.0 Hz, 1H, H-3 Gal), 4.48 (d, J = 8.1 Hz, 1H, H-4 Gal), 4.38 – 4.23 (m, 2H, H-2,6 Gal), 4.12 – 3.96 (m, 3H, CH_2 Gly, H-5 Gal), 1.53 – 1.26 (m, 12H, CH_3 isop). ^{13}C NMR ($CDCl_3$) δ (173.5, 171.8) (CO), (110.1, 109.8, 109.60, 109.6) (C isop), (96.6, 96.5) (C-1 Gal), (73.7, 72.9, 71.3, 71.2, 70.9, 70.8, 70.7, 70.4, 67.8, 66.7) (C-2,3,4,5,6), 41.78 (CH_2 Gly), (26.1, 26.1, 26.0, 25.2, 25.1, 24.4, 24.2) (CH_3 isop). HRMS (MALDI-TOF/TOF) *m/z*: calcd. for $C_{15}H_{23}NO_9Na$ $[M+Na]^+$ 384.1271; found 384.1272.

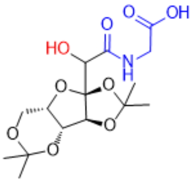

2-((S)-2-hydroxy-2-((3aS,3bR,7aS,8aS)-2,2,5,5-tetramethyltetrahydro-3aH-[1,3]dioxolo[4',5':4,5]furo[3,2-d][1,3]dioxin-8a-yl)acetamido)acetic acid (5m) Yield 76 % (9.6 mg); colorless oil; R_f (DS1,2) = 0.75 (EtOAc/EtOH/HOAc/H_2O 70:10:2:2, v/v); *d.r.* nd. ^1H NMR ($CDCl_3$) δ 7.35 (d, J = 5.2 Hz, 1H), 4.63 (s, 1H), 4.32 (d, J = 8.0 Hz, 2H), 4.16 (s, 1H), 4.13 – 3.98 (m, 4H), 1.40 (m, 12H). ^{13}C NMR ($CDCl_3$) δ 171.1 (CO), (114.5, 113.7) (C isop), 97.9 (C-2 Sor), 85.3 (C-5), (73.7, 73.0, 70.8) (C-1,3,4), 60.5 (C-6), 42.5 (CH_2 Gly), (29.2, 27.7, 26.6, 18.8) (CH_3 isop). HRMS (MALDI-TOF/TOF) *m/z*: calcd. for $C_{15}H_{23}NO_9Na$ $[M+Na]^+$ 384.1271; found 384.1281.

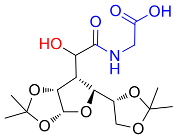

2-(2-((3aS,5S,6R,6aS)-5-((R)-2,2-dimethyl-1,3-dioxolan-4-yl)-2,2-dimethyltetrahydrofuro[2,3-d][1,3]dioxol-6-yl)-2-hydroxyacetamido)acetic acid (6m) Yield 70 % (0.11 mmol, 29 mg); colorless oil; R_f (DS1,2) = 0.62 (EtOAc/EtOH/HOAc/H_2O 70:10:2:2, v/v); *d.r.* 90:10. ^{1}H NMR ($CDCl_3$): δ 7.44 (t, *J* = 5.3 Hz, 0.9H, NH), 7.35 – 7.31 (m, 0.1H, NH), 5.76 (d, *J* = 3.4 Hz, 0.1H, H-3 Alo), 5.74 (d, *J* = 3.5 Hz, 0.9H, H-3 Alo), 5.58 (s, 2H, OH), 4.86 (t, *J* = 4.0 Hz, 0.9H, H-2 Alo), 4.83 (t, *J* = 4.0 Hz, 0.1H, H-2 Alo), 4.63 (d, *J* = 3.3 Hz, 0.1H, H-4 Alo), 4.51 (d, *J* = 7.0 Hz, 0.9H, H-4 Alo), 4.28 – 4.22 (m, 0.1H, H-5 Alo), 4.20 (dd, *J* = 9.5, 6.7 Hz, 0.9H, H-5 Alo), 4.12 – 4.01 (m, 3H, H-1 Alo, H-6, CH_2 Gly), 4.00 – 3.90 (m, 2H, H-6 Alo, CH_2 Gly), 2.66 (dt, *J* = 10.2, 3.8 Hz, 0.1H, H-3 Alo), 2.28 (ddd, *J* = 9.7, 7.1, 4.7 Hz, 0.9H, H-3 Alo), 1.54 (s, 0.3H, CH_3 isop), 1.52 (s, 2.7H, CH_3 isop), 1.43 (s, 0.3H, CH_3 isop), 1.41 (s, 2.7H, CH_3 isop), 1.33 (s, 2.7H, CH_3 isop), 1.31 (s, 2.7H, CH_3 isop), 1.28 (s, 0.6H, CH_3 isop). ^{13}C NMR ($CDCl_3$): δ 173.2 (CO), (112.7, 110.4) (C isop), 104.7 (C1 Alo), (82.9, 79.5, 77.0, 69.3) (C-2,4,5,3 Alo), 67.2 (C-6 Alo), 51.1 (CH_3 Gly), 47.6 (C-3 Alo), 41.6 (CH_2 Gly), (27.0, 26.5, 26.4, 25.5) (CH_3 isop). HRMS (MALDI-TOF/TOF) *m/z*: calcd. for $C_{16}H_{25}NO_9Na$ $[M+Na]^+$ 398.1427; found 398.1445.

^{1}H NMR spectra of reaction mixtures (determination of the *d.r.*)

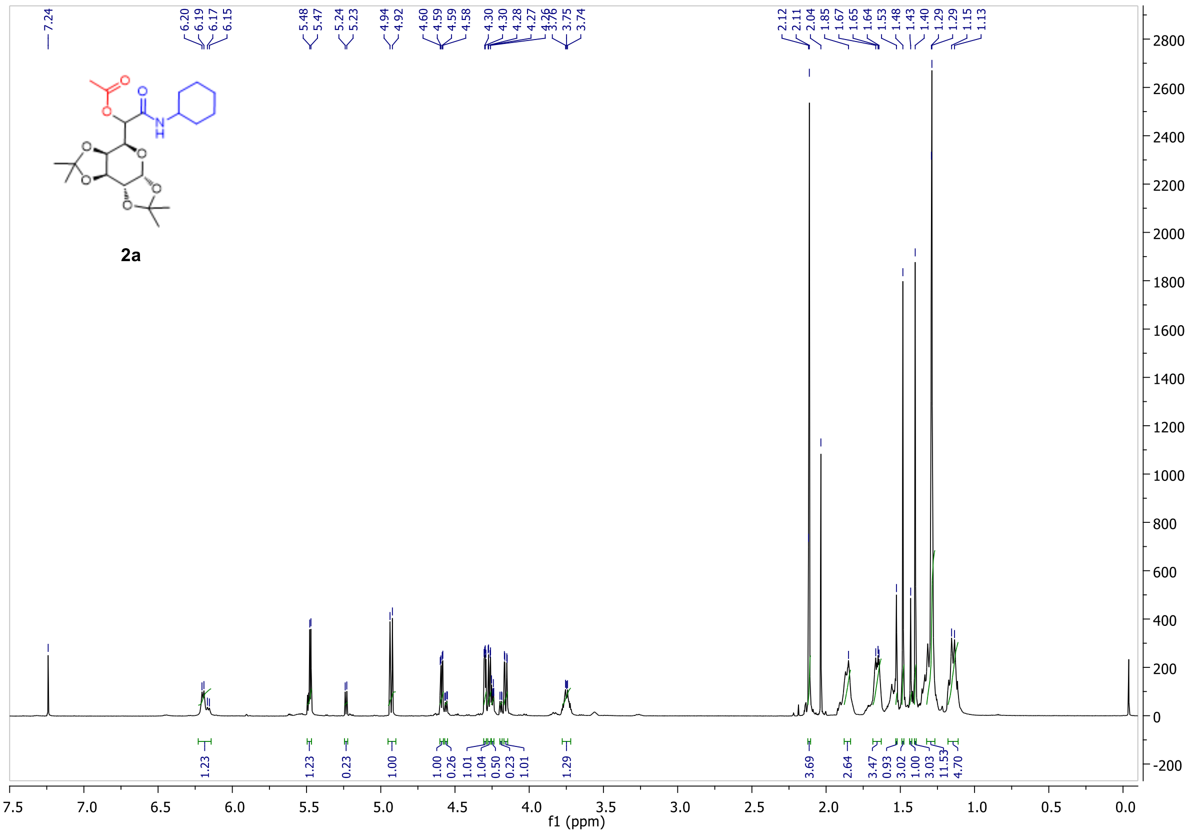

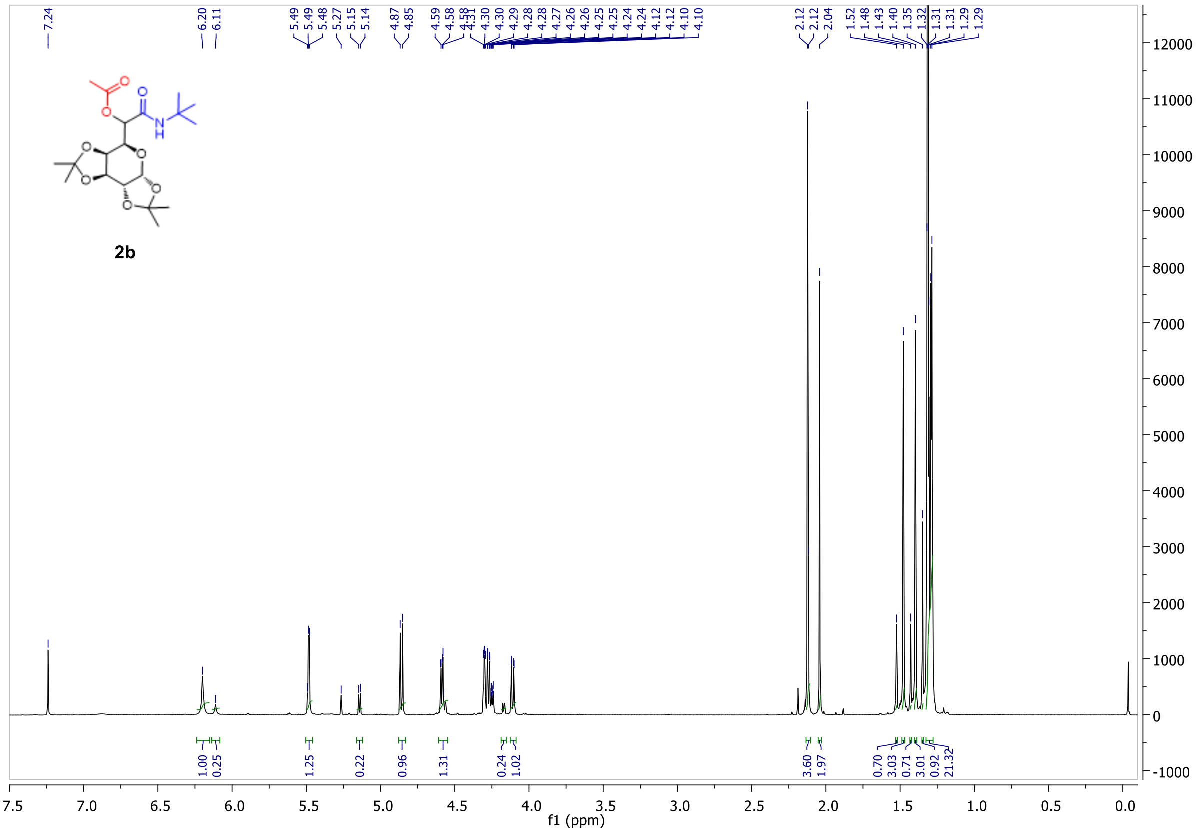

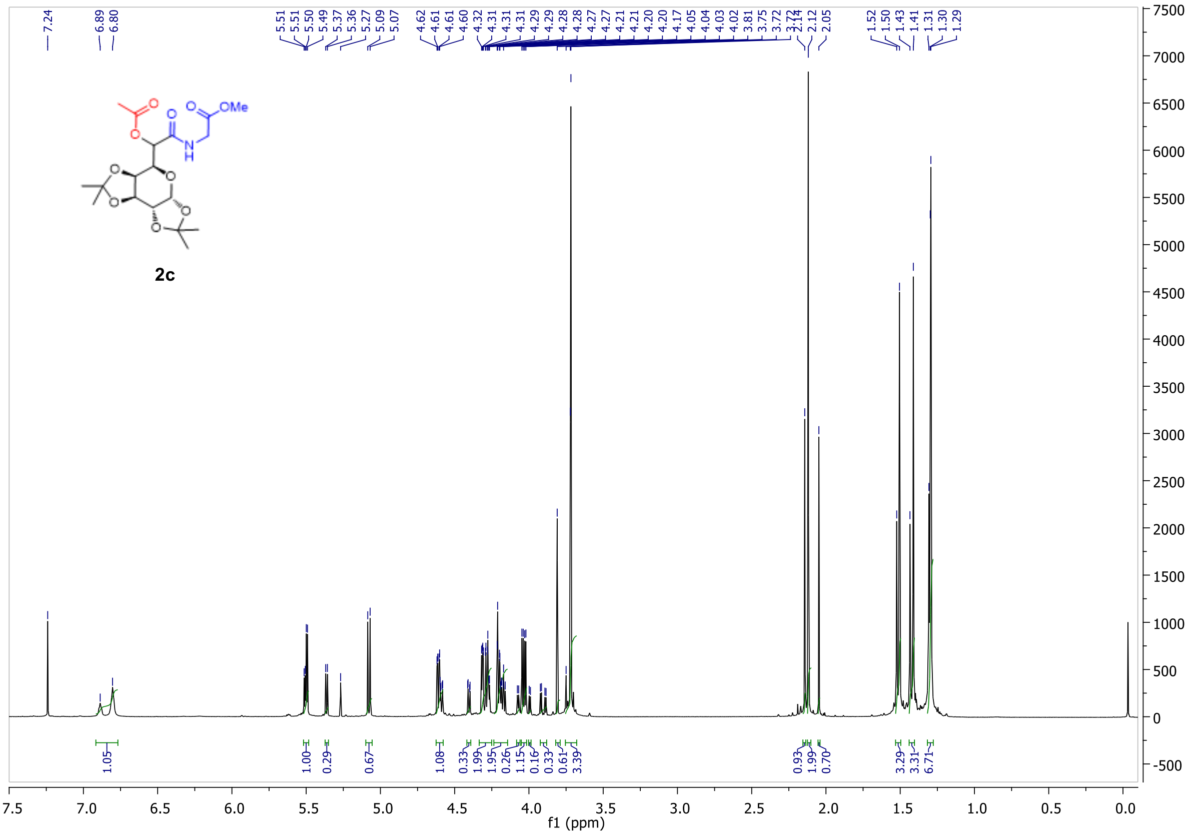

2c

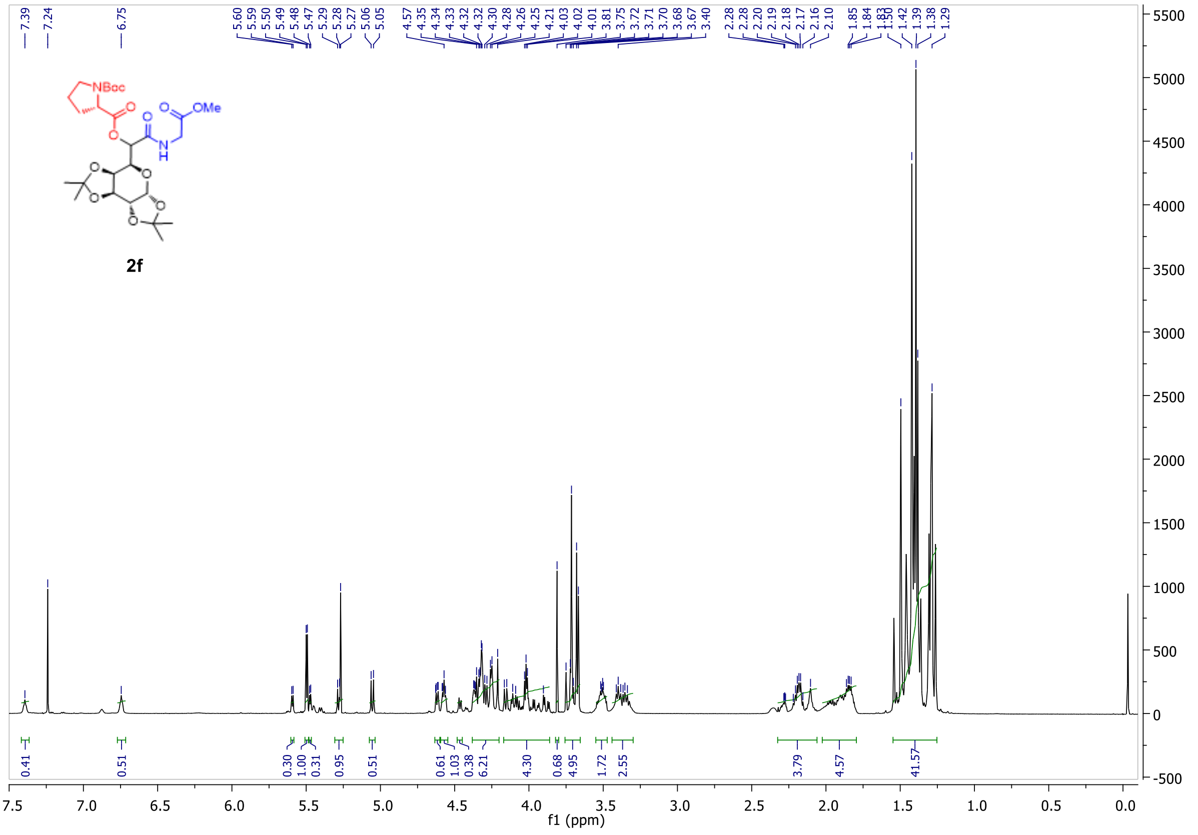

2f

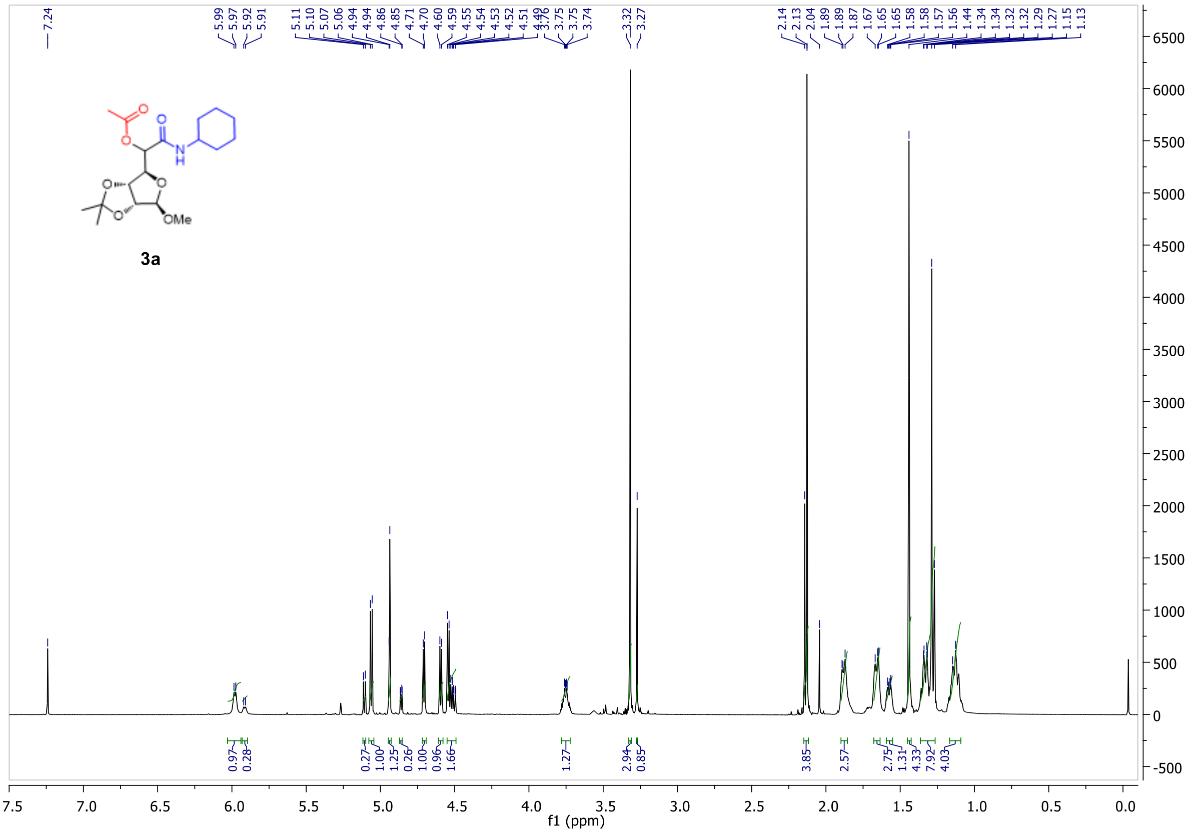

3a

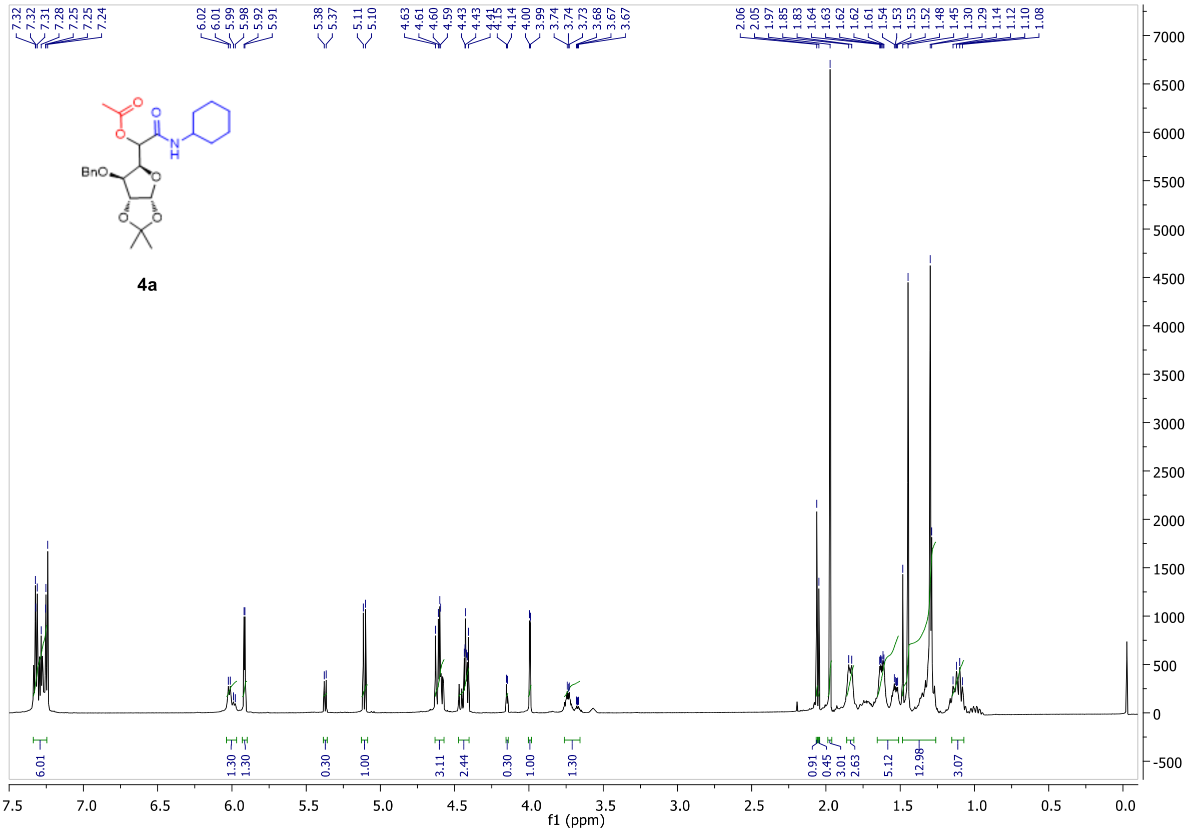

4a

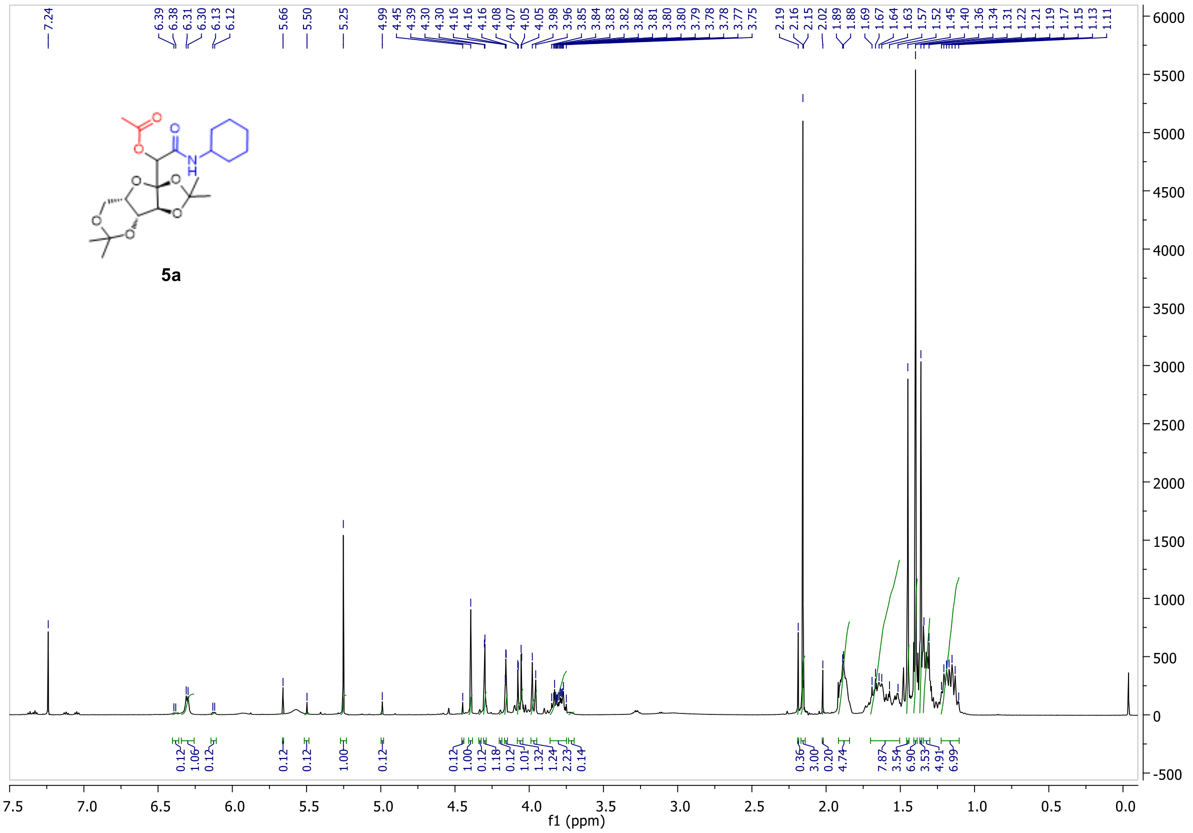

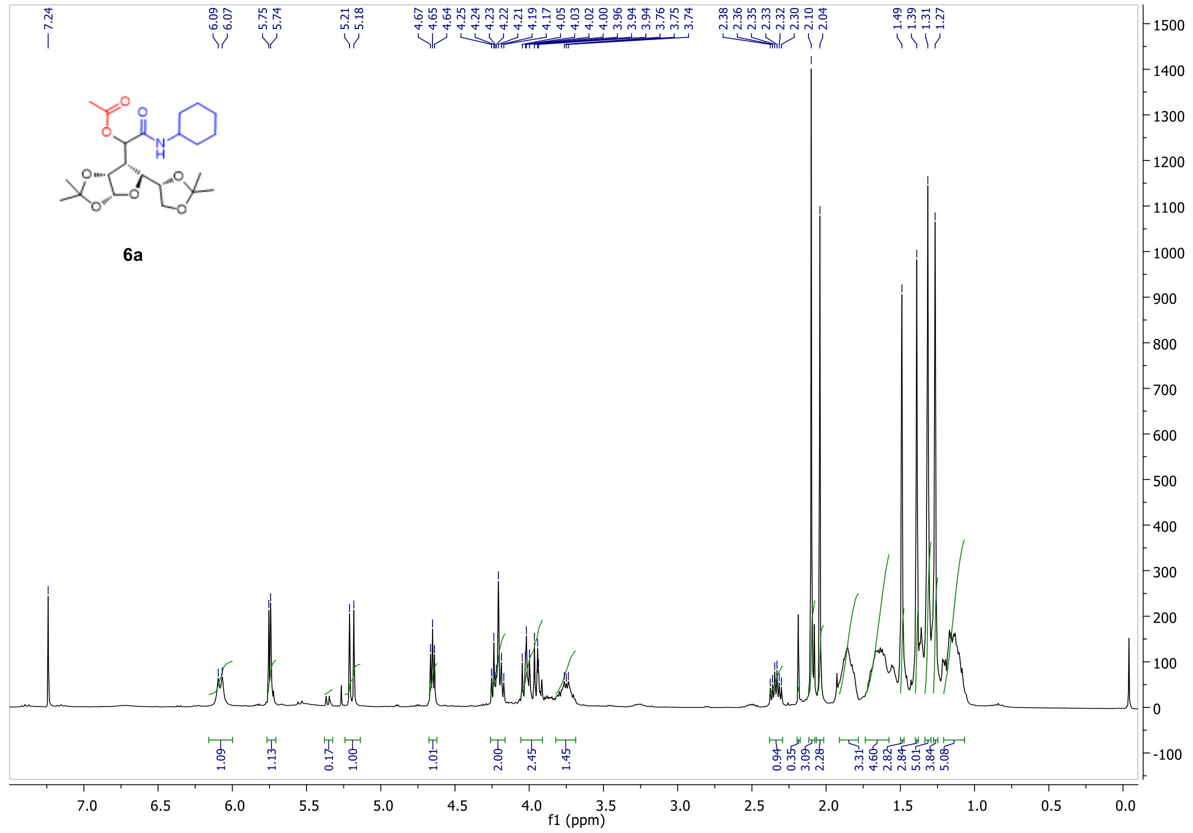

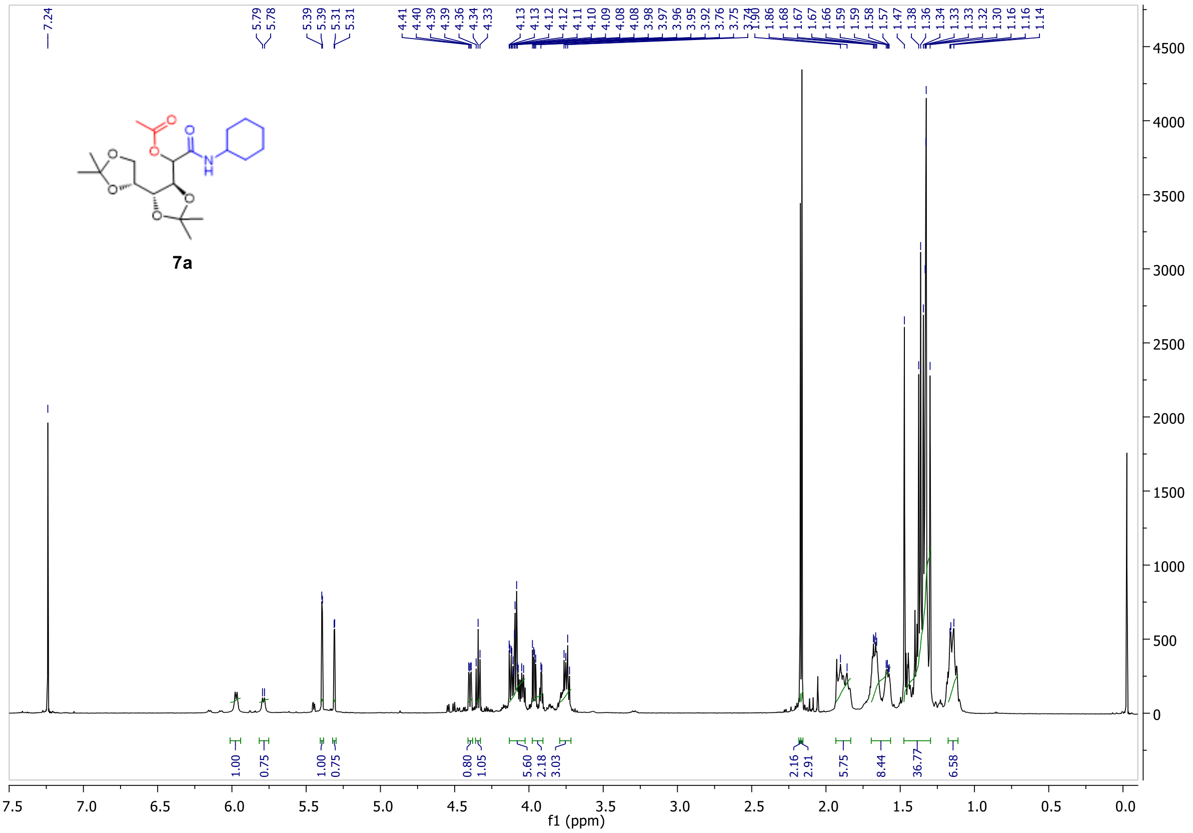

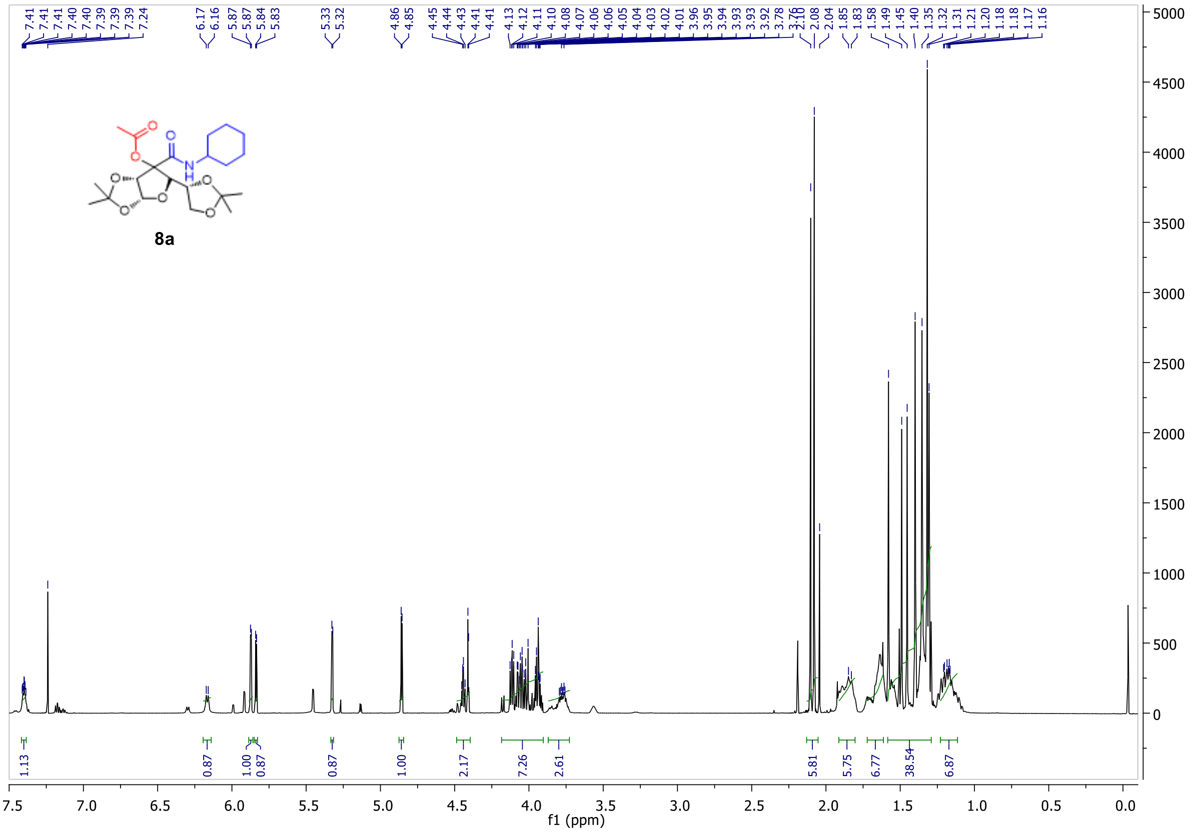

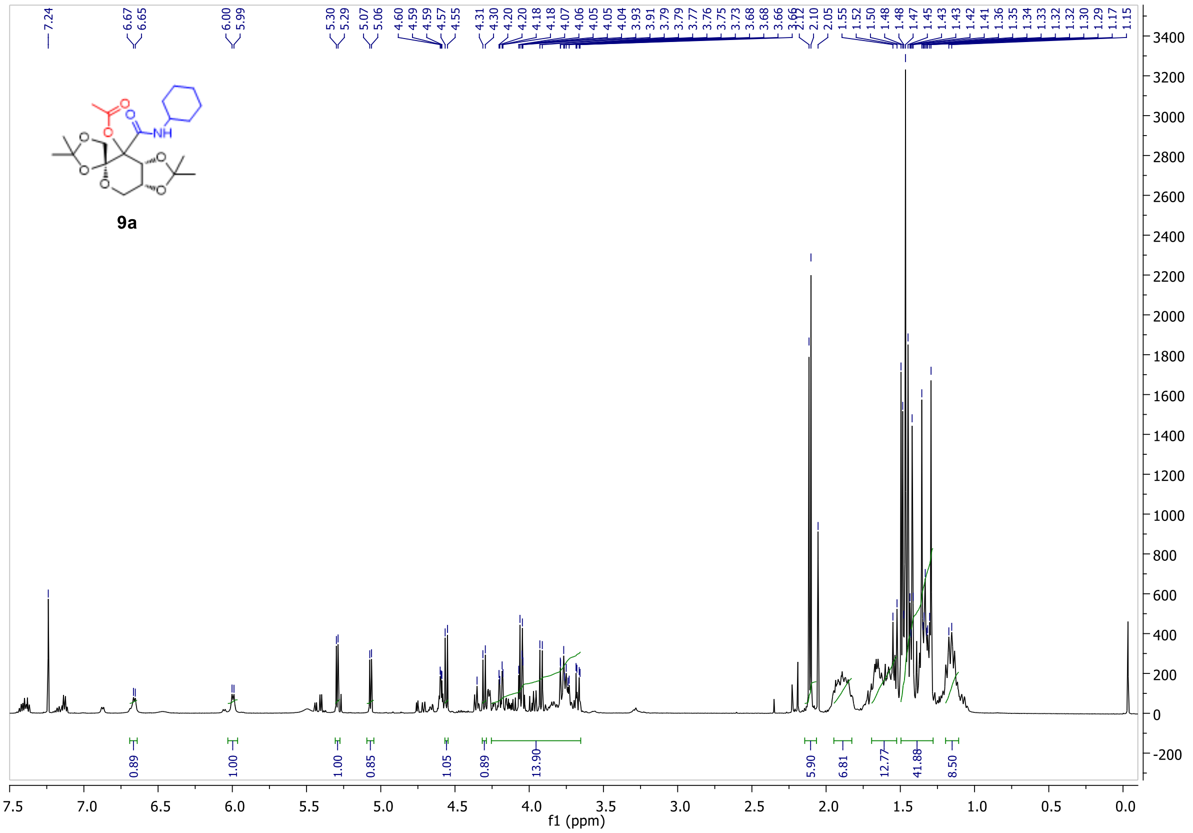
9a
f1 (ppm)

NMR and HRMS spectra of Passerini products obtained after purification:

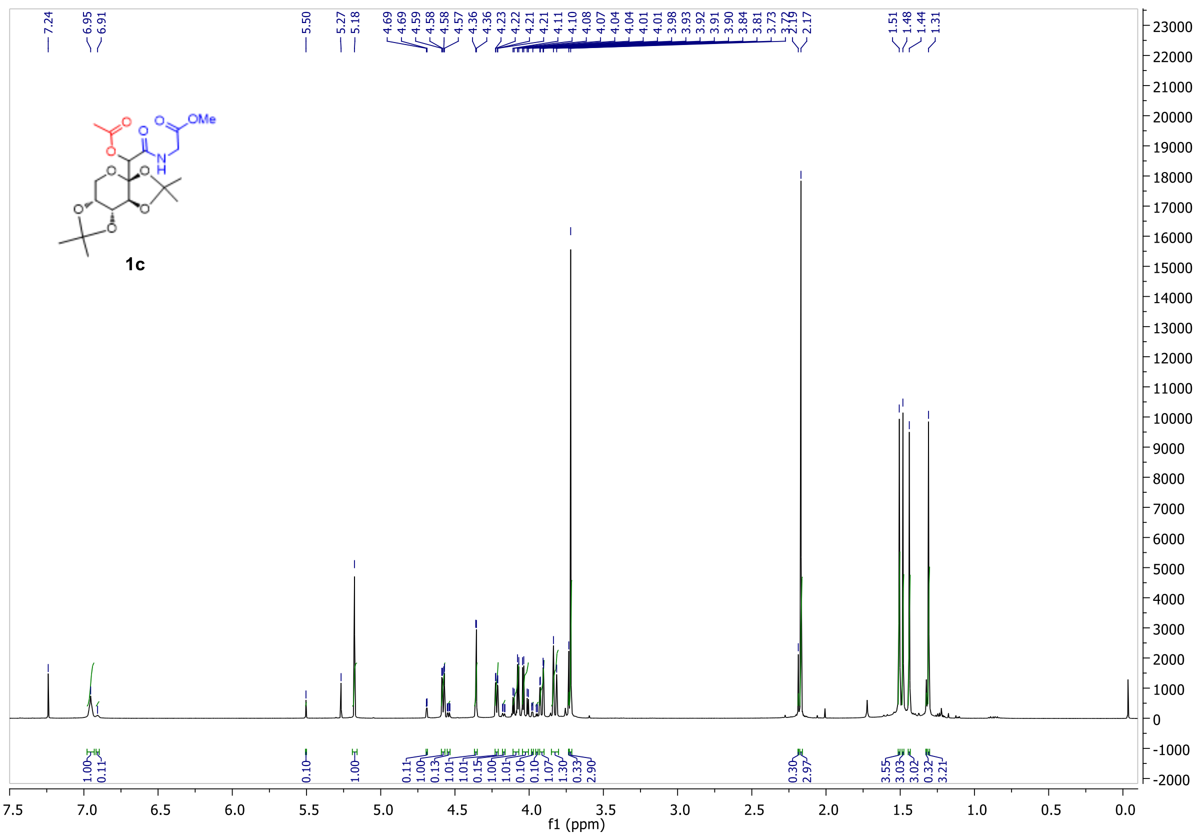

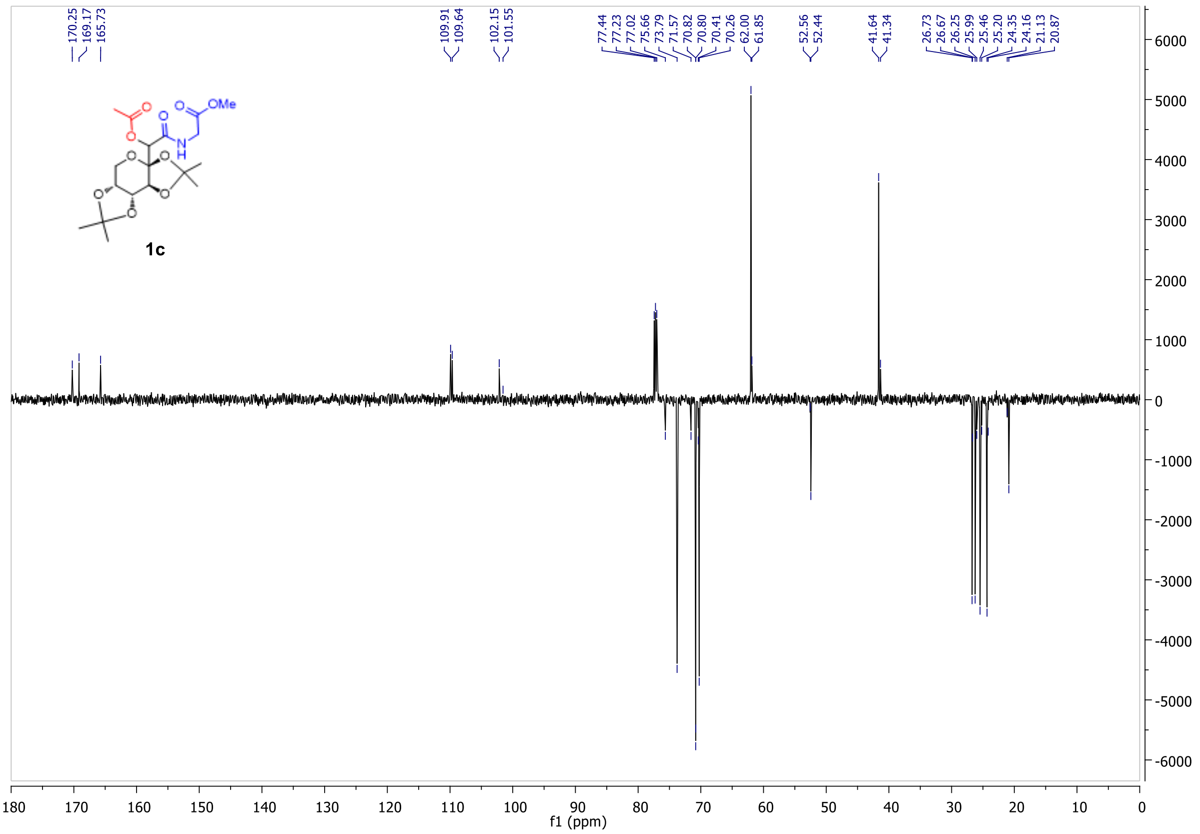

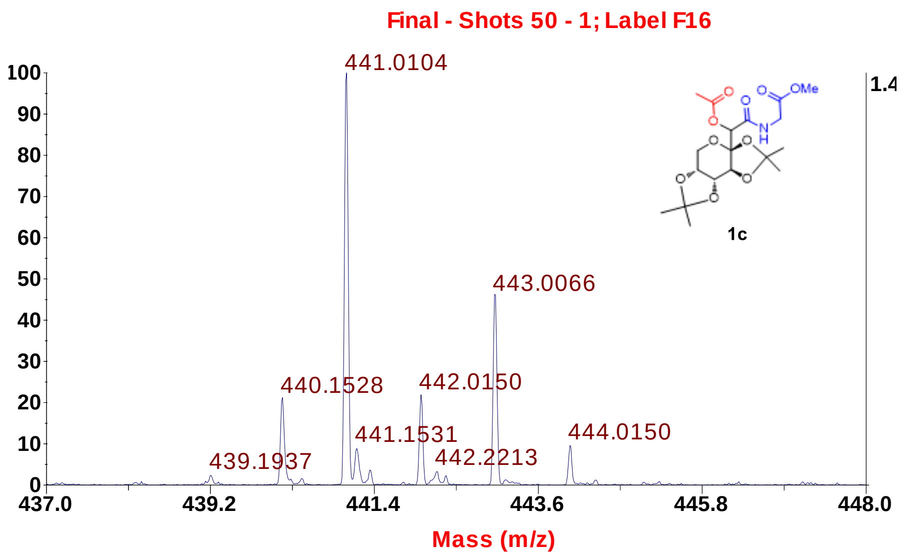
Final - Shots 50 - 1; Label F16
441.0104
443.0066
440.1528
442.0150
441.1531
444.0150
439.1937
442.2213
1.4
1c
Mass (m/z)
437.0
439.2
441.4
443.6
445.8
448.0

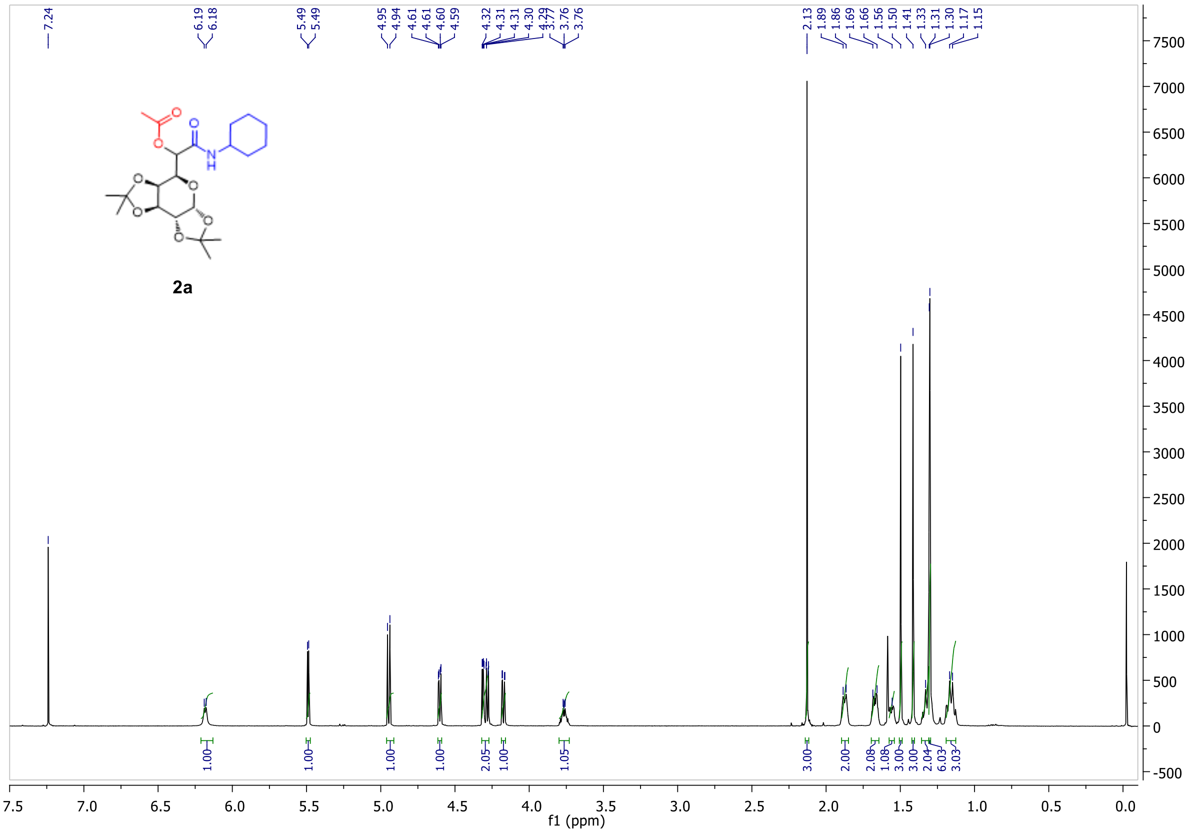
2a
f1 (ppm)

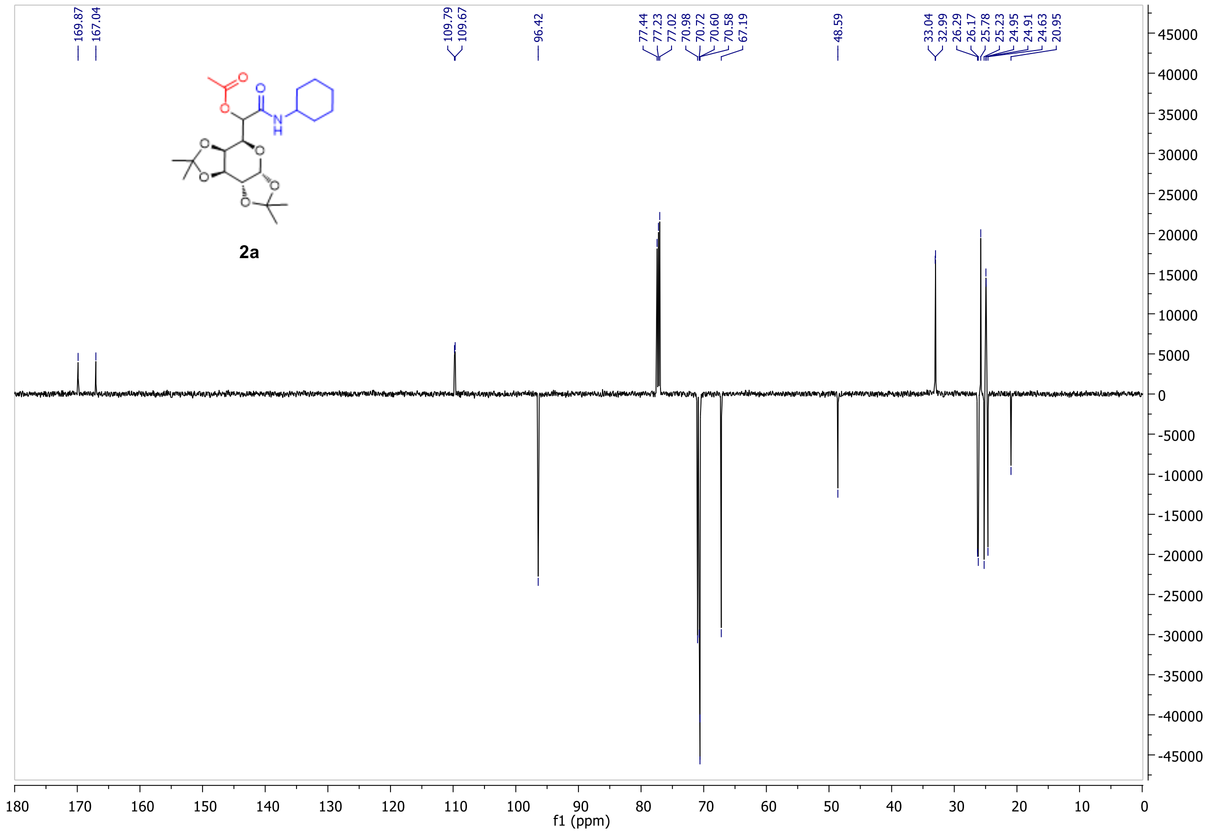
169.87
167.04
109.79
109.67
96.42
77.44
77.23
77.02
70.98
70.72
70.60
70.58
67.19
48.59
33.04
32.99
26.29
26.17
25.78
25.23
24.95
24.91
24.63
20.95
2a
f1 (ppm)

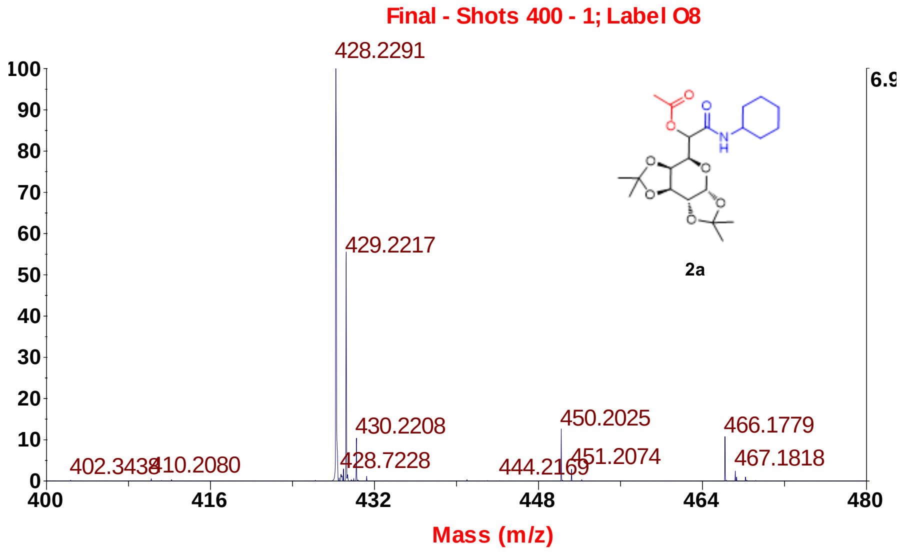
Final - Shots 400 - 1; Label O8
428.2291
429.2217
430.2208
428.7228
450.2025
451.2074
444.2169
466.1779
467.1818
402.3438
410.2080
2a
Mass (m/z)

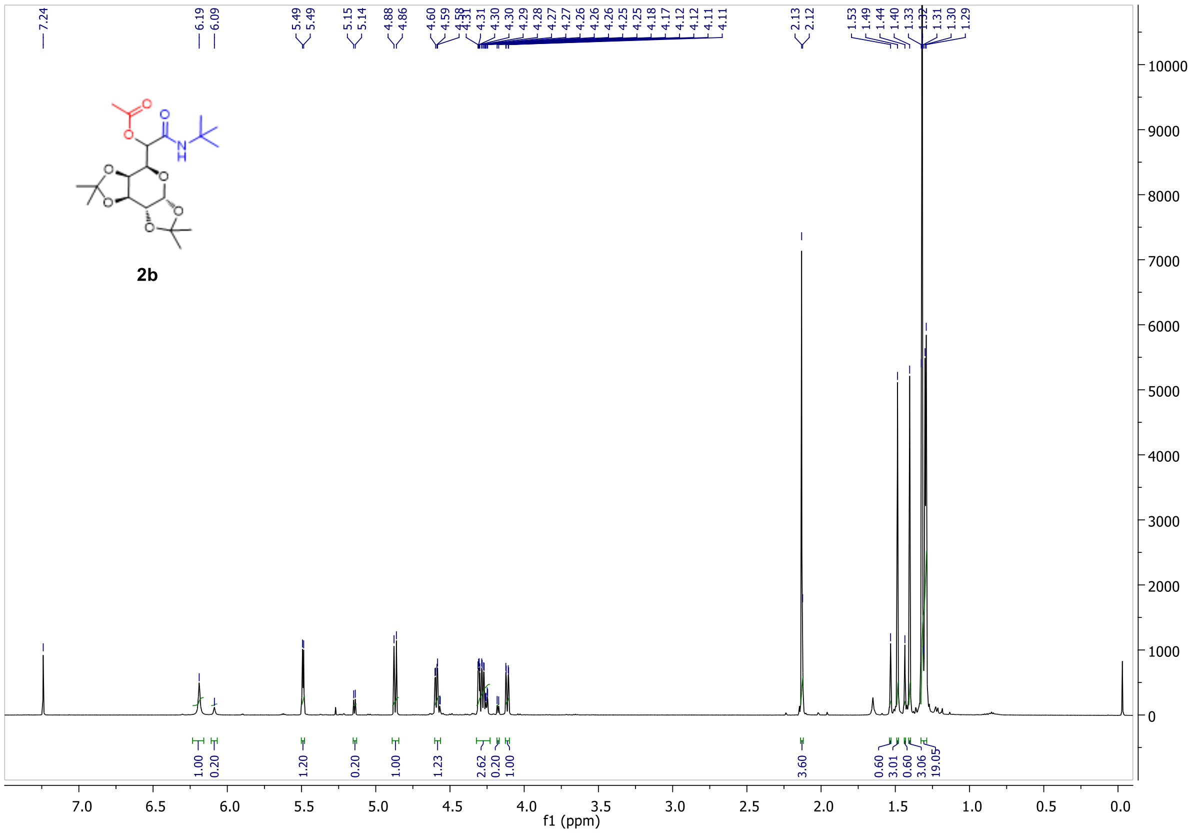
7.24
6.19
6.09
5.49
5.49
5.15
5.14
4.88
4.86
4.60
4.59
4.58
4.31
4.31
4.30
4.30
4.29
4.28
4.27
4.27
4.26
4.26
4.26
4.25
4.18
4.17
4.12
4.12
4.11
4.11
2.13
2.12
1.53
1.49
1.44
1.40
1.33
1.32
1.31
1.30
1.29
2b
1.00
0.20
1.20
0.20
1.00
1.23
2.62
0.20
1.00
3.60
0.60
3.01
0.60
3.06
19.05
f1 (ppm)

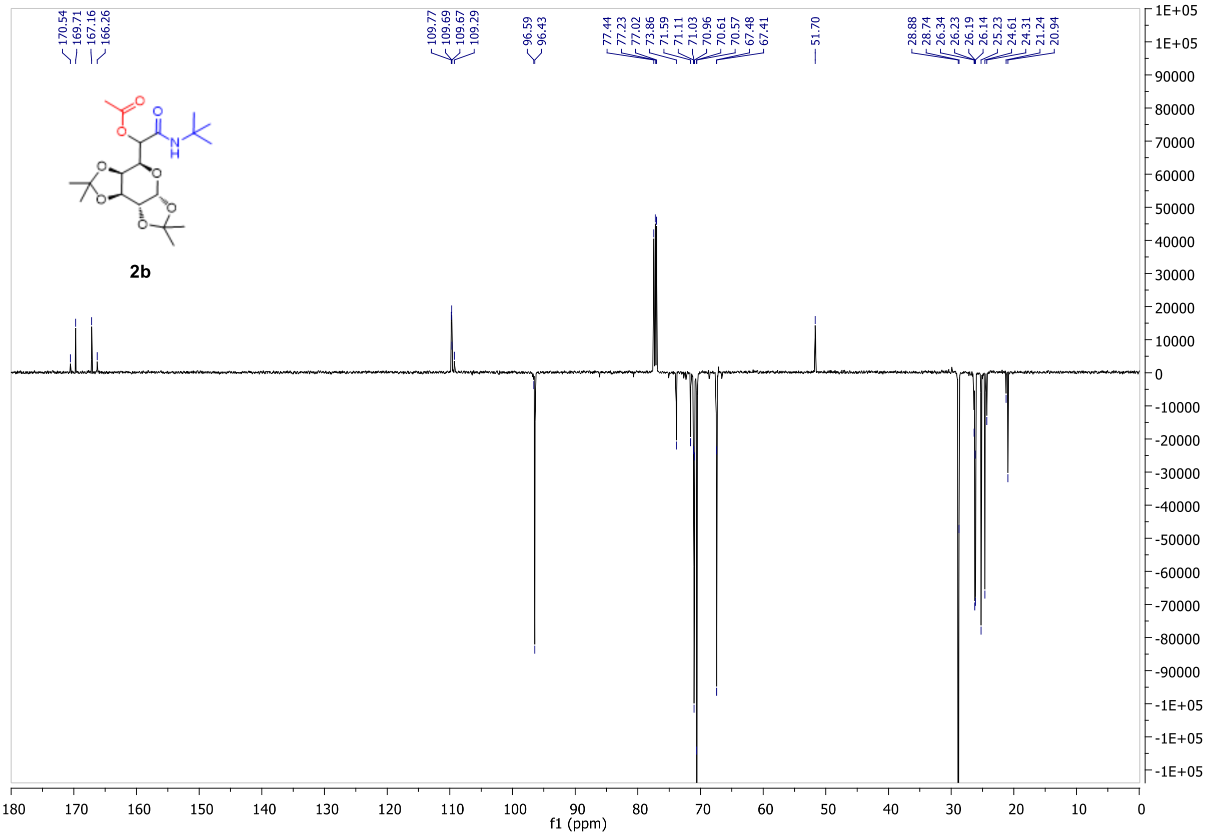
170.54
169.71
167.16
166.26
109.77
109.69
109.67
109.29
96.59
96.43
77.44
77.23
77.02
73.86
71.59
71.11
71.03
70.96
70.61
70.57
67.48
67.41
51.70
28.88
28.74
26.34
26.23
26.19
26.14
25.23
24.61
24.31
21.24
20.94
2b
f1 (ppm)

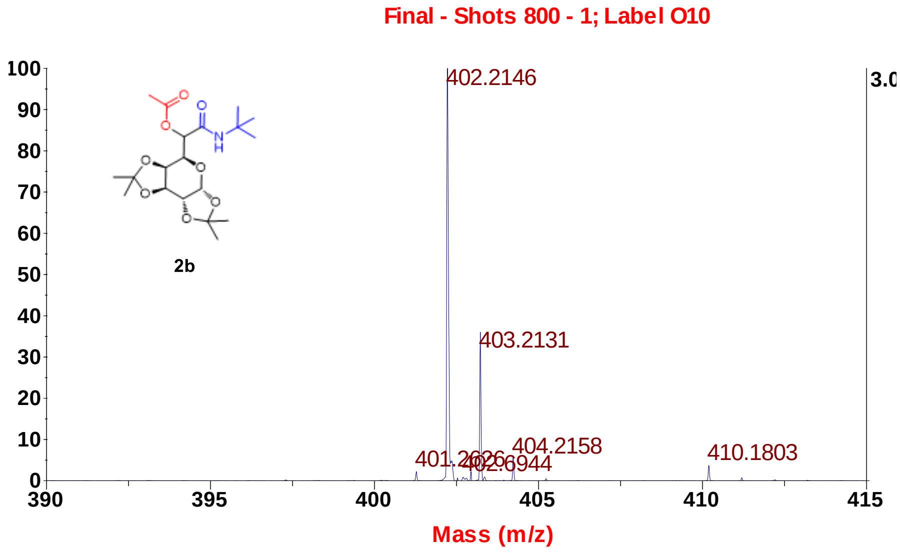
Final - Shots 800 - 1; Label O10
402.2146
403.2131
401.2626
402.6944
404.2158
410.1803
3.0
2b
Mass (m/z)

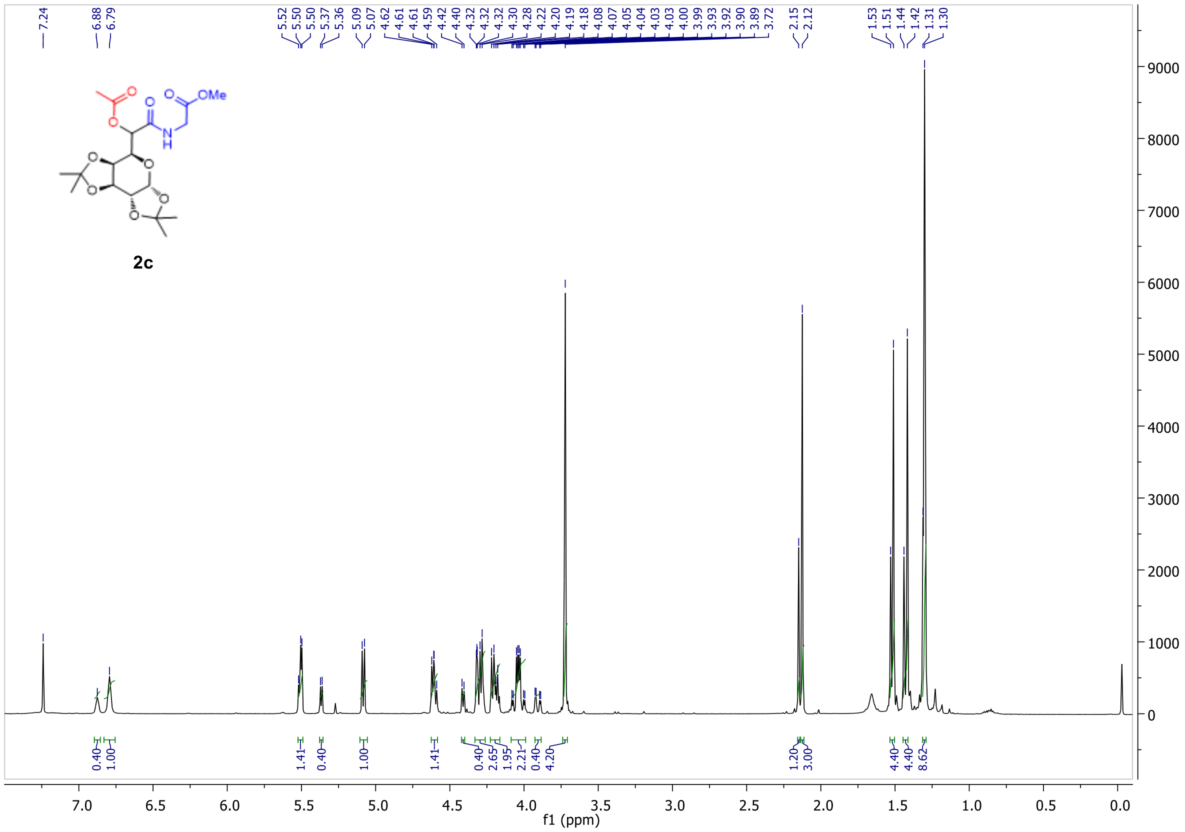
7.24
6.88
6.79
5.52
5.50
5.50
5.37
5.36
5.09
5.07
4.62
4.61
4.61
4.59
4.42
4.40
4.32
4.32
4.32
4.30
4.28
4.22
4.20
4.19
4.18
4.08
4.07
4.05
4.04
4.03
4.03
4.00
3.99
3.93
3.92
3.90
3.89
3.72
2.15
2.12
1.53
1.51
1.44
1.42
1.31
1.30
OMe
2c
0.40
1.00
1.41
0.40
1.00
1.41
0.40
2.65
1.95
2.21
0.40
4.20
1.20
3.00
4.40
4.40
8.62
f1 (ppm)

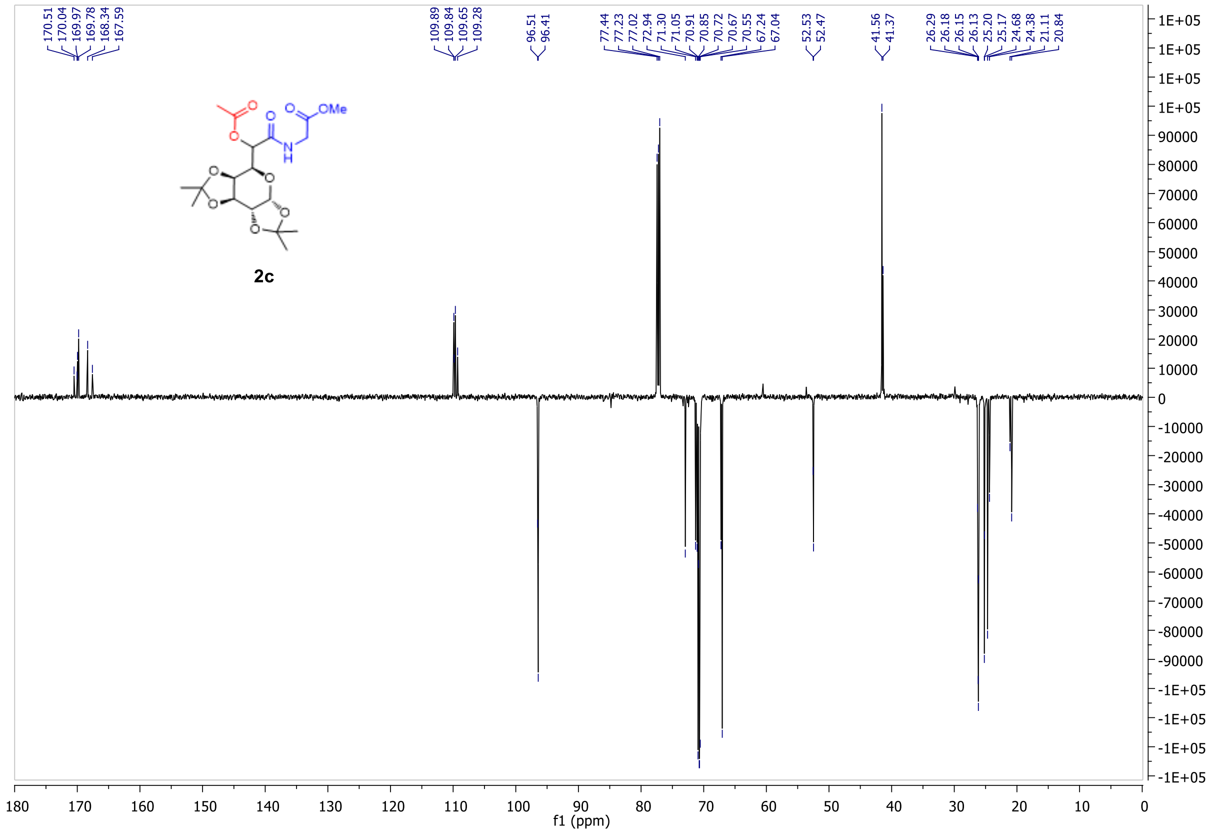

Final - Shots 400 - 1; Label O9

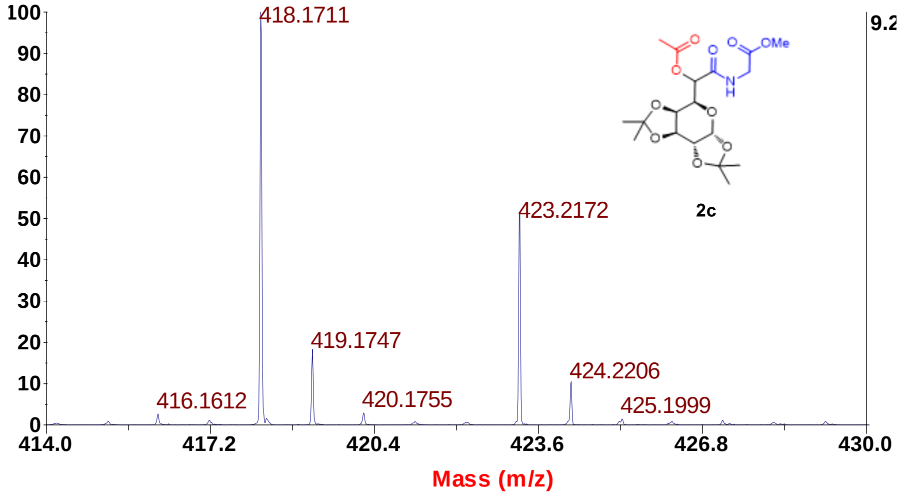

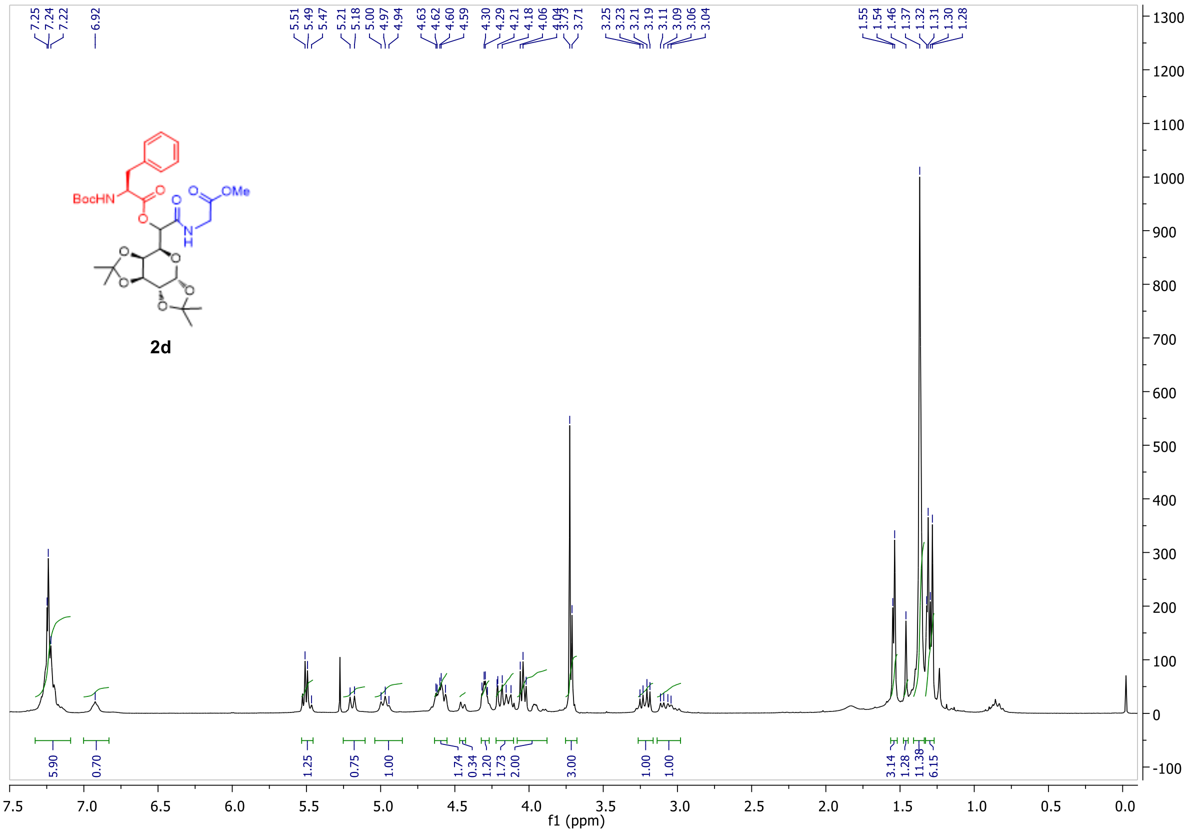
2d
BocHN
OMe
f1 (ppm)

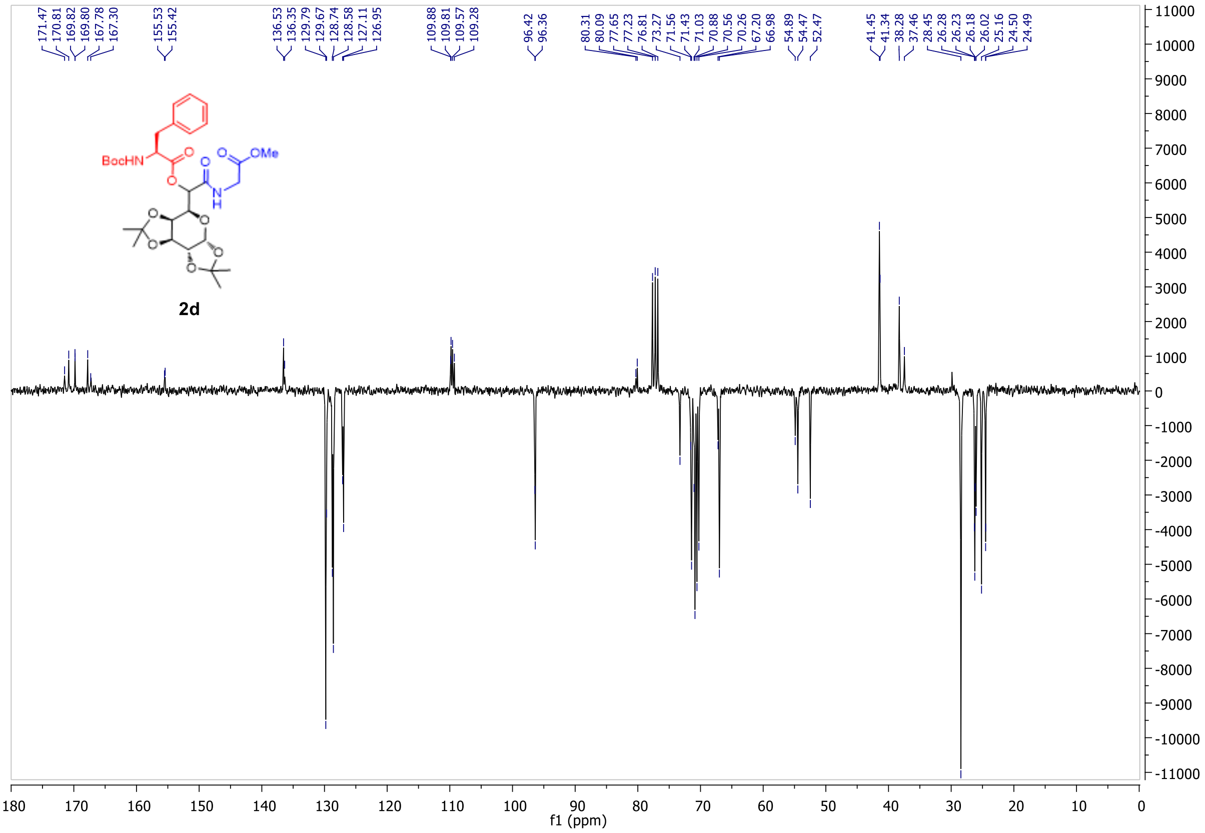
2d
BocHN
OMe
f1 (ppm)

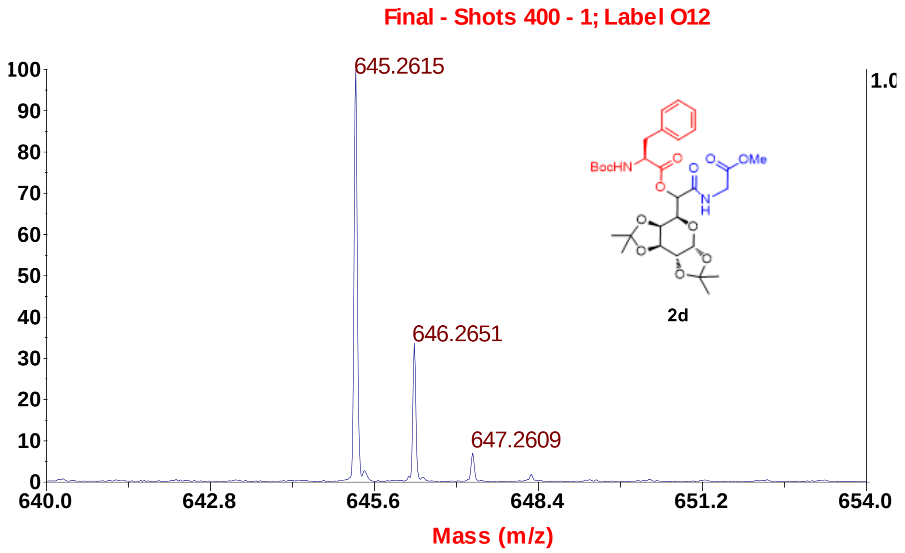

Final - Shots 400 - 1; Label O12
645.2615
646.2651
647.2609
BocHN
OMe
2d
Mass (m/z)

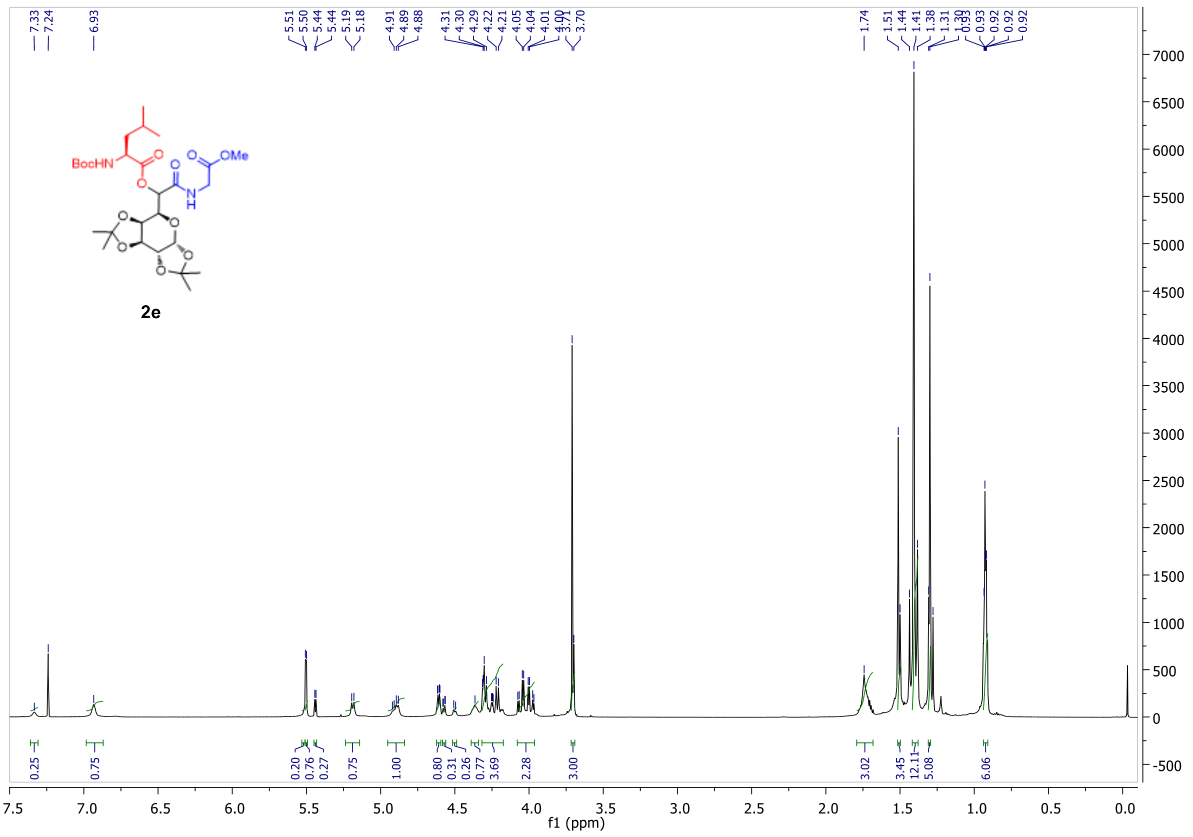

BocHN
OMe
2e
f1 (ppm)

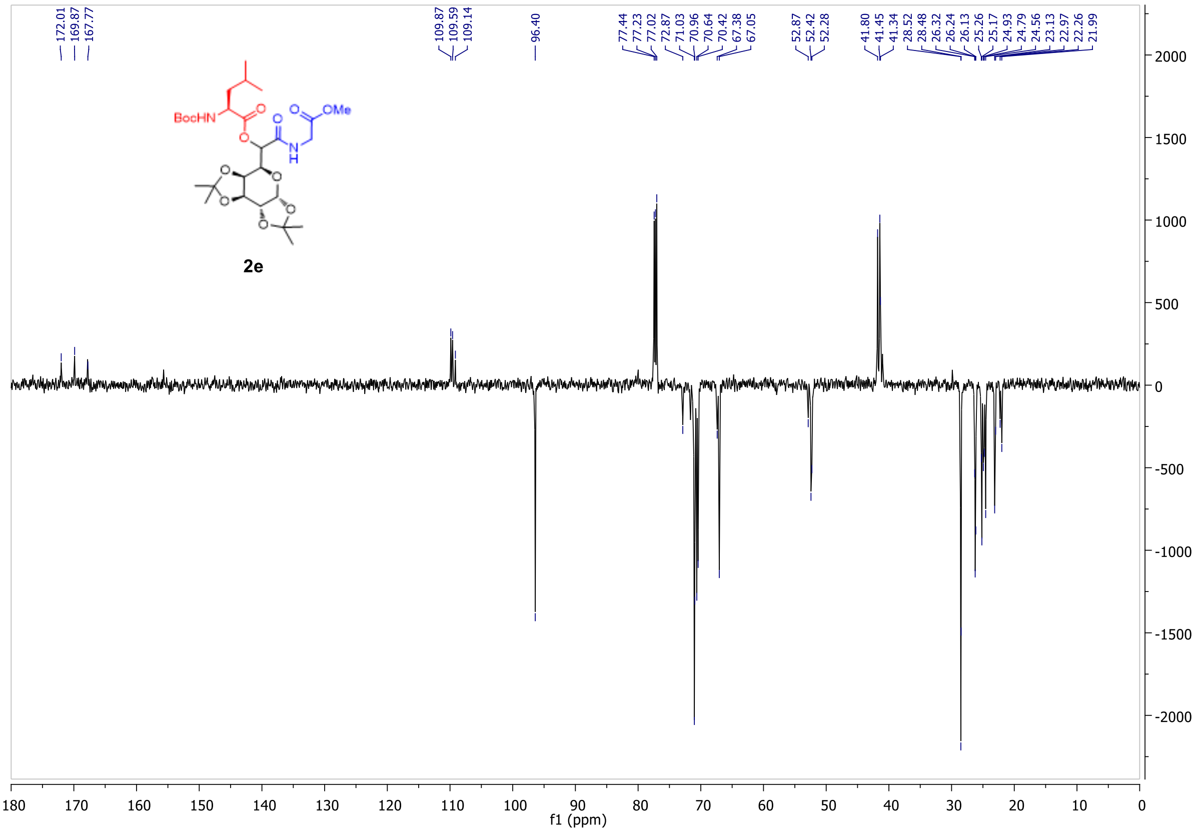
172.01
169.87
167.77
109.87
109.59
109.14
96.40
77.44
77.23
77.02
72.87
71.03
70.96
70.64
70.42
67.38
67.05
52.87
52.42
52.28
41.80
41.45
41.34
28.52
28.48
26.32
26.24
26.13
25.26
25.17
24.93
24.79
24.56
23.13
22.97
22.26
21.99
BocHN
O
O
OMe
N
H
2e
f1 (ppm)

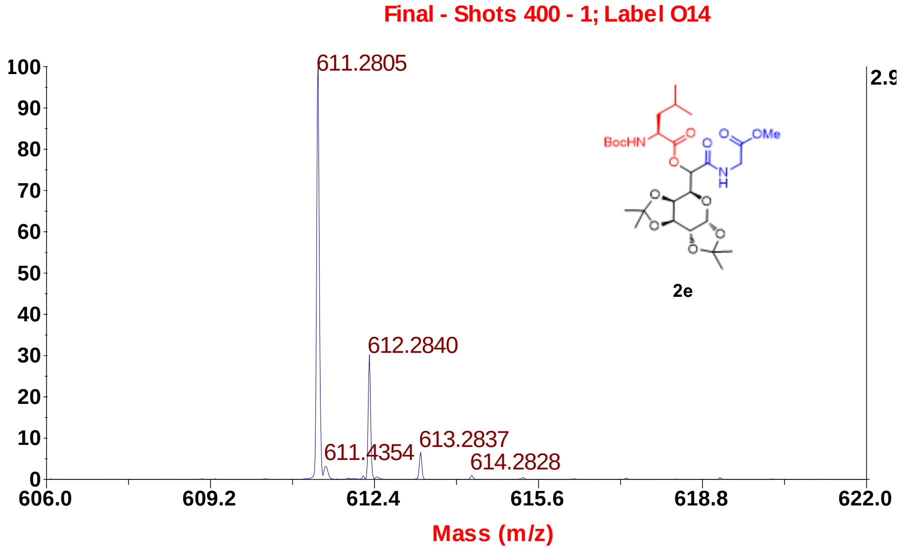
Final - Shots 400 - 1; Label O14
611.2805
612.2840
611.4354
613.2837
614.2828
2.9
BocHN
OMe
2e
Mass (m/z)

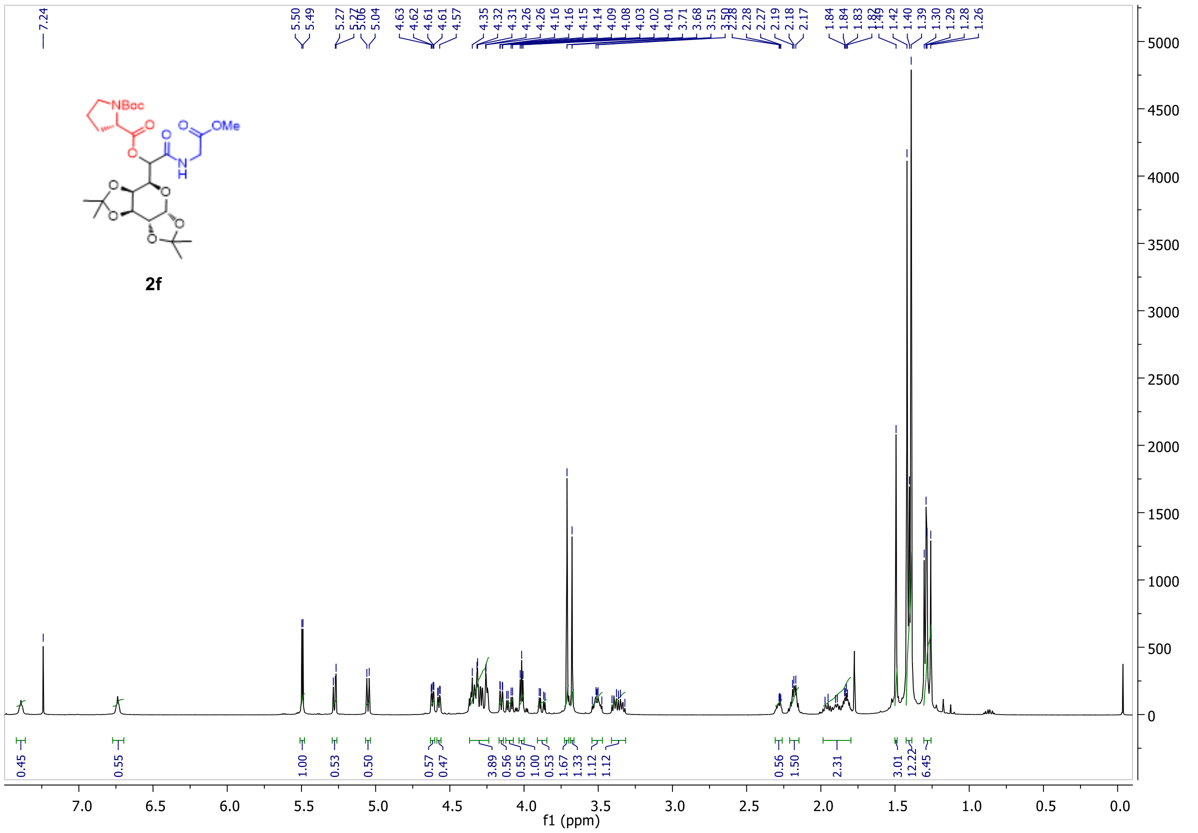
NBoc
OMe
2f
f1 (ppm)

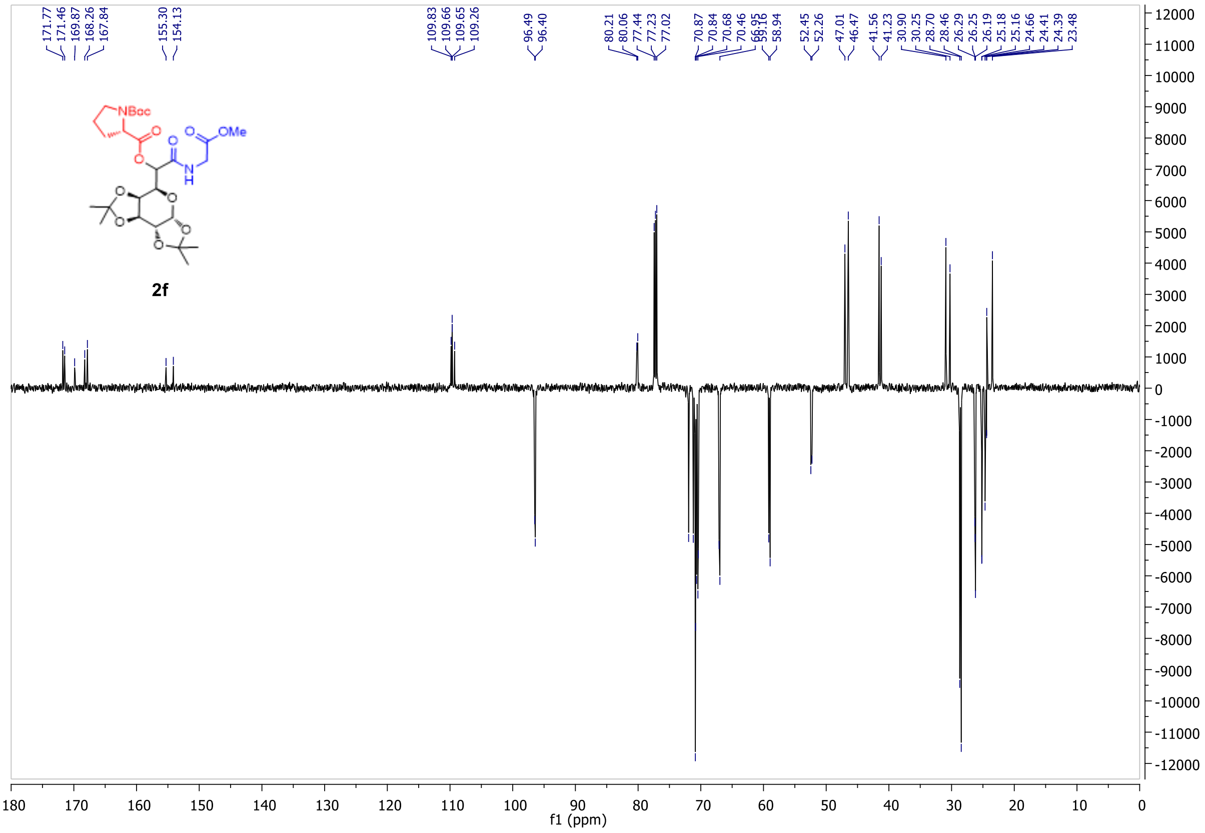
NBoc
OMe
2f
f1 (ppm)

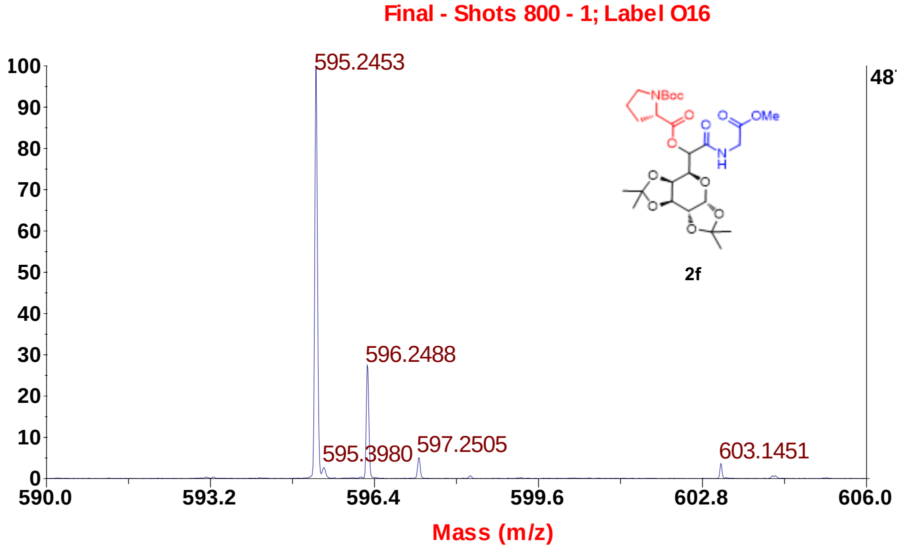
Final - Shots 800 - 1; Label O16
595.2453
596.2488
595.3980
597.2505
603.1451
2f
Mass (m/z)

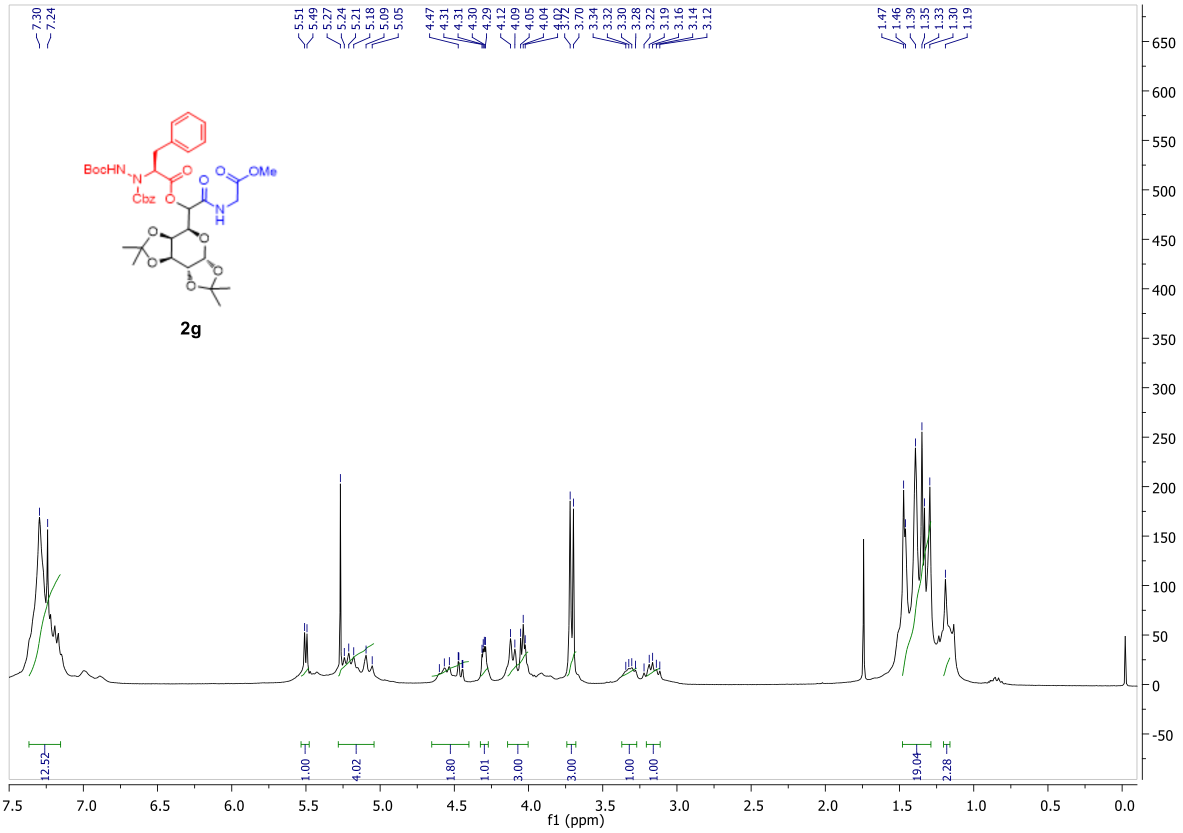
2g
f1 (ppm)

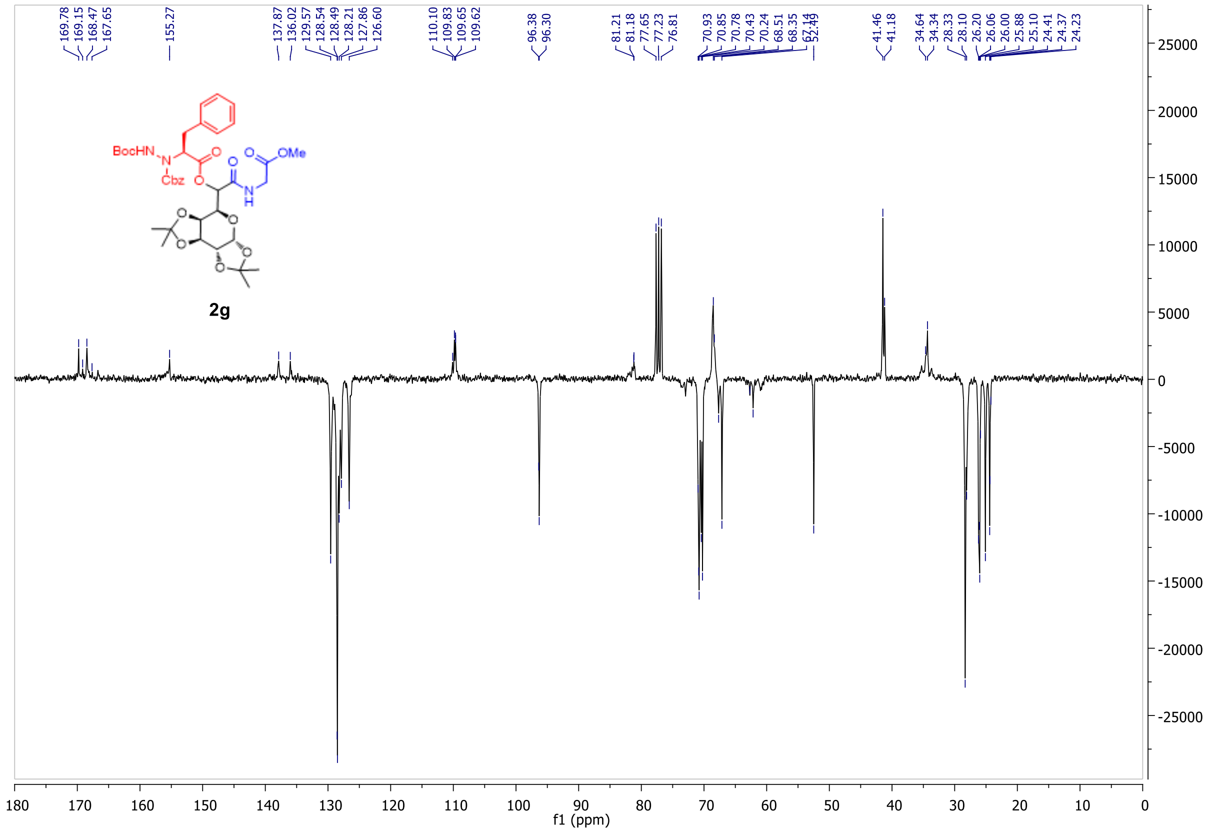
169.78
169.15
168.47
167.65
155.27
137.87
136.02
129.57
128.54
128.49
128.21
127.86
126.60
110.10
109.83
109.65
109.62
96.38
96.30
81.21
81.18
77.65
77.23
76.81
70.93
70.85
70.78
70.43
70.24
68.51
68.35
41.46
41.18
34.64
34.34
28.33
28.10
26.20
26.06
26.00
25.88
25.10
24.41
24.37
24.23
BocHN
N
Cbz
O
O
O
OMe
N
H
2g
f1 (ppm)

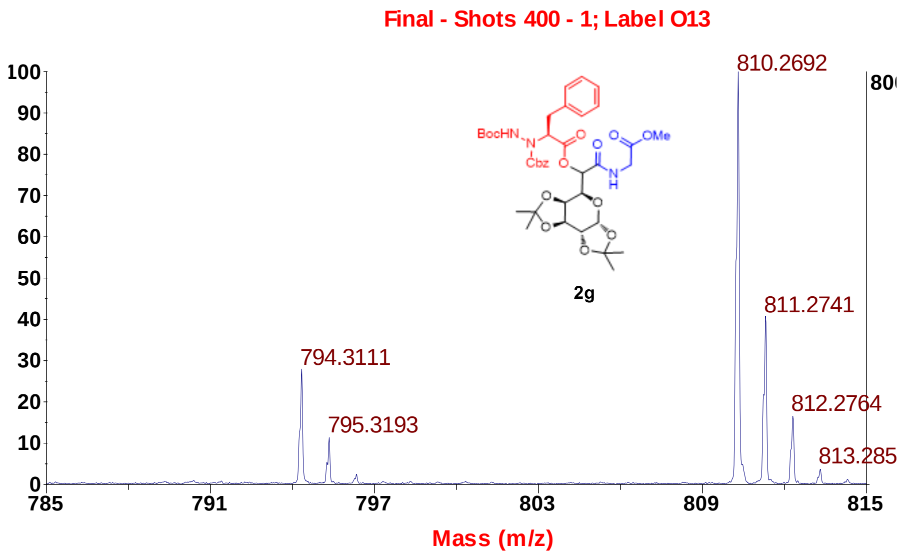
Final - Shots 400 - 1; Label O13
810.2692
811.2741
812.2764
813.285
794.3111
795.3193
BocHN
N
Cbz
O
OMe
H
2g
Mass (m/z)

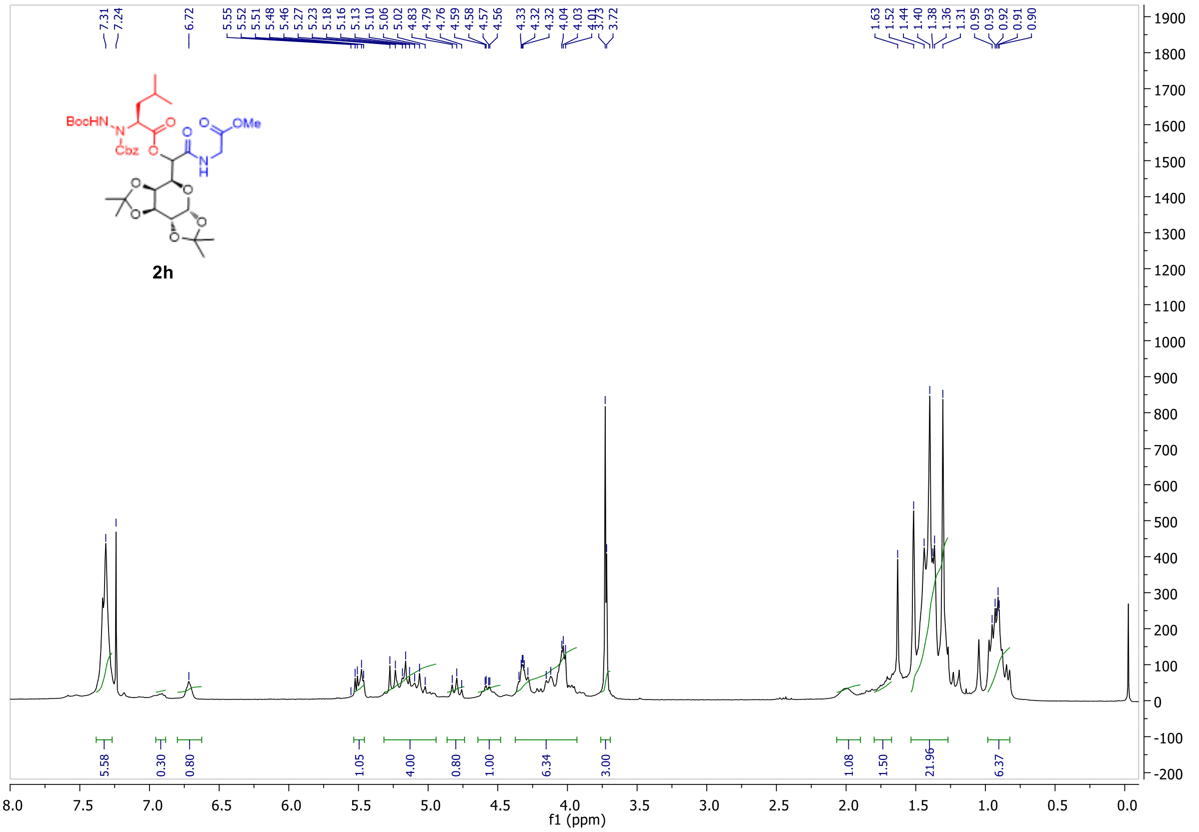
7.31
7.24
6.72
5.55
5.52
5.51
5.48
5.46
5.27
5.23
5.18
5.16
5.13
5.10
5.06
5.02
4.83
4.79
4.76
4.59
4.58
4.57
4.56
4.33
4.32
4.32
4.04
4.03
3.73
3.72
1.63
1.52
1.44
1.40
1.38
1.36
1.31
0.95
0.93
0.92
0.91
0.90
BocHN
N
Cbz
O
O
O
O
OMe
N
H
O
O
O
O
O
2h
5.58
0.30
0.80
1.05
4.00
0.80
1.00
6.34
3.00
1.08
1.50
21.96
6.37
f1 (ppm)

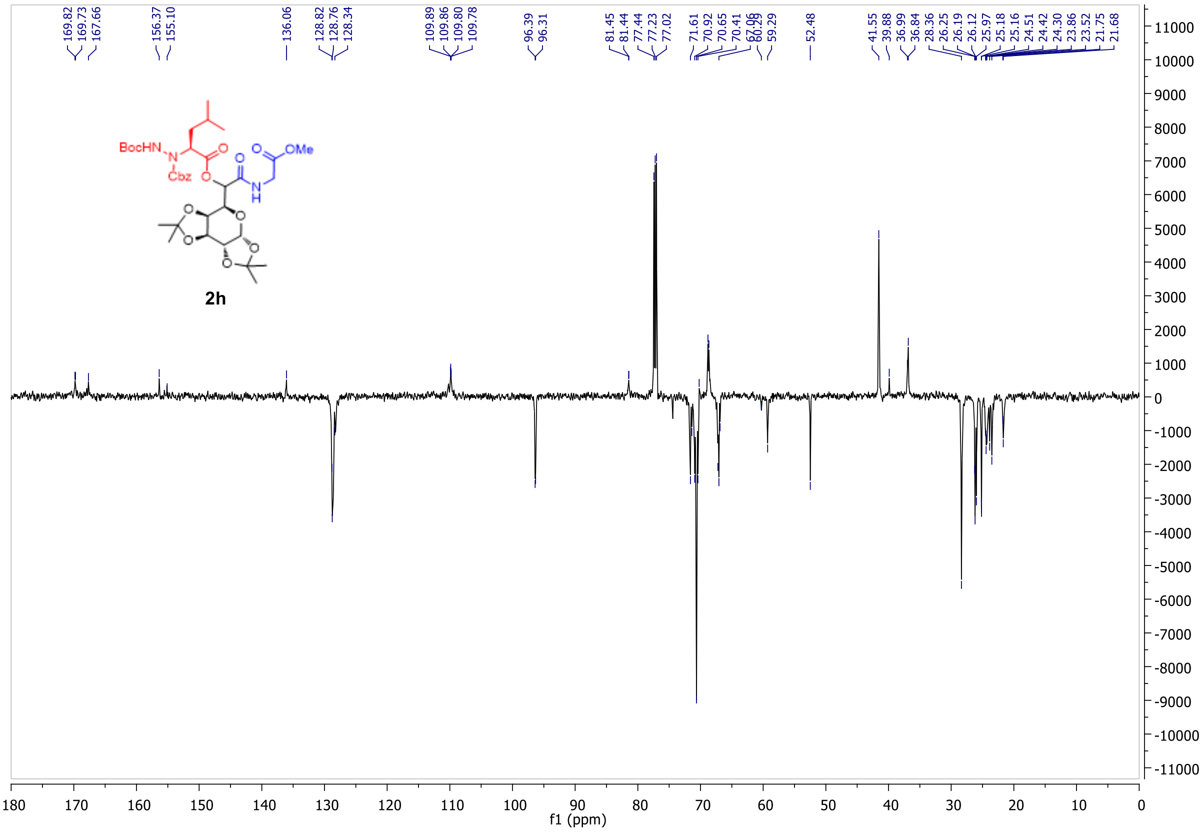
169.82
169.73
167.66
156.37
155.10
136.06
128.82
128.76
128.34
109.89
109.86
109.80
109.78
96.39
96.31
81.45
81.44
77.44
77.23
77.02
71.61
70.92
70.65
70.41
59.29
52.48
41.55
39.88
36.99
36.84
28.36
26.25
26.19
26.12
25.97
25.18
25.16
24.51
24.42
24.30
23.86
23.52
21.75
21.68
BocHN
N
Cbz
O
O
O
O
OMe
N
H
2h
f1 (ppm)

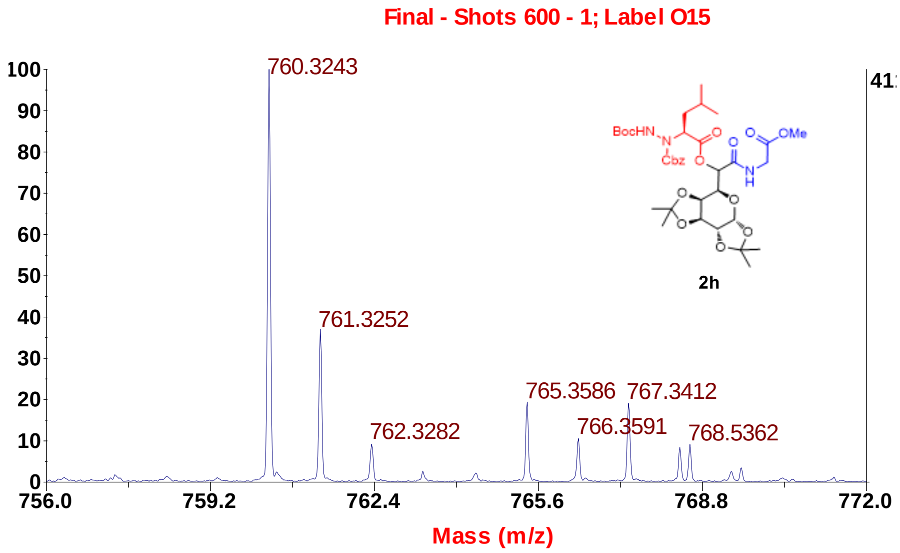
Final - Shots 600 - 1; Label O15
760.3243
761.3252
762.3282
765.3586
767.3412
766.3591
768.5362
Mass (m/z)
BocHN
Cbz
OMe
2h

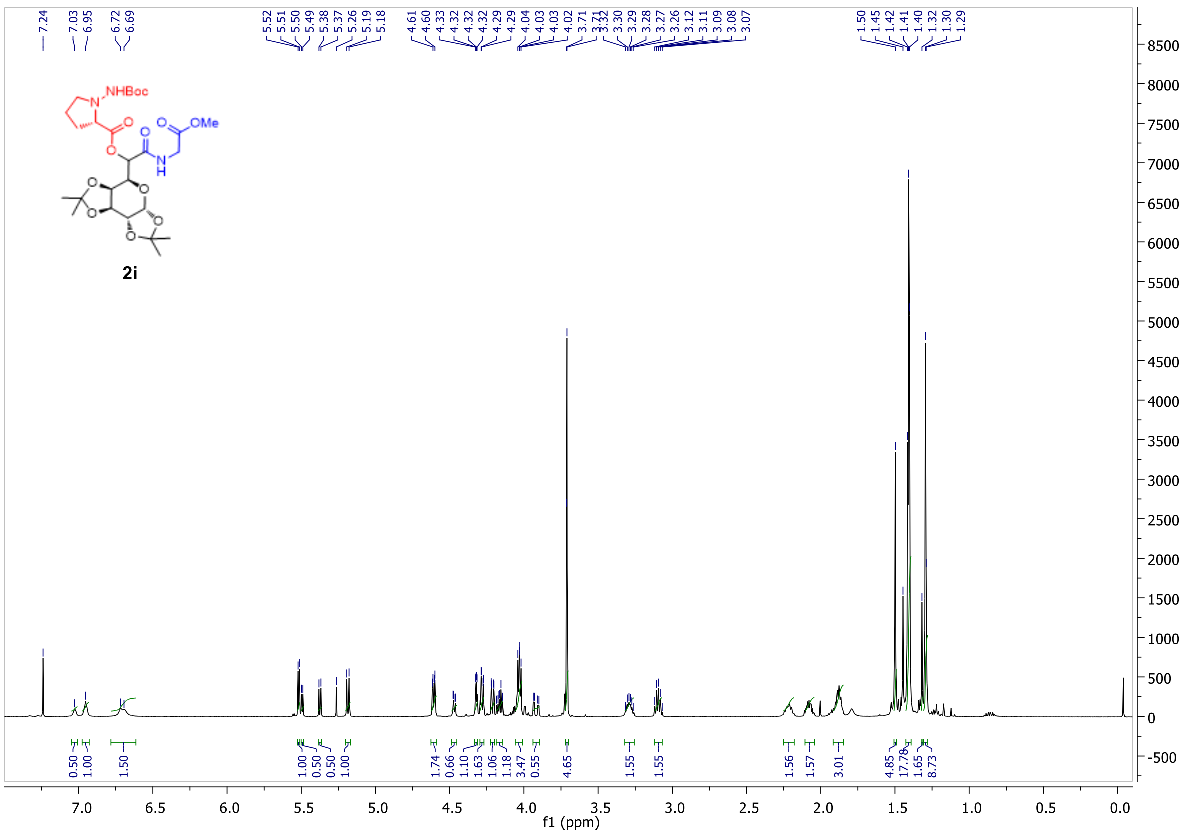
NHBoc
OMe
2i
f1 (ppm)

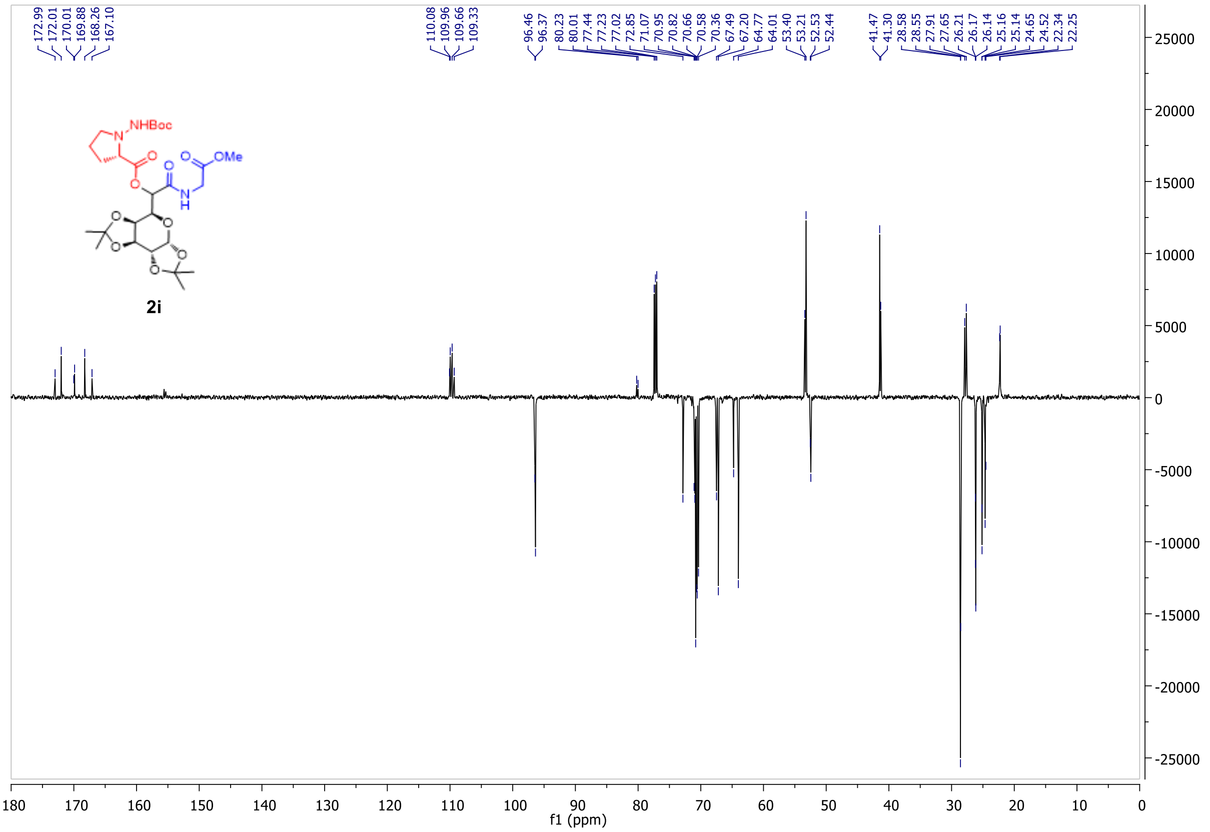
172.99
172.01
170.01
169.88
168.26
167.10
110.08
109.96
109.66
109.33
96.46
96.37
80.23
80.01
77.44
77.23
77.02
72.85
71.07
70.95
70.82
70.66
70.58
70.36
67.49
67.20
64.77
64.01
53.40
53.21
52.53
52.44
41.47
41.30
28.58
28.55
27.91
27.65
26.21
26.17
26.14
25.16
25.14
24.65
24.52
22.34
22.25
NHBoc
OMe
2i
f1 (ppm)

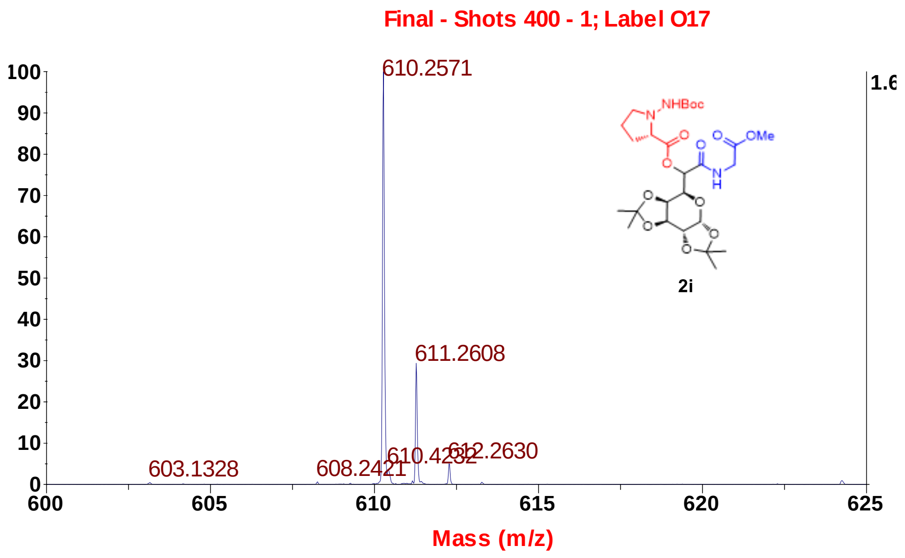
Final - Shots 400 - 1; Label O17
610.2571
611.2608
603.1328
608.2421
610.4232
612.2630
NHBoc
OMe
2i
Mass (m/z)

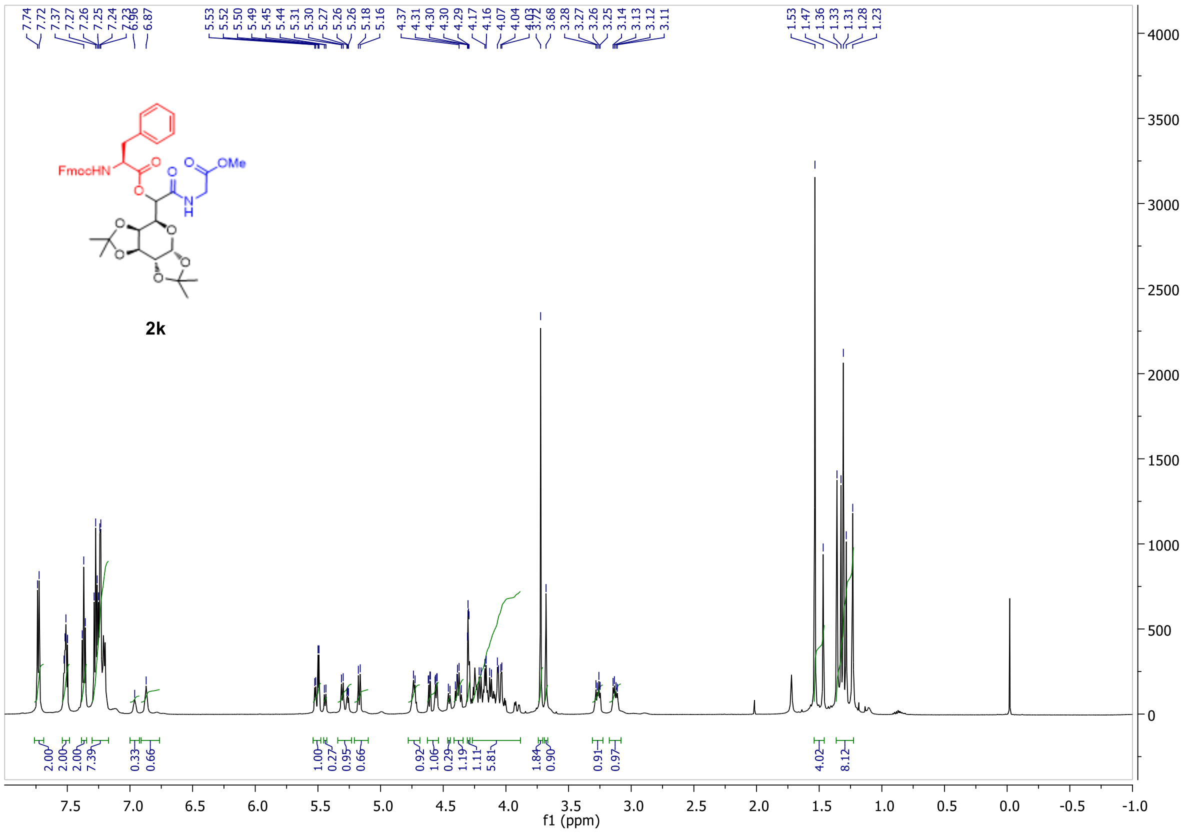

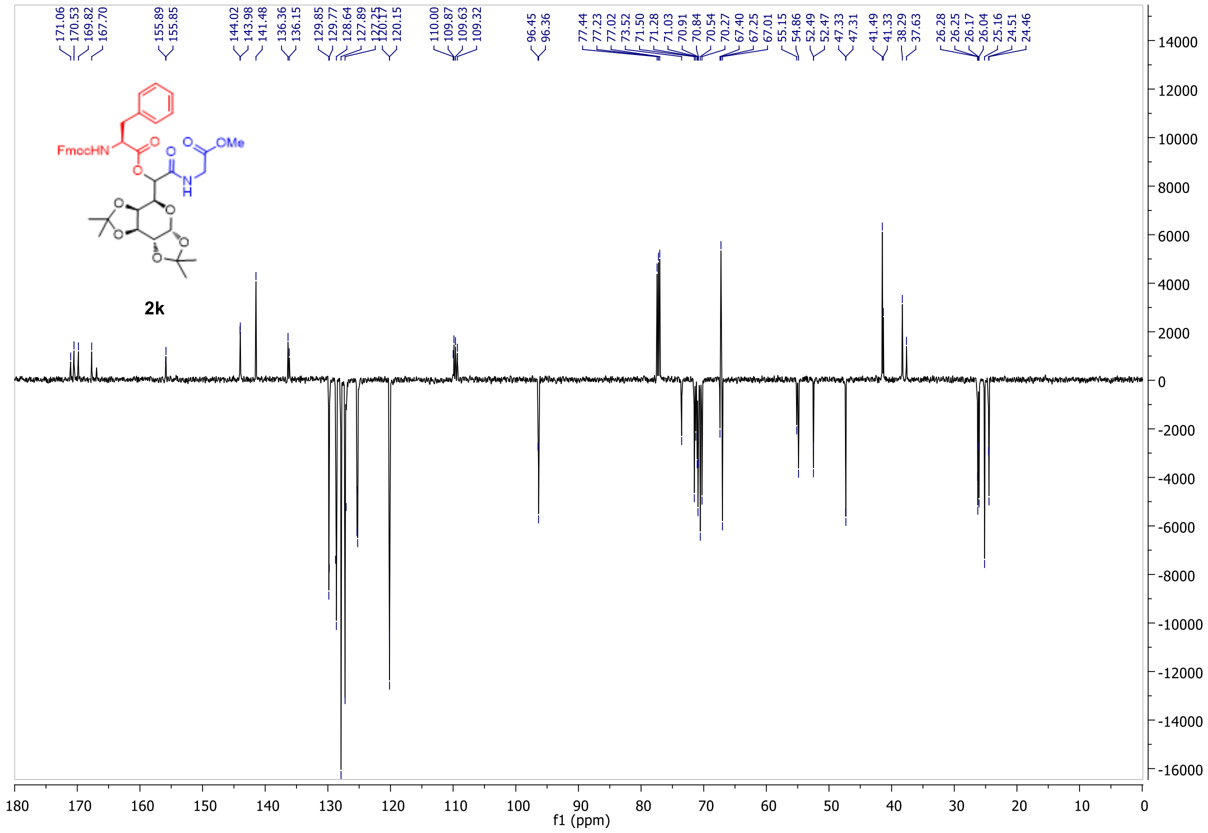

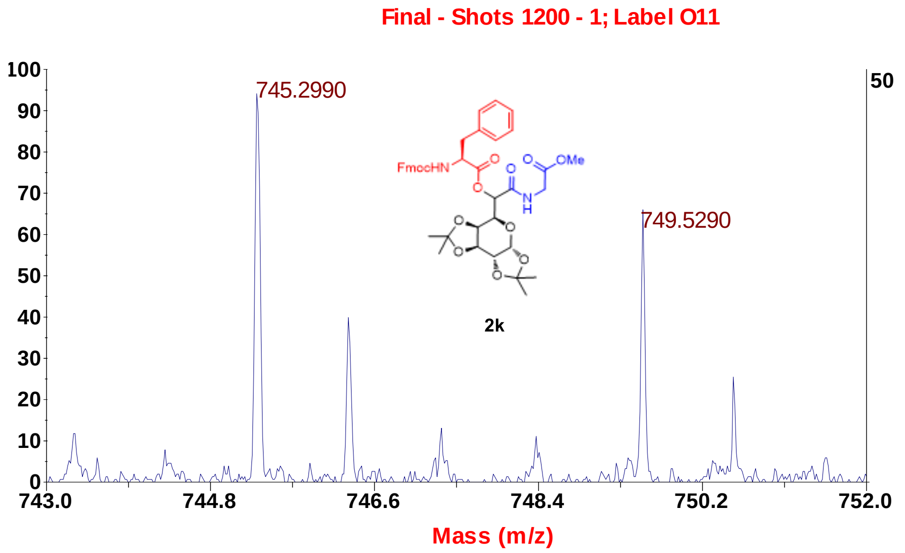

Final - Shots 1200 - 1; Label O11
745.2990
749.5290
FmocHN
OMe
2k
Mass (m/z)

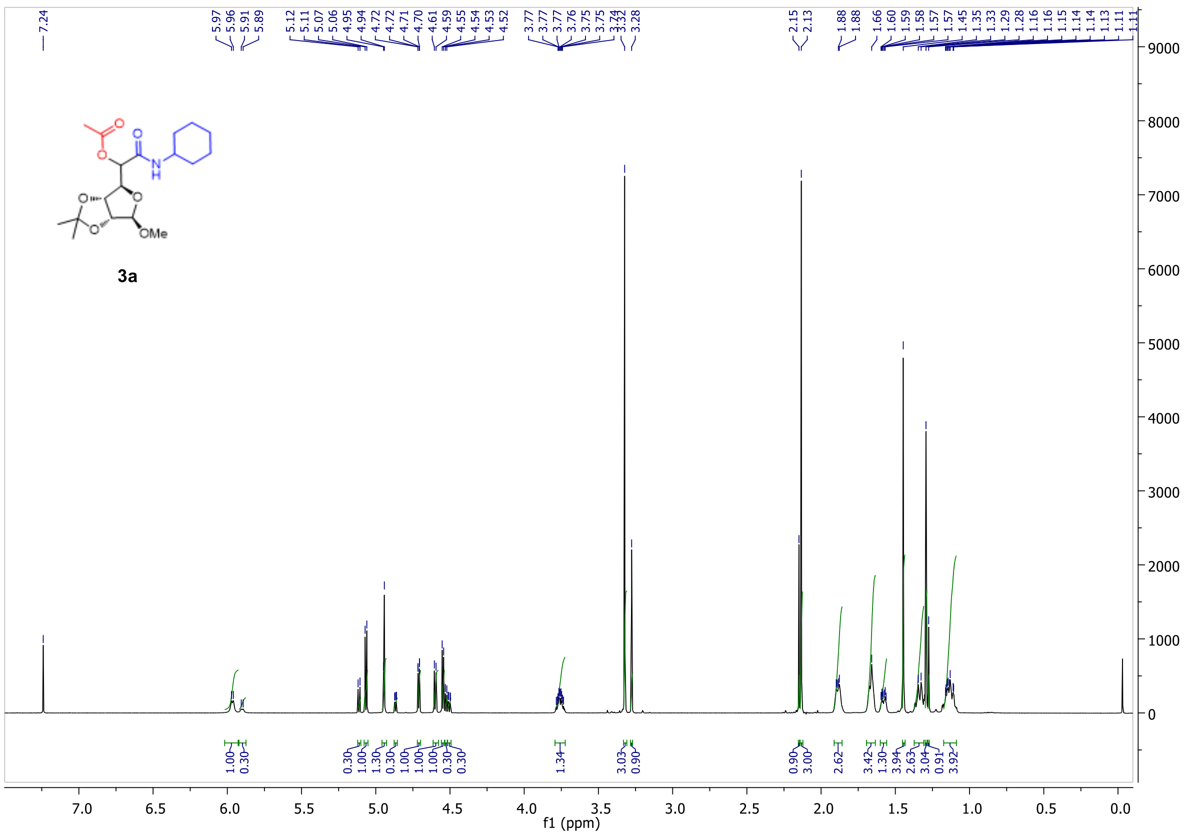

OMe
3a
f1 (ppm)

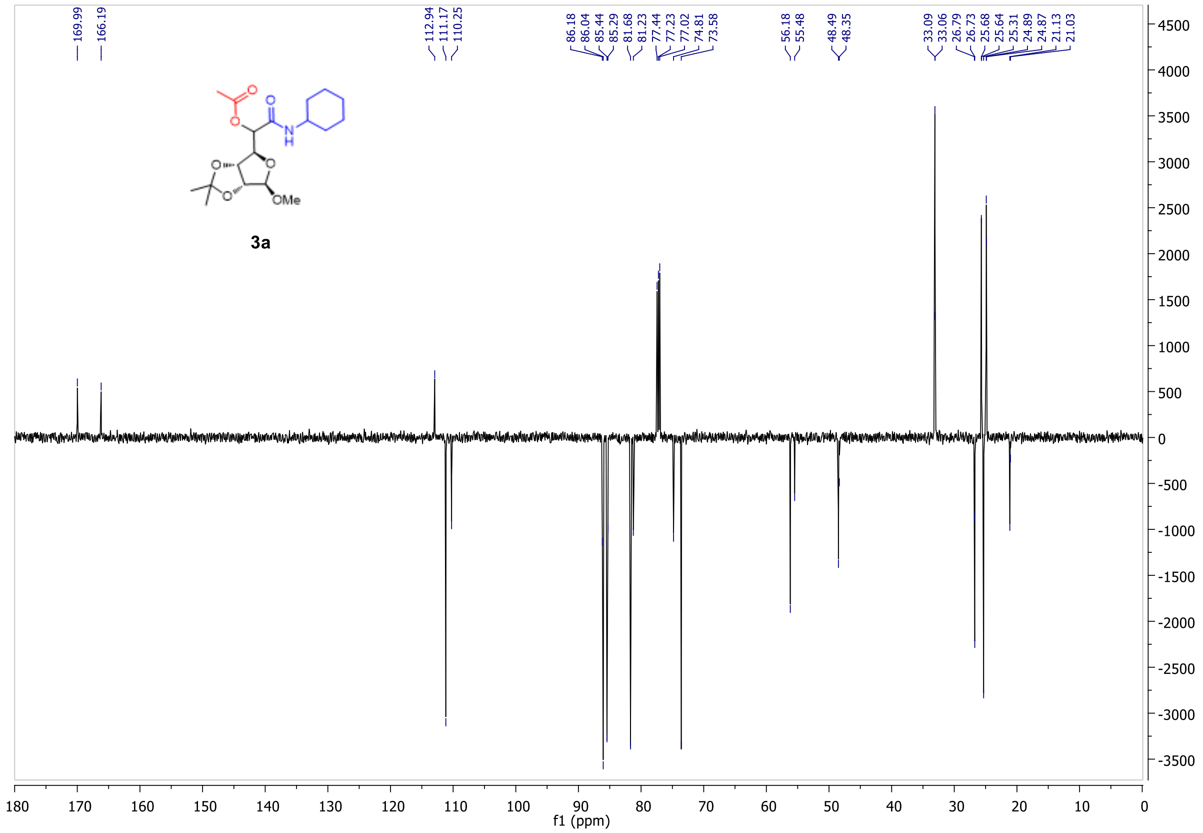
169.99
166.19
112.94
111.17
110.25
86.18
86.04
85.44
85.29
81.68
81.23
77.44
77.23
77.02
74.81
73.58
56.18
55.48
48.49
48.35
33.09
33.06
26.79
26.73
25.68
25.64
25.31
24.89
24.87
21.13
21.03
OMe
3a
f1 (ppm)

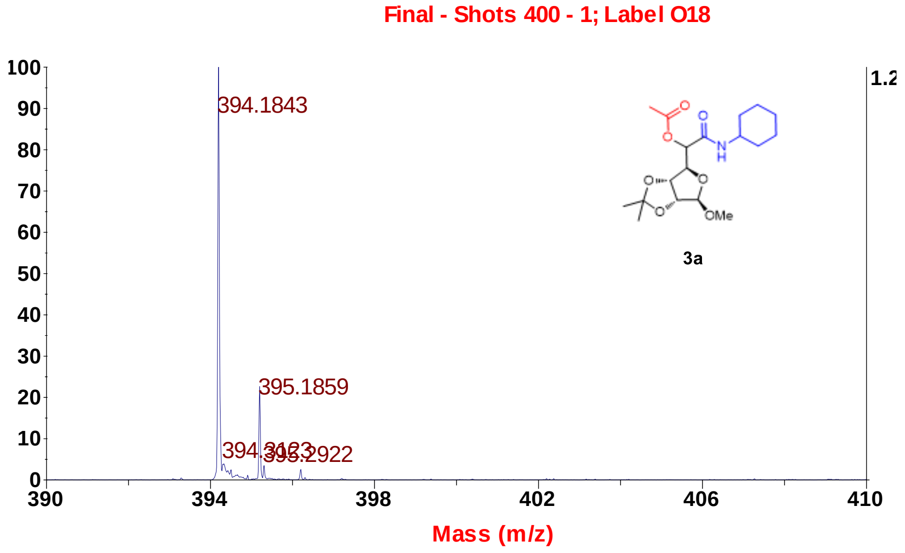
Final - Shots 400 - 1; Label O18
394.1843
395.1859
394.3123
395.2922
OMe
3a
Mass (m/z)

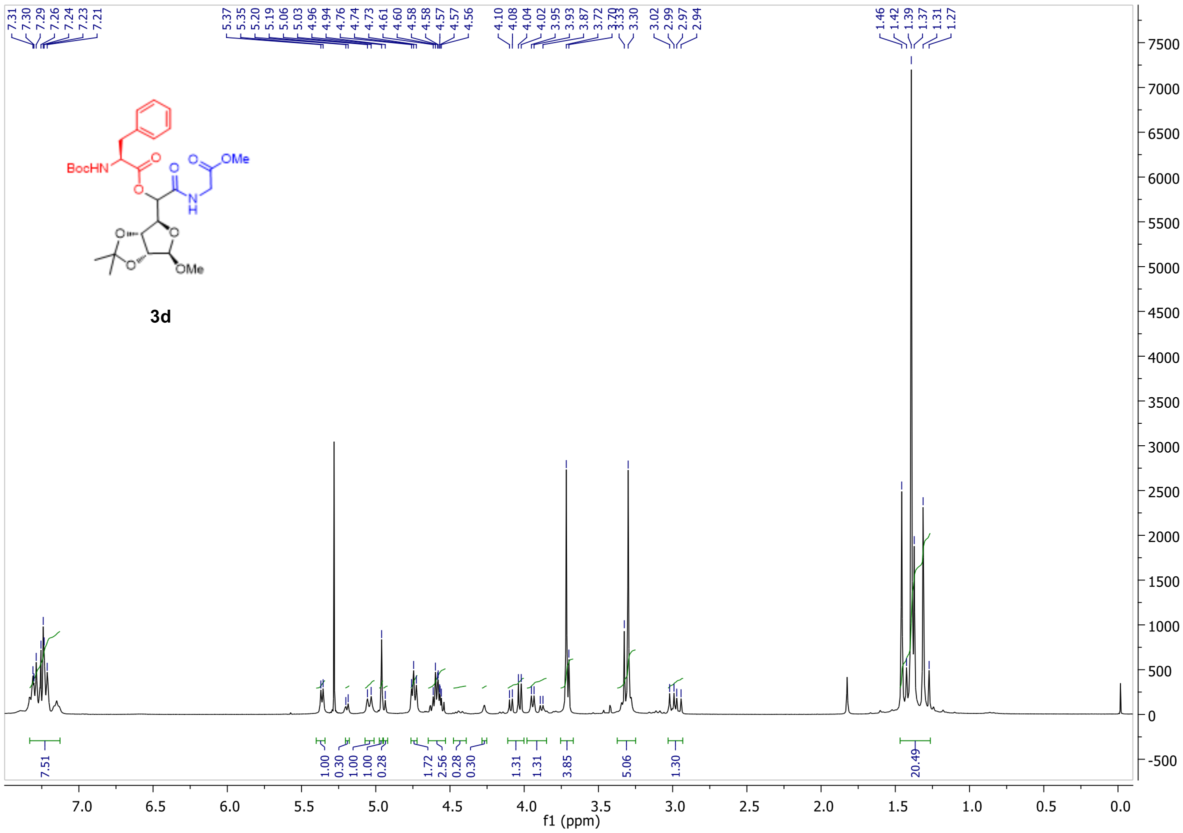

7.31
7.30
7.29
7.26
7.24
7.23
7.21
5.37
5.35
5.20
5.19
5.06
5.03
4.96
4.94
4.76
4.74
4.73
4.61
4.60
4.58
4.58
4.57
4.56
4.10
4.08
4.04
4.02
3.95
3.93
3.87
3.72
3.70
3.33
3.30
3.02
2.99
2.97
2.94
1.46
1.42
1.39
1.37
1.31
1.27
BocHN
O
OMe
3d
7.51
1.00
0.30
1.00
1.00
0.28
1.72
2.56
0.28
0.30
1.31
1.31
3.85
5.06
1.30
20.49
f1 (ppm)

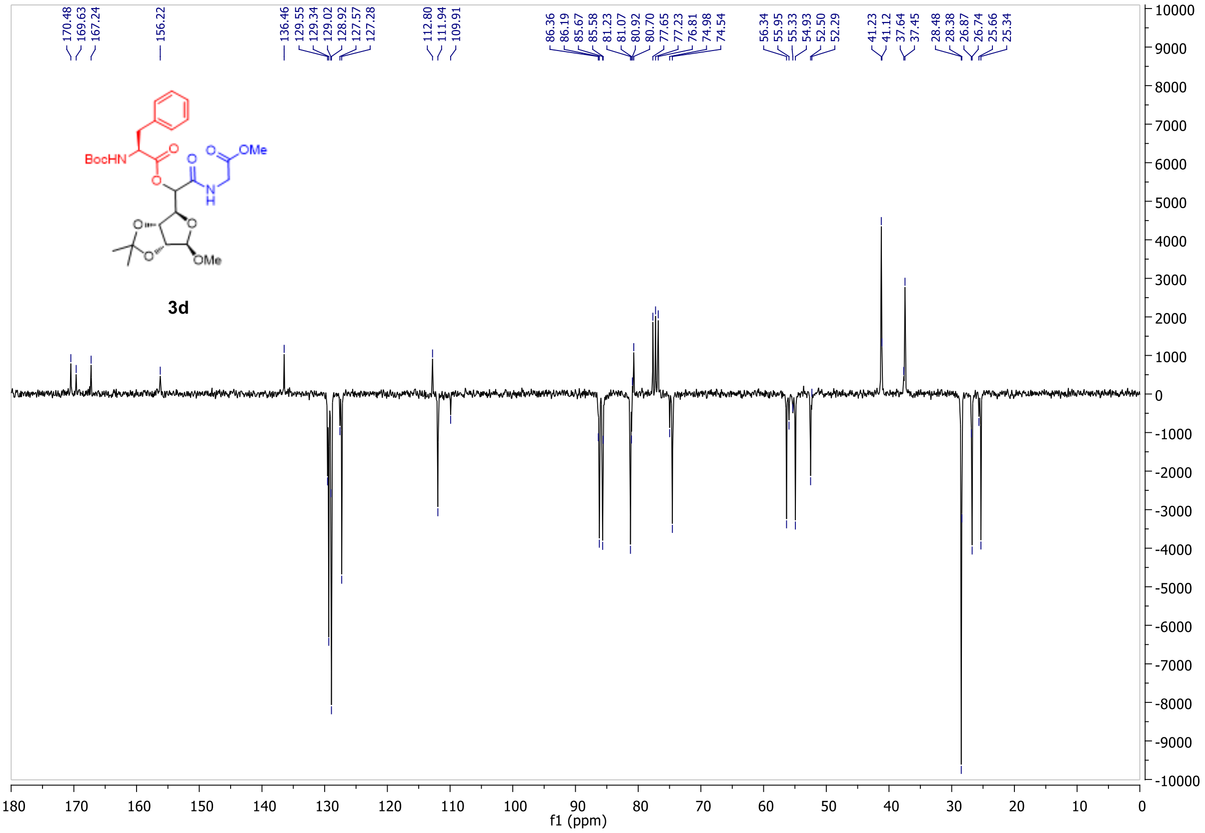

170.48
169.63
167.24
156.22
136.46
129.55
129.34
129.02
128.92
127.57
127.28
112.80
111.94
109.91
86.36
86.19
85.67
85.58
81.23
81.07
80.92
80.70
77.65
77.23
76.81
74.98
74.54
56.34
55.95
55.33
54.93
52.50
52.29
41.23
41.12
37.64
37.45
28.48
28.38
26.87
26.74
25.66
25.34
BocHN
O
OMe
3d
f1 (ppm)

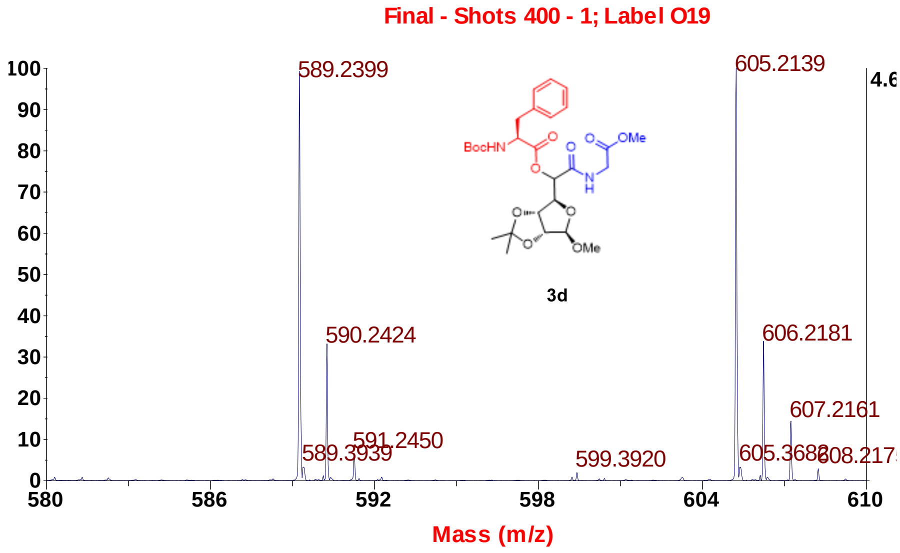

Final - Shots 400 - 1; Label O19
589.2399
605.2139
590.2424
606.2181
607.2161
591.2450
589.3939
599.3920
605.3688
BocHN
OMe
3d
Mass (m/z)

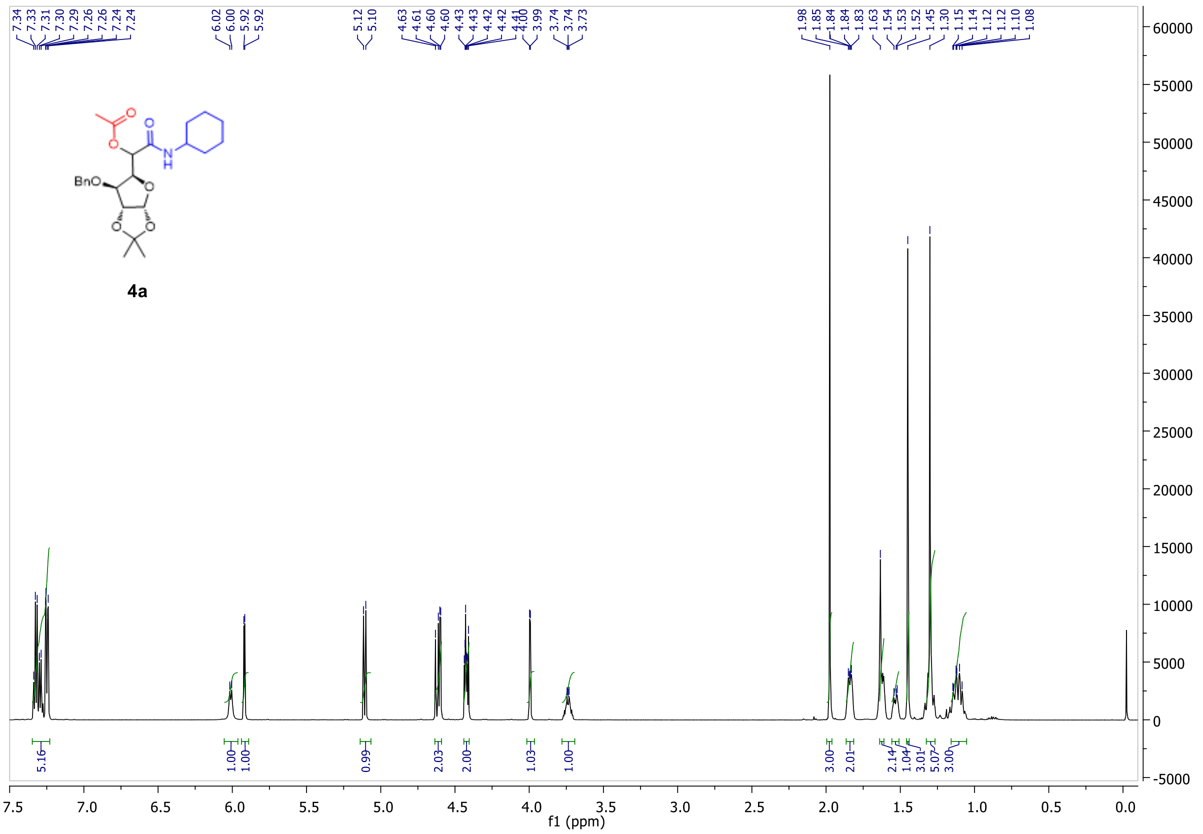

BnO
4a
f1 (ppm)

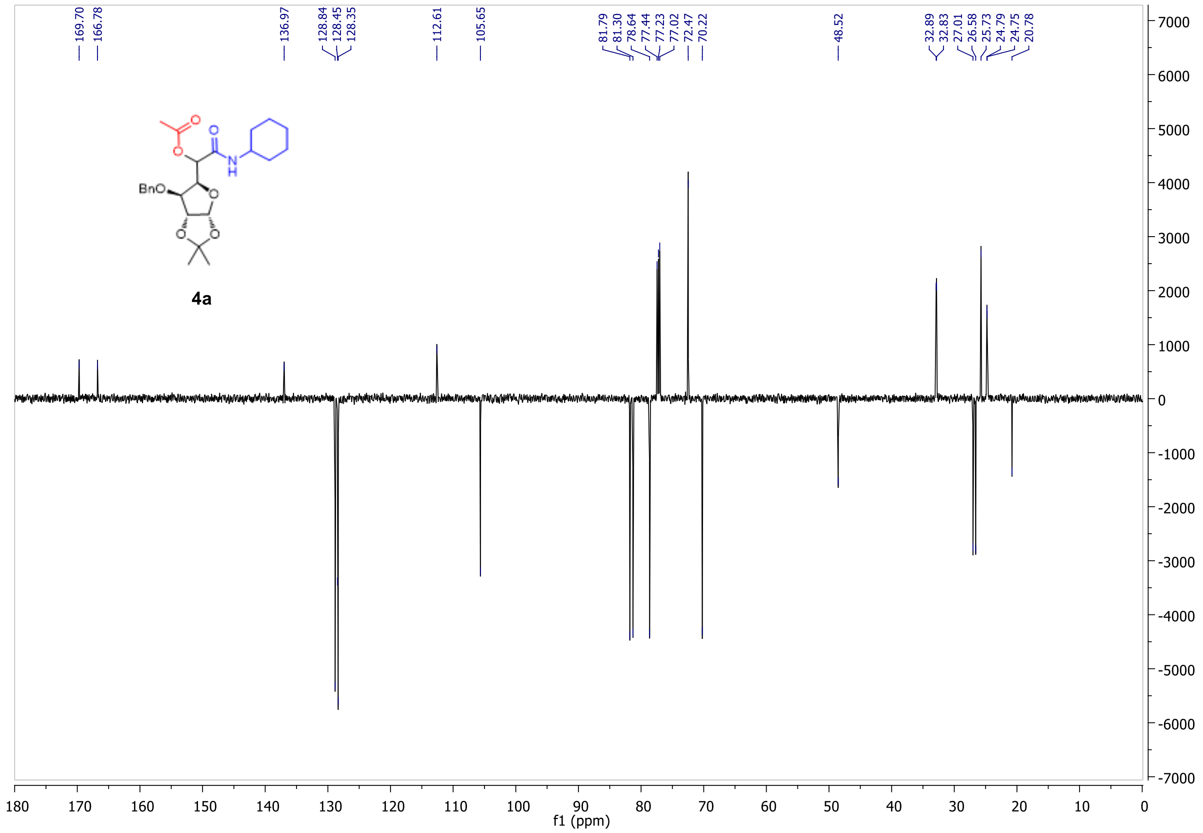
169.70
166.78
136.97
128.84
128.45
128.35
112.61
105.65
81.79
81.30
78.64
77.44
77.23
77.02
72.47
70.22
48.52
32.89
32.83
27.01
26.58
25.73
24.79
24.75
20.78
BnO
4a
f1 (ppm)

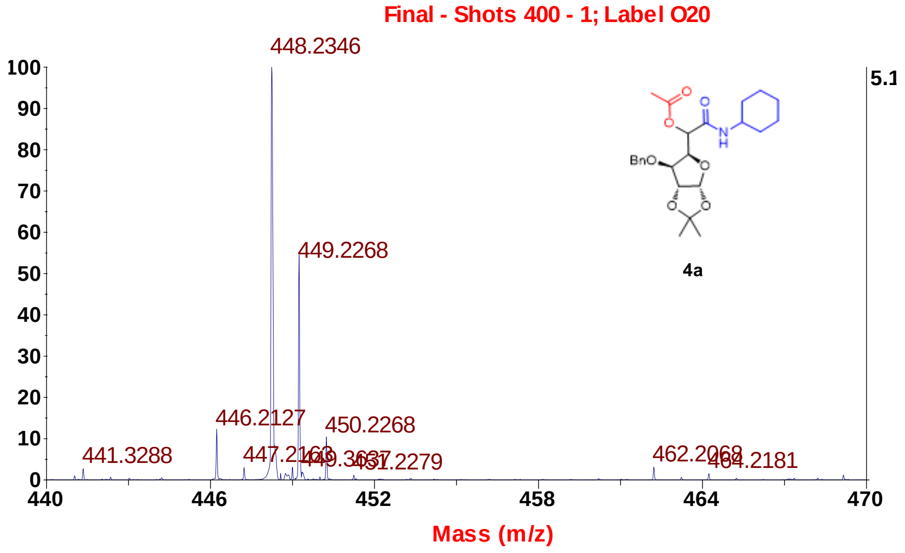
Final - Shots 400 - 1; Label O20
448.2346
449.2268
446.2127
450.2268
441.3288
447.2163
449.3637
451.2279
462.2068
464.2181
5.1
BnO
4a
Mass (m/z)

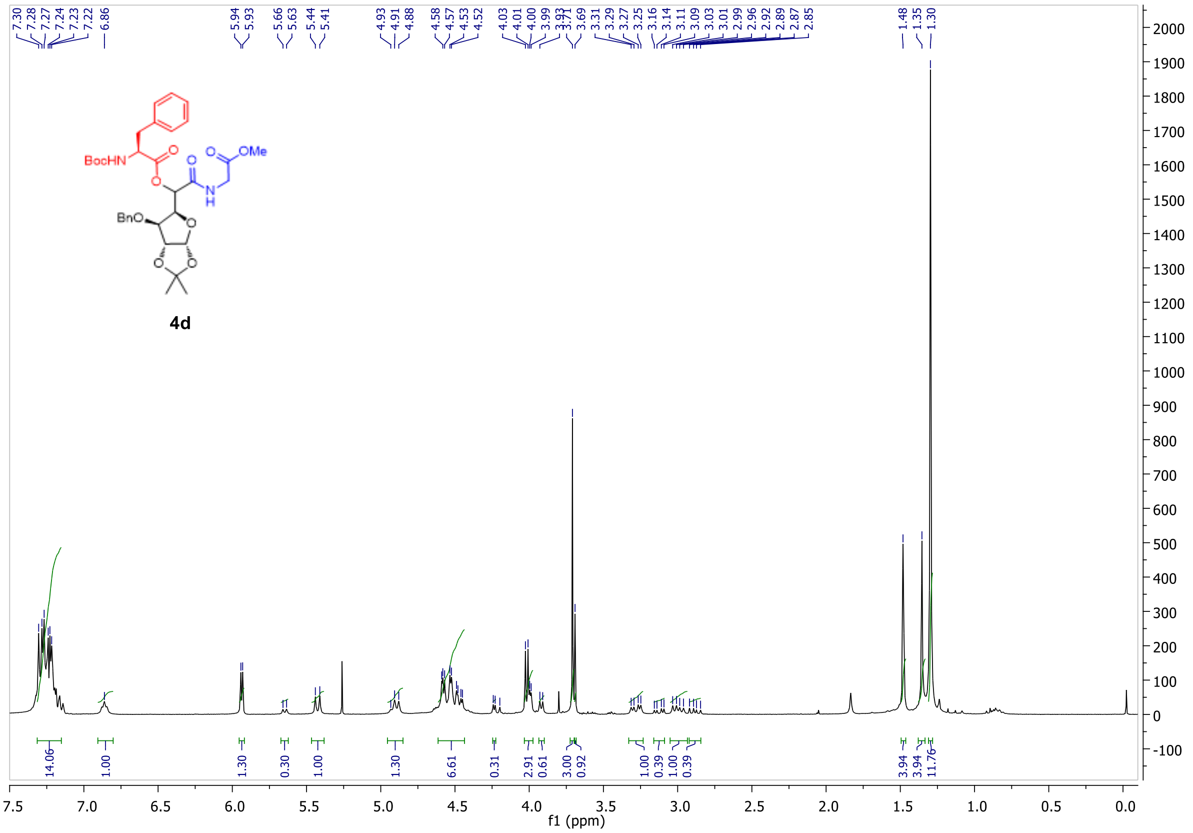
7.30 7.28 7.27 7.24 7.23 7.22 6.86 5.94 5.93 5.66 5.63 5.44 5.41 4.93 4.91 4.88 4.58 4.57 4.53 4.52 4.03 4.01 4.00 3.99 3.93 3.71 3.69 3.31 3.29 3.27 3.25 3.16 3.14 3.11 3.09 3.03 3.01 2.99 2.96 2.92 2.89 2.87 2.85 1.48 1.35 1.30
BocHN
O
OMe
N
H
BnO
4d
14.06 1.00 1.30 0.30 1.00 1.30 6.61 0.31 2.91 0.61 3.00 0.92 1.00 0.39 1.00 0.39 3.94 3.94 11.76
f1 (ppm)

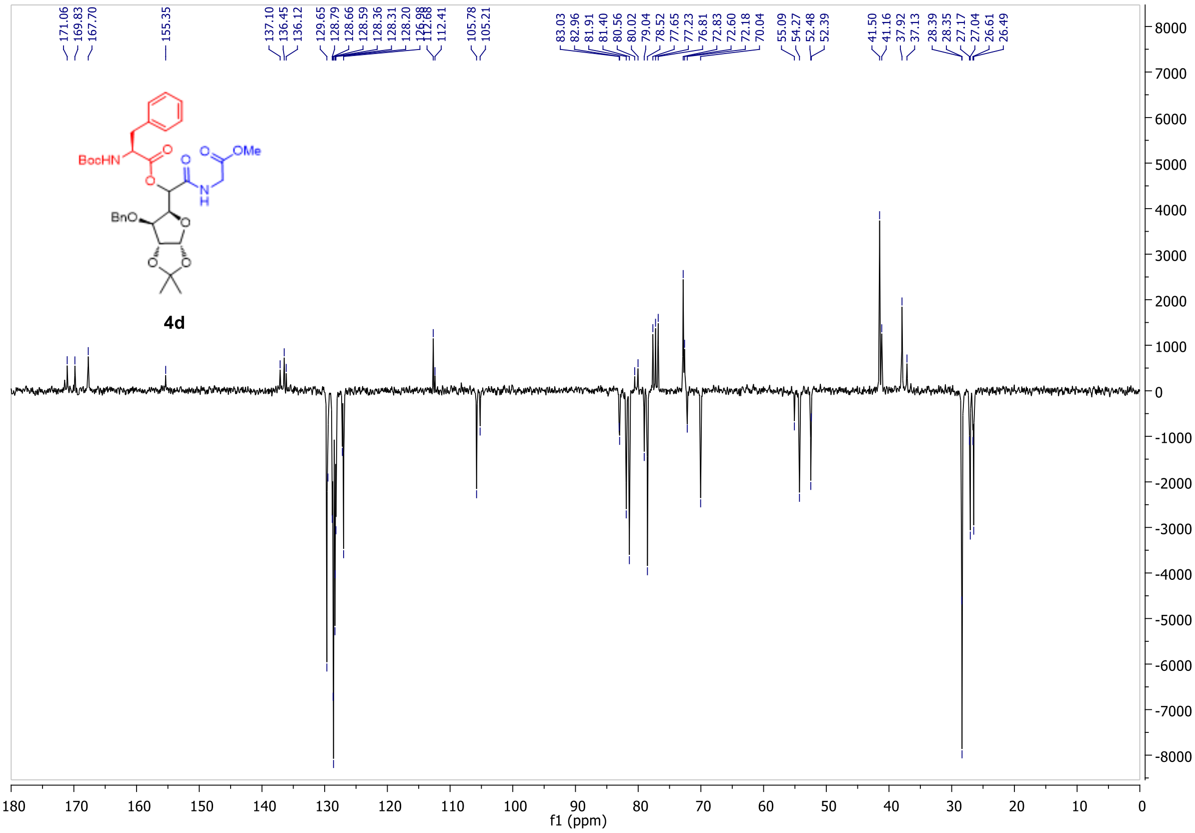
171.06 169.83 167.70 155.35 137.10 136.45 136.12 129.65 128.79 128.66 128.59 128.36 128.31 128.20 126.88 112.68 112.41 105.78 105.21 83.03 82.96 81.91 81.40 80.56 80.02 79.04 78.52 77.65 77.23 76.81 72.83 72.60 72.18 70.04 55.09 54.27 52.48 52.39 41.50 41.16 37.92 37.13 28.39 28.35 27.17 27.04 26.61 26.49
BocHN
O
OMe
N
H
BnO
4d
f1 (ppm)

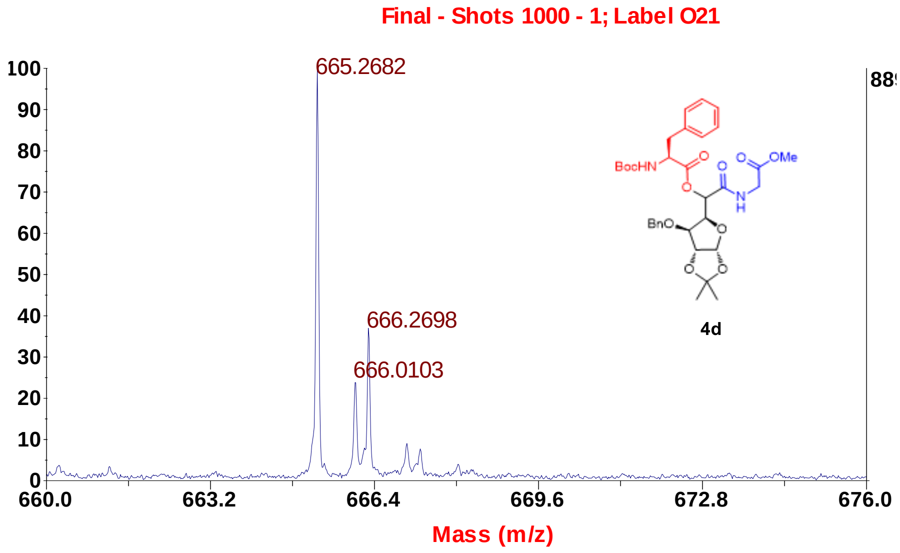
Final - Shots 1000 - 1; Label O21
665.2682
666.2698
666.0103
Mass (m/z)
BocHN
OMe
BnO
4d

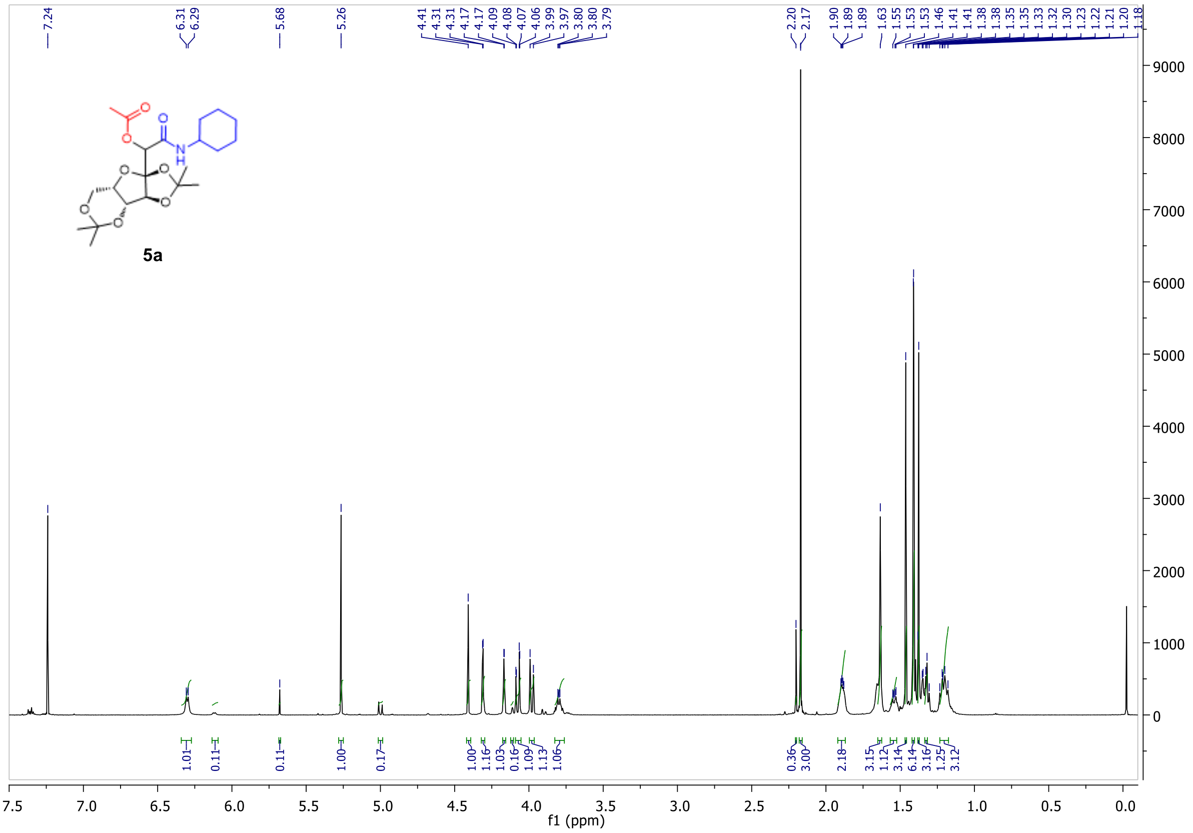
7.24
6.31
6.29
5.68
5.26
2.20
2.17
5a
f1 (ppm)

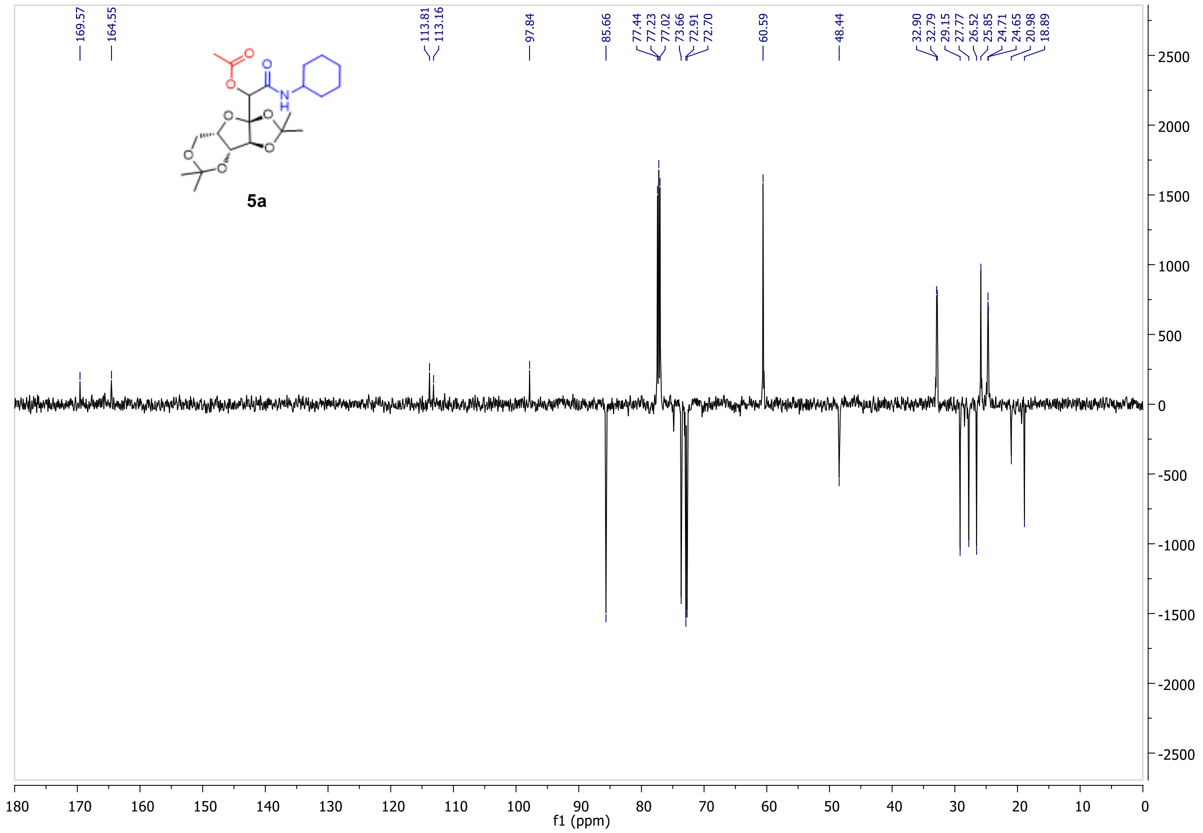
169.57
164.55
113.81
113.16
97.84
85.66
77.44
77.23
77.02
73.66
72.91
72.70
60.59
48.44
32.90
32.79
29.15
27.77
26.52
25.85
24.71
24.65
20.98
18.89
5a
f1 (ppm)

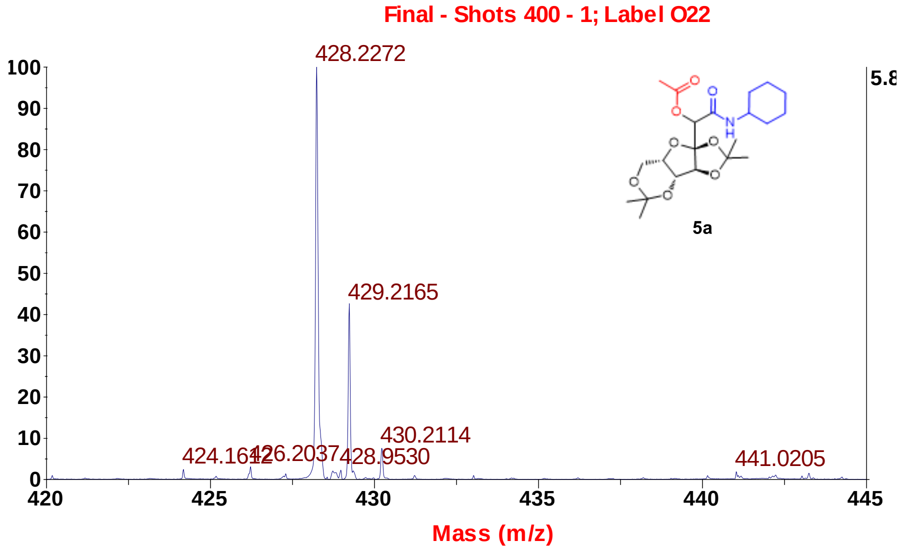
Final - Shots 400 - 1; Label O22
428.2272
429.2165
430.2114
424.1642
426.2037
428.9530
441.0205
5a
Mass (m/z)

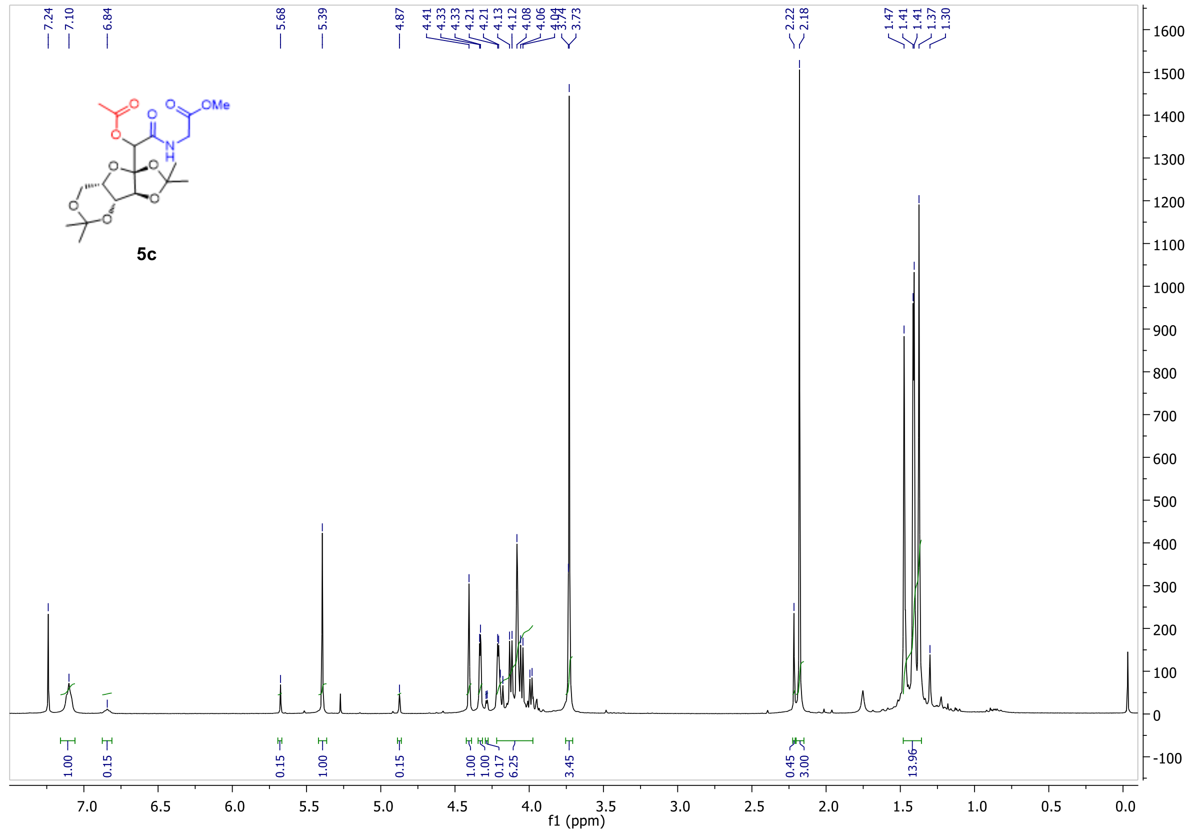
7.24
7.10
6.84
5.68
5.39
4.87
4.41
4.33
4.33
4.21
4.21
4.13
4.12
4.08
4.06
3.73
2.22
2.18
1.47
1.41
1.41
1.37
1.30
OMe
O
O
O
N
H
5c
1.00
0.15
0.15
1.00
0.15
1.00
1.00
0.17
6.25
3.45
0.45
3.00
13.96
f1 (ppm)

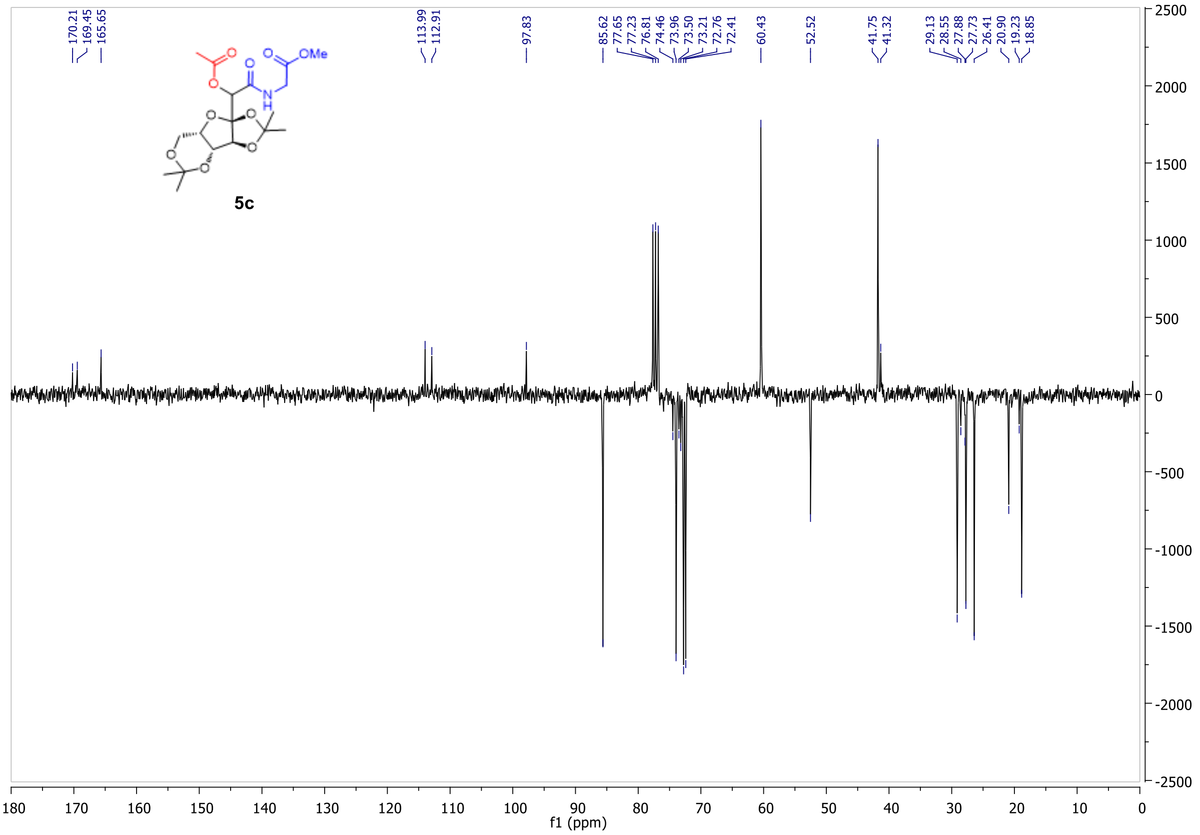
170.21
169.45
165.65
113.99
112.91
97.83
85.62
77.65
77.23
76.81
74.46
73.96
73.50
73.21
72.76
72.41
60.43
52.52
41.75
41.32
29.13
28.55
27.88
27.73
26.41
20.90
19.23
18.85
OMe
5c
f1 (ppm)

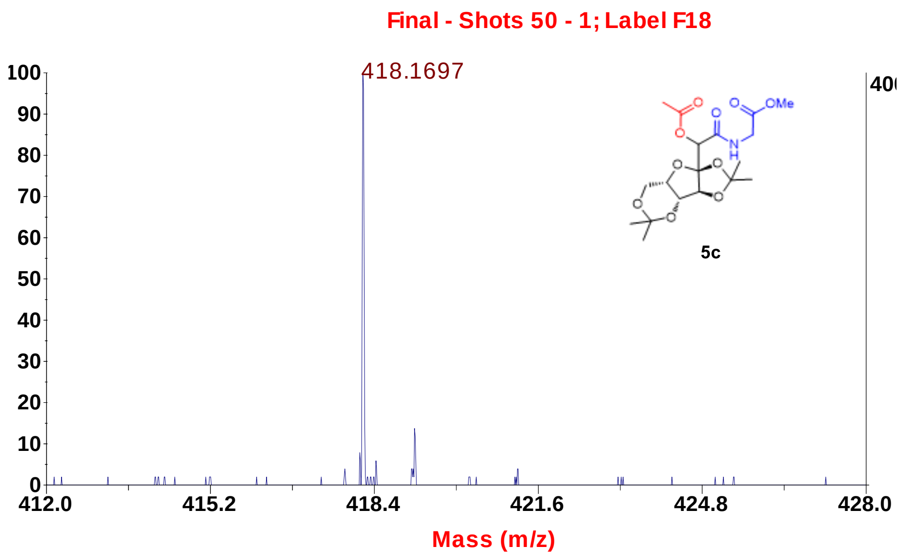
Final - Shots 50 - 1; Label F18
418.1697
100
90
80
70
60
50
40
30
20
10
0
412.0
415.2
418.4
421.6
424.8
428.0
Mass (m/z)
OMe
N
H
5c

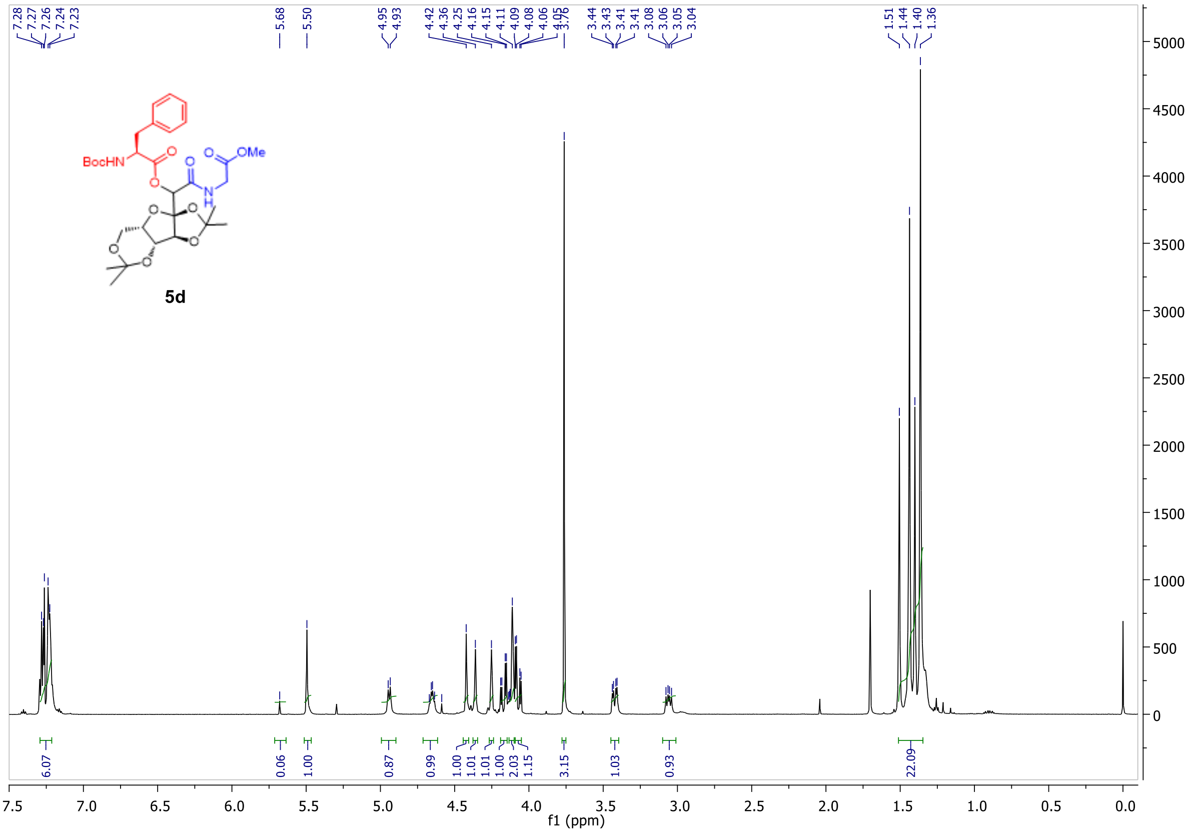
7.28
7.27
7.26
7.24
7.23
5.68
5.50
4.95
4.93
4.42
4.36
4.25
4.16
4.15
4.11
4.09
4.08
4.06
4.05
3.76
3.44
3.43
3.41
3.41
3.08
3.06
3.05
3.04
1.51
1.44
1.40
1.36
BocHN
OMe
N
H
5d
6.07
0.06
1.00
0.87
0.99
1.00
1.01
1.01
1.00
2.03
1.15
3.15
1.03
0.93
22.09
f1 (ppm)

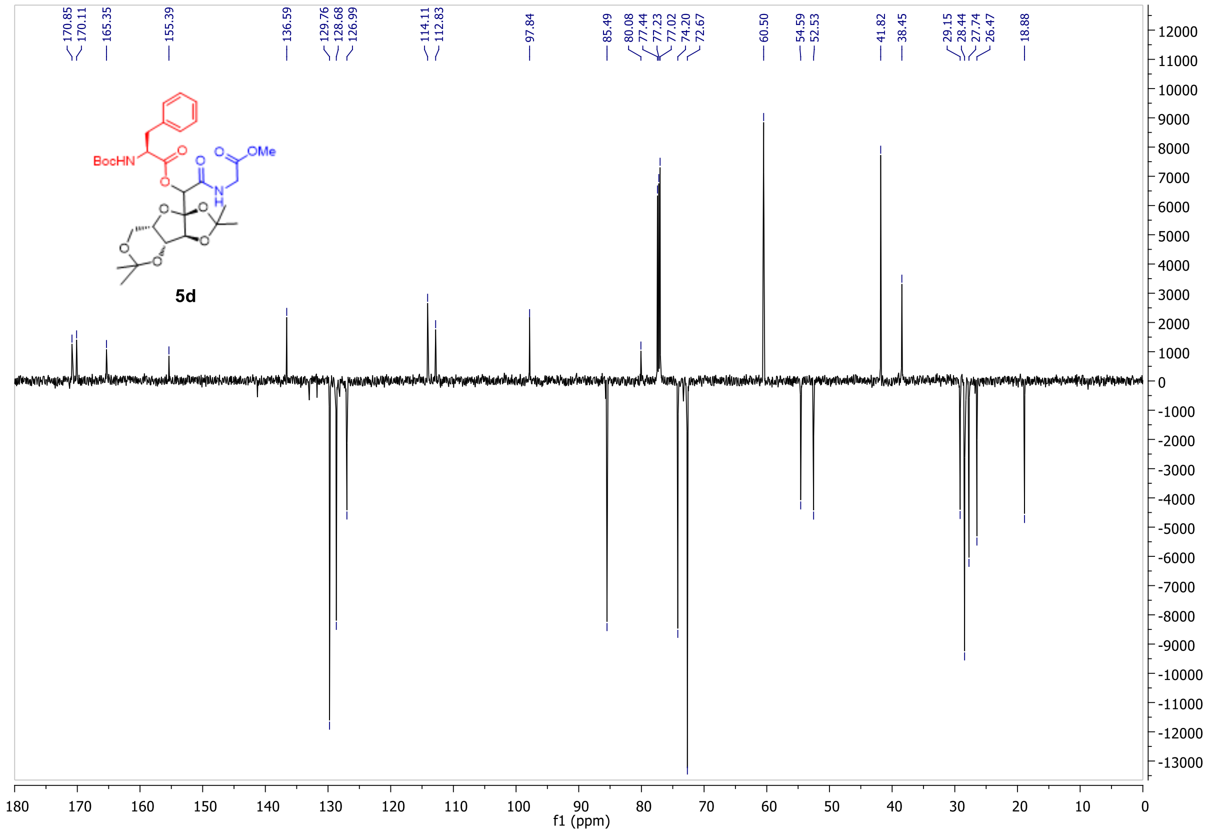
170.85
170.11
165.35
155.39
136.59
129.76
128.68
126.99
114.11
112.83
97.84
85.49
80.08
77.44
77.23
77.02
74.20
72.67
60.50
54.59
52.53
41.82
38.45
29.15
28.44
27.74
26.47
18.88
BocHN
OMe
5d
f1 (ppm)

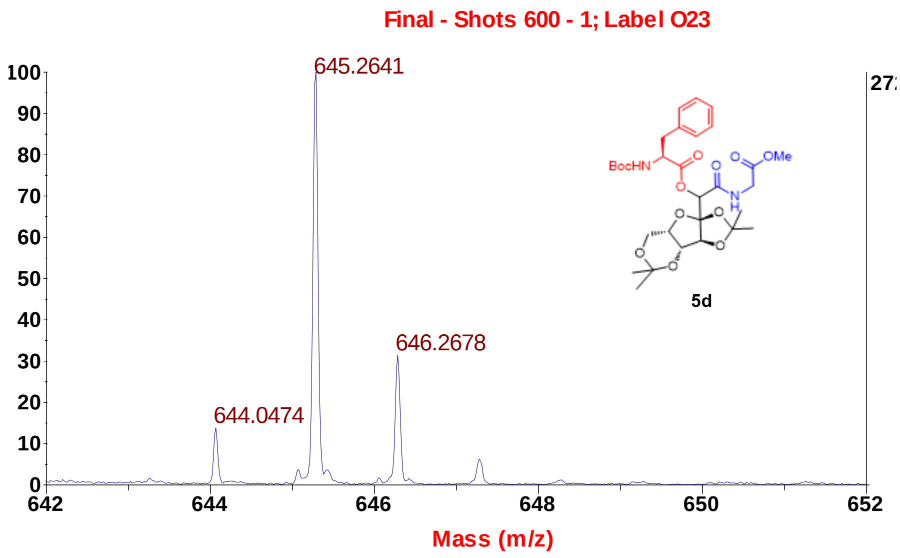
Final - Shots 600 - 1; Label O23
645.2641
646.2678
644.0474
BocHN
OMe
5d
Mass (m/z)

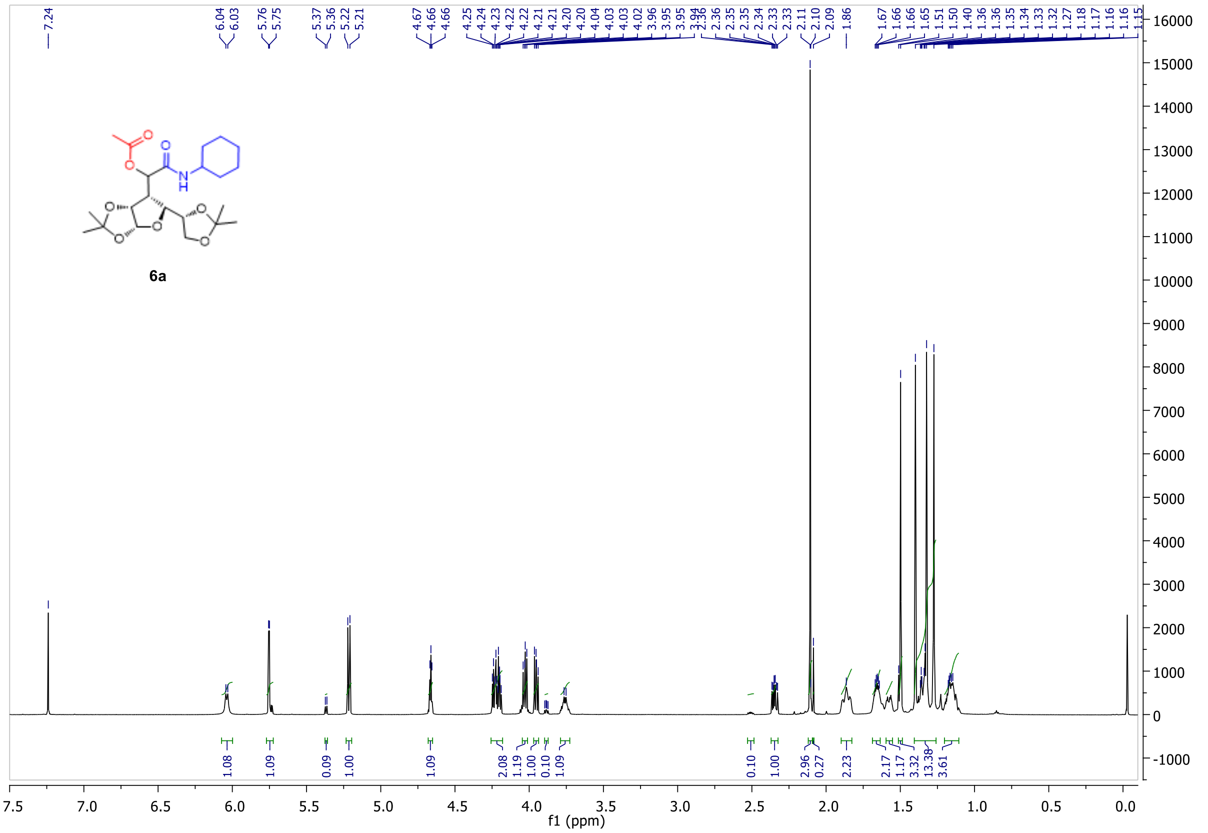

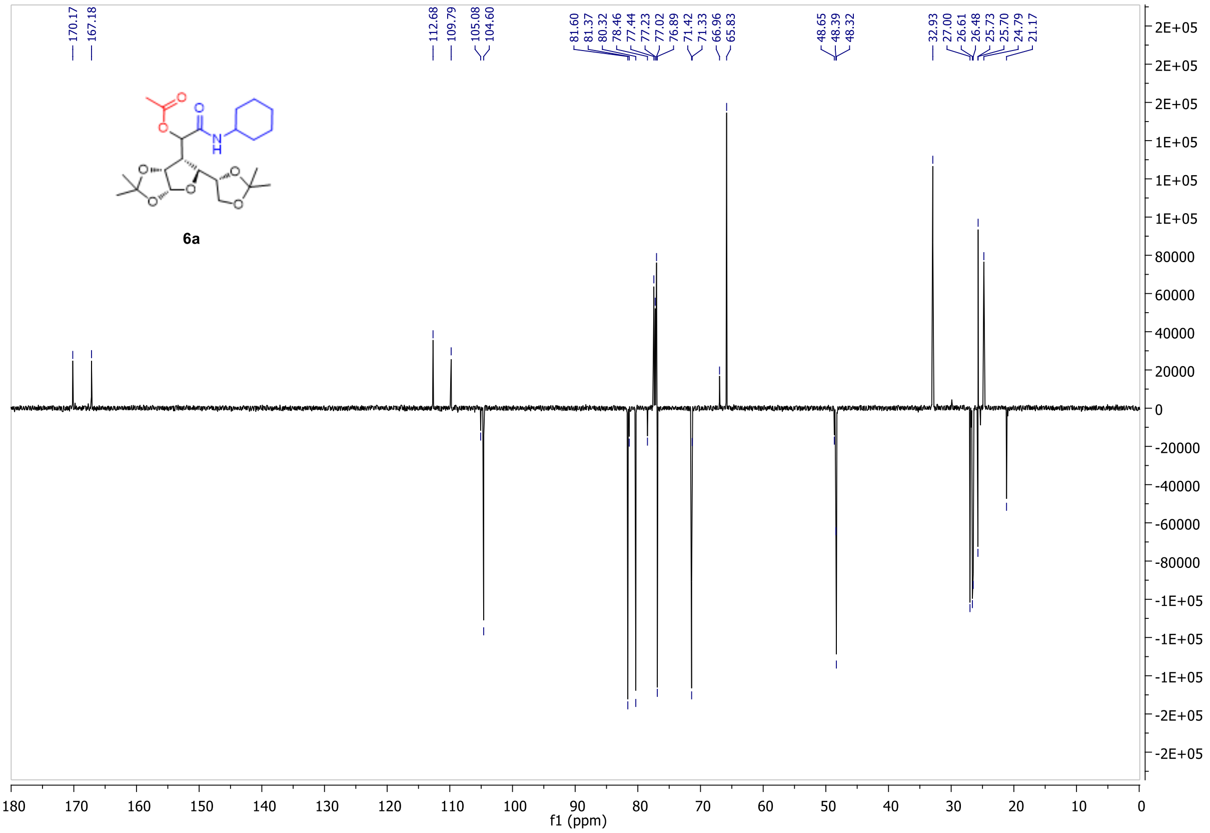

Final - Shots 800 - 1; Label P7

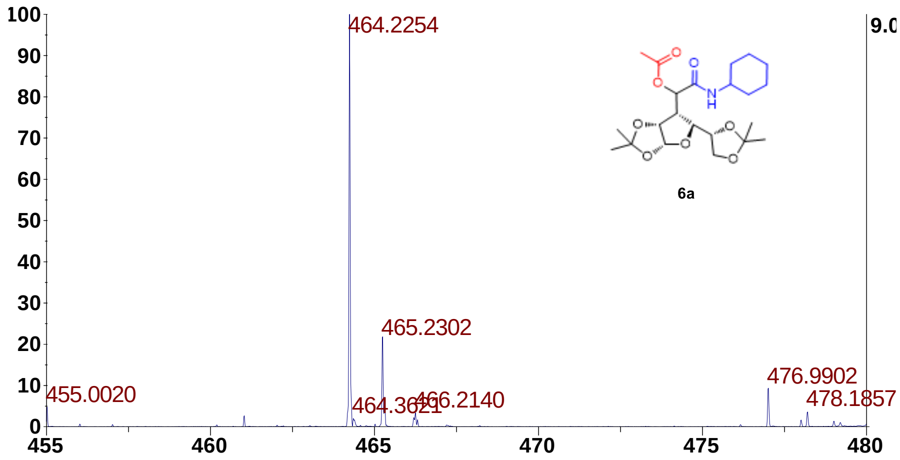

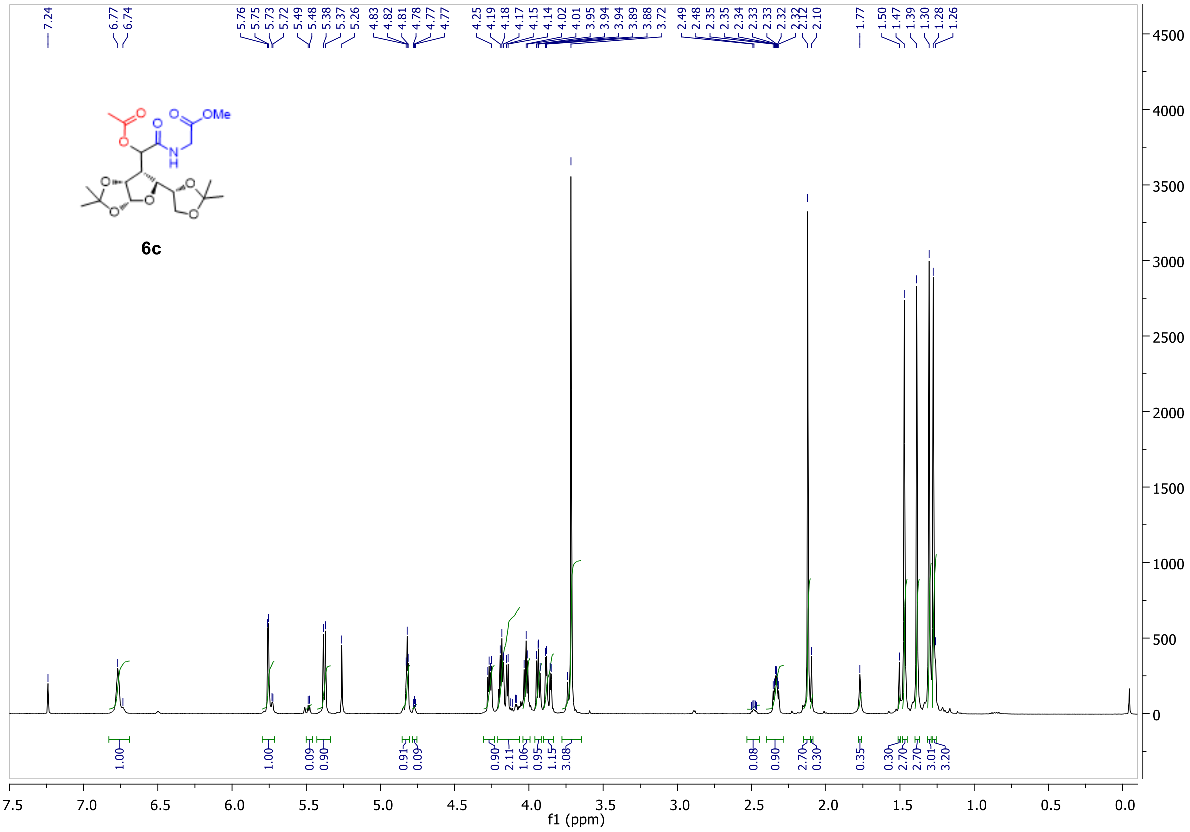

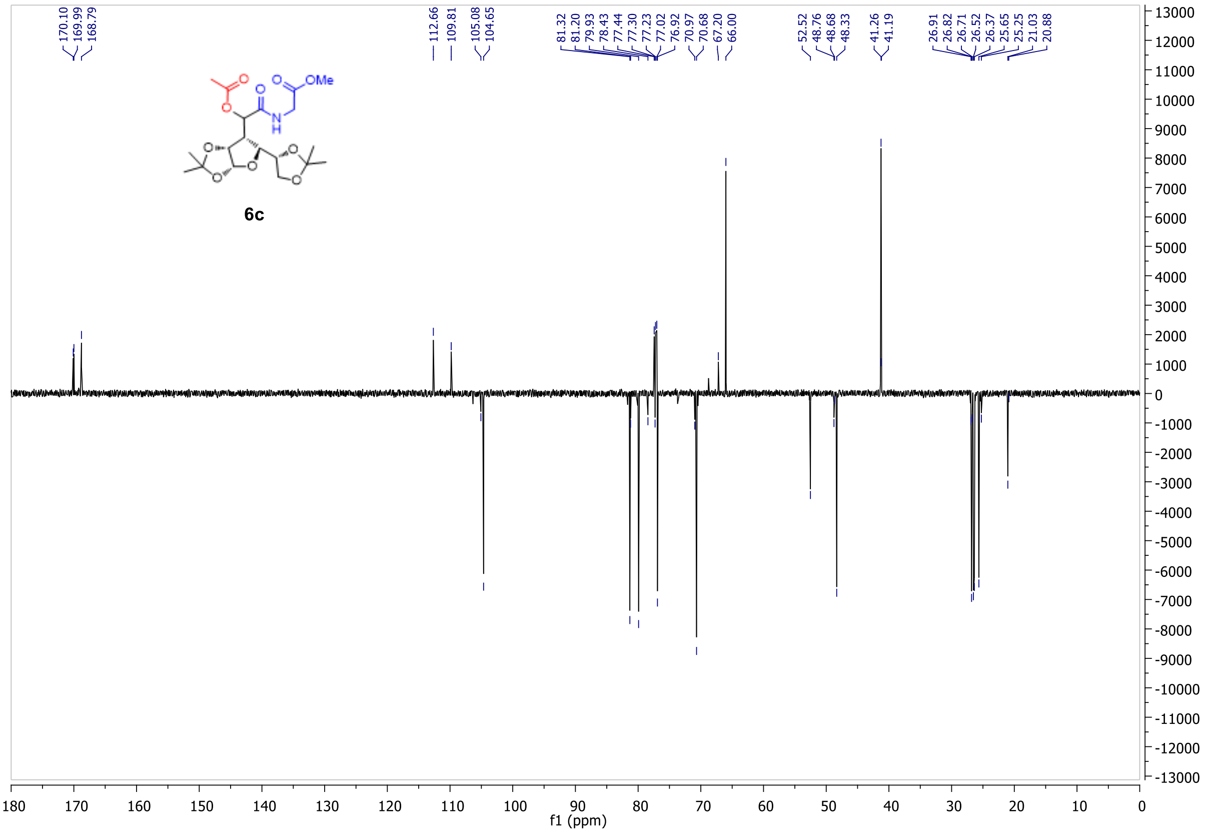
170.10
169.99
168.79
112.66
109.81
105.08
104.65
81.32
81.20
79.93
78.43
77.44
77.30
77.23
77.02
76.92
70.97
70.68
67.20
66.00
52.52
48.76
48.68
48.33
41.26
41.19
26.91
26.82
26.71
26.52
26.37
25.65
25.25
21.03
20.88
6c
f1 (ppm)

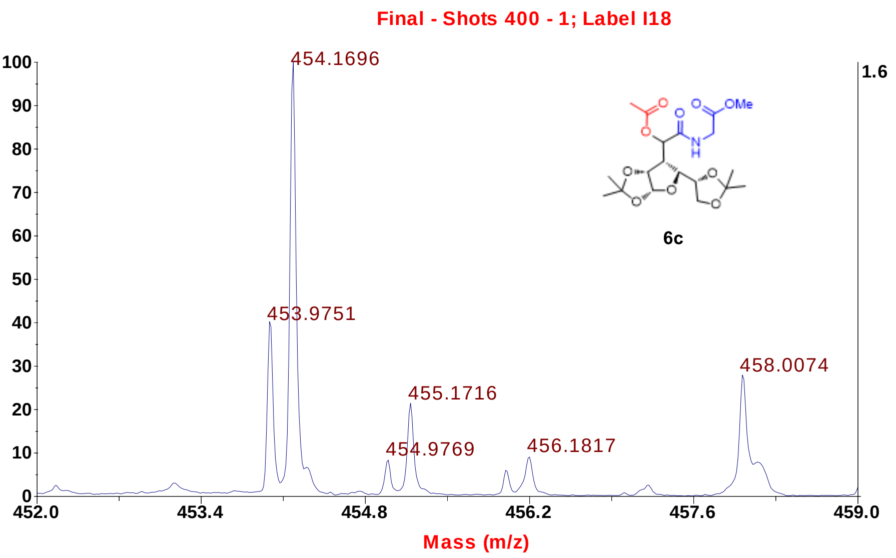
Final - Shots 400 - 1; Label I18
454.1696
453.9751
455.1716
454.9769
456.1817
458.0074
1.6
6c
Mass (m/z)

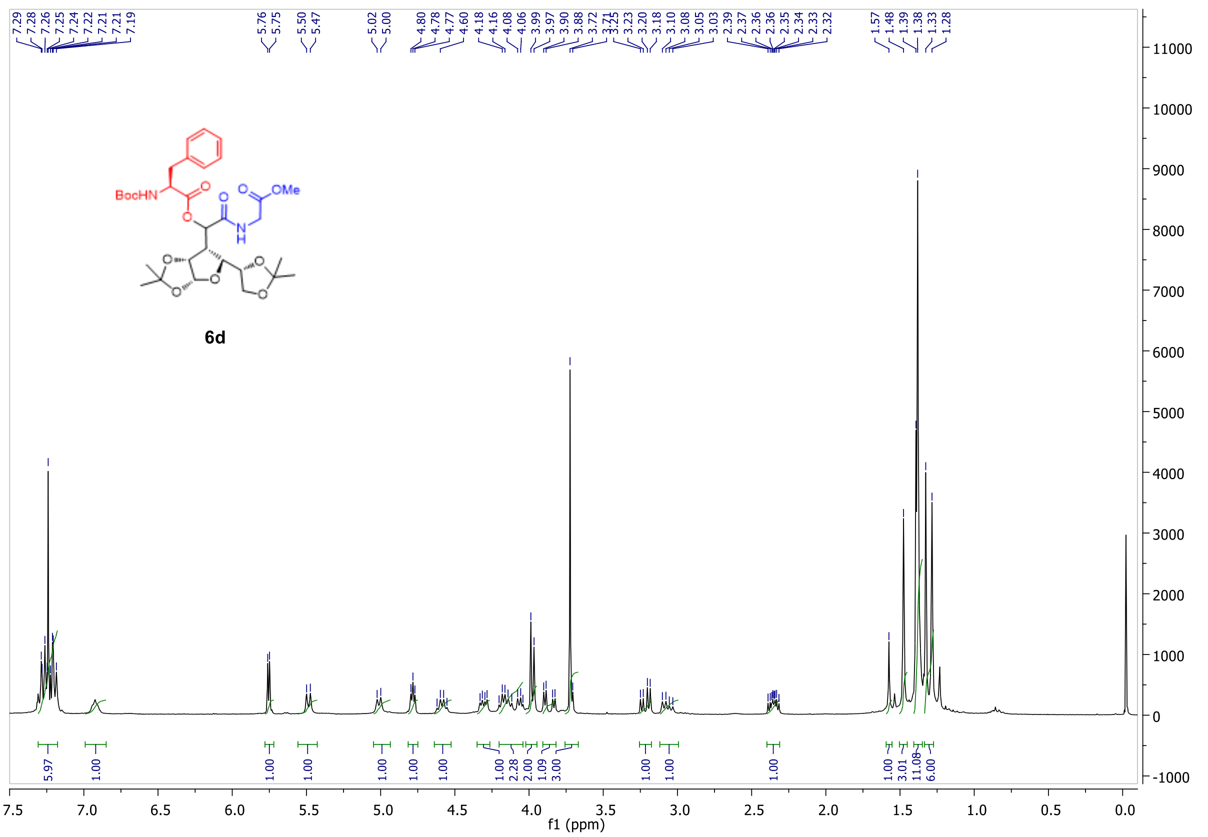

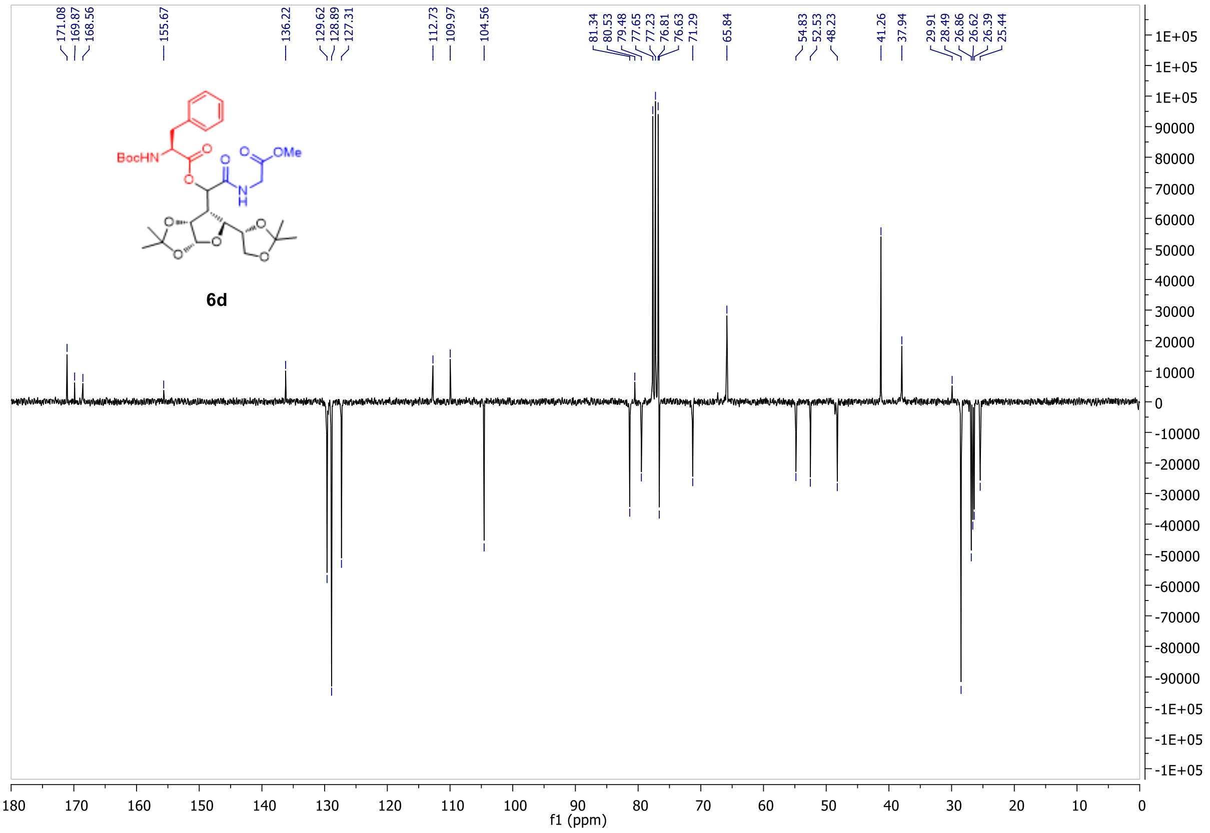

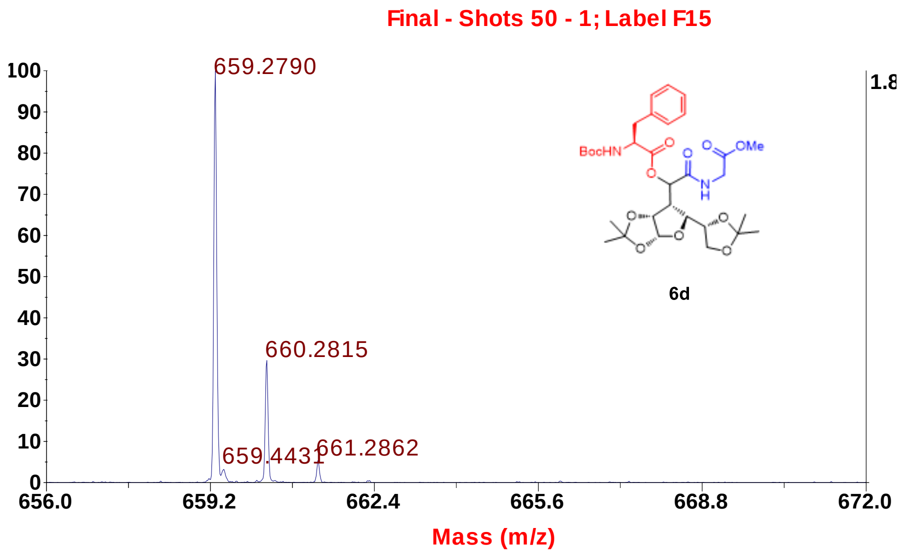
Final - Shots 50 - 1; Label F15
659.2790
660.2815
659.4431
661.2862
1.8
BocHN
OMe
6d
Mass (m/z)
656.0
659.2
662.4
665.6
668.8
672.0

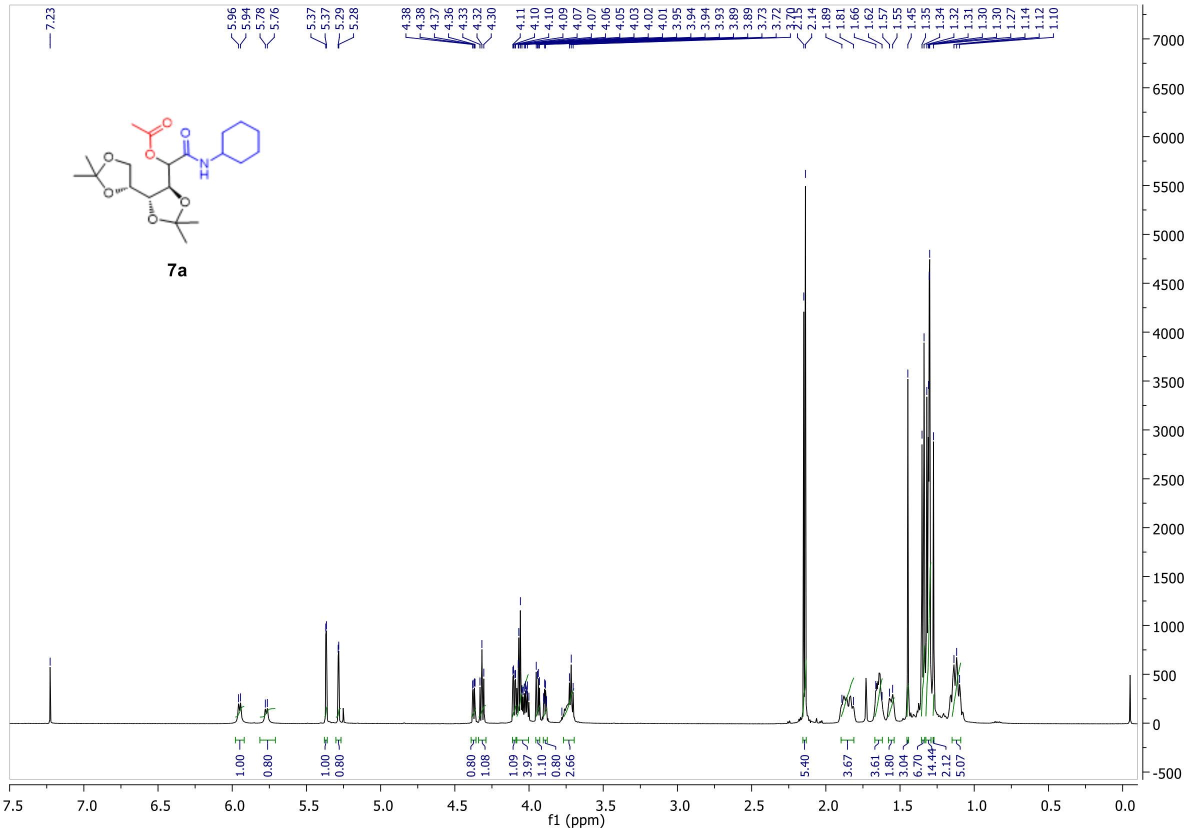
7.23
5.96
5.94
5.78
5.76
5.37
5.37
5.29
5.28
4.38
4.38
4.37
4.36
4.33
4.32
4.30
4.11
4.10
4.10
4.09
4.07
4.07
4.06
4.05
4.03
4.02
4.01
3.95
3.94
3.94
3.93
3.89
3.73
3.72
3.70
2.15
2.14
1.89
1.81
1.66
1.62
1.57
1.55
1.45
1.35
1.34
1.32
1.31
1.30
1.30
1.27
1.14
1.12
1.10
7a
1.00
0.80
1.00
0.80
0.80
1.08
1.09
3.97
1.10
0.80
2.66
5.40
3.67
3.61
1.80
3.04
6.70
14.44
2.12
5.07
f1 (ppm)

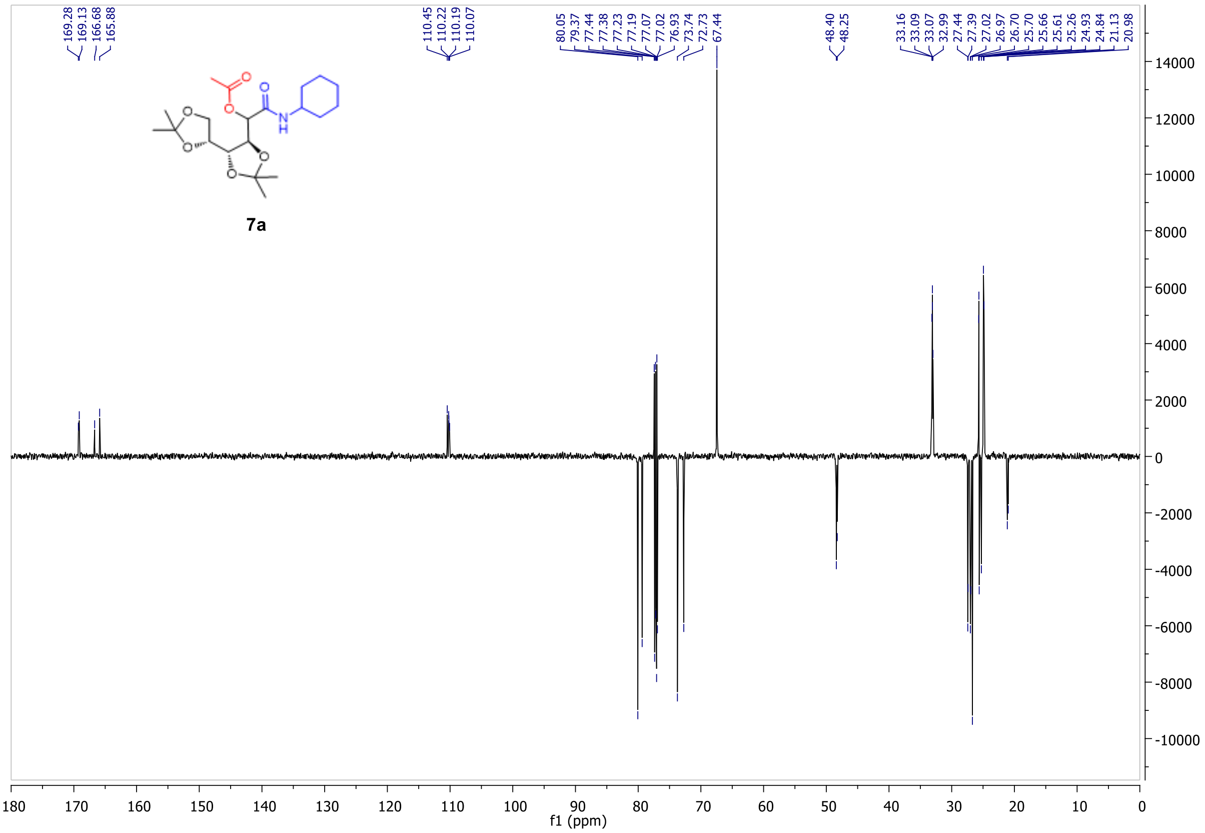
169.28
169.13
166.68
165.88
110.45
110.22
110.19
110.07
80.05
79.37
77.44
77.38
77.23
77.19
77.07
77.02
76.93
73.74
72.73
67.44
48.40
48.25
33.16
33.09
33.07
32.99
27.44
27.39
27.02
26.97
26.70
25.70
25.66
25.61
25.26
24.93
24.84
21.13
20.98
7a
f1 (ppm)

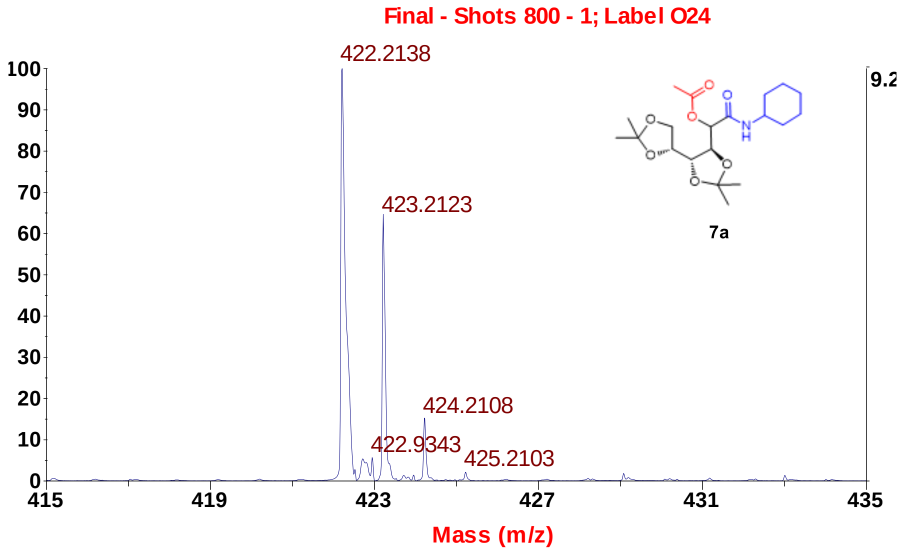
Final - Shots 800 - 1; Label O24
422.2138
423.2123
424.2108
422.9343
425.2103
9.2
7a
Mass (m/z)

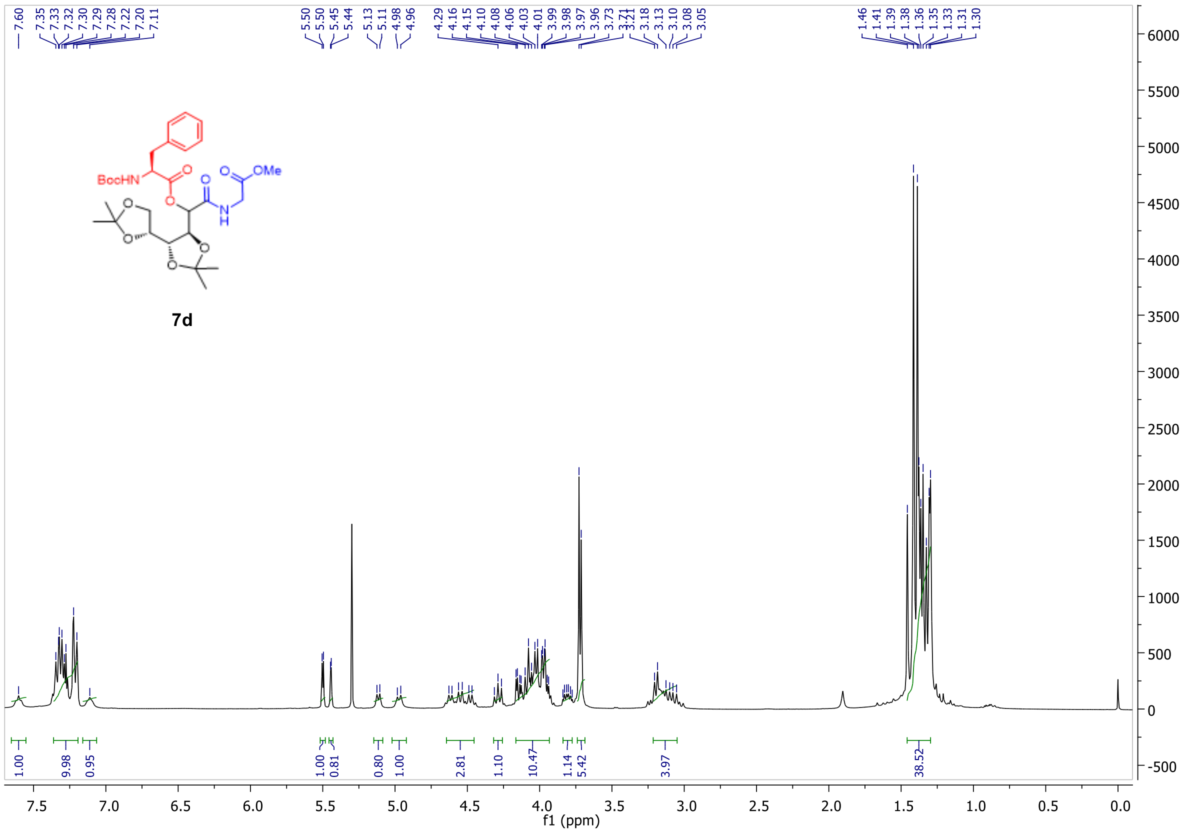
7.60
7.35 7.33 7.32 7.30 7.29 7.28 7.22 7.20 7.11
5.50 5.50 5.45 5.44
5.13 5.11 4.98 4.96
4.29 4.16 4.15 4.10 4.08 4.06 4.03 4.01 3.99 3.98 3.97 3.96 3.73 3.21 3.18 3.13 3.10 3.08 3.05
1.46 1.41 1.39 1.38 1.36 1.35 1.33 1.31 1.30
BocHN
OMe
7d
1.00 9.98 0.95 1.00 0.81 0.80 1.00 2.81 1.10 10.47 1.14 5.42 3.97 38.52
f1 (ppm)

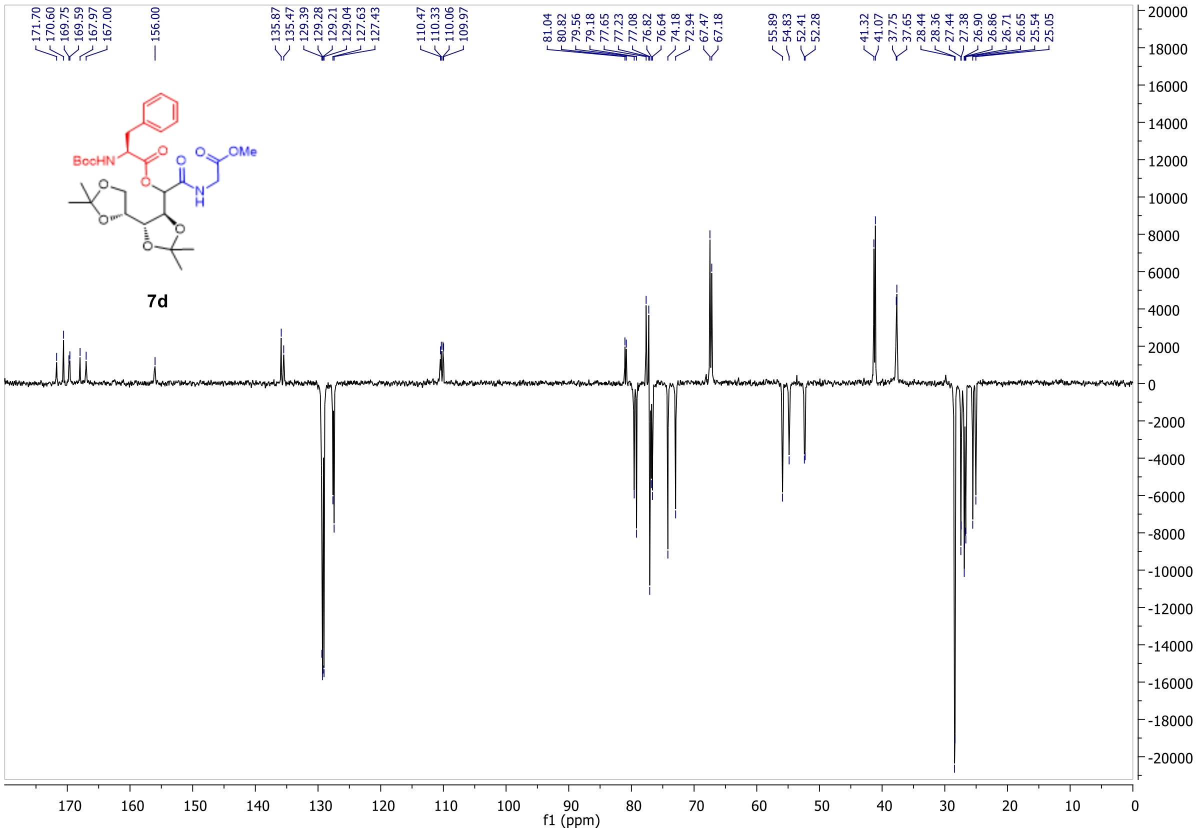
171.70 170.60 169.75 169.59 167.97 167.00
156.00
135.87 135.47 129.39 129.28 129.21 129.04 127.63 127.43
110.47 110.33 110.06 109.97
81.04 80.82 79.56 79.18 77.65 77.23 77.08 76.82 76.64 74.18 72.94 67.47 67.18
55.89 54.83 52.41 52.28
41.32 41.07 37.75 37.65 28.44 28.36 27.44 27.38 26.90 26.86 26.71 26.65 25.54 25.05
BocHN
OMe
7d
f1 (ppm)

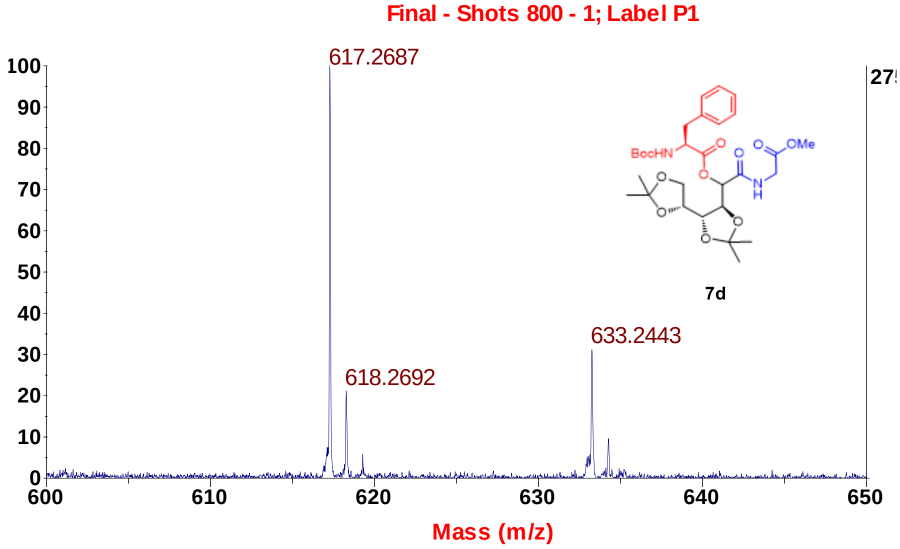
Final - Shots 800 - 1; Label P1
617.2687
618.2692
633.2443
BocHN
OMe
7d
Mass (m/z)

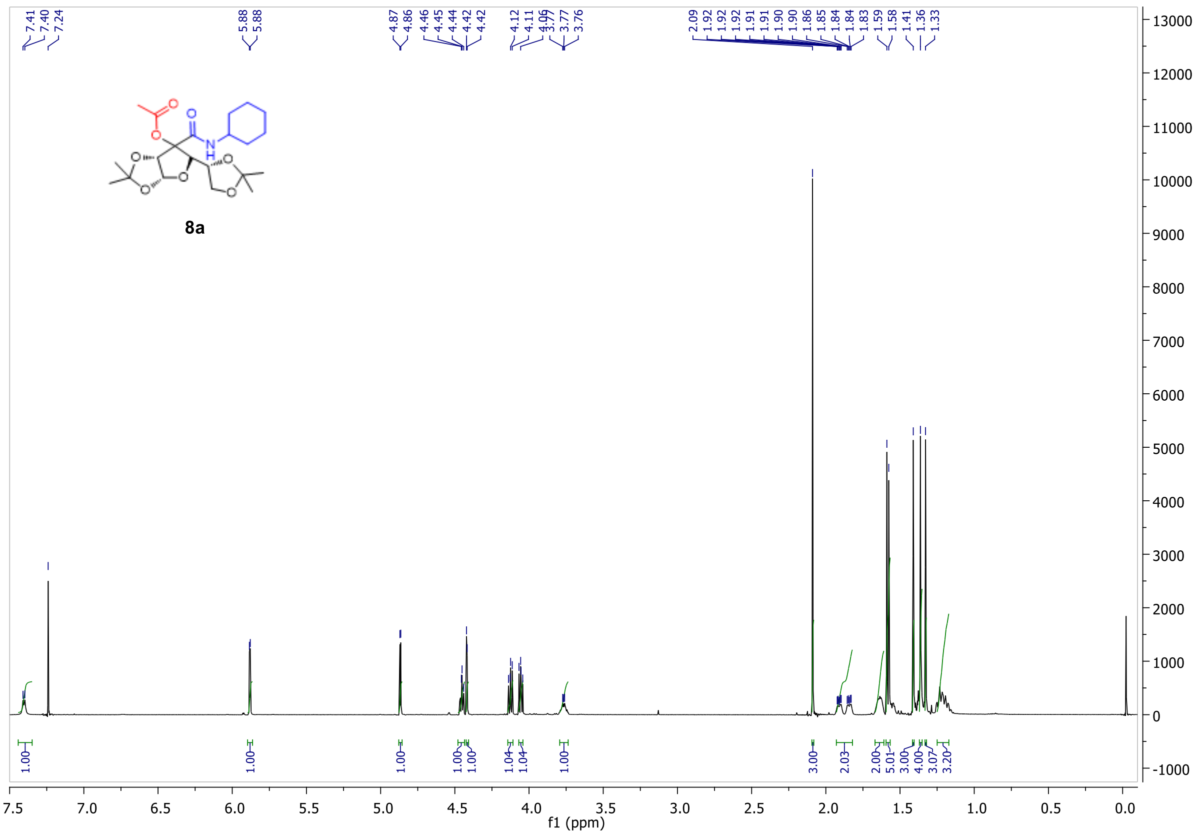
8a
f1 (ppm)

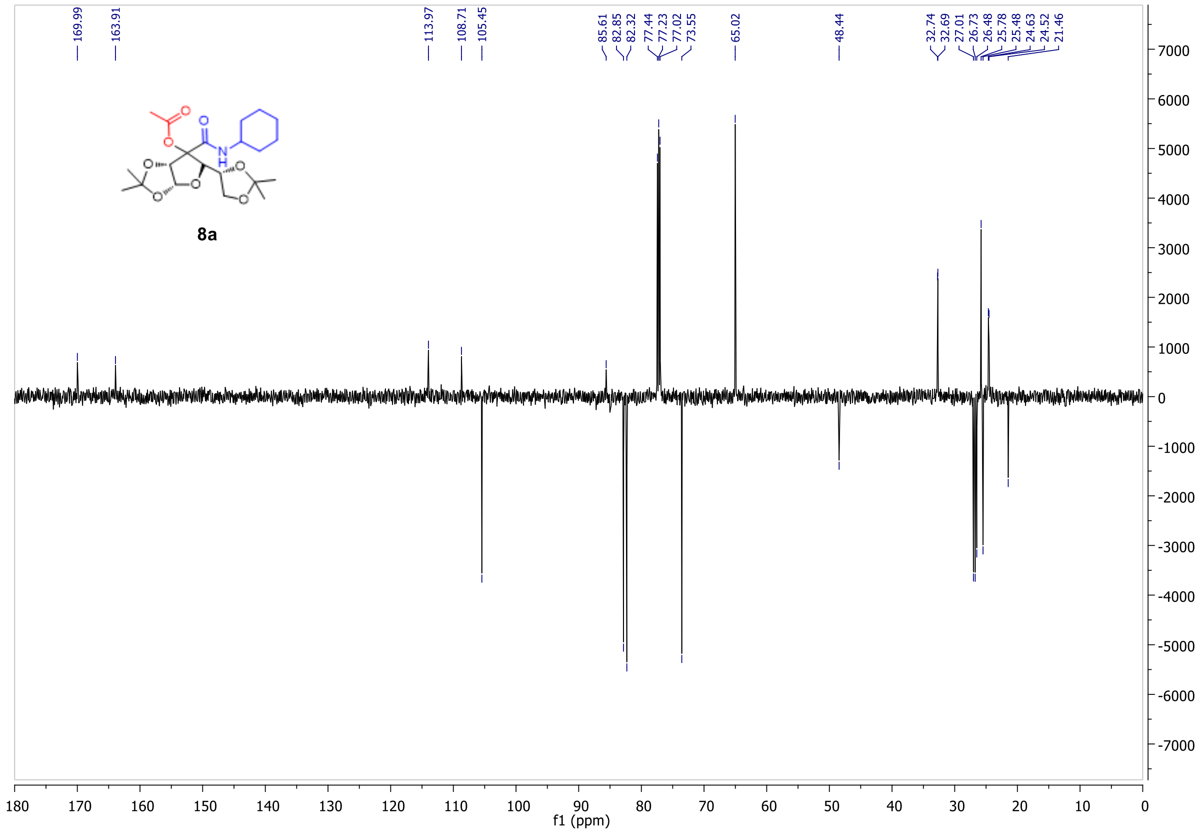
169.99
163.91
113.97
108.71
105.45
85.61
82.85
82.32
77.44
77.23
77.02
73.55
65.02
48.44
32.74
32.69
27.01
26.73
26.48
25.78
25.48
24.63
24.52
21.46
8a
f1 (ppm)

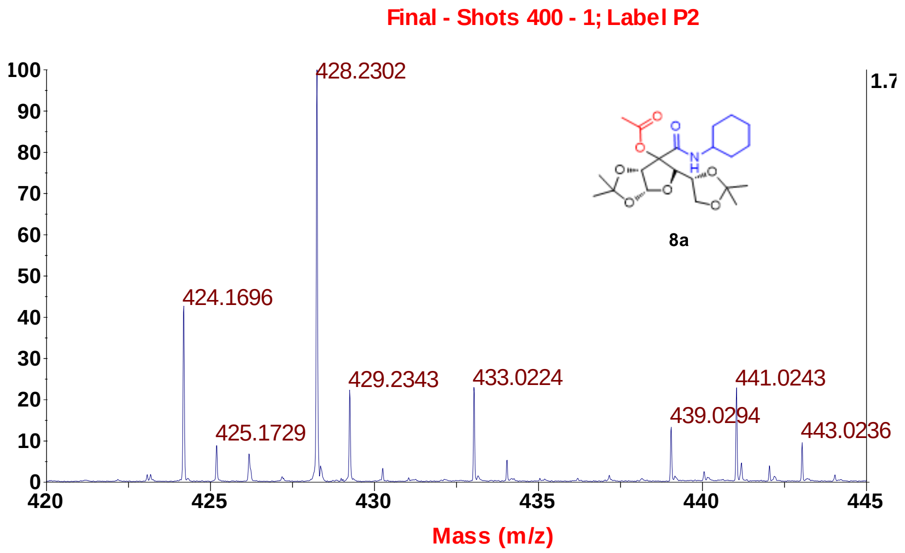
Final - Shots 400 - 1; Label P2
428.2302
424.1696
425.1729
429.2343
433.0224
439.0294
441.0243
443.0236
1.7
8a
Mass (m/z)

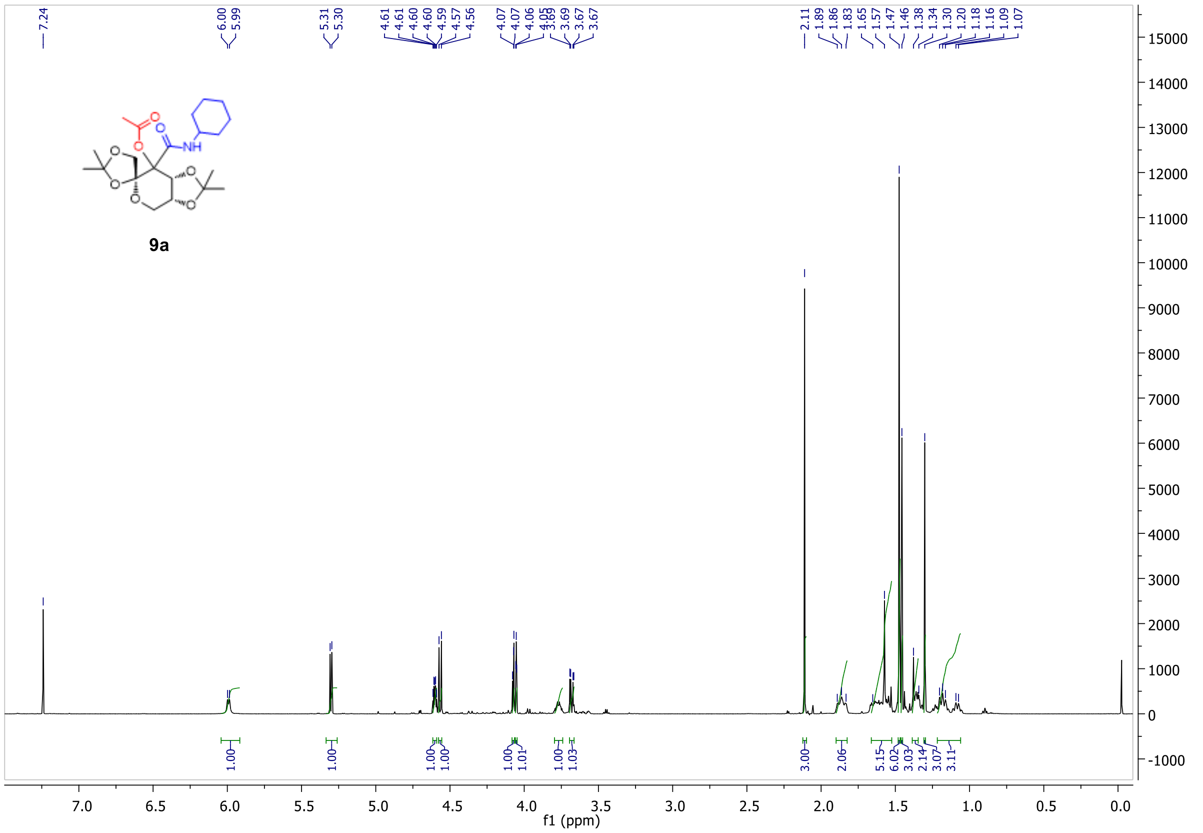

7.24
6.00
5.99
5.31
5.30
4.61
4.61
4.60
4.60
4.59
4.57
4.56
4.07
4.07
4.06
4.05
3.69
3.67
3.67
2.11
1.89
1.86
1.83
1.65
1.57
1.47
1.46
1.38
1.34
1.30
1.20
1.18
1.16
1.09
1.07
NH
9a
1.00
1.00
1.00
1.00
1.00
1.01
1.00
1.03
3.00
2.06
5.15
6.02
3.03
2.14
3.07
3.11
f1 (ppm)

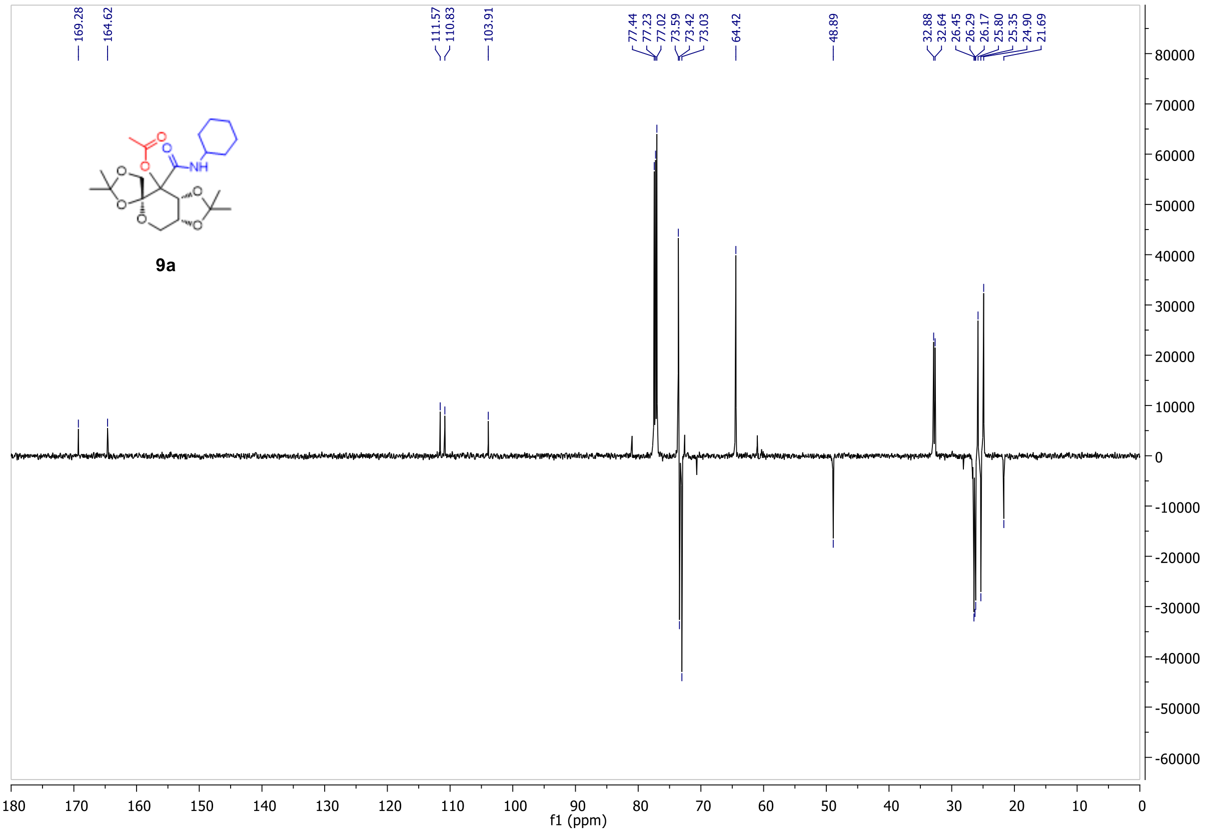

169.28
164.62
111.57
110.83
103.91
77.44
77.23
77.02
73.59
73.42
73.03
64.42
48.89
32.88
32.64
26.45
26.29
26.17
25.80
25.35
24.90
21.69
NH
9a
f1 (ppm)

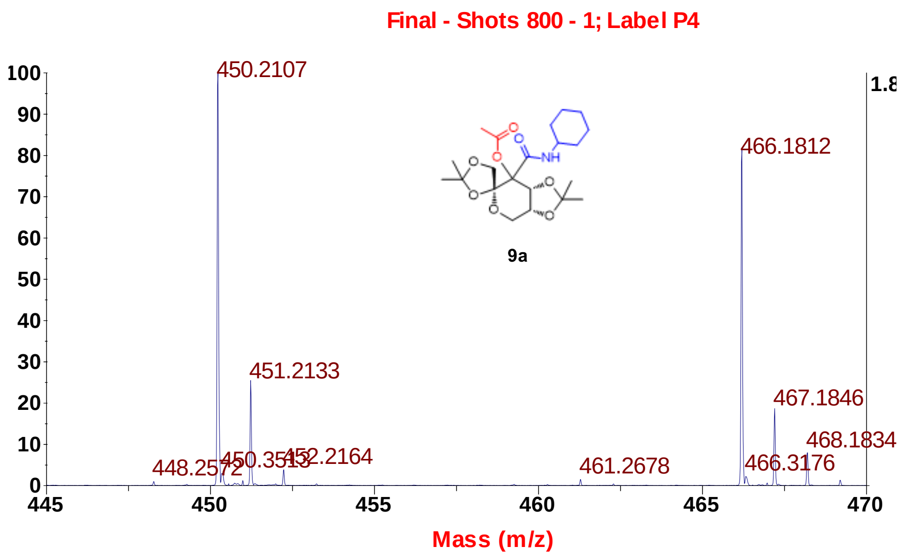
Final - Shots 800 - 1; Label P4
450.2107
466.1812
451.2133
467.1846
468.1834
448.2572
450.3513
452.2164
461.2678
466.3176
Mass (m/z)
9a

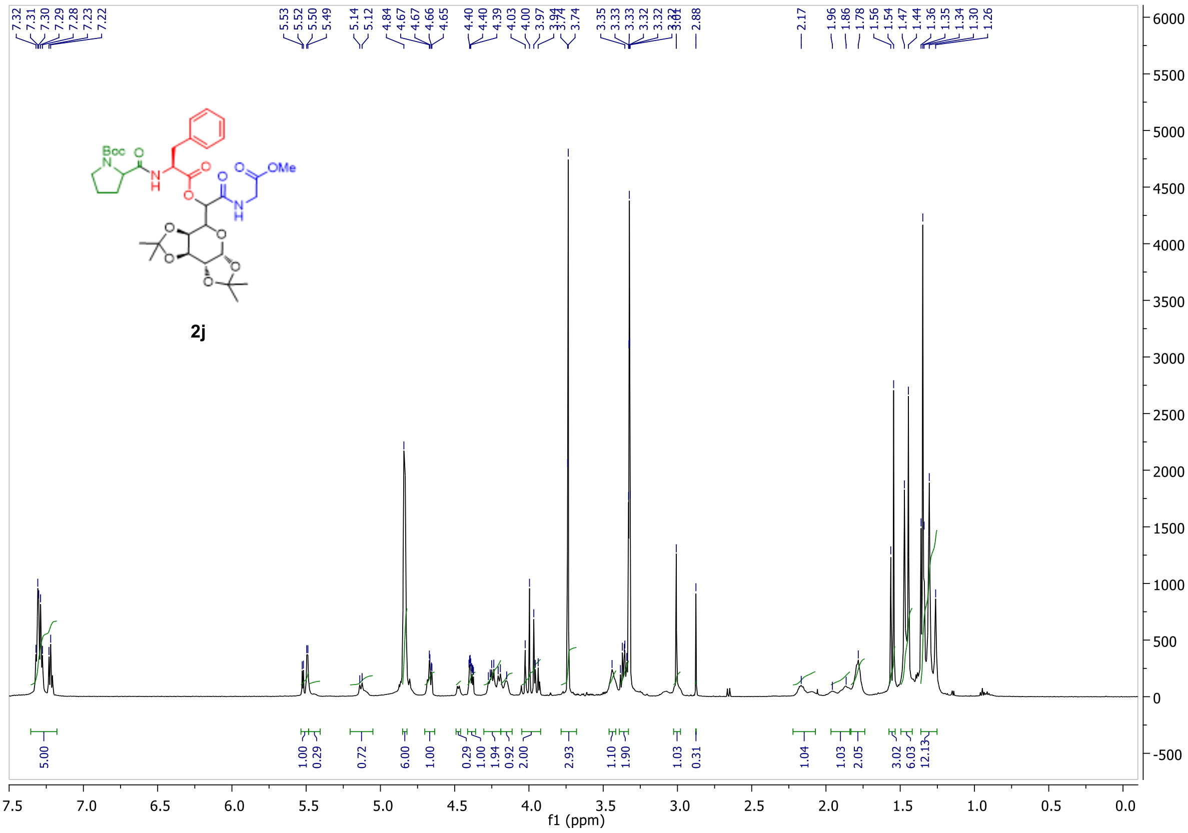
2j
f1 (ppm)

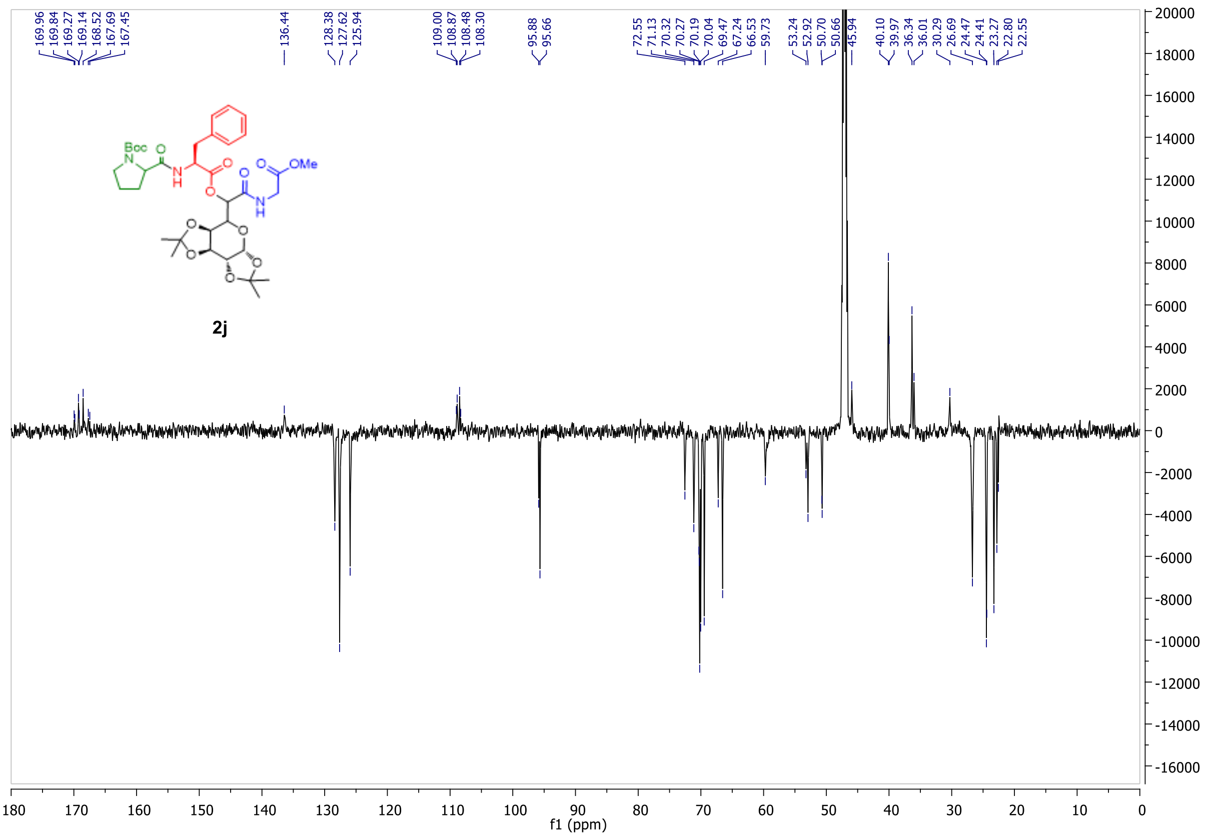
169.96
169.84
169.27
169.14
168.52
167.69
167.45
136.44
128.38
127.62
125.94
109.00
108.87
108.48
108.30
95.88
95.66
72.55
71.13
70.32
70.27
70.19
70.04
69.47
67.24
66.53
59.73
53.24
52.92
50.70
50.66
45.94
40.10
39.97
36.34
36.01
30.29
26.69
24.47
24.41
23.27
22.80
22.55
Boc
OMe
2j
f1 (ppm)

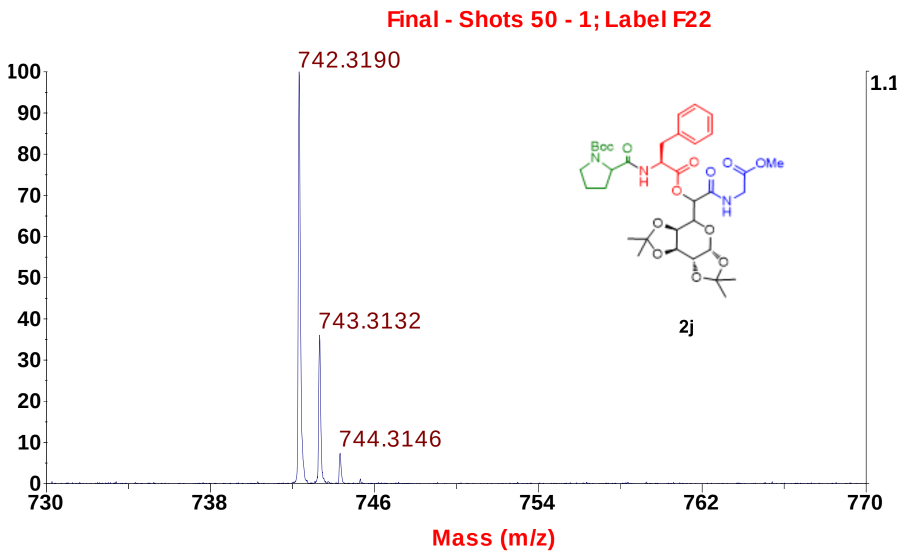
Final - Shots 50 - 1; Label F22
742.3190
743.3132
744.3146
1.1
Boc
OMe
2j
Mass (m/z)

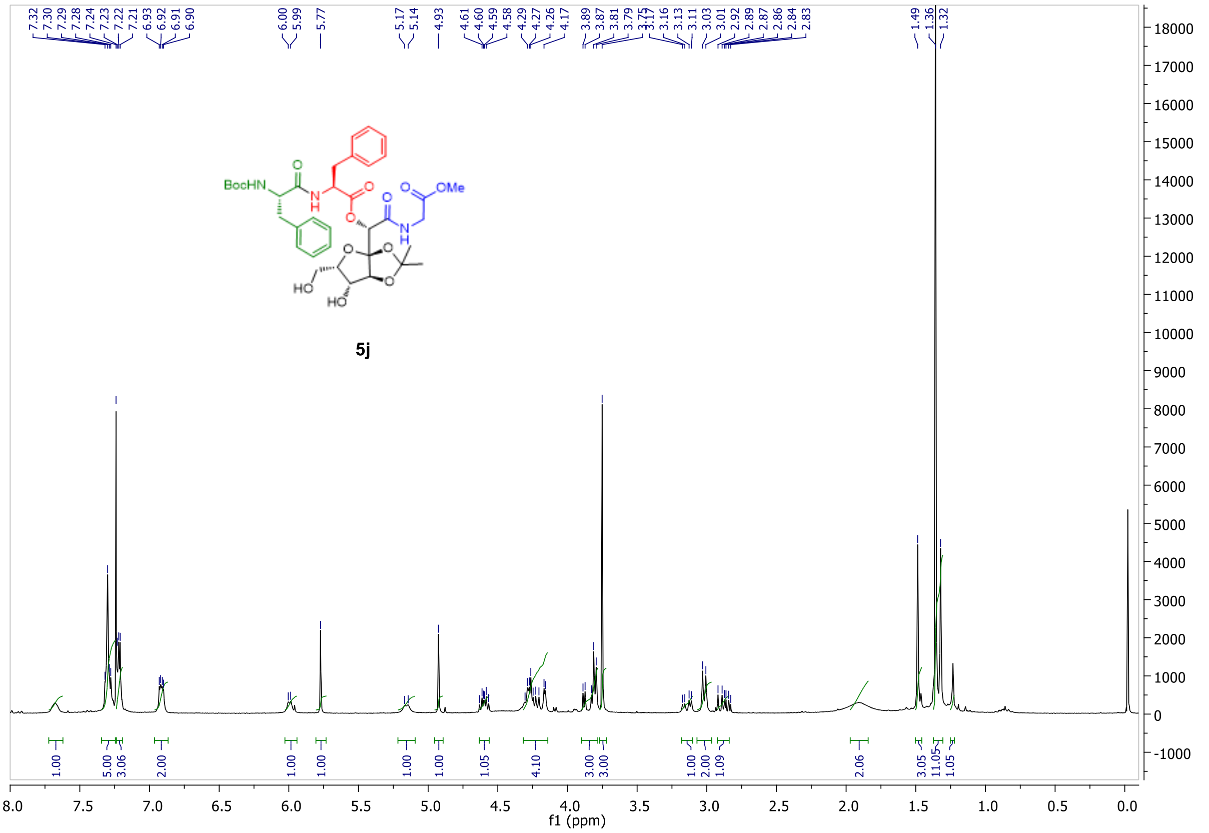

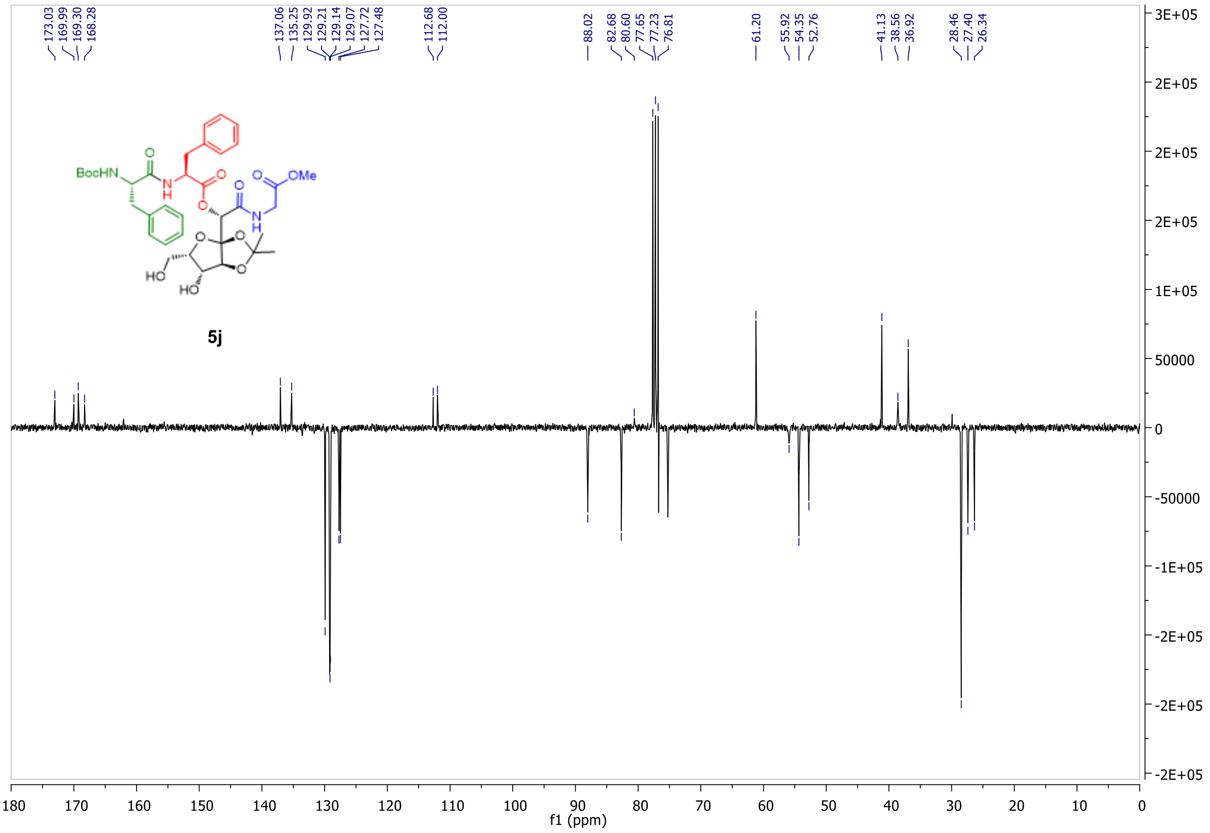

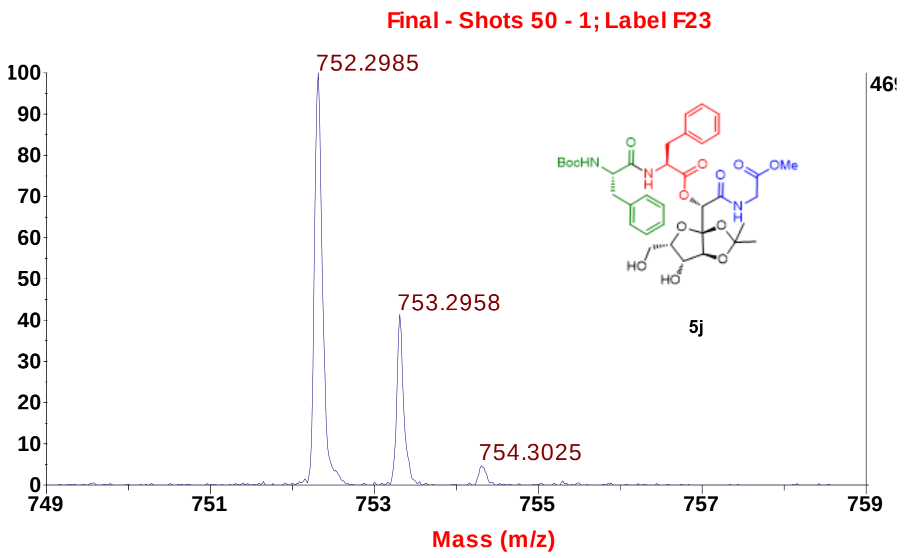

Final - Shots 50 - 1; Label F23
752.2985
753.2958
754.3025
BocHN
OMe
HO
5j
Mass (m/z)

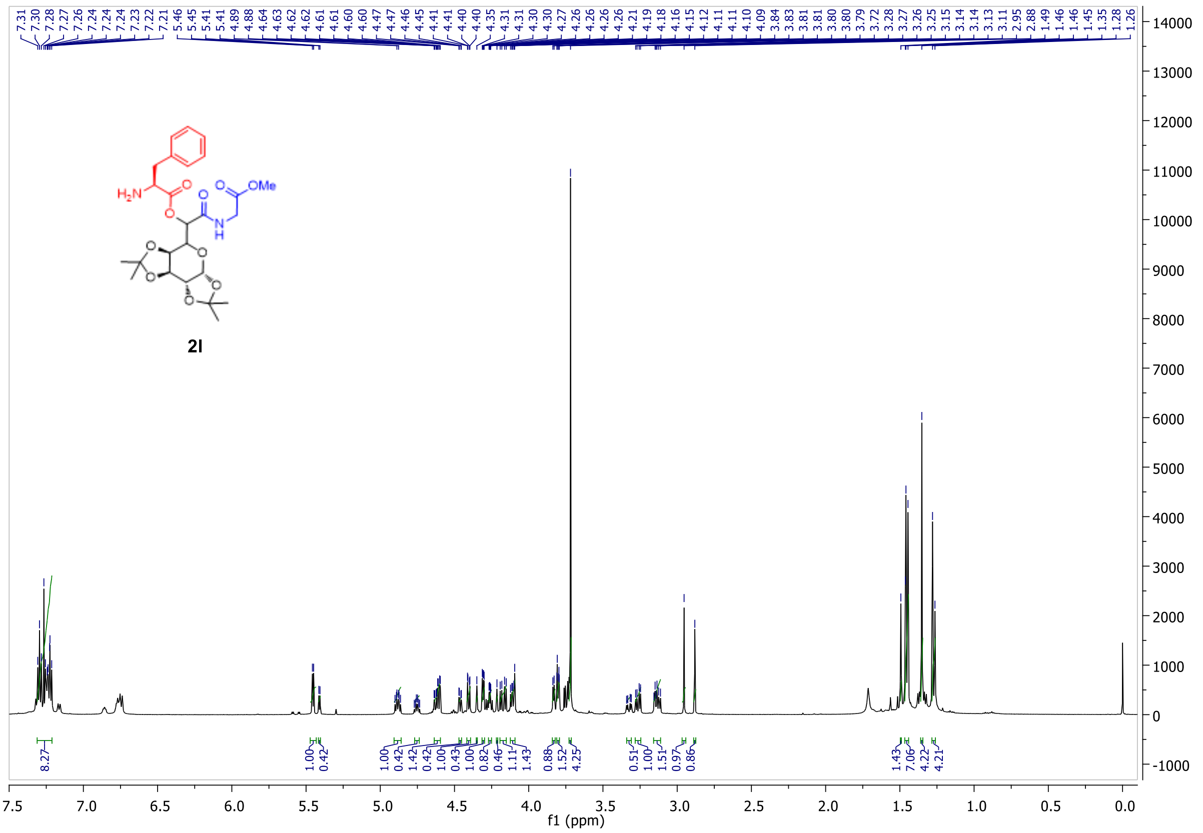

H_2N
OMe
2l
f1 (ppm)

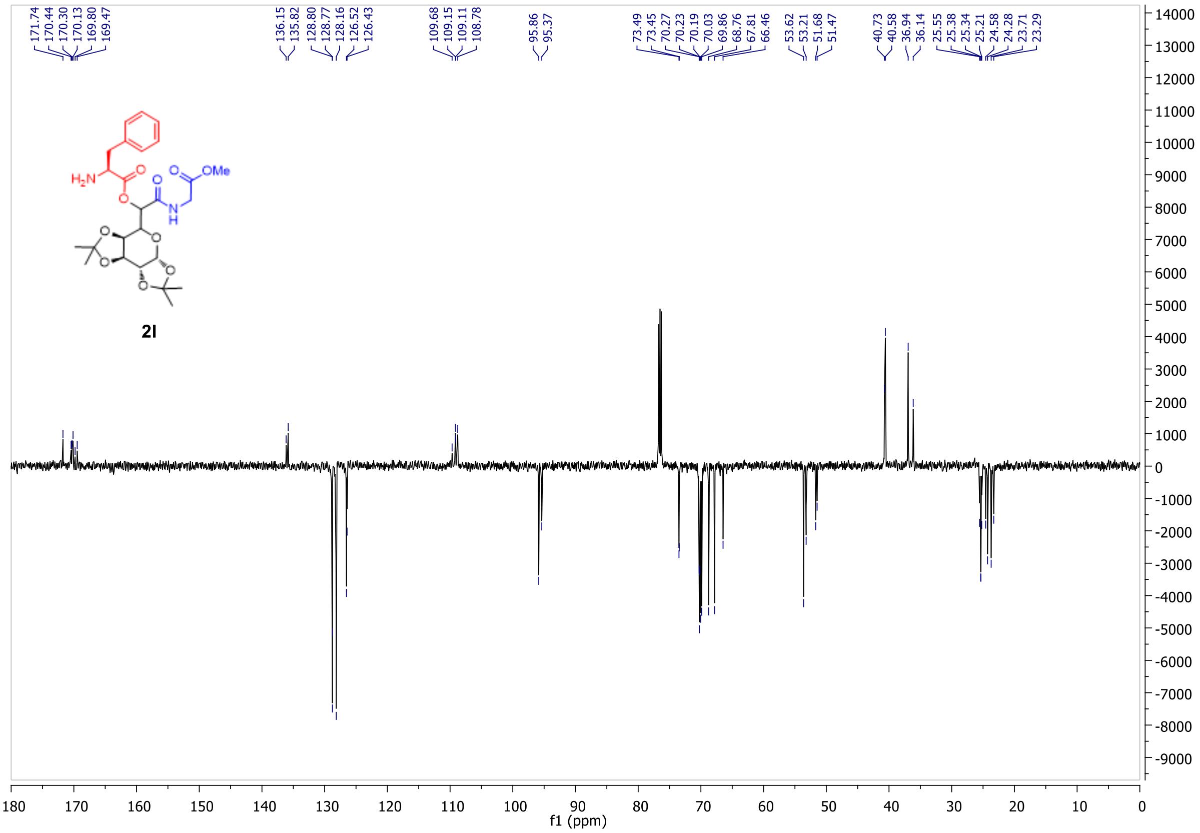

Final - Shots 50 - 1; Label F21

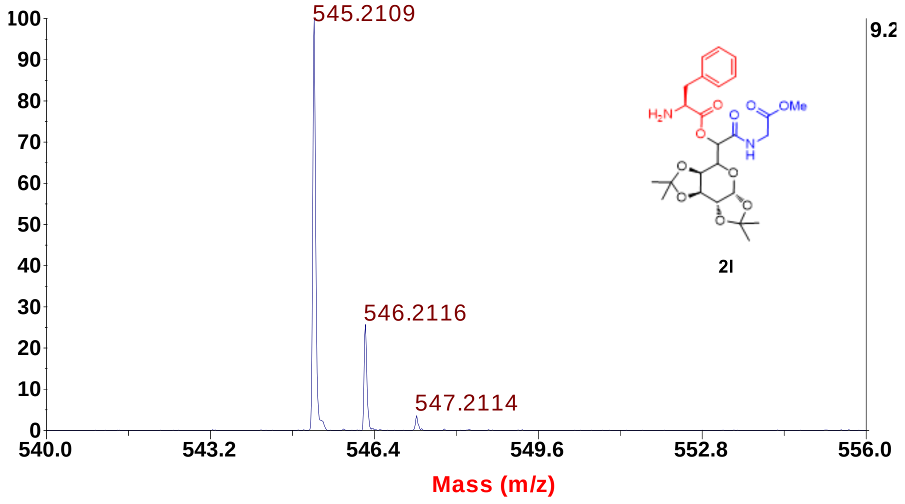

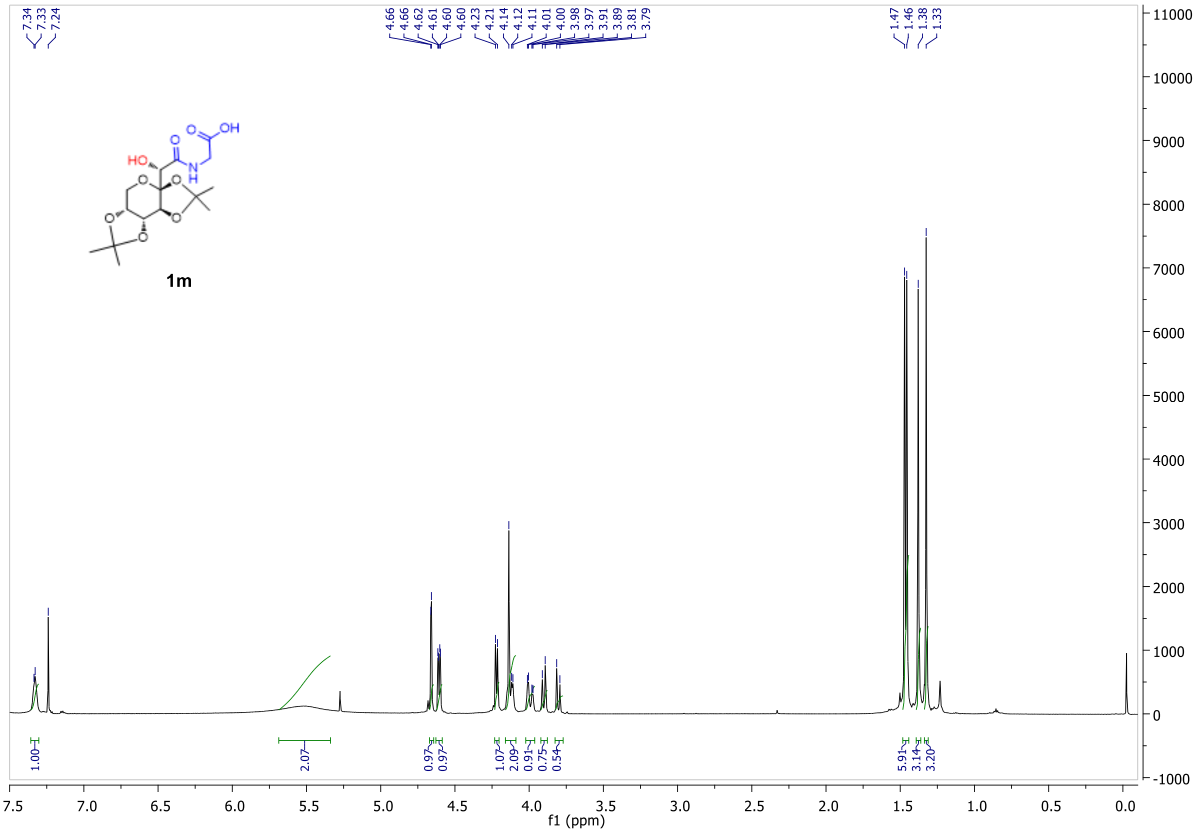

7.34
7.33
7.24
4.66
4.66
4.62
4.61
4.60
4.60
4.23
4.21
4.14
4.12
4.11
4.01
4.00
3.98
3.97
3.91
3.89
3.81
3.79
1.47
1.46
1.38
1.33
HO
O
OH
N
H
1m
1.00
2.07
0.97
0.97
1.07
2.09
0.91
0.75
0.54
5.91
3.14
3.20
f1 (ppm)

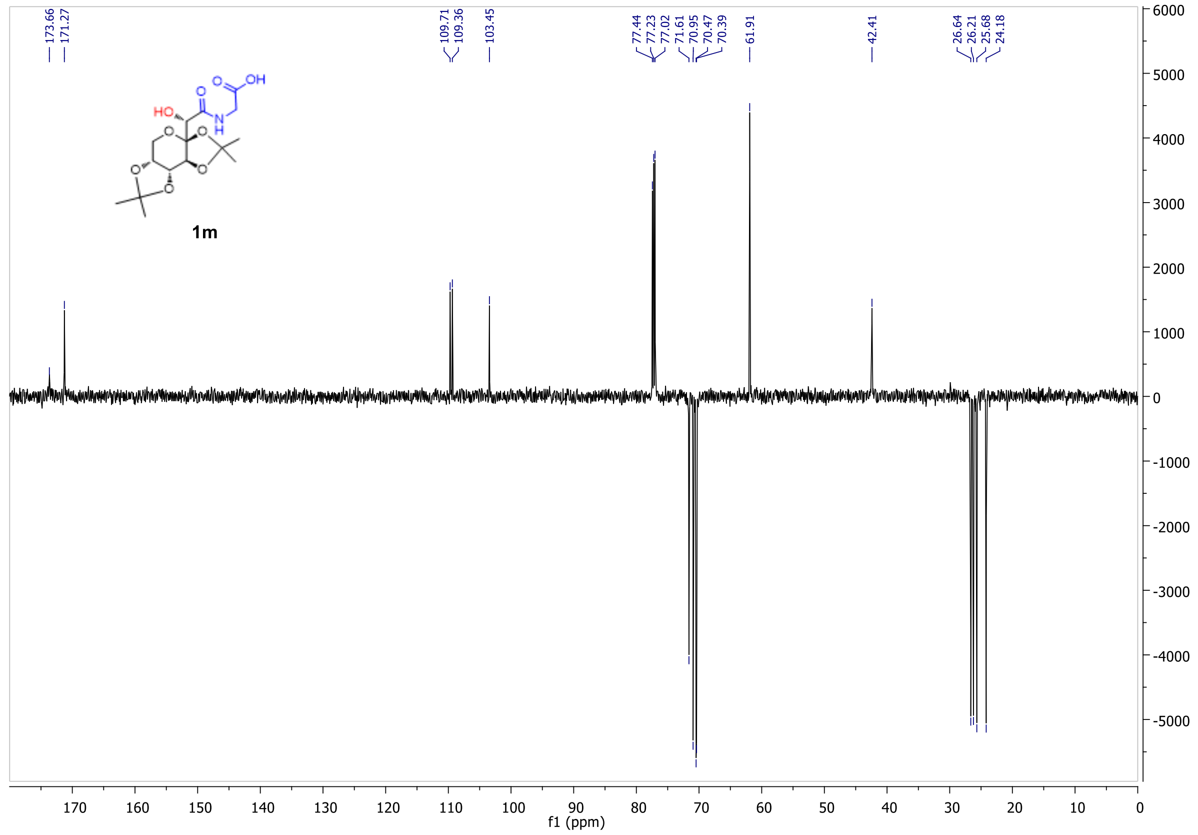

173.66
171.27
109.71
109.36
103.45
77.44
77.23
77.02
71.61
70.95
70.47
70.39
61.91
42.41
26.64
26.21
25.68
24.18
HO
O
OH
N
H
1m
f1 (ppm)

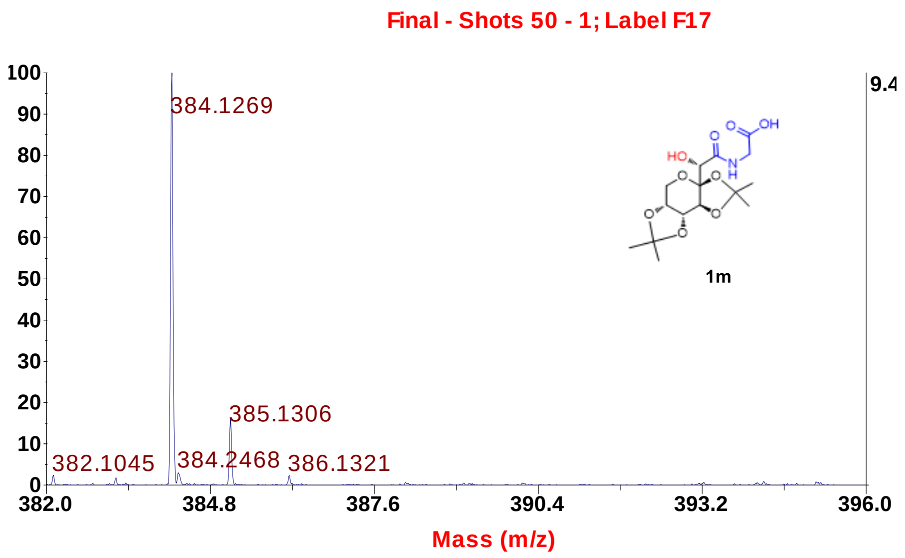
Final - Shots 50 - 1; Label F17
384.1269
385.1306
382.1045
384.2468
386.1321
9.4
Mass (m/z)
1m

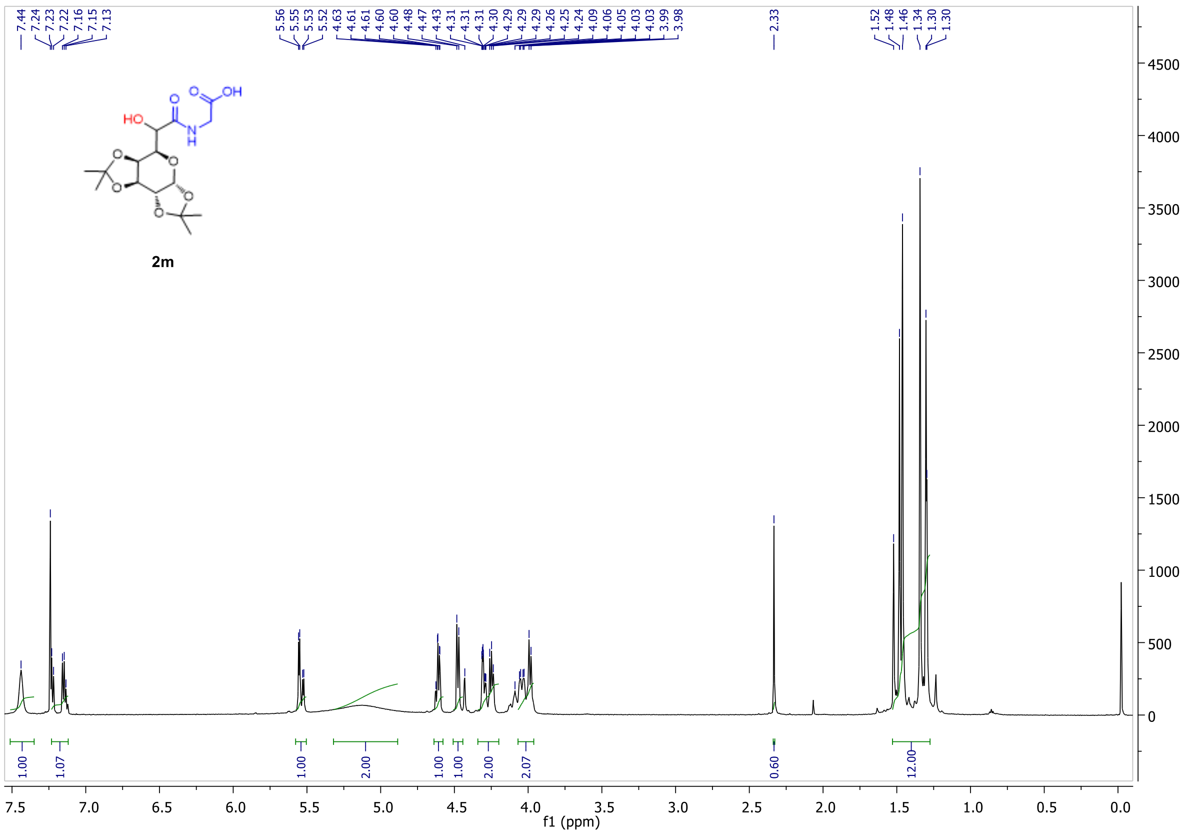
2m
f1 (ppm)

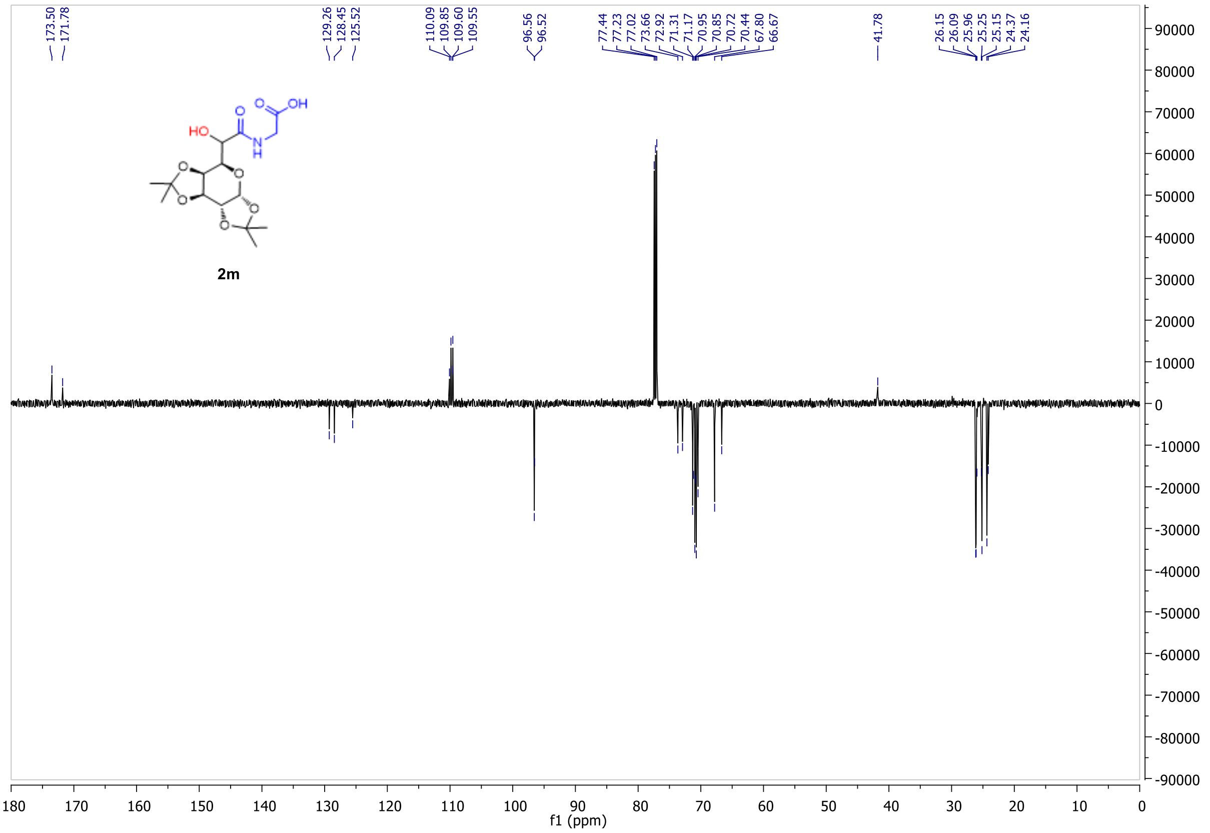
173.50
171.78
129.26
128.45
125.52
110.09
109.85
109.60
109.55
96.56
96.52
77.44
77.23
77.02
73.66
72.92
71.31
71.17
70.95
70.85
70.72
70.44
67.80
66.67
41.78
26.15
26.09
25.96
25.25
25.15
24.37
24.16
HO
O
O
OH
N
H
O
O
O
O
O
2m
f1 (ppm)

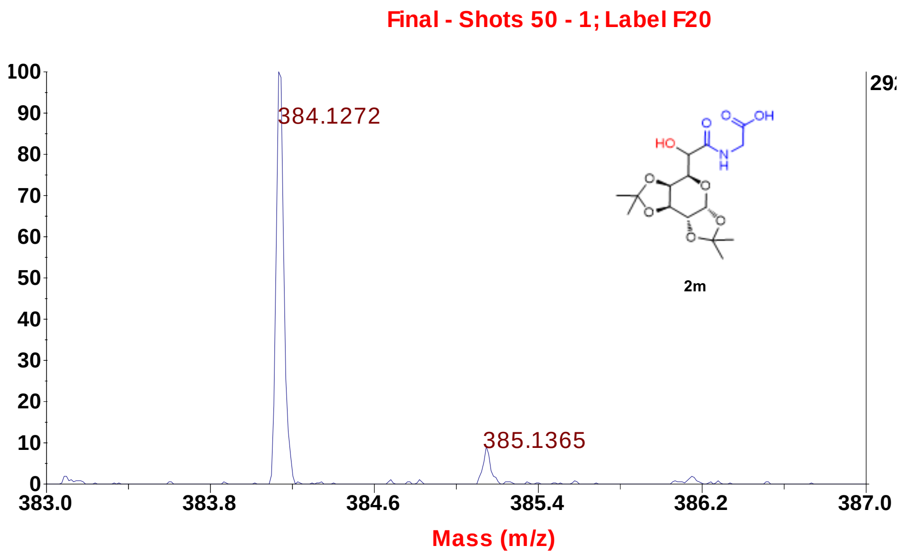
Final - Shots 50 - 1; Label F20
384.1272
385.1365
HO
O
O
OH
N
H
O
O
O
O
O
2m
Mass (m/z)

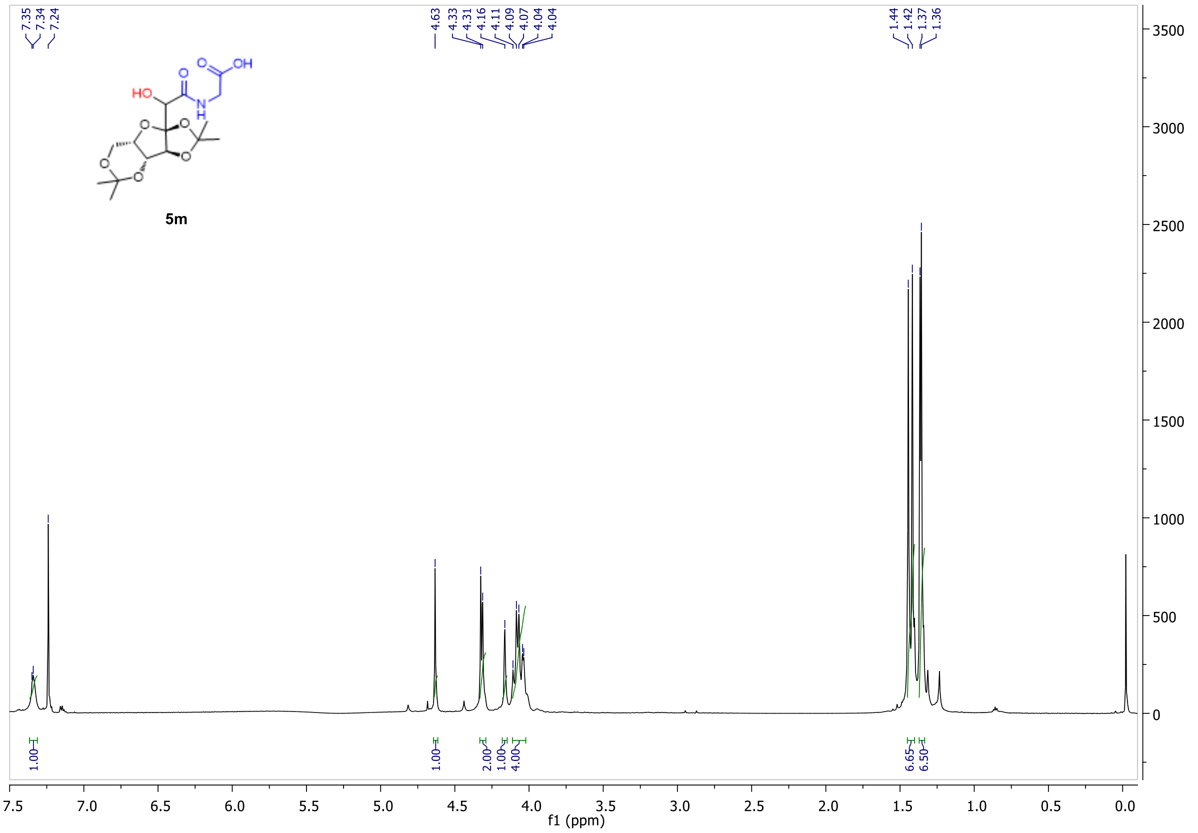
7.35
7.34
7.24
4.63
4.33
4.31
4.16
4.11
4.09
4.07
4.04
4.04
1.44
1.42
1.37
1.36
HO
O
N
H
O
OH
O
O
O
O
O
5m
1.00
1.00
2.00
1.00
4.00
6.65
6.50
f1 (ppm)

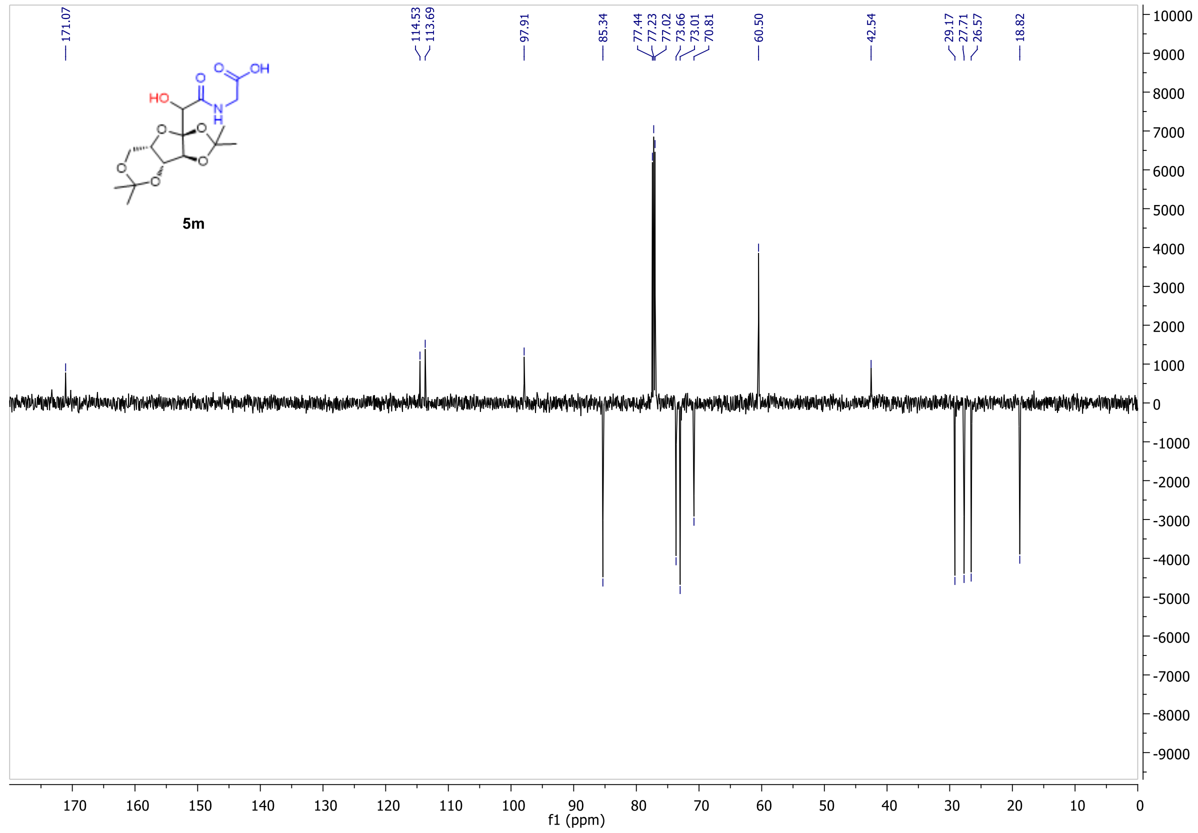
171.07
114.53
113.69
97.91
85.34
77.44
77.23
77.02
73.66
73.01
70.81
60.50
42.54
29.17
27.71
26.57
18.82
HO
O
N
H
O
OH
5m
f1 (ppm)

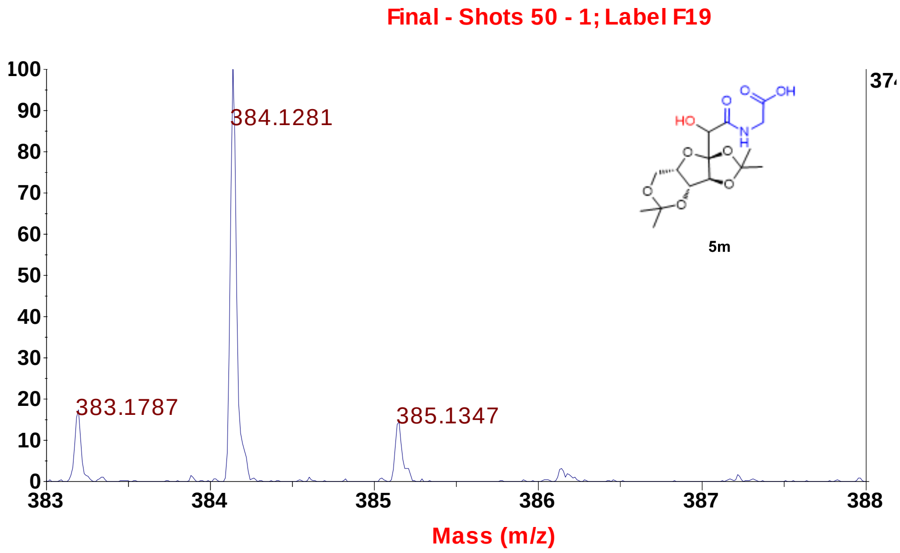
Final - Shots 50 - 1; Label F19
384.1281
383.1787
385.1347
5m
Mass (m/z)

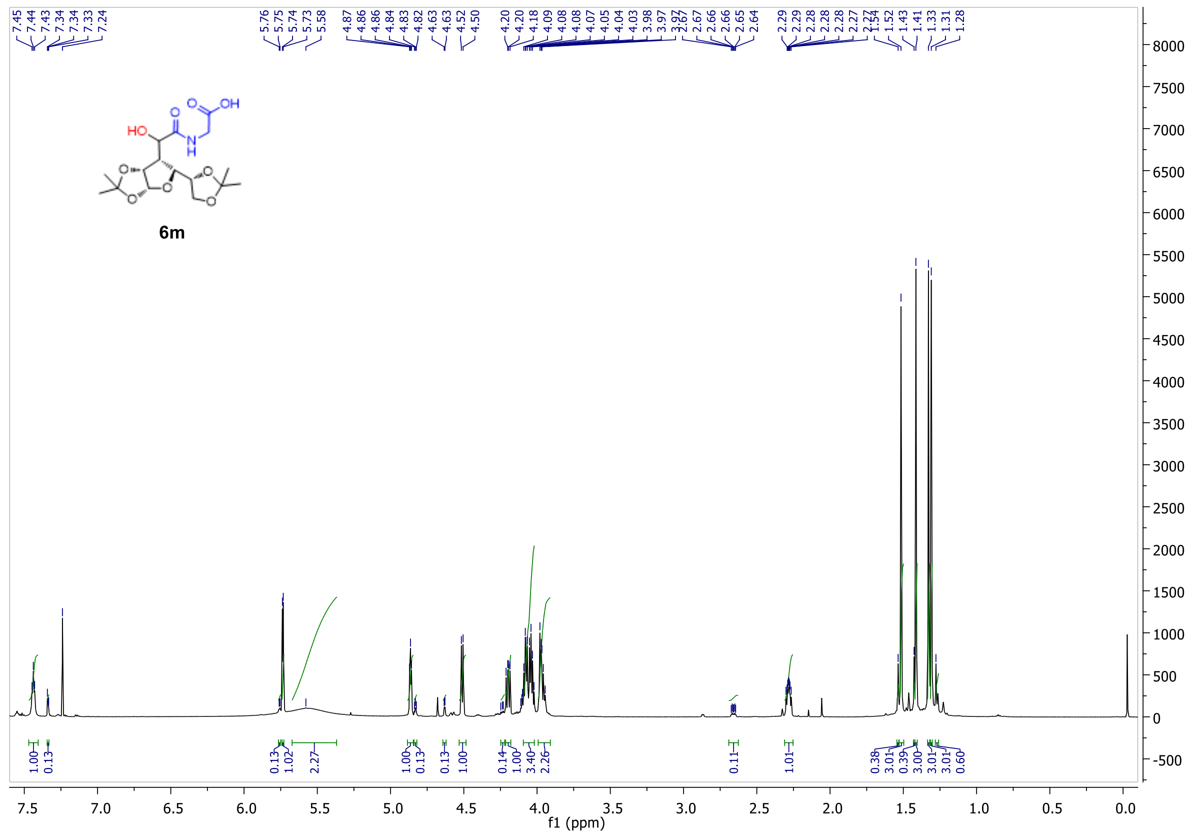
6m
f1 (ppm)

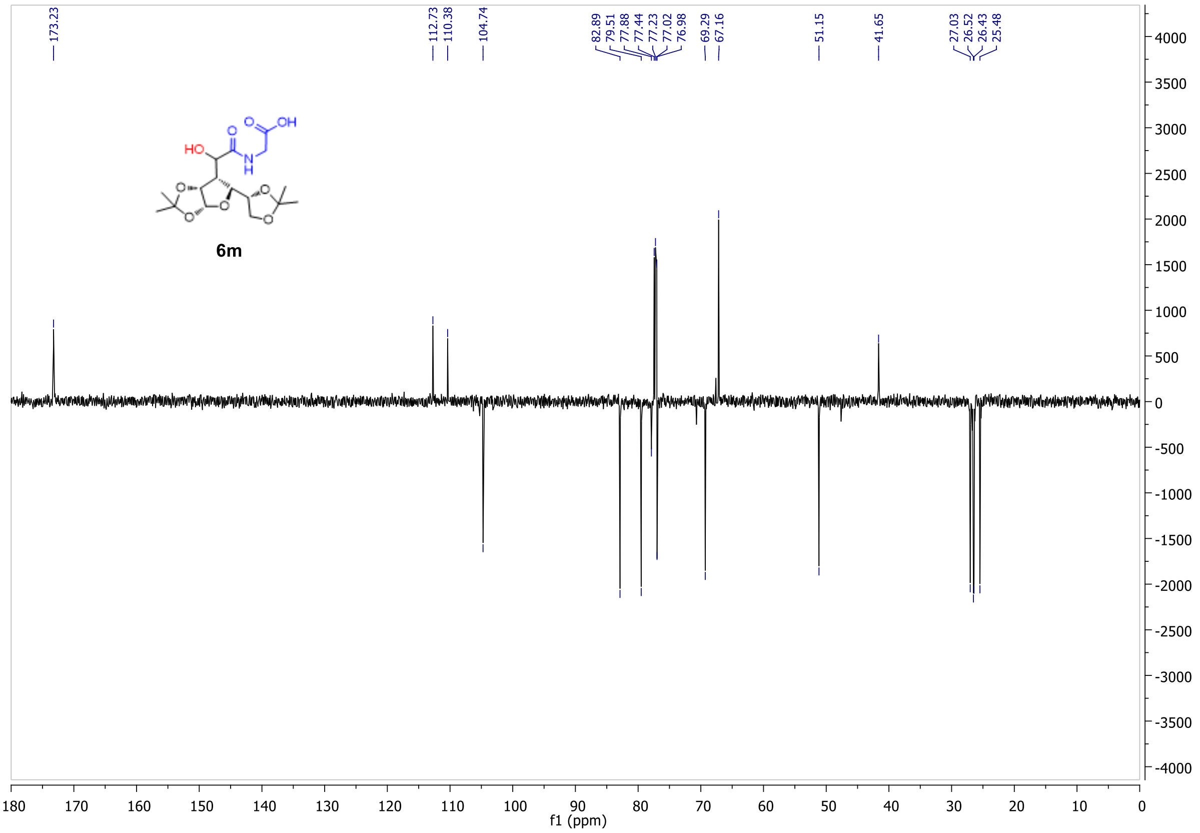
173.23
112.73
110.38
104.74
82.89
79.51
77.88
77.44
77.23
77.02
76.98
69.29
67.16
51.15
41.65
27.03
26.52
26.43
25.48
HO
O
OH
N
H
6m
f1 (ppm)

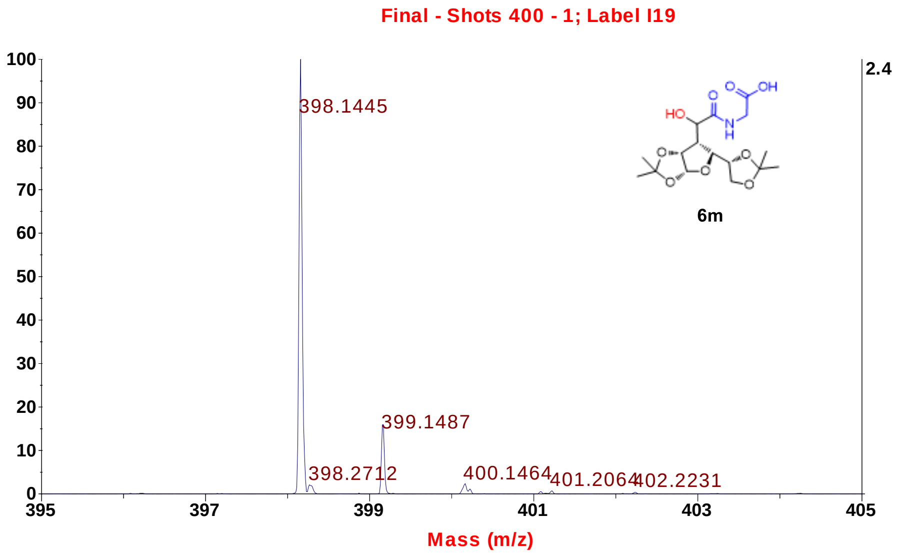
Final - Shots 400 - 1; Label I19
398.1445
399.1487
398.2712
400.1464
401.2064
402.2231
2.4
HO
O
OH
N
H
6m
Mass (m/z)

X-ray structure – compound 2a

Crystal data, data collection and refinement parameters are summarized in **Error! Reference source not found.**1. Crystal were prepared by a slow evaporation from from *n*-hexane/*i*-PrOH (9/1, v/v) solution. Data collections was performed at room temperature on an Oxford Diffraction Xcalibur Nova R diffractometer with a microfocusing Cu tube (λ = 1.54179 Å). Data reduction and cell refinement were carried out using the CRYSALIS PRO software[1]. Structures were solved by direct methods with SIR2014[2] and refined by a full matrix least-squares refinement based on F^2, with SHELX.[3] Molecular illustrations were prepared with MERCURY[4] included into the WinGX package.[5] Calculations of molecular geometries and crystal packing parameters were performed with PLATON.[6] Hydrogen atoms were either included in their geometrically calculated positions and refined according to the riding model, or located in the Fourier map and refined freely.

CCDC 1865101 contain the supplementary crystallographic data. These data can be obtained free of charge *via* http://www.ccdc.cam.ac.uk/conts/retrieving.html, or from the Cambridge Crystallographic Data Centre, 12 Union Road, Cambridge CB2 1EZ, UK; fax:(+44) 1223-336-033; or e-mail: deposit@ccdc.cam.ac.uk.

Table S1. Crystal data and structure refinement results for compound **2a**.

Brutto formula	$C_{21}H_{33}NO_8$
Mol. weight (gmol^{-1})	427.48
Crystal colour and habit	colourless plate
Crystal dim. (mm)	0.18 x 0.14 x 0.04
Space group	P1 (triclinic)
a (Å)	5.3974(15)
b (Å)	10.320(2)
c (Å)	10.9130(17
V (Å^3)	565.6(2)
[illegible]	1
[illegible] (Cu*K*[illegible]) (mm^{-1})	0.798
Absorption correction	multi-scan
F(000)	230
θmax (°)	70.582
No. refl. measured	4362
No. refl. unique	3283

[1] CrysAlis CCD, Oxford Diffraction Ltd., Version 1.171.32.29 (release 10-02008 CrysAlis171.NET).

[2] Burla, M. C.; Caliandro, R.; Carrozzini, B.; Cascarano, G. L.; Cuocci, C.; Giacovazzo, C.; Mallamo, M.; Mazzone, A.; Polidori, G. Crystal structure determination and refinement *via SIR2014*. *J. Appl. Cryst.* **2015**, *48*, 306-309.

[3] Sheldrick, G. M. SHELX97: Program for the Refinement of Crystal Structures, Universität Göttingen, Germany, 1997.

[4] Macrae, C. F.; Bruno, I. J.; Chisholm, J. A.; Edgington, P. R.; McCabe, P.; Pidcock, E.; Rodriguez-Monge, L.; Taylor, R.; van de Streek, J.; Wood, P. A. *Mercury CSD 2.0* - new features for the visualization and investigation of crystal structures. *J. Appl. Cryst.* **2008**, *41*, 466-470.

[5] Faruggia, L. J. *WinGX* suite for small-molecule single-crystal crystallography. *J. Appl. Cryst.* **1999**, *32*, 837-838.

[6] Spek, A. L. Structure validation in chemical crystallography *Acta Cryst.* **2009**, *D65*, 148-155.

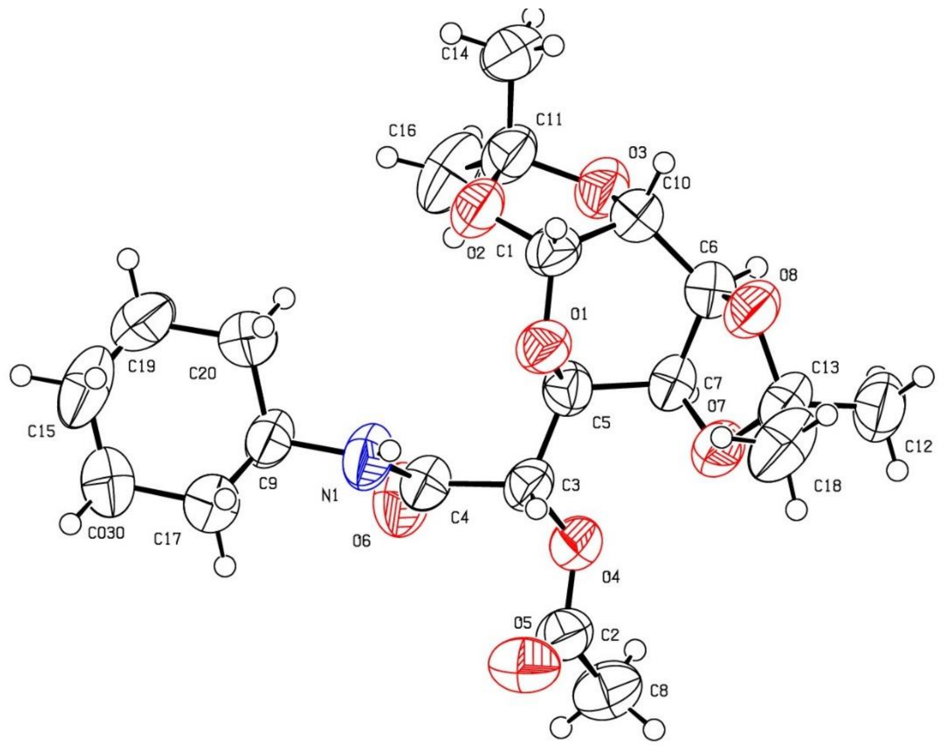

Figure S1. Figure captions: ORTEP drawing of the structure **2a** with the atom numbering. Thermal ellipsoids are scaled to a 50 % probability level.
